# Design, Synthesis, Molecular Docking, Antiapoptotic and Caspase-3 Inhibition of New 1,2,3-Triazole/*Bis*-2(1*H*)-Quinolinone Hybrids

**DOI:** 10.3390/molecules25215057

**Published:** 2020-10-30

**Authors:** Essmat M. El-Sheref, Ashraf A. Aly, Mohammed B. Alshammari, Alan B. Brown, Sara Mohamed Naguib Abdel-Hafez, Walaa Yehia Abdelzaher, Stefan Bräse, ElShimaa M. N. Abdelhafez

**Affiliations:** 1 Chemistry Department, Faculty of Science, Minia University, El-Minia 61519, Egypt; e.m.elsheref1980@gmail.com; 2 College of Sciences and Humanities, Prince Sattam bin Abdulaziz University, Alkharj 11942, Saudi Arabia; m.alshammari@psau.edu.sa; 3 Florida Institute of Technology, 150 W University Blvd, Melbourne, FL 32901, USA; abrown@fit.edu; 4 Faculty of Medicine, Histology Department, Minia University, El-Minia 61519, Egypt; sara_histology@yahoo.com; 5 Faculty of Medicine, Pharmacology Department, Minia University, El-Minia 61519, Egypt; walaayehia22@yahoo.com; 6 Institute of Organic Chemistry, Karlsruhe Institute of Technology, 76131 Karlsruhe, Germany; 7 Institute of Biological and Chemical Systems (IBCS-FMS), Karlsruhe Institute of Technology, 76344 Eggenstein-Leopoldshafen, Germany; 8 Department of Medicinal Chemistry, Faculty of Pharmacy, Minia University, El-Minia 61519, Egypt; shimaanaguib_80@yahoo.com

**Keywords:** caspase-3, 1,2,3-triazole-biquinolin-2-one, antiapoptotic, NAC, histopathology, antioxidant, testis, docking

## Abstract

A series of novel 1,2,3-triazoles hybridized with two quinolin-2-ones, was designed and synthesized through click reactions. The structures of the synthesized compounds were elucidated by NMR, IR, and mass spectra in addition to elemental analysis. The synthesized compounds were assessed for their antiapoptotic activity in testis, as testicular torsion is the main cause of male infertility. This effect was studied in light of decreasing tissue damage induced by I/R in the testis of rats using *N*-acetylcysteine (NAC) as an antiapoptotic reference. Compounds **6a**–**c** were the most active antiapoptotic hybrids with significant measurements for malondialdehyde (MDA) and total antioxidant capacity (TAC) and the apoptotic biomarkers (testicular testosterone, TNFα, and caspase-3) in comparison to the reference. A preliminary mechanistic study was performed to improve the antiapoptotic activity through caspase-3 inhibition. A compound assigned as 6-methoxy-4-(4-(((2-oxo-1,2-dihydroquinolin-4-yl)oxy)methyl)-1*H*-1,2,3-triazol-1-yl)quinolin-2(1*H*)-one (**6c**) was selected as a representative of the most active hybrids in comparison to NAC. Assay of cytochrome *C* for **6c** revealed an attenuation of cytochrome *C* level about 3.54 fold, comparable to NAC (4.13 fold). In caspases-3,8,9 assays, **6c** was found to exhibit more potency and selectivity toward caspase-3 than other caspases. The testicular histopathological investigation was carried out on all targeted compounds **6a**–**g**, indicating a significant improvement in the spermatogenesis process for compounds **6a**–**c** if compared to the reference relative to the control. Finally, molecular docking studies were done at the caspase-3 active site to suggest possible binding modes. Hence, it could conceivably be hypothesized that compounds **6a**–**c** could be considered good lead candidate compounds as antiapoptotic agents.

## 1. Introduction

Apoptosis is an essential programmed biological process in cells during animal development, [1] homeostasis [2], and immune responses [3]. Although apoptosis occurs normally in tissues, imbalance probably occurs in some cases between apoptotic and antiapoptotic mediators leading to some diseases [4]. Apoptosis could be abrogated, which means cells that should be eliminated may persist and become immortal, for example, in cancer [5] and autoimmunity [6]. On the other hand, when apoptosis exaggerated, it kills too many cells and inflicts grave tissue damage; as in strokes [7], neurodegenerative disorders such as Alzheimer’s [8], retinal cell death [9], myocardial and testicular ischemia [10,11] inflammatory diseases such as rheumatoid arthritis [12], sepsis [13], osteoarthritis (OA) [14], and asthma [15].

The caspase family has been an intensely studied target in drug design for many years [1]. To date, 14 mammalian caspases were identified, among them, caspase -2, -3, -6, -7, -8, -9, and -10, which were described as apoptotic caspases [1]. Interestingly, caspase-3 was found to be generally more promiscuous than caspase-7, 8, and 9 and revealed the major executioner caspase during the demolition phase of apoptosis [16,17]. Additionally, caspase-3 plays a key executioner role and its inhibition can drastically prevent apoptosis in vitro and in vivo [1].

Currently, one of the urological emergency conditions in male infertility is testicular torsion, which is common in new-born kids, children, and adolescent boys [18,19]. Testicular torsion, or a twisted spermatic cord, decreases the blood flow to the testis, causing tissue ischemia and a loss of germ cells [20]. Previous studies have shown that the pathophysiology of testicular torsion/detorsion centers around acute ischemia/reperfusion (I/R) injury of the testis resulting in the possibility of infertility whereas torsion comprises the ischemic period, while detorsion comprises reperfusion injury [21]. Therefore, several studies have been revealed that testicular torsion/detorsion or (I/R) injury triggers the generation of reactive oxygen species, proinflammatory cytokines, anoxia, and apoptosis, which is followed by activation of the necrosis/apoptosis pathway, possibly causing subsequent infertility [22].

On the other hand, the discovery of quinolone-based drugs has attracted wide attention due to their diverse spectrum of biological activities [23] as an antimicrobial effect [24,25]. Arylidenes of the quinolin-2-one scaffold with anticancer activity was working as Erlotinib analogs with activities against leukemia through inhibition of EGFR TK/STAT-3 pathways [26], however, other quinoline-2-one/pyrazole derivatives exhibited antiapoptotic activity with caspase-3 inhibition [1].

The promising application of triazoles in drug design such as anti-tuberculosis [27], anticancer [28], and the lead compound, SN606078, 2-(2,6-dichloro-4-trifluoromethylphenyl)-4-(4,5-dicyano-1*H*-imidazol-2-yl)-2*H*-l,2,3-triazoIe acting as antagonists of GABA receptors [29,30] is further propelled by the advent of click chemistry in the synthesis of triazoles [31]. 

**Figure 1:**
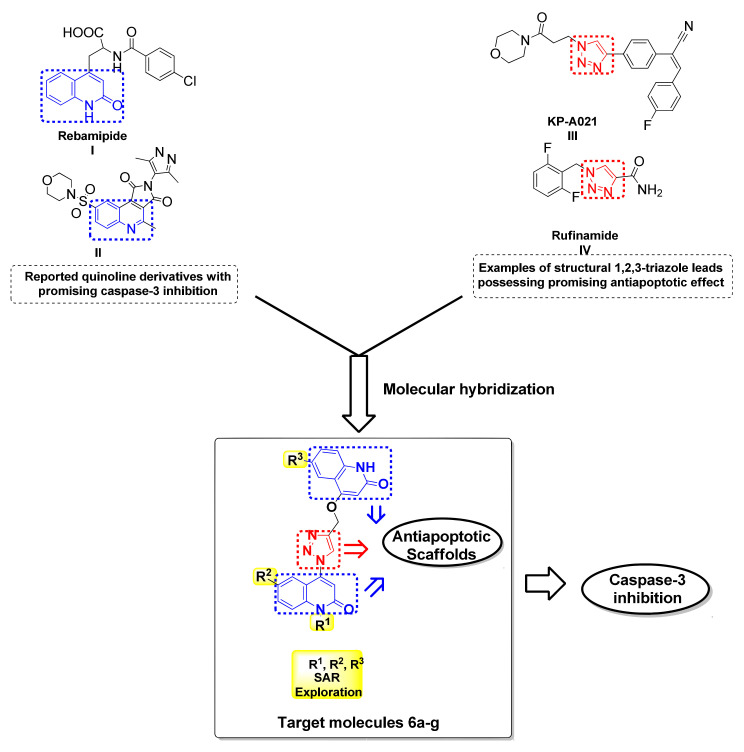
Examples of reported quinoline and 1,2,3-triazole based antiapoptotic drugs and caspase-3 and the design strategy for the target hybrids **6a**–**g**.

Recently, there have been several reports of potential therapeutic agents comprising either quinoline or triazole scaffold each in a discrete molecule, which were investigated against (I/R) injury through apoptosis inhibition. In 2003, Kojima et al. [32] reported on Rebamipide **I** as an antioxidative agent able to attenuate the elevation in apoptosis in the intestinal mucosa after I/R through caspase-3 inhibition, but not to completely abolish it [32]. This can be seen also in the case of compound **II**, a quinoline containing scaffold that exhibited antiapoptotic activity through inhibition of caspase-3 with an IC_50_ = 4 nM (Figure 1) [1].

Additionally, the 1,2,3-triazole-based scaffold was reported for KPRA020 **III** to be used as an antioxidant and antiapoptotic agent to prevent ototoxicity caused by cisplatin-induced oxidative stress [33]. Furthermore, the results demonstrated by Park et al. suggested that Rufinamide **V** can display a neuroprotective effect against cerebral ischemia involving the attenuation of ischemia-induced glial activation [34] (Figure 1).

**Figure 2:**
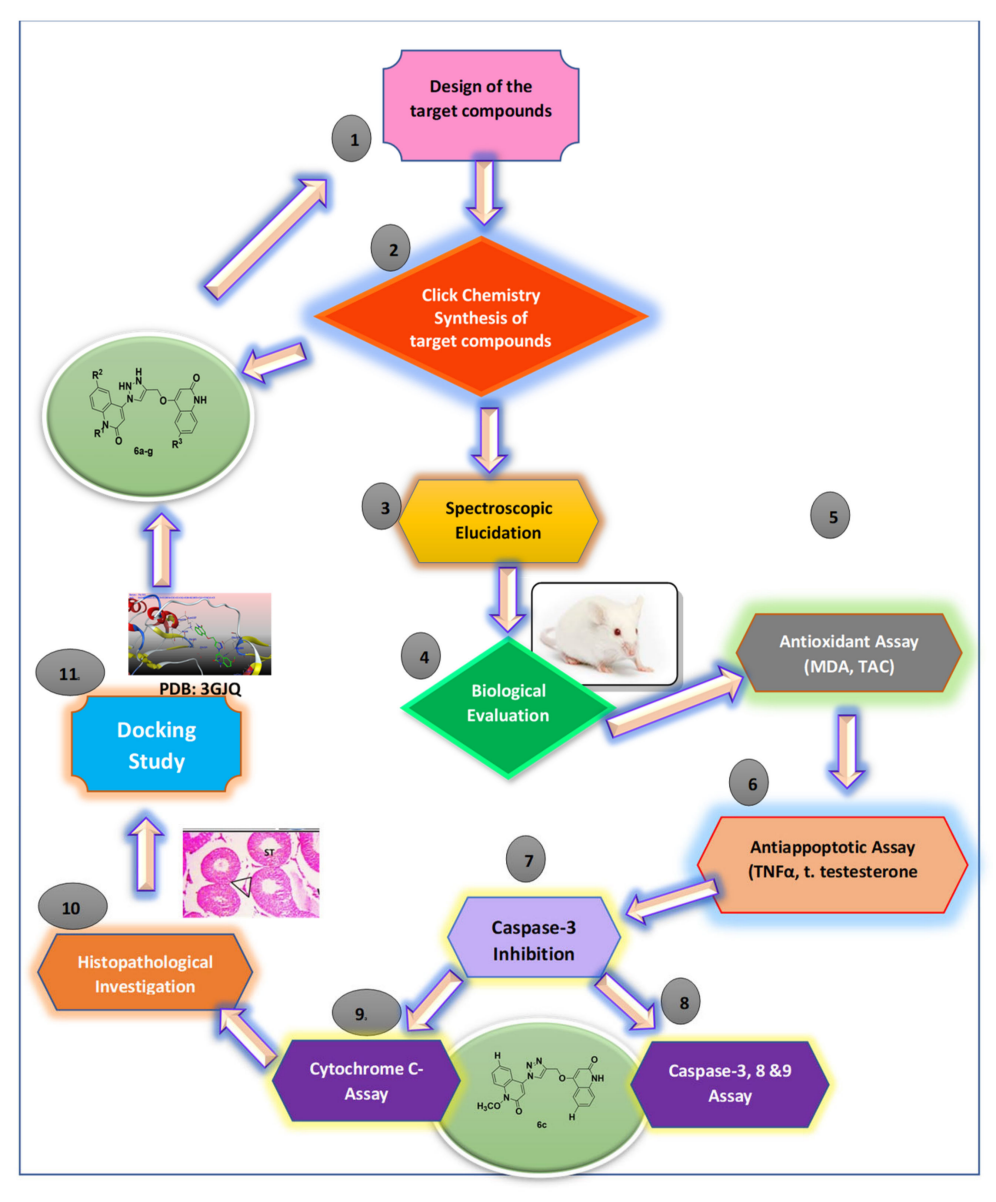
Schematic diagram illustrating all methodological steps.

In response to the previously mentioned aspects, we here reported our medicinal chemistry efforts, starting from the core scaffold of caspase inhibitor that led to gathering two types of scaffolds, triazole and quinolin-2-one, respectively, in one compact hybrid to design simpler and more efficient antiapoptotic compounds investigating the caspase-3 inhibition to minimize, or even prevent, testicular I/R injury as illustrated in a summarized schematic diagram (Figure 2). 

## 2. Results and Discussion

### 2.1. Chemistry 

To obtain 4-azidoquinolin-2(1*H*)-ones **4a**–**d**, 4-hydroxy-2-quinolinones **1a**–**d** were synthesized according to literature procedures [35,36], then treated with excess POCl_3_ in the presence of PCl_5_ at reflux for 1 h to give 2,4-dichloroquinolines **2a**–**c** and 4-chloro-2-quinolinone **2d** [37]. 

**Scheme 1:**
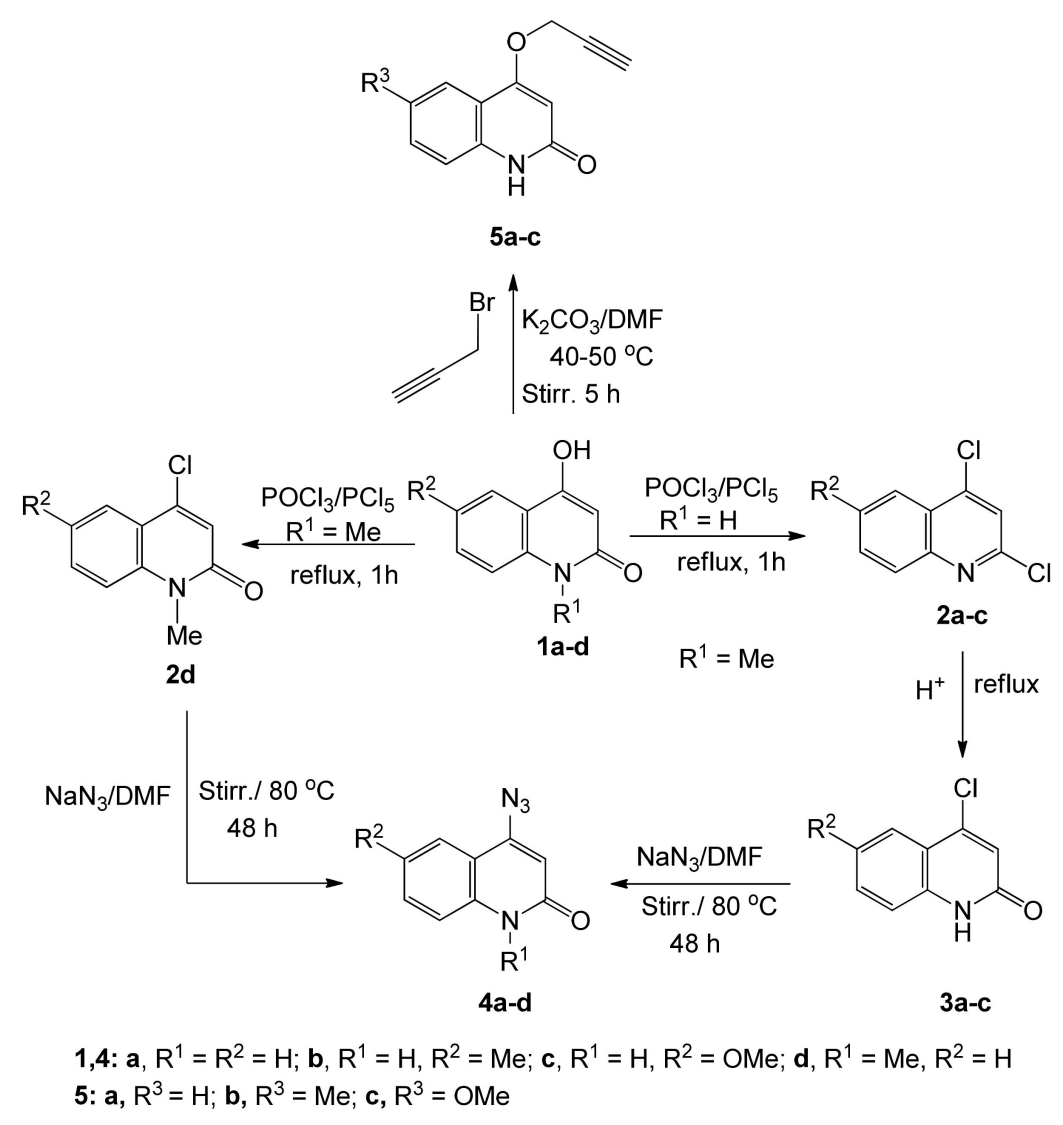
Preparation of starting materials **4a**–**d** and **5a**–**c**.

Compounds **2a**–**c** were heated with acetic acid/water (3:1) at reflux, to convert the Cl-group in position 2 into a carbonyl group to obtain 4-chloro-2-quinolinones **3a**–**c** [38]. Finally, either **2d** or **3a**–**c** was subjected to sodium azide in DMF with stirring at 70–80 °C, to give 4-azidoquinolin-2(1*H*)-ones **4a**–**d** according to literature procedures [38,39,40,41] (Scheme 1). The reaction of 6-substituted 4-hydroxy-quinolin-2-(1*H*)-ones **1a**–**d** in DMF and K_2_CO_3_ with propargyl bromide gave products **5a**–**c** as white precipitates (Scheme 1).

**Scheme 2:**
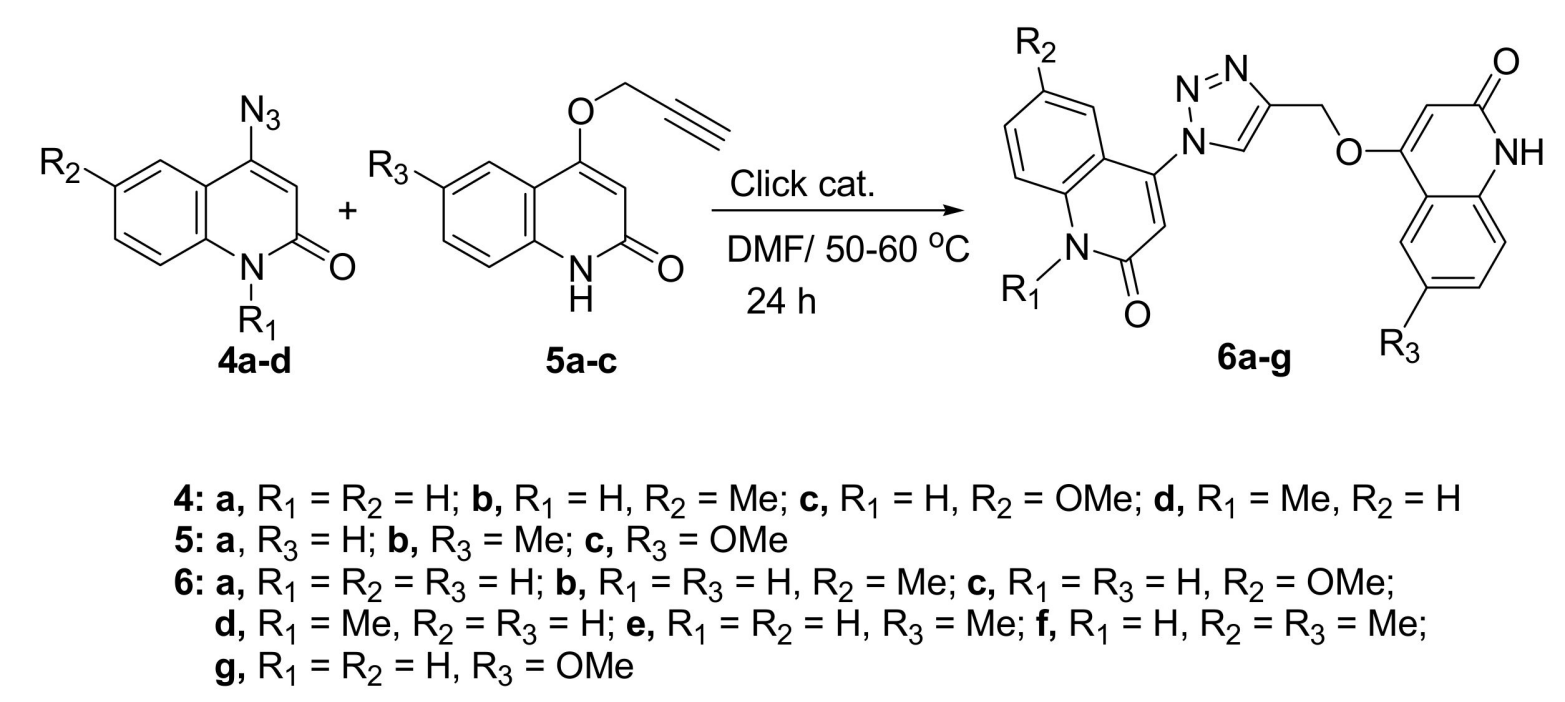
Click reactions between 4-azido-quinolin-2(1*H*)-ones **4a**–**d** and alkynes **5a**–**c**.

Aly et al. reported on the promising aspect of 3,3′-methylenebis(4-hydroxyquinolin-2(1*H*)-ones) as anti-Covid-19 [42]. In our present work, 1,2,3-triazoles were accessed by Huisgen type thermal 1,3-dipolar addition, which resulted in a mixture of 1,4- and 1,5-disubstituted triazoles [43]. The novel hybrids **6a**–**g** were synthesized through click chemistry, which is a powerful tool for quick, highly selective, and reliable access to a reaction product with good yields. Therefore, we carried out the [3+2] cycloadditions of 4-azidoquinolin-2(1*H*)-ones **4a**–**d** with 4-(prop-2-yn-1-yloxy)quinolin-2(1*H*)-ones **5a**–**c** to give the corresponding 4-((1-(2-oxo-1,2-dihydroquinolin-4-yl)-1*H*-1,2,3-triazol-4-yl)methoxy)quinolin-2(1*H*)-ones **6a**–**g** (Scheme 2). 

**Figure 3:**
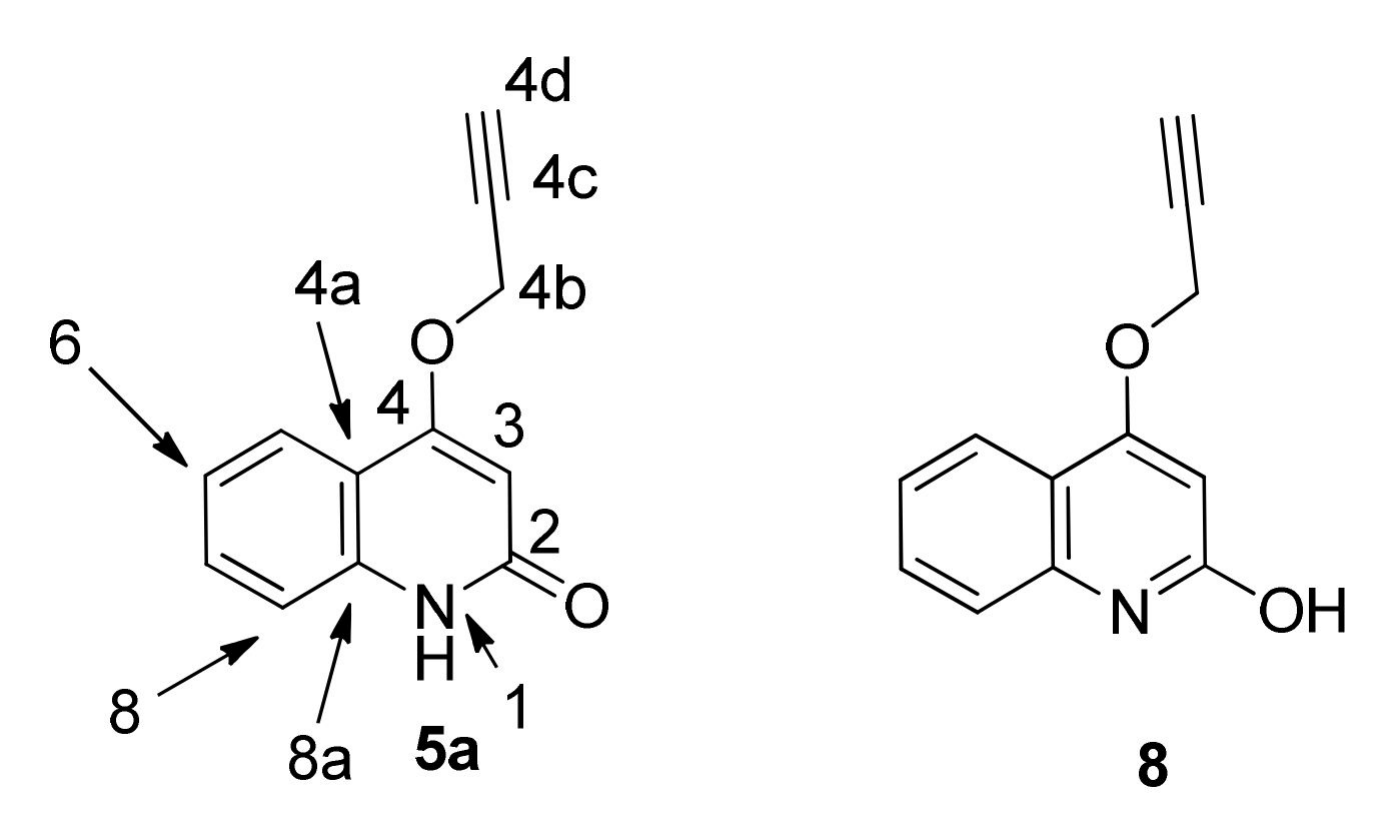
Structure of 4-(prop-2-yn-1-yloxy)quinolin-2(1*H*)-one: **5a** rather than **8**.

In the published synthesis of 4-(prop-2-yn-1-yloxy)quinolin-2(1*H*)-one (**5a**) [44], it is described as a quinolin-2-ol **8** (Figure 3). However, we observe an HSQC correlation between *N*-1 and H-1 not only for **5a** but throughout series **5** and **6** (Table 1). The HSQC sequence with standard settings (in this case, ^1^*J*_N-H_ = 90 Hz) is well-known to detect one-bond N-H couplings but not long-range couplings [33,34]; therefore, we assign the series **4**–**6** as quinolin-2(1*H*)-ones. Our chemical shifts for **5a** in CDCl_3_ do not match those reported for **8** [32]; the tautomer population may vary with different samples.

**Figure 4:**
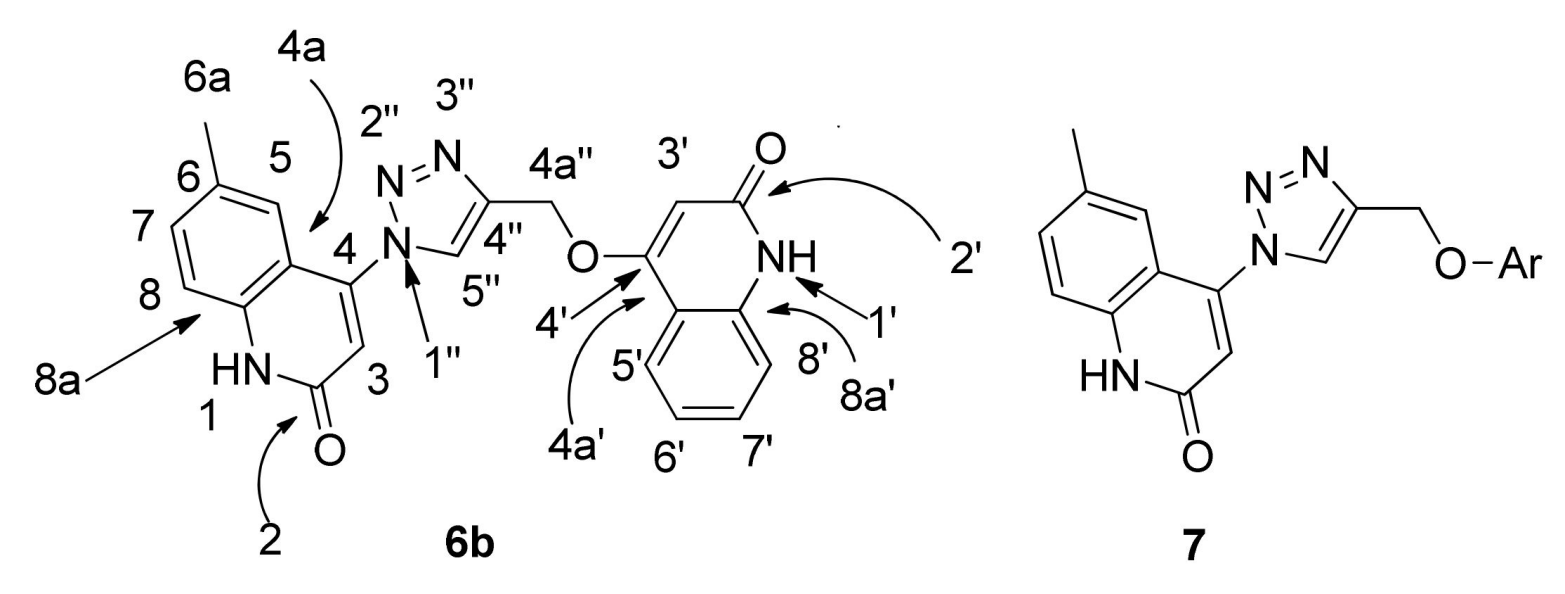
6-methyl-4-(4-(((2-oxo-1,2-dihydroquinolin-4-yl)oxy)methyl)-1*H*-1,2,3-triazol-1-yl)quinolin-2(1*H*)-one **6b** and **7** (Ar = *p*-formylphenyl).

The structures of compounds **6a**–**g** were confirmed by using different spectral data such as ^1^H-NMR, ^13^C-NMR, 2D-NMR, ^15^N-NMR, and mass spectrometry, as well as elemental analyses. For example, compound **6b** was assigned as 6-methyl-4-(4-(((2-oxo-1,2-dihydroquinolin-4-yl)oxy)methyl)-1*H*-1,2,3-triazol-1-yl)quinolin-2(1*H*)-one. It exhibited a molecular formula of C_22_H_17_N_5_O_3_, representing a product from one molecule of **4b** and one molecule of **5a** without elimination. This result was further confirmed by mass spectrometry with *m*/*z* = 399 and elemental analysis (Figure 4). In the ^1^H-NMR spectrum of compound **6b**, two quinolinone-NH protons appeared as broad signals at δ_H_ 12.22 and 11.44, one of them for the quinolinone carrying the triazole ring and the other one for the quinolinone initially carrying the propargyloxy group, respectively; these two protons were assigned exactly by analogy with compound **7** (Figure 4) [35] and from 2D-NMR (Table 2). Also, there were two doublets at δ_H_ 7.84 (*J* = 6.8 Hz; H-5′) and 7.30 (*J* = 7.4 Hz; H-8′), and five singlets at δ_H_ 8.98, 6.86, 6.22, 5.48 and 2.31, which were assigned as H-5″, H-3, H-3′, CH_2_ and CH_3_, respectively. 

In the ^13^C-NMR spectrum of **6b**, two signals appeared at δ_C_ 163.16 and 160.83, for two carbonyl groups assigned as C-2′ and C-2, respectively. Also, the signal at δ_C_ 61.81, which gives HSQC correlation with the proton at δ_H_ 5.48, was assigned as C-4a″ (methylene group). The signal at δ_C_ 20.52 ppm, which gives HSQC and HMBC correlation with the proton signal at δ_H_ 2.31, and H–H correlation with the proton at δ_H_ 7.27, was assigned as C-6a (methyl group). Other signals in the ^13^C-NMR of **6b** appeared at δ_C_ 161.75, 143.38, 126.73, 123.81, 117.81 and 97.93, and were assigned to C-4′, C-4″, C-5″, C-5, C-3 and C-3′, respectively (Table 2). Also, the triazole signals were distinctive in the proton spectrum at δ_H_ 5.48 (2H; H-4a″) and 8.98 (1H; H-5″); correlations with the proton signals led to the assignment of the carbons at δ_C_ 142.39 (C-4″), 126.73 (C-5″), and 61.81 (C-4a″). ^15^N-NMR showed three types of nitrogen. One appears at δ_N_ 248.0, which indicates an *sp*^2^ nitrogen; it was assigned as N-1″ and gives HMBC correlation with a proton at δ_H_ 8.98 (H-5″) but gives no HSQC correlation. The other two nitrogen atoms resonated at δ_N_ 151.6 and 144.0; their chemical shifts are characteristic of amide-type nitrogen atoms, and they give HSQC correlation with the attached protons at δ_H_ 12.29 and 11.43; therefore, these nitrogen atoms were assigned as N-1 and N-1′, respectively.

### 2.2. Evaluation of Biological Activity

**Figure 5:**
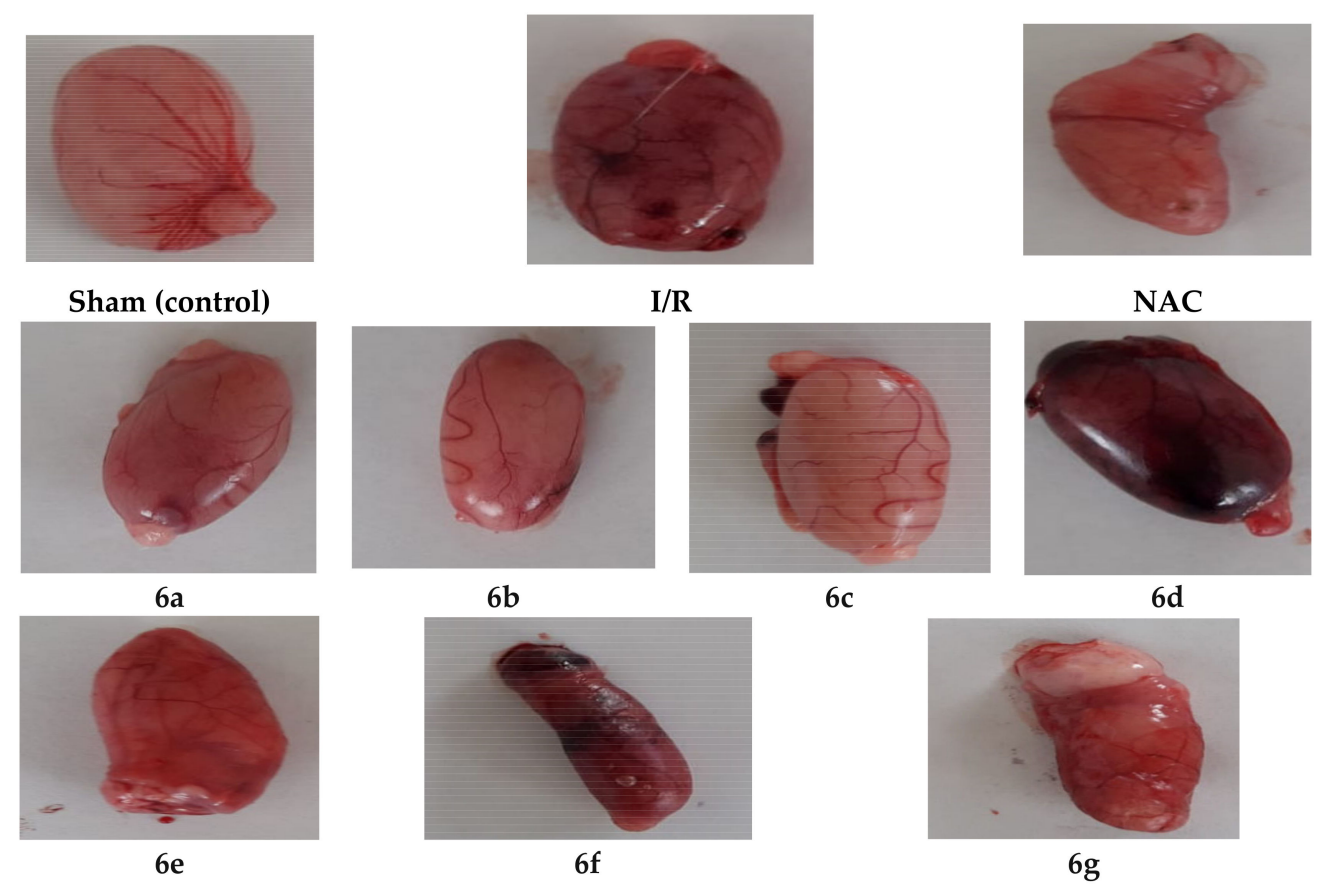
Photos of testicular specimens for groups of sham, I/R, reference (*N*-acetylcysteine, NAC) and treated with compounds **6a**–**g**.

This section describes the methods used in the investigation of the antiapoptotic effect of the novel compounds **6a**–**g** then supported by further histological and molecular docking studies. The antiapoptotic effect of **6a**–**g** was evaluated through evaluation of decreasing tissue damage induced by I/R in the testis of rats in a dose equivalent to 30 mg *N*-acetylcysteine (NAC) as an antiapoptotic reference [45,46]. The rats were subdivided into sham, I/R, NAC and treated with tested compounds groups, each group comprises of 6 rats (weighing 140–150 g). The targeted compounds **6a**–**g** were administrated intra-peritoneal (i.p.) an hour before ischemia, which lasted for (0.5 h) then reperfusion (1 h) followed by cutting the organ (Figure 5). Different biomarkers concentrations were measured in serum to detect testis injury due to ischemia/reperfusion (I/R). Some of these biomarkers are increased and others decreased indicating an apoptotic effect as shown in Table 3.

#### 2.2.1. In Vivo Assay of Antioxidant Biomarkers in Testis (MDA and TAC)

**Figure 6:**
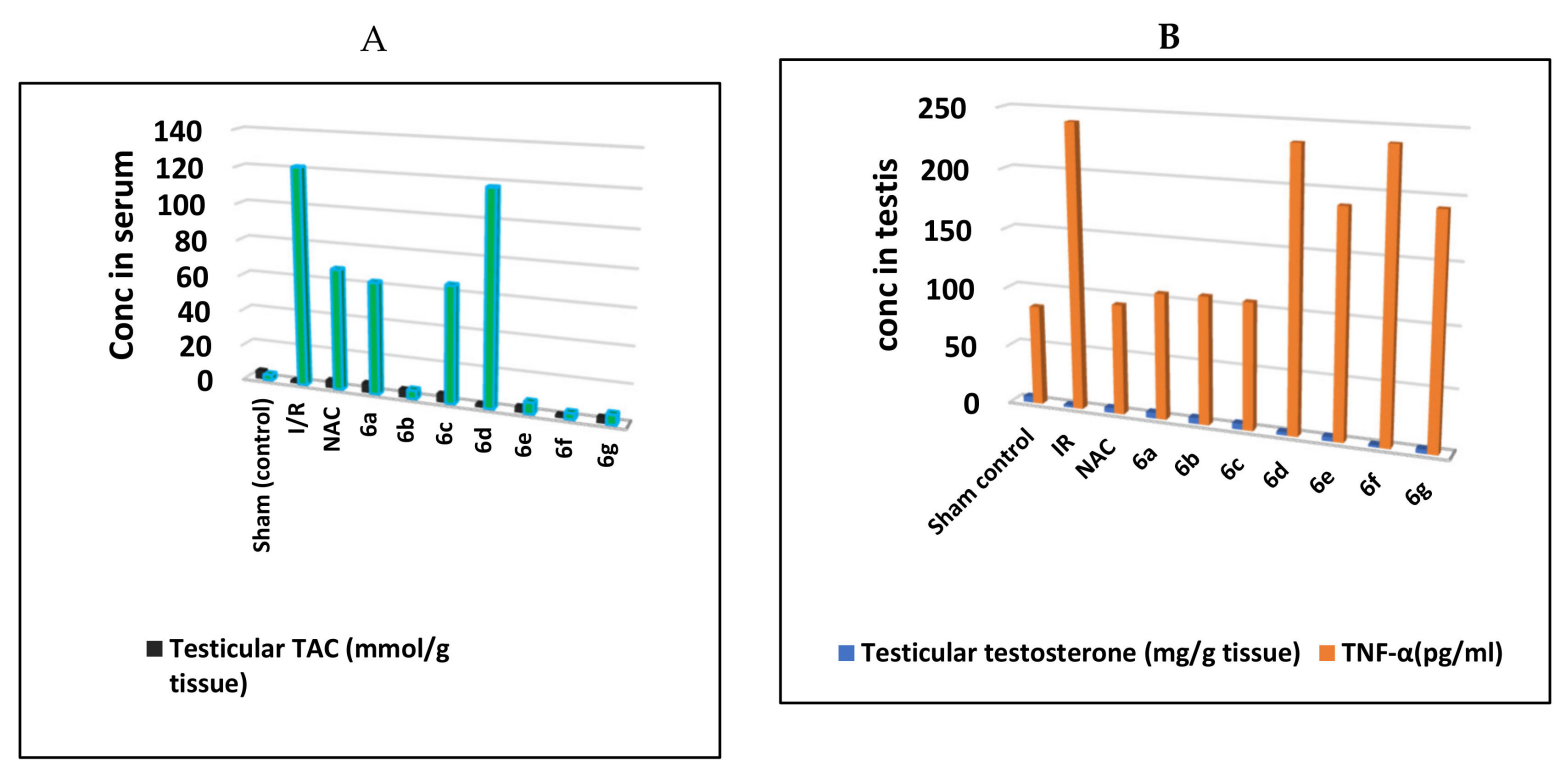
Concentrations of (**A**); testicular malondialdehyde (MDA) and testicular total antioxidant capacity (TAC), (**B**); testicular testosterone and TNFα in the testis of I/R rats treated with compounds **6a**–**g** and NAC.

During the long testicular torsion, oxidation damage affects the testis by the production of the reactive oxygen species (ROS) [51]. Excessive generation of the ROS interacts with lipids, proteins, and nucleic acids, which has an adverse effect on cell function and damage [52]. The testis has high cell metabolism such that excessive ROS production weakens antioxidant capacity [53]. Malondialdehyde (MDA) is the end product of lipid peroxidation and increased MDA level has an adverse effect on sperm fertility [54]. Furthermore, total antioxidant capacity (TAC) is the measure of the number of free radicals scavenged by a test solution, being used to evaluate the antioxidant capacity of biological samples [55]. The data collected in Table S3 (Supplementary Materials) have identified that there was a significant increase in testicular MDA with a significant decrease in testicular TAC in the I/R group as well as compounds **6d** and **6f** when compared to sham control and NAC groups. These findings indicate that both **6d** and **6f** are the worst antiapoptotic compounds compound amongst all the synthesized compounds. Meanwhile, **6a**–**c** are the most potent derivatives showing a significant decrease in testicular MDA (conc 62.38, 61.84 and 64.93 nmol/g tissue, respectively) along with a significant increase in testicular TAC (5.48, 4.62, 4.78 mmol/g tissue; respectively) relative to NAC (67.58 nmol/g tissue and 4.45 mmol/g tissue, respectively) when compared to I/R group. Compounds **6e** and **6g** were still showing significantly not improved testicular MDA and TAC (Figure 6A).

#### 2.2.2. In Vivo Assay of Apoptotic Biomarkers (Testicular Testosterone and TNFα)

It was reported that excessive apoptosis in the testis could damage the Leydig cells and apparent decline in serum testosterone levels [56]. Furthermore, excessive apoptosis of Leydig cells results in decreased testosterone. The assays of the apoptotic biomarkers (testicular testosterone and TNFα) were done in comparison with the sham and NAC treated group and the results were outlined in Table S4 (Supplementary Materials). Groups of I/R and both compounds **6d** and **6f** showed a significant decrease in testicular testosterone with a significant increase in TNF-α when compared to sham control and NAC groups (Figure 6B). Interestingly, groups of compounds **6a**–**c** showed a significant increase in testicular testosterone with a significant decrease in TNF-α in comparison to the IR group and relative to the reference NAC group. On the other hand, compounds **6e** and **6g** revealed remarkable testicular testosterone and TNF-α (Table S4 in Supplementary Materials).

#### 2.2.3. Assay of Caspase-3

##### Assay of Caspase-3 Inhibition in Serum

**Figure 7:**
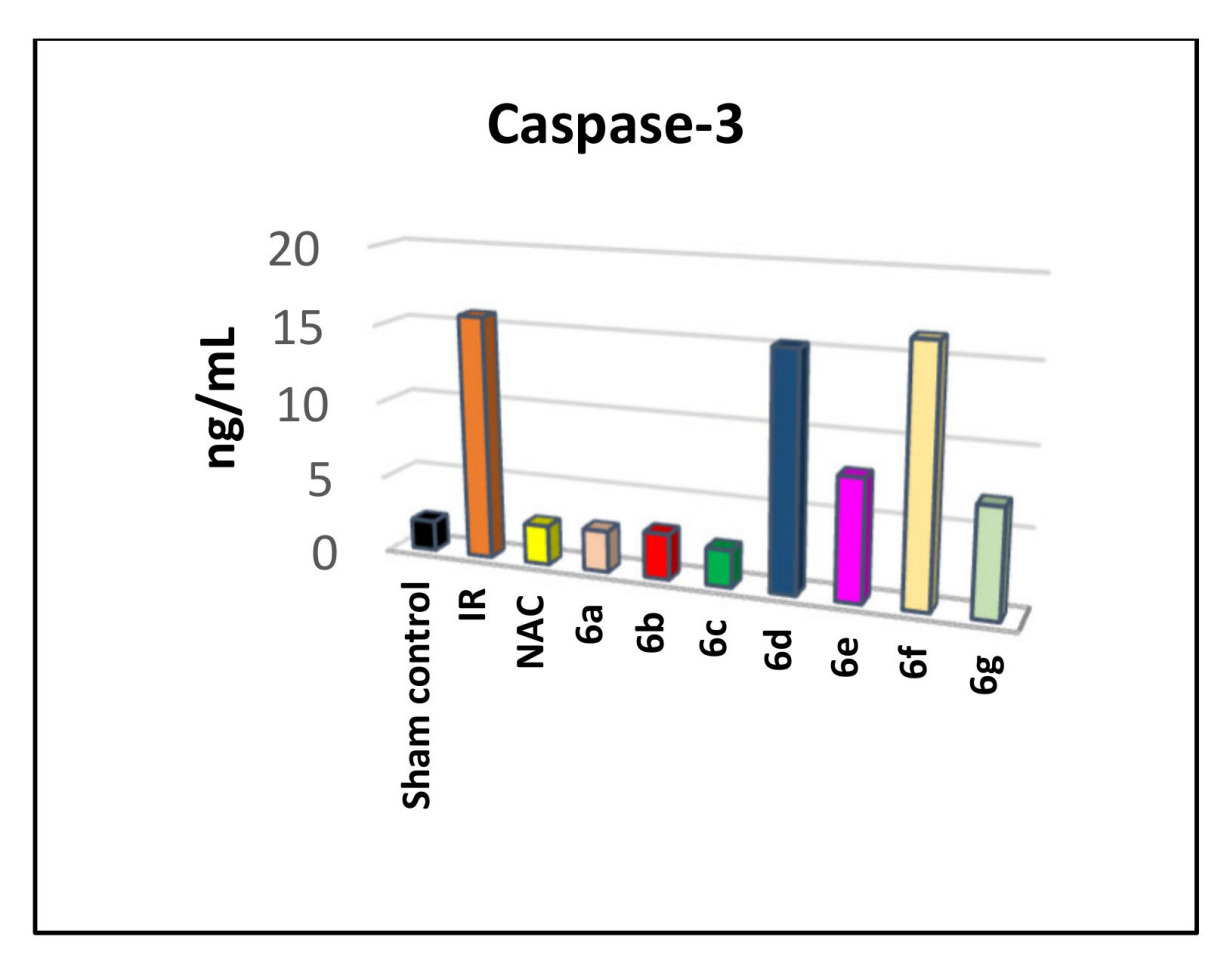
Caspase-3 level (ng/mL) in the serum of testicular I/R rats treated with compounds **6a**–**g** and NAC.

The above findings indicate a remarkable antiapoptotic activity of our target compounds that inhibition of caspase-3 might probably be the suggested target mechanism. Caspases can delay apoptosis, implicating a potential role in drug screening efforts [1]. Therefore, we studied the mechanism of the caspase-3 inhibition activity. Caspase-3 was assayed in the serum of rats treated with **6a**–**g** in comparison with sham and the NAC treated group, as shown in Table S5 (Supplementary Materials). Groups of I/R and both compounds **6d** and **6f** showed significant caspase-3 when compared to sham control and NAC groups (Figure 7). Moreover, groups of compounds **6a**–**c** showed a significant decrease in caspase-3 in comparison to the IR group and relative to the reference NAC group. On the other hand, compounds **6e** and **6g** displayed a remarkable testicular caspase-3 as in Table S5 (Supplementary Materials).

##### Caspases-3, 8 and 9 Selectivity Assay in MOLT-4 Cell Line

To outline compounds **6c** antiapoptotic mechanism whether it is through the inhibition of the intrinsic or the extrinsic pathway or both, their effect on caspase-8 and caspase-9 was also evaluated. Moreover, to investigate the caspase-3 inhibition as well as selectivity being the suggested mechanism, the most active caspase-3 inhibitor 1,2,3-triazole/biquinolinone hybrid **6c** was further studied for its effect on caspase-3, 8, and 9 and compared to the reference NAC.

**Figure 8:**
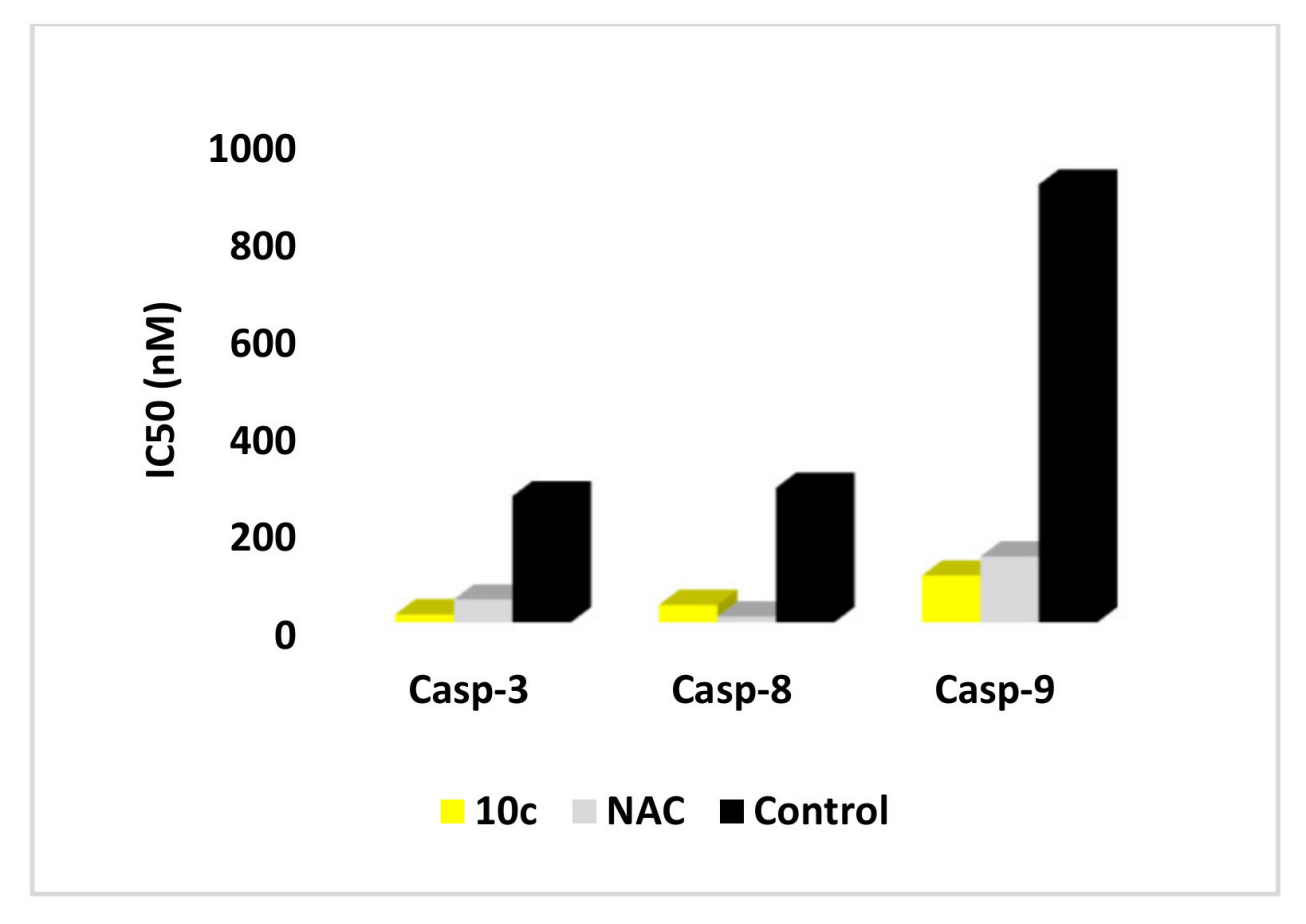
IC_50_ (nM) of compounds **6c** and NAC on the active caspases-3, 8, and 9.

The assay utilizes synthetic peptide substrate DEVD-AFC (AFC, 7-amino-4-trifluoromethyl coumarin). Cell-based caspase-3, and proapoptotic caspase-8 and 9 cleave the synthetic substrate to release free AFC, which can then be quantified by fluorometry. Compounds to be screened can directly be added to the reaction, and the level of inhibition of caspase-3 activity can be determined by comparison of the fluorescence intensity in samples with and without the testing inhibitors. Compound **6c** showed downregulation in the level of active caspase-3 with IC_50_ = 16.31 nM compared to NAC (IC_50_ = 46.83 nM) as shown in (Table S1, Supplementary Materials). Moreover, the effect of compound **6c** on caspases-8 and 9 was also evaluated revealing a decrease in the levels of caspases 8 and 9 with IC_50_ = 35.57 and 96.24 nM, respectively, relative to NAC and compared to the untreated control (Figure 8). The data indicate that **6c** is a putative inhibitor of both intrinsic and extrinsic caspase pathways with more selectivity and potency effect on caspase-3.

##### Assay of Cytochrome C in SR Human Cell Line

For more proof of caspase-3 inhibition by our synthesized compounds, cell-based cytochrome *C* was assayed. It was reported that cytochrome *C* concentration in the cell has a critical role in the inactivation of caspases and prohibiting the intrinsic apoptosis pathway [57]. Antiapoptotic proteins can inhibit apoptosis by blocking the release of cytochrome *C* while proapoptotic members function as activators of its release. When cytochrome *C* level is maintained in mitochondria*,* caspase-8 and 9 will not be activated leading to caspase-3 inactivation, which in turn ceased apoptosis and reserved the cell from the programmed death [58].

The 1,2,3-triazole/biquinolinone derivative **6c** was evaluated for cytochrome *C* against the SR human cell line and the results are listed in Table 4. Compound **6c** caused down-expression of cytochrome *C* level about 3.54 fold, comparable to NAC (4.13 fold), and lower than control. The displayed results could be a good guide for suggesting that antiapoptotic activity may be attributed to the attenuation of cytochrome *C* and hence inactivation of the intrinsic apoptotic pathway induced by the tested compounds.

#### 2.2.4. Histopathological Investigation

Throughout the previous work, results were consistent that motivated us to carry out a histopathological investigation of the tested compounds in the testis.

##### Spermatogenesis Scoring

**Figure 9:**
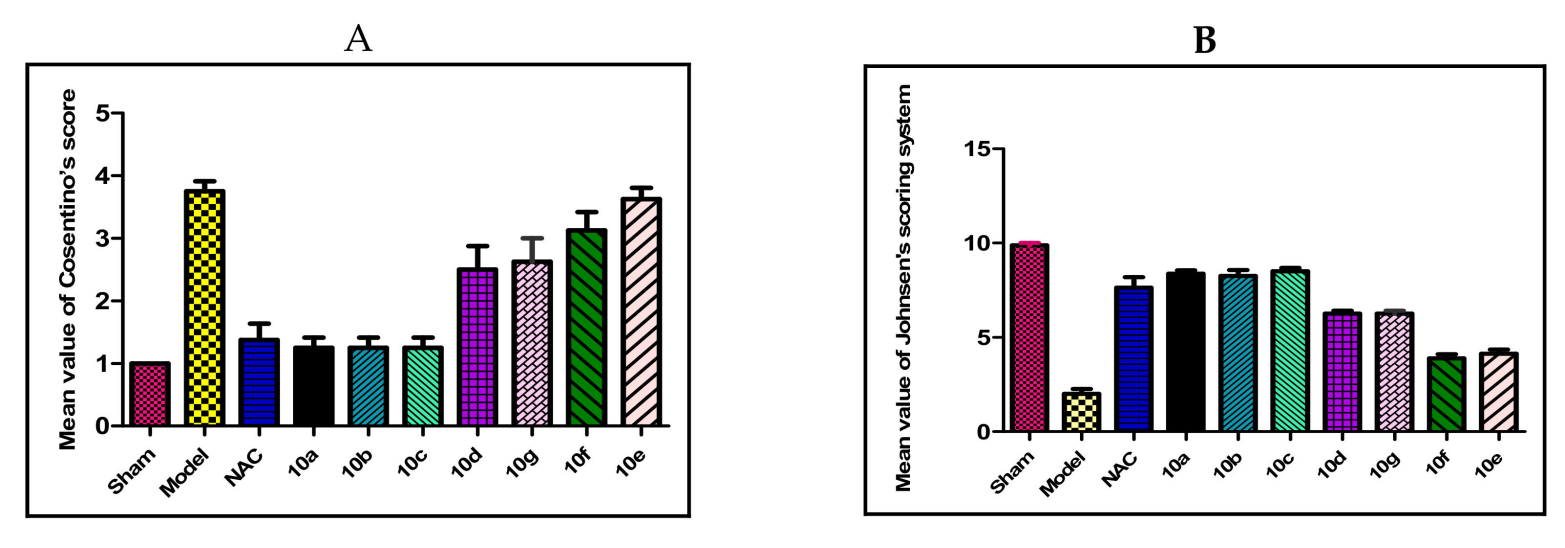
The histopathological findings of (**A**); Cosentino’s score, (**B**); Johnson’s scoring. Results were represented by the mean ± *SE* (*n* = 6).

The testicular I/R significantly made more histopathological changes (Figure 9A) of the tested compounds if compared to the control group. Also, it decreased spermatogenesis when compared to the control group by using Johnson’s scoring system (Figure 9B). Instead of that, groups of compounds **6a**–**c** showed no significant difference if compared to the reference (NAC) rats. On the other hand, compounds **6e** and **6g** showed weak significant differences if compared to the reference group. Whereas compounds **6d** and **6f** showed a highly significant histopathological difference compared to the reference group (all *p*-value < 0.005).

##### Histopathological Study

**Figure 10:**
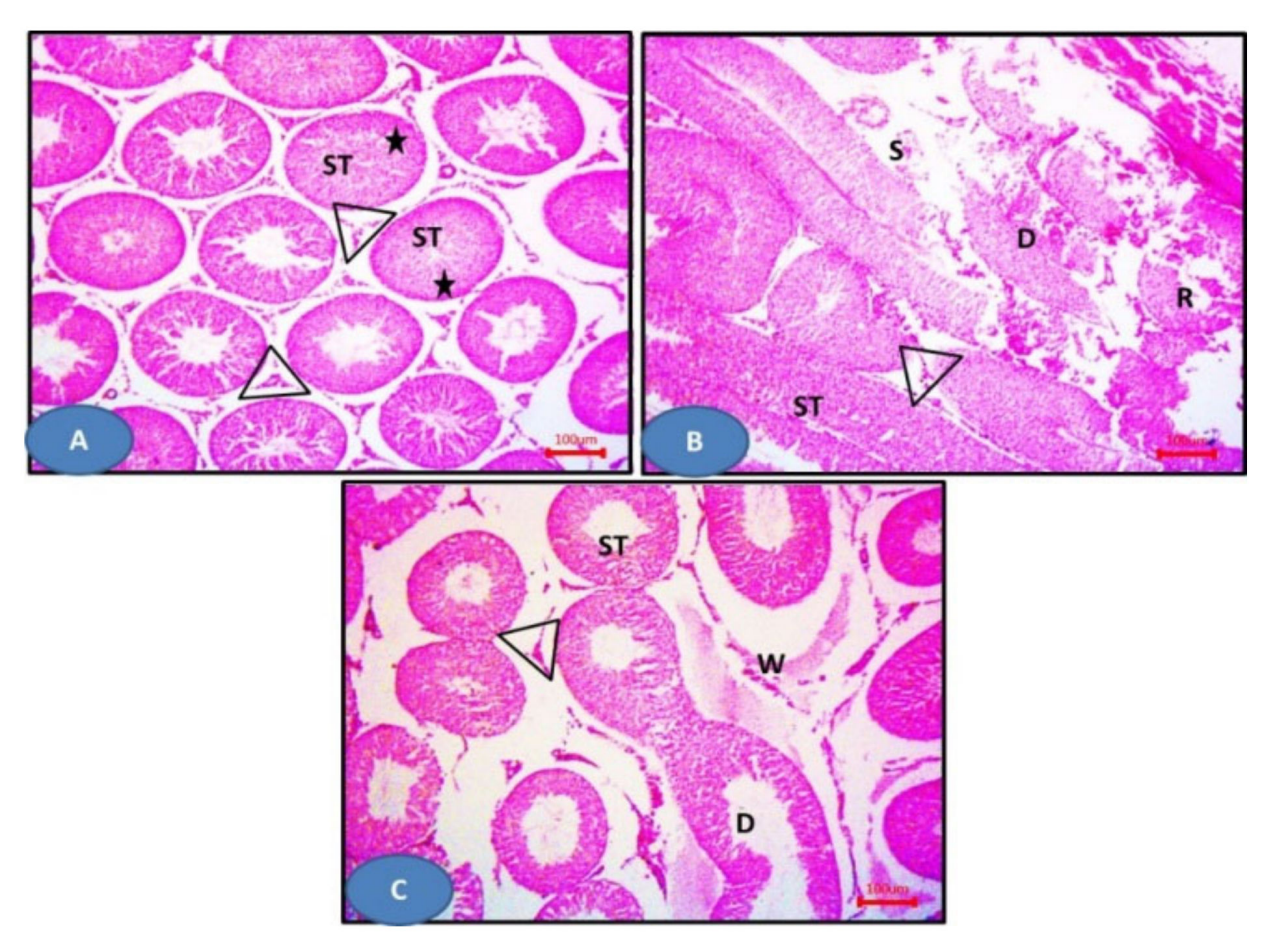
Photomicrographs of sections of the testis: (**A**) The sham group showing seminiferous tubules (ST) with germinal epithelium (stars) and Leydig cells (triangles). (**B**) Model group showing distorted seminiferous tubules lined (D) by disorganized epithelium. Notice the ruptured tubules (R) and the distorted Leydig cells (triangles). Sloughed cells (S) can be observed. (**C**) The NAC group showing more or less normal seminiferous tubules (ST) and Leydig cells (triangles), except some areas showing distorted tubules (ST). Notice the widening of inter-tubular spaces (W). H&E × 100.

**Figure 11:**
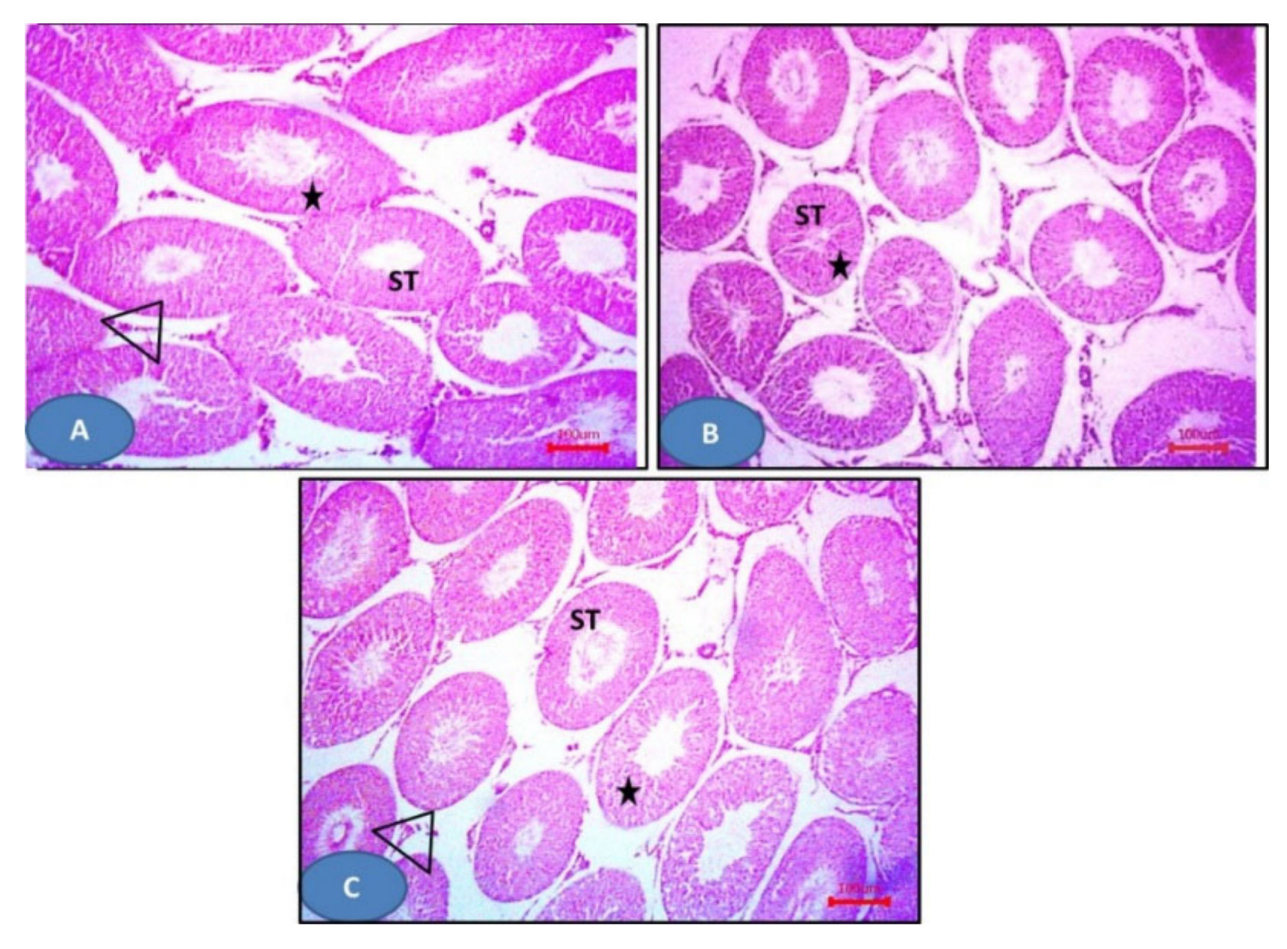
Photomicrographs of sections of the testis: (**A**–**C**) **6a**–**c** respectively showing more or less normal seminiferous tubules (ST) with germinal epithelium (stars) and Leydig cells (triangles). H&E × 100.

**Figure 12:**
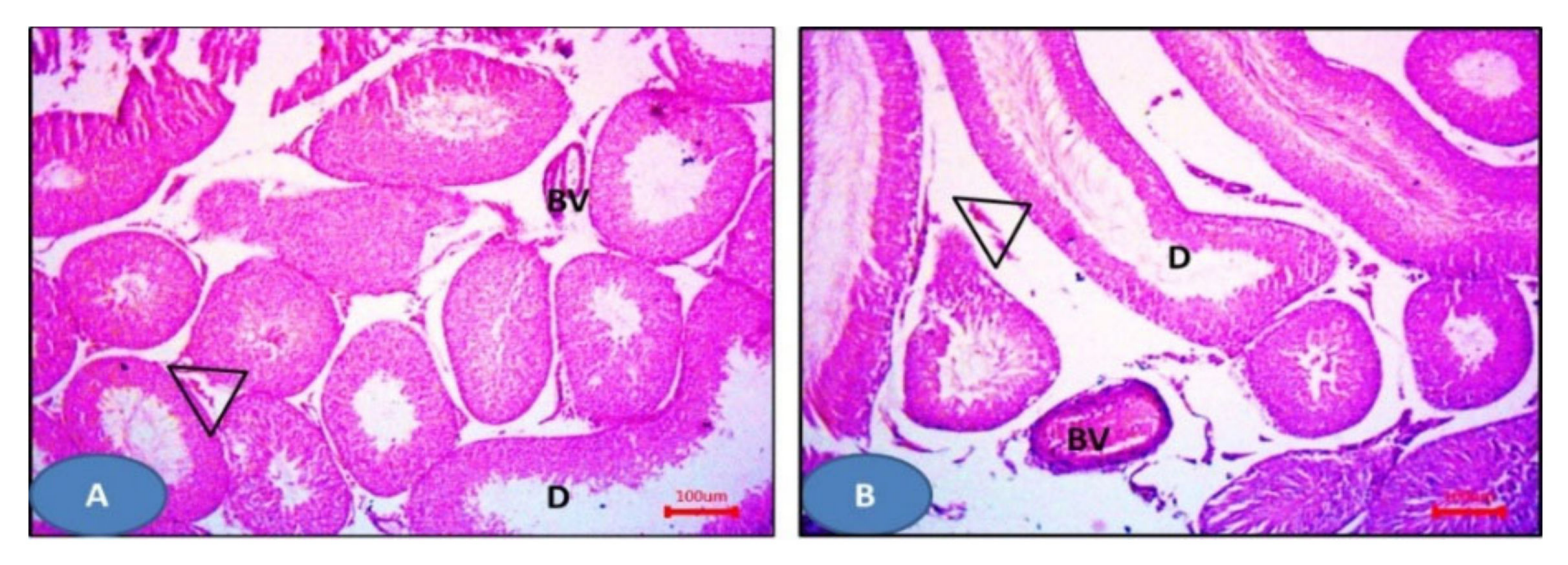
Photomicrographs of sections of the testis: (**A**,**B**) **6e** and **6g** respectively showing dilated seminiferous tubules (D) with distorted Leydig cells (triangles). Notice the dilated blood vessels. H&E × 100.

**Figure 13:**
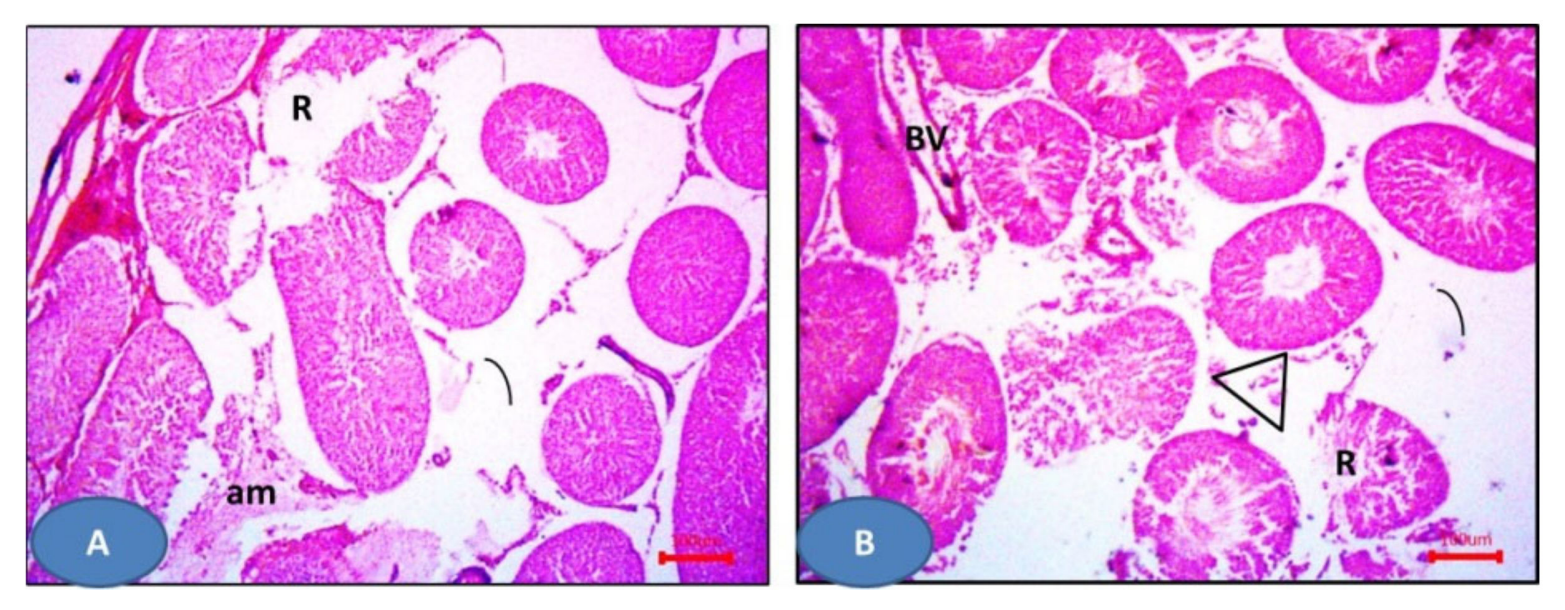
Photomicrographs of sections of the testis: (**A**,**B**) **6d** and **6f** respectively showed the ruptured seminiferous tubules (R) with distorted Leydig cells (triangles). Notice the dilated blood vessels (BV) and the amyloid substance (am). H&E × 100.

Hematoxylin and eosin slides of the sham group displayed the normal histological testicular morphology. The seminiferous tubules were seen lined with stratified testicular epithelium rested on basement membranes. The interstitial tissues contained clusters of Leydig cells (Figure 10A), while sections from the I/R group showed distorted seminiferous tubules lined by disorganized epithelium. Ruptured tubules and distorted Leydig cells and sloughed cells could be also observed (Figure 10B). NAC group showed more or less normal seminiferous tubules and Leydig cells except some areas displayed distorted tubules and widening of inter-tubular spaces (Figure 10C). Furthermore, drug **6a**–**c** showed more or less normal seminiferous tubules with germinal epithelium and Leydig cells (Figure 11). Additionally, **6e** and **6g** showed dilated seminiferous tubules. Distorted Leydig cells with dilated blood vessels (BV) were frequently seen among the sections (Figure 12). Compounds **6d** and **6f** exhibited ruptured seminiferous tubules with distorted Leydig cells. Dilated blood vessels and the presence of amyloid substance were also found (Figure 13).

*In summary*, testicular I/R induced many histopathological modalities in the form of germinal cell derangement and atrophy with necrosis, which is significantly different from the sham group by Cosentino’s score. Additionally, the spermatogenesis process was significantly decreased if compared to the sham control group using Johnson’s score, which confirmed the pathological damage in response to testicular I/R. These results match with the previously published reported results [59].

## 3. Computational Analysis

### 3.1. Docking Studies

Aiming to investigate the docking fitness scores of the bioactive conformations of the synthesized compounds and their specificity for caspase-3, docking of compounds **6a**–**g** was performed at the active site of the caspase-3 enzyme in complex with NAC to explore their binding modes as caspase-3 inhibitors using Molecular Operating Environment (MOE^®^) version 2014.09 (Montreal, QC, Canada). The crystal structure of caspase-3 protein taken from the protein data bank (PDB: 3GJQ) was used as a good template for many validation sets of structure-based pharmacophore modeling of caspase-3 inhibitors.

Molecular docking results were found to agree with the findings of biochemical and histopathological studies, for the highly active derivatives such as **6a**–**c** by caspase-3 inhibition activity.

### 3.2. Binding Modes of Tested Compounds with Caspase-3 Enzyme Active Site

As a first step, validation of the docking protocol settings was done through the re-docking of the extracted co-crystallized ligand Ac-IEPD-Cho from the 3D structure using the same protocol for the docked compounds (RMSD 0.38 Å). The used docking protocol closely reproduced the bound structure confirming the confidence in our docking study. The docked compounds were built using the builder tool in MOE then subjected to an energy minimization followed by a systematic conformational search using the default force field and settings, then the lowest energy conformer of each molecule was used for the docking experiment.

The docking study will be valid proof of the previously displayed mechanistic antiapoptotic study. Docking will be based on some aspects (i) binding with a caspase-3 catalytic binding site (ASP135 and LYS137), where the more binding, the more caspase inhibitory activity and the more antiapoptotic activity [60] along with the high-affinity binding score (s-score). (ii) The value of root-mean-square deviation refinement of atomic positions (RMSD_refine), whereas its values for the tested compounds are of range 1.79–3.68 Å, reflecting the fitting and stability of the compound conformation into the pocket. 

Recently, it is reported that an RMSD < 2.0 Å corresponds to good docking solutions. On the other hand, docking solutions with RMSD between 2.0 and 3.0 Å deviate from the position of the reference, but they keep the desired orientation. Finally, docking solutions with RMSD > 3.0 Å are completely wrong [61]. This could be explained by the fact that the highly active compounds **6b** showed low RMSD refined (root mean square deviation refinement) values near to the pose of the co-crystallized ligand, which ensures both the validity of the docking protocol and the good interactions of the ligands and the main residues at the active site domain.

**Figure 14:**
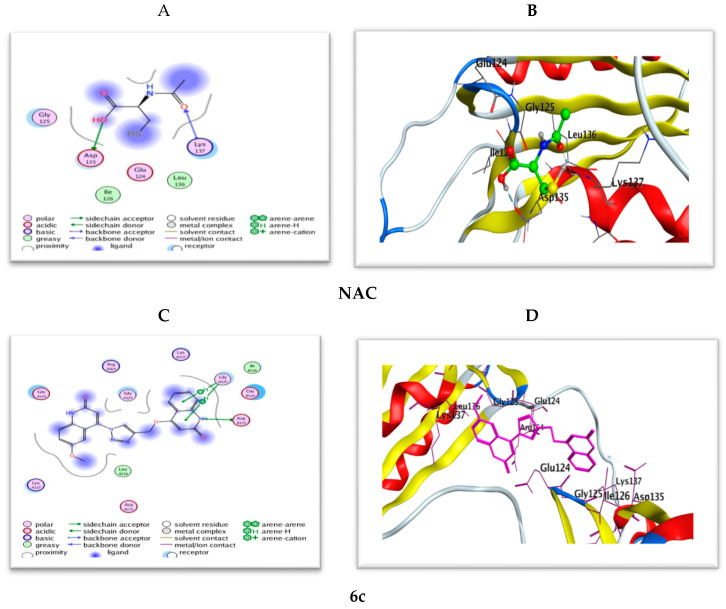
(**A**,**B**) 2D and 3D diagrams illustrate the binding modes of the reference **NAC** and (**C**,**D**) are for **6c** interacted with the active site of caspase-3 (PDB: 3GJQ).

Table S2 (Supplementary Materials) illustrates the binding free energies from the best favorable poses with the least S-score of the tested compounds at pKa = 7 and are shown in Figure 14. Most of the tested compounds have high binding affinity to the enzyme as their binding free energy (ΔG) values range from −0.6 to −9.4 Kcal/mol better than the reference NAC (ΔG = −0.8 to −6.5 Kcal/mol).

The docking study results of bis(quinolinone)/1,2,3-triazole derivatives **6a**–**g** showed better mode of interactions than the reference **NAC**. The 2D and 3D diagrams of the compounds showed crucial binding with LYS137 GLY125 and THR140 through quinoline NH and triazole C=N functionality. The next study, therefore, moves on to discuss the hypothesis that says linking with ASP135 with good binging energy and a low RMSD value give good caspase inhibition. Furthermore, stabilization of the reference NAC within the active site occurred through two strong hydrogen bond interactions with amino acid residues ASP135 and LYS137. Compound **6c** (Figure 14) exhibited the same interactions with the conserved amino acid ASP135 as NAC; however, most of the tested derivatives **6a,b,** and **d**–**g** possess interaction with LYS137 as the reference.

Most of the compounds showed interaction greater than NAC to interact with the same amino acids with an additional hydrophobic bond with GLY125, THR140, and PHE158. On the other hand, compounds **6f** (Figure S2) dismissed hydrogen binding interactions with the amino acid residue ASP135 and LYS137. Also, compound **6c** kept two hydrogen bond interactions with GLY125.

**Figure 15:**
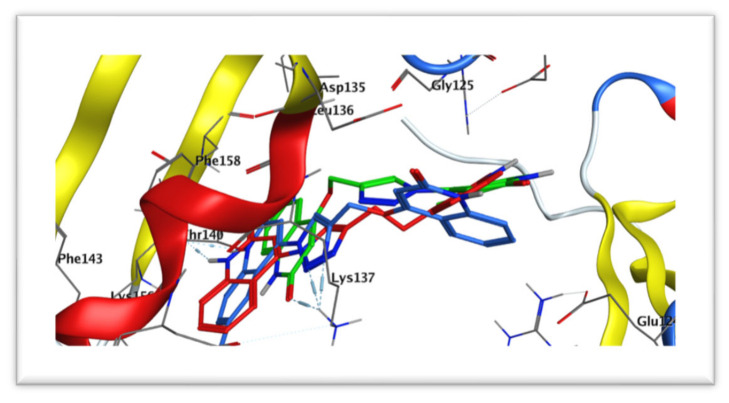
Docking of compounds **6a** (red) aligned with **6b** (blue) and **6c** (green) within the active site of caspase-3 (PDB: 3GJQ).

Furthermore, overall docking results revealed that the most potent new compounds **6a**–**c,** which are lacking substitutions at position R^1^and R^3^ of the two quinoline moieties showed binding modes within the active site indicating hydrogen bonding interaction with LYS137 (Figure 15). This finding is in agreement with what is explained in the SAR section that unsubstitution on the two 2-quinolone relieve steric hindrance, which may probably enhance binding with the caspase-3 active site.

### 3.3. Prediction of Physicochemical Properties, Pharmacokinetics, and Drug-Likeness Profile In Silico

In clinical trials, election of a new drug is considered so complicated due to inappropriate ADME (absorption, distribution, metabolism, and excretion) possessions in addition to the costs needed for developing a new drug. Hence, design and applied new drugs are considered to be complicated. Therefore, evaluating the pharmacokinetic properties of a new drug is a critical step in the process of drug development and can directly contribute to optimization efforts into recovered analogs [62]. Currently, The most promising compounds can be picked in silico ADMET screens, reducing the chance of degradation of drugs in late stages [63]. To achieve a desired in vivo response, it should be balanced between pharmacodynamics and pharmacokinetic properties. Also, information about regimen and drug dose is given by the prediction of brain penetration, volume of distribution, oral bioavailability, and clearance. Many parameters such as drug solubility S, partition coefficients, polar surface PSA, cell permeability, human intestinal absorption HIA, and drug-likeness score have been studied during virtual screening methods. An available oral drug elected in agreement with Lipinski’s rule if the molecular weight is less than 500, Log P is not higher than five, the number of hydrogen bond acceptors is less than 10 and the number of donor hydrogen bond donors is less than five [64]. The number of rotatable bonds reflects molecular flexibility that plays an important role in oral bioavailability and means less orally active in a flexible molecule. The number of hydrogen bonding groups has also been suggested as a consideration to substitute for the polar surface area (PSA) and also to measure the percentage absorption (%ABS) as it is inversely proportional to tPSA

%ABS = 109 − 0.345 tPSA(1)

The higher oral bioavailability exhibited by compounds with tPSA of fewer than 140 A^2^ and 10 or fewer rotatable bonds.

Herein, we used Pre-ADMET (https://preadmet.bmdrc.kr/) [65], Molinspiration (https://www.molinspiration.com/) [66], Molsoft (https://www.molsoft.com/about.html) [67], and SwissADME (http://www.swissadme.ch/) software [62] for predicting the pharmacokinetic parameters of the reported compounds. The results are shown in Table 5, all the target compounds **6a**–**g** obey the Lipinski’s rule with Log P values from 2.32 to 2.73 (<5), an MW range from 385.38 to 415.40 (<500), HBD from 1 to 2 (≤5) and HBA from 5 to 6 (<10). They would theoretically show a strong oral absorption and this property cannot be attributed to variations in their bioactivity. Furthermore, the topological PSA values of the compounds ring are between 94.80 and 114.89 A^2^ (<140 A^2^) and the corresponding percentage oral absorption was between 72.55 and 69.36%, exhibit strong permeability, absorption and transport across a biological membrane. Furthermore, the drug-likeness model score and solubility for compounds was confirmed by Molsoft software (Table 6). Aqueous solubility can change the absorption and distribution characteristics. The more positive the drug-likeness model scores, the more likely it is to be a drug molecule, and these compounds have fulfilled their solution ability specifications at LogS values between −3.30 and −3.78 (above Log S = −4). Positive model-scores (0.45, 0.36 and 0.31 respectively) were anticipated for compounds **6b, 6c** and **6f** while that for other compounds was negative (−0.18 to −0.23). 

Additionally, the following pharmacokinetic parameters were experimented with in silico using Pre-ADMET software: blood–brain barrier partition coefficient (BBB), cytochrome inhibition of cytochrome P4502D6 (CYP2D6), Caco2, coefficient (human colon adenocarcinoma), MDCK (Madin–Darby canine kidney cells) permeability coefficient, human intestinal absorption (HIA) and human plasma–protein binding (PPB). The results of the ADME parameters are shown in Table 7. The findings from compounds with moderate CNS absorption ranges between 0.039 and 0.075 (≤0.1); investigated compounds exhibited medium to low cell permeability in Caco-2, MDCK models range from 21.79 and 24.63, 4.39 to 1.02 nm/s, respectively. This is aligned with non-inhibitors of the CYP2D6 enzyme and thus may pretend to have no interactions with CYP2D6 inhibitors and/or inducers. Furthermore, all the compounds **6a**–**g** showed high human intestinal absorption values of 95.58 to 100%, indicating very well-absorbed compounds especially for **6a**, which exhibited 100% intestinal absorption. The examined compounds were found to be highly-bound to human plasma proteins from 89.41 to 97.71% except for **6g** that was low bounded to plasma protein 84.71%. 

### 3.4. Structure–Activity Relationship

Based on the previous results, we can deduce that when the two quinoline moieties are unsubstituted at position R^1^ and R^3^ as in compounds **6a**–**c**, the antiapoptotic activity revealed the highest potency. Meanwhile, upon substitution of R^3^ with an electron-donating group such as methyl (compound **6e**) or -OCH_3_ group (compound **6g**), the antiapoptotic activity was particularly diminished to become abolished upon substitution either at R^1^ only (compound **6d**) or at R^2^ and R^3^ for compound **6f**. These biochemical findings can be explained as a substitution at both two quinoline-2-one rings, which would hinder interaction with the target protein that may be attributed to the resulting steric hindrance. Because the target compounds **6a**–**g** revealed interesting downregulation of caspase-3 in testis, this work has undergone further histopathological mechanistic investigations to prove exactly the explained hypothesis. The in silico study revealed that all the target compounds **6a**–**g** obey the Lipinski’s rule in addition to the drug-likeness model score and solubility for compounds was confirmed by Molsoft software. The ADME predictions displayed moderate to good parameters for **6a**–**g**. These results confirm the successful design of the target compounds.

## 4. Material and Methods

### 4.1. General Information

All reagents were used as purchased from Merck (St. Louis, MO, USA). The progress of all reactions was monitored with thin-layer chromatography (TLC) on Merck alumina-backed TLC plates and visualized under UV light. Spectra were measured in DMSO-*d*_6_ on a Bruker AV-400 spectrometer (400 MHz for ^1^H, 100 MHz for ^13^C, and 40.54 MHz for ^15^N, in the Chemistry Department, Florida Institute of Technology, 150 W University Blvd, Melbourne, FL 32901, USA. Chemical shifts are expressed in δ (ppm) versus internal tetramethylsilane (TMS) = 0 ppm for ^1^H and ^13^C, and external liquid ammonia = 0 ppm for ^15^N. Coupling constants are stated in Hz. Correlations were established using ^1^H-^1^H COSY, and ^1^H-^13^C and ^1^H-^15^N HSQC and HMBC experiments. All ^15^N signals were observed indirectly, via HSQC or HMBC experiments. Chemical shifts (δ) are reported in parts per million (ppm) relative to tetramethylsilane (TMS) as internal standard, and the coupling constants (*J*) are reported in Hertz (Hz). Splitting patterns are denoted as follows: singlet (s), doublet (d), multiplet (m), triplet (t), quartet (q), doublet of doublets (dd), doublet of triplets (dt), triplet of doublets (td), and doublet of quartet (dq). Melting points (mp) were determined with a Stuart melting point instrument in the Chemistry Department, Florida Institute of Technology, 150 W University Blvd, Melbourne, FL, USA, and are expressed in °C as well as Mass spectra were recorded on a Finnigan Fab 70 eV at Al-Azhar University, Egypt. Elemental analyses were carried out on a Perkin Elmer device at the Microanalytical Institute of Organic Chemistry, Karlsruhe Institute of Technology, Karlsruhe, Germany.

### 4.2. Starting Materials

All 4-azidoquinolin-2(1*H*)-ones **4a**–**d** were prepared according to the literature as 4-azidoquinolin-2(1*H*)-one (**4a**) [68,69], 4-azido-6-methyl-quinoline-2(1*H*)-one (**4b**) [47], 4-azido-6-methoxy-quinoline-2(1*H*)-one (**4c**) [47] and 4-azido-1-methyl-quinoline-2(1*H*)-one (**4d**) [69], respectively.

### 4.3. General Procedure for the Formation of Compounds ***5a**–**c***

To a solution of 6-substituted 4-hydroxy-quinolin-2-(1*H*)-one (4 mmol) in DMF (30 mL) and K_2_CO_3_ (1.104 g, 8 mmol) was added propargyl bromide (0.944 g, 8 mmol) dropwise at 30–40 °C with stirring for 5 h and the reaction mixture was mentioned by TLC. Then the reaction mixture was poured into 200 g ice to give a white precipitate, which was filtered off and recrystallized from ethanol to give products **5a**–**c**.

4-(Prop-2-yn-1-yloxy)quinolin-2(1*H*)-one (**5a**). White solid, yield: 112 mg, 40%; mp 210–212 °C; ^1^H-NMR (DMSO-*d*_6_) δ_H_ 1.44 (s, 1H; H-1), 7.76 (d, *J* = 8.0, 1H; H-5), 7.53 (dd, *J* = 7.7, 7.7, 1H; H-7), 7.30 (d, *J =* 8.2, 1H; H-8), 7.18 (dd, *J* = 7.9, 7.3, 1H; H-6), 5.97 (s, 1H; H-3), 5.01 (d, *J* = 2.2, 2H; H-4c), 3.75 (t, *J* = 2.0, 1H; H-4e). ^1^H-NMR (CDCl_3_) δ_H_ 11.95 (b, 1H; H-1), 7.95 (d, *J* = 8.1, 1H; H-5), 7.55 (ddd, *J* = 7.7, 7.7, 1.2, 1H; H-7), 7.41 (d, *J =* 8.2, 1H; H-8), 7.24 (dd, *J* = 7.2, 7.2, 1H; H-6), 6.17 (s, 1H; H-3), 4.88 (d, *J* = 2.3, 2H; H-4c), 2.64 (t, *J* = 2.3, 1H; H-4e). ^13^C-NMR (DMSO-*d*_6_): δ_C_ 162.91 (C-2), 160.91 (C-4), 138.64 (C-8a), 131.04 (C-7), 122.11 (C-5), 121.44 (C-6), 115.22 (C-8), 114.29 (C-4a), 98.13 (C-3), 79.32 (C-4e), 77.89 (C-4d), 56.34 (C-4c). ^13^C-NMR (CDCl_3_): δ_C_ 163.11 (C-4), 138.10 (C-8a), 131.54 (C-7), 122.94 (C-5), 122.64 (C-6), 116.11 (C-8), 115.47 (C-4a), 97.17 (C-3), 77.17 (C-4e), 76.45 (C-4d), 56.55 (C-4c). ^15^N-NMR (DMSO-*d*_6_): δ_N_ 144.1 (N-1). ^15^N-NMR: (CDCl_3_): δ_N_ 145.3 (N-1). *Anal. Calcd. for* C_12_H_9_NO_2_: C, 72.35; H, 4.55; N, 7.03. Found: C, 72.44; H, 4.39; N, 6.99.

6-Methyl-4-(prop-2-yn-1-yloxy)quinolin-2(1*H*)-one (**5b**). White solid, yield: 176 mg, 60%; mp 214–216 °C; ^1^H-NMR (DMSO-*d*_6_): δ_H_ 11.37 (b, 1H; NH-1), 7.55 (bs, 1H; H-5), 7.35 (bd, *J* = 8.1, 1H; H-7), 7.20 (d, *J* = 8.1, 1H; H-8), 5.94 (s, 1H; H-3), 4.99 (bd, *J* = 1.2, 2H; H-4b), 3.75 (bt, 1H; H-4d), 2.35 (s, 3H; CH_3_). ^13^C-NMR (DMSO-*d*_6_): δ_C_ 162.79 (C-2), 160.77 (C-4), 136.65 (C-8a), 132.21 (C-7), 130.47 (C-6), 121.55 (C-5), 115.18 (C-8), 114.19 (C-4a), 98.09 (C-3), 79.26 (C-4d), 77.93 (C-4c), 56.27 (-O-CH_2_), 20.45 (CH_3_). ^15^N-NMR (DMSO-*d*_6_): δ_N_ 144.1 (NH). *Anal. Calcd. for* C_13_H_11_NO_2_: C, 73.23; H, 5.20; N, 6.57. Found: C, 73.33; H, 5.08; N, 6.68.

6-Methoxy-4-(prop-2-yn-1-yloxy)quinolin-2(1*H*)-one (**5c**). White solid, yield: 155 mg, 50%; mp 220–222 °C; ^1^H-NMR (DMSO-*d*_6_): δ_H_ 11.36 (b, 1H; NH-1), 7.50 (bs, 1H; H-5), 7.35 (bd, *J* = 7.8, 1H; H-7), 7.23 (d, *J* = 7.8, 1H; H-8), 5.91 (s, 1H; H-3), 4.99 (bd, *J* = 1.3, 2H; H-4b), 3.75 (bt, 1H; H-4d), 3.73 (s, 3H; -OCH_3_). ^13^C-NMR (DMSO-*d*_6_): δ_C_ 162.59 (C-2), 160.76 (C-4), 151.35 (C-6), 135.50 (C-8a), 132.21 (C-7), 122.05 (C-5), 115.48 (C-8), 114.13 (C-4a), 97.99 (C-3), 79.22 (C-4d), 77.93 (C-4c), 55.98 (-O-CH_2_), 55.13 (-OCH_3_). ^15^N-NMR (DMSO-*d*_6_): δ_N_ 144.1 (NH). *Anal. Calcd for* C_13_H_11_NO_3_: C, 68.11; H, 4.84; N, 6.11. Found: C, 68.29; H, 4.77; N, 5.98.

### 4.4. General Procedure for the Formation of Compounds ***6a**–**g***

In a round bottom flask, mixing (1 mmol) of terminal alkynes **5a**–**c** in 20 mL dimethylformamide (DMF), CuI (10 mol%) was stirred for 10 min at room temperature. Then, 4-azido compounds **4a**–**d** (1.0 mmol) were added to the mixture. The reaction mixture was allowed to stir at 50–60 °C for 24 h. The reaction was monitored with TLC. After completion, the reaction mixture was poured on 200 g ice and extracted by filtration.

4-((1-(2-Oxo-1,2-dihydroquinolin-4-yl)-1*H*-1,2,3-triazol-4-yl)methoxy)quinolin-2(1*H*)-one (**6a**). Yellowish green solid, yield: 328 mg, 85%; mp 300–302 °C; ^1^H-NMR (DMSO-*d*_6_): δ_H_ 12.29 (bs, 1H; NH-1), 11.43 (bs, 1H; NH-1′), 9.00 (s, 1H; H-5″), 7.84 (d, *J* = 7.6, 1H; H-5′), 7.67 (dd, *J* = 7.1, 7.1, 1H; H-7), 7.52-7.48 (m, 3H; H-5,7′,8), 7.29-7.27 (m, 2H; H-6,8′), 7.16 (dd, *J* = 7.0, 7.0, 1H; H-6′), 6.91 (s, 1H; H-3), 6.21 (s, 1H; H-3′), 5.47 (s, 2H; -O-CH_2_). ^13^C-NMR (DMSO-*d*_6_): δ_C_ 163.12 (C-2′), 161.75 (C-4′), 160.96 (C-2), 143.59 (C-8a), 142.41 (C-4″), 139.45 (C-4), 138.67 (C-8a′), 131.89 (C-7), 131.02 (C-7′), 126.74 (C-5″), 124.02 (C-5), 122.60, 122.50 (C-5′,6), 121.32 (C-6′), 117.82 (C-3), 115.93 (C-4a/4a′), 115.15 (C-8), 114.46 (C-4a′/4a), 114.41 (C-8′), 97.93 (C-3′), 61.81 (-O-CH_2_). EI-MS (*m*/*z*,%): 385 (M^+^, 26). *Anal. Calcd for* C_21_H_15_N_5_O_3_: C, 65.45; H, 3.92; N, 18.17. Found: C, 65.60; H, 3.88; N, 18.29.

6-Methyl-4-(4-(((2-oxo-1,2-dihydroquinolin-4-yl)oxy)methyl)-1*H*-1,2,3-triazol-1-yl)quinolin-2(1*H*)-one (**6b**). Yellowish green solid, yield: 360 mg, 90%; mp 298 °C; NMR: see Table 2. EI-MS (*m*/*z*,%): 399 (M^+^, 10). *Anal. Calcd for* C_22_H_17_N_5_O_3_: C, 66.16; H, 4.29; N, 17.53. Found: C, 66.09; H, 4.33; N, 17.69.

6-Methoxy-4-(4-(((2-oxo-1,2-dihydroquinolin-4-yl)oxy)methyl)-1*H*-1,2,3-triazol-1-yl)-quinolin-2(1*H*)-one (**6c**). White solid, yield: 270 mg, 65%; mp 295–297 °C; ^1^H-NMR (DMSO-*d*_6_): δ_H_ 12.21 (bs, 1H; NH-1), 11.44 (bs, 1H; H-1′), 9.04 (s, 1H; H-5″), 7.84 (d, *J* = 8.0, 1H; H-5′), 7.53 (dd, *J* = 7.5, 7.5, 1H; H-7′), 7.45 (d, *J* = 9.0, 1H; H-8), 7.36 (dd, *J* = 9.0, 2.6, 1H; H-7), 7.30 (d, *J* = 8.1, 1H; H-8′), 7.16 (dd, *J* = 7.4, 7.4, 1H; H-6′), 6.96 (d, *J* = 2.6, 1H; H-5), 6.91 (s, 1H; H-3), 6.21 (s, 1H; H-3′), 5.49 (s, 2H; H-4a″), 3.71 (s, 3H; H-6b). ^13^C-NMR (DMSO-*d*_6_): solubility was low. Carbon signals were observed indirectly, using HSQC or HMBC experiments. δ_C_ 162.1 (C-4′), 154.9 (C-6), 143.4 (C-4), 142.8 (C-4″), 134.9 (C-8a), 131.4 (C-7′), 127.1 (C-5″), 123.0 (C-5′), 121.7 (C-7), 121.5 (C-6′), 118.7 (C-3), 117.9 (C-8), 115.5 (C-8′), 106.2 (C-5), 62.3 (-O-CH_2_), 55.8 (OCH_3_). C-2, 2′, 3′, 4a, 4a′, and 8a′ were not observed. EI-MS (*m*/*z*,%): 415 (M^+^, 30). *Anal. Calcd for* C_22_H_17_N_5_O_4_: C, 63.61; H, 4.12; N, 16.86. Found: C, 63.72; H, 4.29; N, 16.99.

1-Methyl-4-(4-(((2-oxo-1,2-dihydroquinolin-4-yl)oxy)methyl)-1*H*-1,2,3-triazol-1-yl)quinolin-2(1*H*)-one (**6d**). White solid, yield: 270 mg, 68%; mp 285–287 °C; ^1^H-NMR (DMSO-*d*_6_): δ_H_ 11.43 (bs, 1H; NH-1′), 8.99 (s, 1H; H-5″), 7.84 (d, *J* = 8.0, 1H; H-5′), 7.80 (dd, *J* = 7.8, 7.8, 1H; H-7), 7.74 (d, *J* = 8.4, 1H; H-8), 7.52 (dd, *J* = 8.0, 8.0, 1H; H-7′), 7.51 (d, *J* = 8.1 Hz; 1H, H-5), 7.36 (dd, *J* = 7.5, 7.5, 1H; H-6), 7.30 (d, *J* = 8.2, 1H; H-8′), 7.16 (dd, *J* = 7.8, 7.8, 1H; H-6′), 7.04 (s, 1H; H-3), 6.22 (s, 1H; H-3′), 5.48 (s, 2H; -O-CH_2_), 3.74 (s, 3H; N-CH_3_). ^13^C-NMR (DMSO-*d*_6_): δ_C_ 163.12 (C-2′), 161.75 (C-4′), 160.30 (C-2), 142.59 (C-4), 142.41 (C-4″), 140.15 (C-8a), 138.67 (C-8a′), 132.33 (C-7), 131.02 (C-7′), 126.96 (C-5″), 124.50 (C-5), 122.75 (C-6), 122.49 (C-5′), 121.32 (C-6′), 117.30 (C-3), 115.57, 115.54 (C-4a, 8), 115.15 (C-8′), 114.41 (C-4a′), 97.93 (C-3′), 61.79 (-O-CH_2_), 29.59 (CH_3_). ^15^N-NMR (DMSO-*d*_6_): δ_N_ 247.0 (N-1″), 147.2 (N-1), 144.1 (N-1′). EI-MS (*m*/*z*,%): 399 (M^+^, 20). *Anal. Calcd for* C_22_H_17_N_5_O_3_: C, 66.16; H, 4.29; N, 17.53. Found: C, 65.99; H, 4.36; N, 17.49.

6-Methyl-4-((1-(2-oxo-1,2-dihydroquinolin-4-yl)-1*H*-1,2,3-triazol-4-yl)methoxy)quinolin-2(1*H*)-one (**6e**). Yellowish green, yield: 320 mg, 80%; mp 272–274 °C; ^1^H-NMR (DMSO-d_6_): δ_H_ 12.29 (bs, 1H; NH-1), 11.35 (bs, 1H; NH-1′); 8.99 (s, 1H; H-5″), 7.67 (dd, *J* = 7.6, 7.6, 1H; H-7), 7.61 (bs, 1H; H-5′), 7.51 (d, *J* = 9.1, 1H; H-5), 7.49 (d, *J* = 9.2, 1H; H-8), 7.35 (d, *J* = 8.2, 1H; H-7′), 7.27 (dd, *J* = 7.6, 7.6, 1H; H-6), 7.21 (d, *J* = 8.2, 1H; H-8′), 6.91 (s, 1H; H-3), 6.19 (s, 1H; H-3′), 5.46 (s, 2H; -O-CH_2_), 2.33 (s, 3H; H-6a′). ^13^C-NMR (DMSO-*d*_6_): δ_C_ 163.01 (C-2′), 161.62 (C-4′), 160.97 (C-2), 143.61 (C-4), 142.36 (C-4″), 139.45 (C-8a), 136.68 (C-8a′), 132.21 (C-7′), 131.90 (C-7), 130.38 (C-6′), 126.81 (C-5″), 124.03 (C-5), 122.61 (C-6), 121.81 (C-5′), 117.84 (C-3), 115.94 (C-8), 115.13 (C-8′), 114.47 (C-4a), 114.29 (C-4a′), 97.90 (C-3′), 61.68 (-O-CH_2_), 20.45 (CH_3_). ^15^N-NMR (DMSO-*d*_6_) δ_N_ 247.8 (N-1″), 152.2 (N-1), 143.2 (N-1′). EI-MS (*m*/*z*,%): 399 (M^+^, 22). *Anal. Calcd for* C_22_H_17_N_5_O_3_: C, 66.16; H, 4.29; N, 17.53. Found: C, 66.09; H, 4.44; N, 17.38.

6-Methyl-4-((1-(6-methyl-2-oxo-1,2-dihydroquinolin-4-yl)-1*H*-1,2,3-triazol-4-yl)meth-oxy)quinolin-2(1*H*)-one (**6f**). White solid, yield: 363 mg, 88%; mp 270–272 °C; ^1^H-NMR (DMSO-*d*_6_): δ_H_ 12.22 (bs, 1H; NH-1), 11.36 (bs, 1H; NH-1′), 8.98 (s, 1H; H-5″), 7.61 (bs, 1H; H-5′), 7.49 (d, *J* = 8.0, 1H; H-7), 7.39 (d, *J* = 8.0, 1H; H-8), 7.34 (d, *J* = 8.1, 1H; H-7′), 7.26 (bs, 1H; H-5), 7.20 (d, *J* = 8.1, 1H; H-8′), 6.87 (s, 1H; H-3), 6.19 (s, 1H; H-3′), 5.46 (s, 2H; -O-CH_2_), 2.32 (s, 3H; H-6a′), 2.31 (s, 3H; H-6a). ^13^C-NMR (DMSO-*d*_6_): δ_C_ 163.04 (C-2′), 161.61 (C-4′), 160.83 (C-2), 143.41 (C-4), 142.36 (C-4″), 137.53 (C-8a), 136.69 (C-8a′), 133.19 (C-7), 132.19 (C-7′), 131.79 (C-6), 130.37 (C-6′), 126.81 (C-5″), 123.14 (C-5), 121.81 (C-5′), 117.87 (C-3), 115.92 (C-8), 115.14 (C-8′), 114.40, 114.31 (C-4a′, 4a), 97.91 (C-3′), 61.71 (-O-CH_2_), 20.52 (C-6a), 20.45 (C-6a′). ^15^N-NMR (DMSO-*d*_6_): δ_N_ 248.0 (N-1″), 151.5 (N-1), 143.2 (N-1′). EI-MS (*m*/*z*,%): 413 (M^+^, 10). *Anal. Calcd for* C_23_H_19_N_5_O_3_: C, 66.82; H, 4.63; N, 16.94. Found: C, 66.98; H, 4.77; N, 16.99.

6-Methoxy-4-((1-(2-oxo-1,2-dihydroquinolin-4-yl)-1*H*-1,2,3-triazol-4-yl)methoxy)quinolin-2-(1*H*)-one (**6g**). Pale yellow, yield: 270 mg, 65%; mp 300–302 °C; ^1^H-NMR (DMSO-*d*_6_) δ_H_ 12.29 (bs, 1H; NH-1), 11.33 (bs, 1H; NH-1′), 8.99 (s, 1H; H-5″), 7.66 (dd, *J* = 7.3, 7.3, 1H; H-7), 7.49 (m; 2H, H-5, 8), 7.26 (m; 3H, H-6, 5′, 8′), 7.21 (m; 1H, H-7′), 6.90 (s; 1H, H-3), 6.22 (s, 1H; H-3′), 5.49 (s; 2H, -O-CH_2_), 3.77 (s, 3H; -OCH_3_). ^13^C-NMR (DMSO-*d*_6_): δ_C_ 162.67 (C-2′), 161.26 (C-4′), 160.95 (C-2), 153.97 (C-6′), 143.60 (C-4), 142.41 (C-4″), 139.45 (C-8a), 133.18 (C-8a′), 131.89 (C-7), 126.76 (C-5″), 123.98 (C-5), 122.58 (C-6), 119.90 (C-7′), 117.86 (C-3), 116.62 (C-8′), 115.94 (C-8), 114.82, 114.48 (C-4a, 4a′), 104.33 (C-5′), 98.41 (C-3′), 61.79 (-O-CH_2_), 55.51 (-OCH_3_). ^15^N-NMR (DMSO-*d*_6_): δ_N_ 247.9 (N-1″), 142.7 (N-1). EI-MS (*m*/*z*,%): 415 (M^+^, 60). *Anal. Calcd for* C_22_H_17_N_5_O_4_: C, 63.61; H, 4.12; N, 16.86. Found: C, 63.77; H, 4.10; N, 17.00.

## 5. Biochemical Assay

### 5.1. Assay of Antioxidant Biomarkers in Testis (MDA and TAC)

TAC was assessed by the colorimetric technique using commercial kits (El-Minia, Egypt) (See Supplementary Materials). Testicular MDA, an index of lipid peroxidation, was determined by using 1,1,3,3-tetramethoxypropane as standard [70] (See Supplementary Materials).

### 5.2. Assay of Apoptotic Biomarkers (Testicular Testosterone, TNF-α, and Caspase-3)

TNF-α was measured by ELISA kit (Elabscience, Houston, TX, USA). Caspase-3 was measured by ELISA kit Cusabio, Houston, TX, USA (See Supplementary Materials). Testosterone concentration in testicular samples was determined by the ELISA kit (“DRG”, Marburg, Germany) (See Supplementary Materials).

### 5.3. Assay of Caspases

#### 5.3.1. Assay of Caspase-3, 8, and 9 Inhibition

The Caspase-3 Inhibitor Drug Screening Kit (Catalog #JM-K153-100; 15 B Constitution Way Woburn, MA 01801, USA) provides an effective means for screening caspase inhibitors using fluorometric methods. The assay utilizes synthetic peptide substrate DEVD-AFC (AFC, 7-amino-4-trifluoromethylcoumarin). Active caspase-3 cleaves the synthetic substrate to release free AFC, which can then be quantified by fluorometry. Compounds to be screened can directly be added to the reaction and the level of inhibition of caspase-3 activity can be determined by comparison of the fluorescence intensity in samples with and without the testing inhibitors. (See Supplementary Materials).

#### 5.3.2. Assay of Cytochrome C

Cells were collected from American Type Culture Collection to be grown in SR containing 10% fetal bovine serum at 37 °C, stimulated with the compounds to be tested for cytochrome C using Cytochrome C Human ELISA Kit (ab119521—Cytochrome C Human ELISA Kit, Vaccera institute, Egypt) [57] (See Supplementary Materials).

### 5.4. Histopathological Investigation

Multiple testicular specimens were stained by hematoxylin-eosin (ab245880, Abcam, Cambridge, MA, USA) using Cosentino’s score [59] for semi-quantitation of pathological changes in different seminiferous tubules. By using Johnson’s scoring system [59], the effect of ischemia on the spermatogenesis can be studied (See Supporting Materials).

#### 5.4.1. Photography

An Olympus light microscopy and digital camera (Olympus, Markham, ON, Canada), were used in this study. Images were executed using Adobe Photoshop.

#### 5.4.2. Morphometric Study

Semi-quantitative data were measured and counted per section in ten randomly selected non-overlapping fields using power × 400 magnifications of the sections from each rat [71]. The results were carried out using the Image J program analysis software (Image J 1.48V, Maryland, MD, USA*;* Wayne Rasb and National Institutes of Health).

#### 5.4.3. Statistical Analysis

Data were evaluated as means (standard error of the mean). One-way analysis of variance (ANOV) with the use of Turkey’s post-test was carried out for the analysis of the results to detect the significant difference statistically. *p* values < 0.05 were considered significant.

### 5.5. Computational Analysis

#### 5.5.1. Molecular Docking Study

The docking simulation study was carried out using Molecular Operating Environment (MOE^®^) version 2014.09, Chemical Computing Group Inc., Montreal, QC, Canada). The computational software operated under “Windows XP” installed on an Intel Pentium IV PC with a 1.6 GHz processor and 512 MB memory. The target compounds were constructed into a 3D model using the builder interface of the MOE program and docked into the active site of caspase-3 (PDB: 3GJQ). Checking their structures and the formal charges on atoms by 2D depiction was carried out and the energy, was minimized until an RMSD (root mean square deviations) gradient of 0.01 Kcal/mol and RMS (Root Mean Square) distance of 0.1 A with MMFF94X (Merck molecular force field 94x) force-field and the partial charges were automatically calculated (See Supplementary Materials).

#### 5.5.2. Prediction of Physicochemical Properties, Pharmacokinetics, and Drug-Likeness Profile In Silico

The physicochemical and lipophilicity of the target compounds were collected using Swiss ADME software (http://www.swissadme.ch/index.php) and Molinspiration software (https://preadmet.bmdrc.kr/). In addition, Lipinski drug-likeness of the target compounds were obtained by using Molsoft software (https://www.molsoft.com/) and Swiss ADME software. The percent of absorption was calculated through this equation:

%ABS = 109 − 0.345 tPSA(2)

where TPSA: topological polar surface area; %ABS: percentage of absorption.

### 5.6. Ethical Approval

Minia University Faculty of Medicine, Research Ethics Committee “BFMREC” approved this research proposal regarding the source of the animals, health status, inclusion criteria, exclusion criteria, caging, comfort, and the detailed experimental design and procedures. It was conducted in agreement with the NIH Guide for Care and Use of Laboratory Animals. The approval number is: 251-2/2018.

## 6. Conclusions

A series of biquinoline-2-one/1,2,3-triazole hybrids **6a**–**g** have been synthesized and identified with different spectroscopic techniques. The antiapoptotic activity of the target compounds in the testis was biochemically evaluated compared with the reference NAC. The most robust compounds were **6a**–**c,** revealing promising antiapoptotic and antioxidant readings. Moreover, they exhibited a caspase-3 inhibition level of about 2.66, 2.94, and 2.39 ng/mL, respectively. Compound **6c** can be evaluated as a hit for further screenings to acquire more active antiapoptotic agents due to its selectivity toward caspase-3 rather than caspase-8 and -9 as well as a higher cytochrome C potency with conc 0.28 ng/mL. Further histopathological study was performed in a specimen of testis treated with the tested compounds. Furthermore, the molecular docking study was performed on the caspase-3 active site revealing a good interaction with the enzyme. Finally, a computational prediction of physicochemical properties, pharmacokinetics, and drug-likeness profile in silico revealed that compound **6a**–**c** should be taken into consideration as good antiapoptotic candidates for further study.

## Figures and Tables

**Table 1:** ^1^H-^15^N HSQC correlations in Series **5** and **6.**

Compound	δ_H_ (H-1)	δ_N_ (N-1)	Solvent
**5a**	144.1 145.3	11.44 11.95	DMSO-*d*_6_ CDCl_3_
**5b**	144.1	11.37	DMSO-*d*_6_
**5c**	144.1	11.36	DMSO-*d*_6_
**6b**	144.0 (N-1′) 151.6	11.43 (H-1′) 12.29	DMSO-*d*_6_
**6d**	144.1 (N-1′)	11.43 (H-1′)	DMSO-*d*_6_
**6e**	143.2 (N-1′) 152.2	11.35 (H-1′) 12.29	DMSO-*d*_6_
**6f**	143.2 (N-1′) 151.5	11.36 (H-1′) 12.22	DMSO-*d*_6_

(Compounds not shown were too insoluble for H-N correlation experiments).

**Table 2:** NMR data for compound **6b**.

**^1^H-NMR** (DMSO-*d*_6_)	**^1^H-^1^H COSY**		**Assig.**
12.22 (bs; 1H)			H-1
11.44 (bs; 1H)			H-1′
8.98 (s; 1H)			H-5″
7.84 (d, *J* = 6.8; 1H)	7.15		H-5′
7.51-7.49 (m; 2H)	7.40, 7.30, 7.15		H-7,7′
7.40 (m; 1H)	7.50		H-8
7.30 (d, *J* = 7.4; 1H)	7.50		H-8′
7.27 (bs; 1H)	2.31		H-5
7.15 (m; 1H)	7.84, 7.51		H-6′
6.86 (s; 1H)			H-3
6.22 (s; 1H)			H-3′
5.48 (s; 2H)			H-4a″
2.31 (s; 3H)	7.27		H-6a
**^13^C-NMR** (DMSO-*d*_6_)	**HSQC**	**HMBC**	**Assig.**
163.16			C-2′
161.27		7.84, 622, 5.48	C-4′
160.83			C-2
143.38		7.27, 6.86	C-4
142.39		8.98, 5.47	C-4″
138.66		7.84, 7.51	C-8a′
137.52		7.49, 7.27	C-4
133.18	7.49	2.31	C-7
131.79			C-6
131.02	7.51	7.84	C-7′
126.73	8.98	5.48	C-5″
123.17	7.27	2.31	C-5
122.49	7.84	7.29	C-5′
121.32	7.15	7.30	C-6′
117.81	6.86		C-3
115.92			C-4a/4a′
115.16	7.40	7.27, 6.91	C-8
114.42	7.30	7.15, 6.22	C-8′, 4a′/4a
97.93	6.22		C-3′
61.81	5.48		C-4a″
20.52	2.31	2.31	C-6a
**^15^N-NMR** (DMSO-*d*_6_)	**HSQC**	**HMBC**	**Assig.**
248.0		8.98	N-1″
151.6	12.29		N-1
144.0	11.43		N-1′

**Table 3:** The expression of the biomarkers during apoptosis in testis.

* Biomarker	Effect During Apoptosis in Testis
TNFα [47], MDA [48], Caspase-3 [1]	Increase
TAC [49], Testosterone [50]	Decrease

* **TNF** = tumor necrosis factor, **MDA** = malondialdehyde, **TAC** = total antioxidant capacity.

**Table 4:** Effects of compound **6c** and NAC on cytochrome *C* in SR human cell line.

	Cyt *C*	
Code	Conc ng/mL	Fold Change
**6c**	0.28 ± 0.011	3.54
**NAC**	0.24 ± 0.019	4.13
**Control**	0.99 ± 0.036	6.03

**Table 5:** Physicochemical and lipophilicity of the target compounds using Swiss absorption, distribution, metabolism, and excretion (ADME) and Molinspiration software.

Compound	Physicochemical Properties									
	Lipophilicity Consensus log P	MW ^a^ g/mol	Heavy Atoms	Aromatic Heavy Atoms	Rot. Bond	H-Bond Acc.	H-Bond Don.	MR ^b^	TPSA^c^ (A2)	%ABS ^d^
**6a**	2.41	385.38	29	25	4	5	2	108.59	105.66	72.55
**6b**	2.45	399.40	30	25	4	5	1	113.49	94.80	76.29
**6c**	2.32	415.40	31	25	5	6	1	114.67	104.03	73.11
**6d**	2.73	399.40	30	25	4	5	2	113.56	105.66	72.55
**6e**	2.64	399.40	30	25	4	5	2	113.56	105.66	72.55
**6f**	2.72	413.43	31	25	4	5	1	118.46	94.80	76.29
**6g**	2.38	415.40	31	25	5	6	2	115.08	114.89	69.36

Abbreviation: **MW** ^a^, molecular weight; **MR** ^b^, molar refractivity; **TPSA** ^c^, topological polar surface area; %**ABS** ^d^**:** percentage of absorption.

**Table 6:** Lipinski drug-likeness of the target compounds using Molsoft and Swiss ADME software.

Compound	Log S ^a^	Drug Likeness Model Score	Lipinski Violations	Bioavailability Score
**6a**	−3.30	−0.23	0	0.55
**6b**	−3.48	0.45	0	0.55
**6c**	−3.53	0.36	0	0.55
**6d**	−3.60	−0.18	0	0.55
**6e**	−3.60	−0.18	0	0.55
**6f**	−3.78	0.31	0	0.55
**6g**	−3.36	−0.21	0	0.55

Log S ^a^: Log of solubility.

**Table 7:** ADME data of tested compounds calculated using preADMET software.

Compound	Pharmacokinetics					
	BBB ^a^	Caco-2 ^b^	HIA ^c^	MDCK ^d^	PPB ^e^	CYP 2D6 ^f^
**6a**	0.041	22.28	100.00	16.84	90.09	Non
**6b**	0.069	23.96	96.82	6.56	98.07	Non
**6c**	0.075	23.23	97.44	6.31	94.65	Non
**6d**	0.039	21.81	95.58	4.39	89.29	Non
**6e**	0.039	21.79	95.58	4.39	89.41	Non
**6f**	0.067	24.63	96.74	1.02	97.71	Non
**6g**	0.05	21.85	96.01	8.51	84.71	Non

**^a^ BBB:** blood–brain barrier penetration; **^b^ CACO-2:** permeability through cells derived from human colon adenocarcinoma; **^c^ HIA:** percentage human intestinal absorption; **^d^ MDCK:** permeability through Madin–Darby canine kidney cells; **^e^ PPB:** plasma protein binding; **^f^ CYP2D6:** cytochrome P450 2D6.

## Supplementary Materials

The following are available online, Figure S1: 2D and 3D diagrams illustrate the binding modes of the reference NAC and 6a-g interacted with the active site of caspase-3 (PDB: 3GJQ), Table S1: Effect of compounds 6c and NAC on the active Cell-based caspases-3, 8 and 9 in MOLT-4 cell line, Table S2: Energy scores for the complexes formed by the tested compounds 6a-g and the reference NAC in the active site of caspase-3 (PDB: 3GJQ), Table S3: Testicular MDA and Testicular TAC concentrations in testis of I/R rats treated with compounds **6a**–**g** and NAC, Table S4: Testicular testosterone and TNFα concentrations in testis of I/R rats treated with compounds **6a**–**g** and NAC, Table S5: Caspase-3 level in serum of testicular I/R rats treated with compounds **6a**–**g** and NAC.
