# Supplementary material for: Design, Synthesis, Molecular Docking, Antiapoptotic and Caspase-3 Inhibition of New 1,2,3-Triazole/Bis-2(1H)-Quinolinone Hybrids
Source: Molecules. 2020 Oct 30;25(21):5057. doi: 10.3390/molecules25215057 (PMC7672604; doi:10.3390/molecules25215057)
Supplement: Supplementary file 1 [file molecules-25-05057-s001.zip › molecules-969911-supplementary.pdf]

Supporting Information

Design, Synthesis, Molecular Docking, Anti-Apoptotic and Caspase-3 Inhibition of New 1,2,3-Triazole/*Bis*-2(1*H*)-quinolinone Hybrids

Essmat M. El-Sheref,[1] Ashraf A. Aly,[1]* Mohammed B Alshammari,[2] Alan B. Brown,[3] Sara Mohamed Naguib Abdelhafez,[1] Walaa Yehia Abdelzaher,[1] Stefan Bräse[4,5]* and El-Shimaa M. N. Abdelhafez[1]

[1] Essmat M. El-Sheref, Ashraf A. Aly, Chemistry Department, Faculty of Science, Minia University, 61519-El-Minia, Egypt; e-mails: e.m.elsheref1980@gmail.com; ashrafaly63@yahoo.com,ashraf.shehata@mu.edu.eg;
Sara Mohamed Naguib Abdelhafez, Faculty of Medicine, Histology Department, Minia University, 61519-El-Minia, Egypt, e-mail: sara_histology@yahoo.com
Walaa Yehia Abdelzaher Faculty of Medicine, Pharmacology Department, Minia University, 61519-El-Minia, Egypt, e-mail: walaayehia22@yahoo.com;
El-Shimaa M. N. Abdelhafez, Department of Medicinal Chemistry, Faculty of Pharmacy, Minia University, 1519 El-Minia, Egypt, e-mail: shimaanaguib_80@yahoo.com
[2] College of Sciences and Humanities, Prince Sattam bin Abdulaziz University, Alkharj 11942, Saudi Arabia; e-mail: m.alshammari@psau.edu.sa
[3] Florida Institute of Technology,150 W University Blvd, Melbourne, FL 32901, USA, e-mail: abrown@fit.edu.
[4,5] Institute of Organic Chemistry, Karlsruhe Institute of Technology, 76131 Karlsruhe, Germany, Institute of Biological and Chemical Systems (IBCS-FMS), Karlsruhe Institute of Technology, Eggenstein-Leopoldshafen, Germany, e-mail: stefan.braese@kit.edu

Spectroscopic data.

SI Fig. 1. ^{1}H NMR spectrum (DMSO-d_6) of compound **5a**.

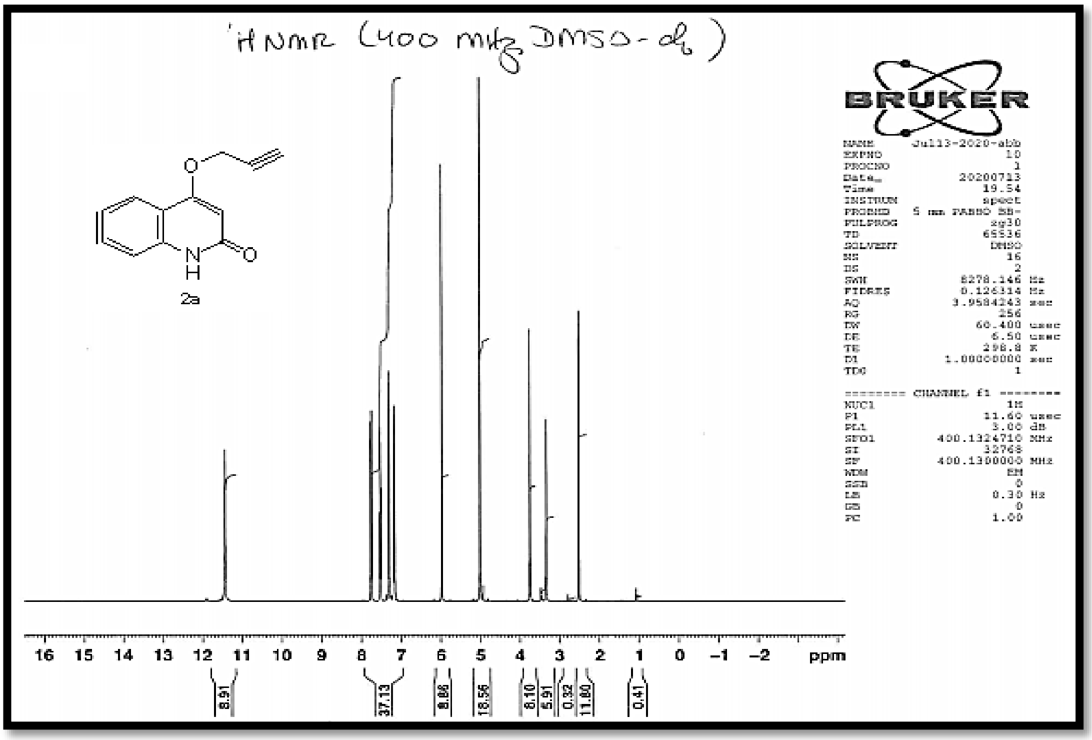

SI Fig. 2. A section of ^{1}H NMR spectrum (DMSO-d_6) of compound **5a**.

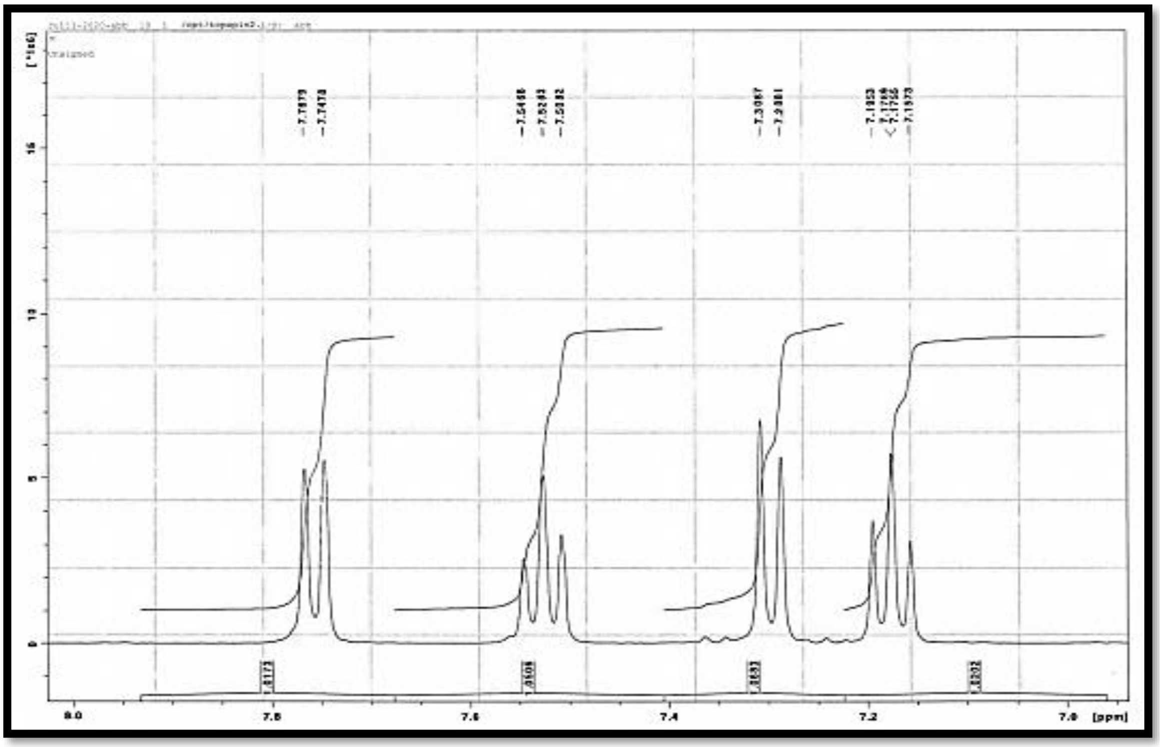

SI Fig. 3. ^{13}C NMR spectrum (DMSO-d_6) of compound **5a**.

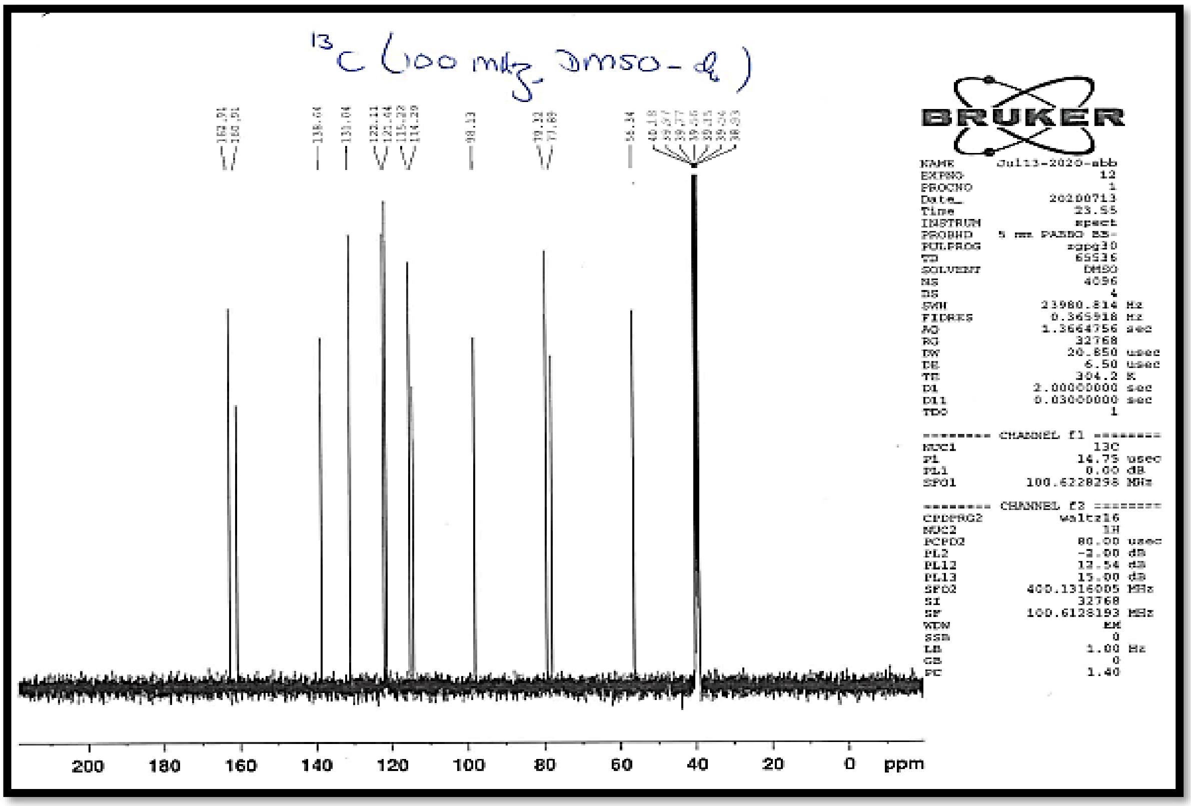

SI Fig. 4. ^{1}H ^{1}H COSY spectrum (DMSO-d_6) of compound **5a**.

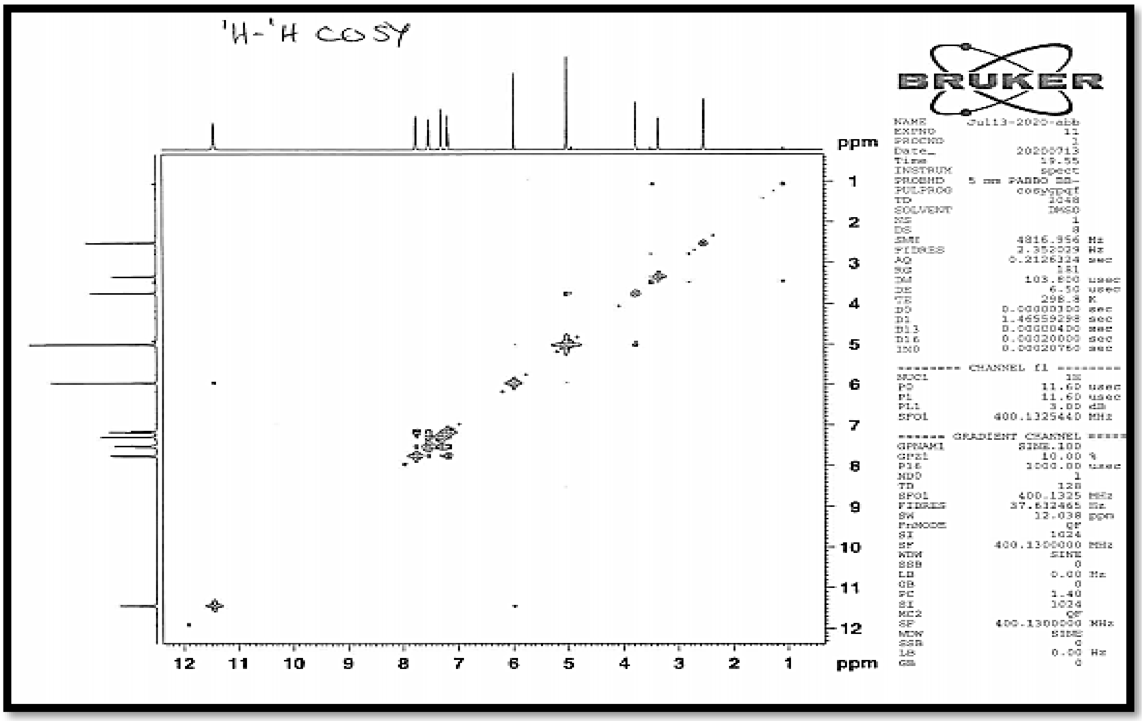

SI Fig. 5. A section of ^{1}H ^{1}H COSY spectrum (DMSO-d_6) of compound **5a**.

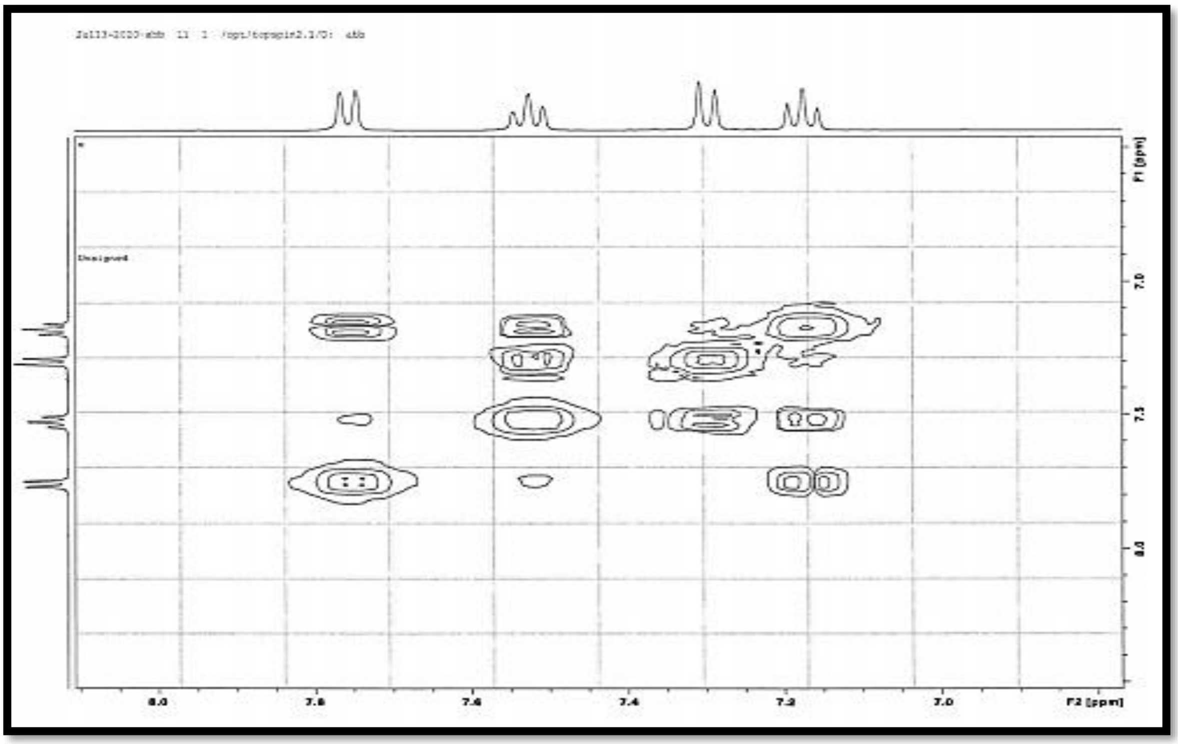

SI Fig. 6. ^{1}H ^{13}C HSQC (DMSO-d_6) of compound **5a**.

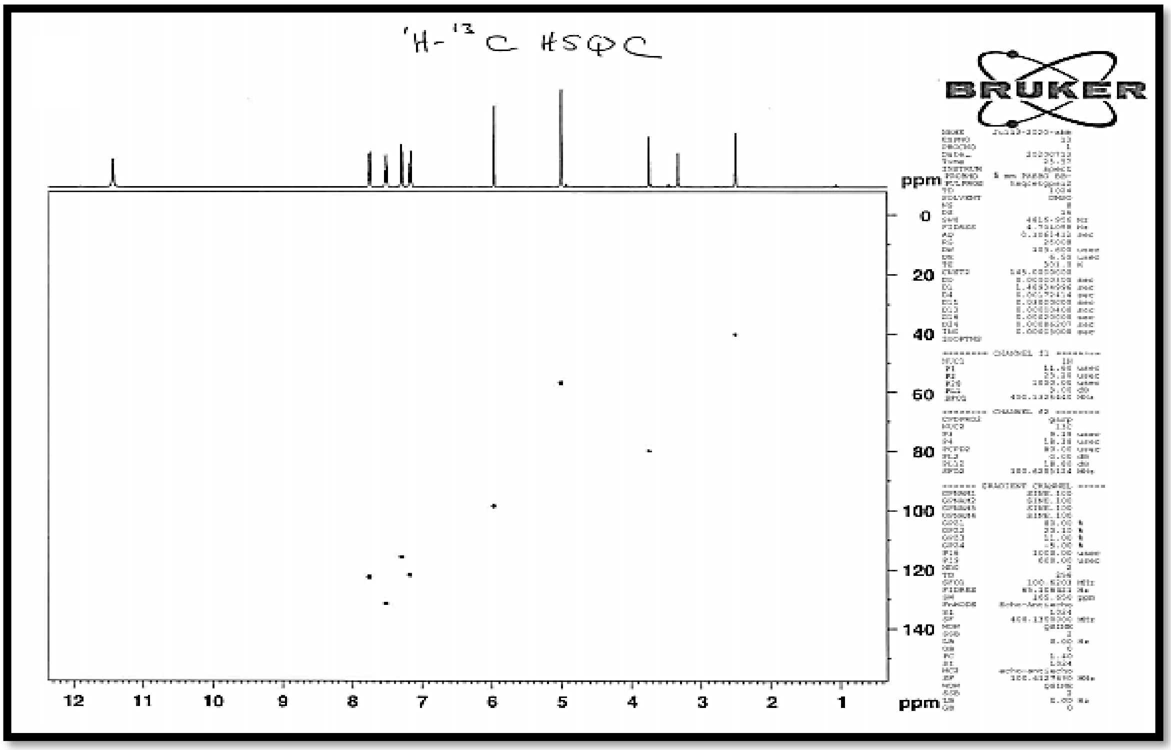

SI Fig. 7. ^{1}H ^{13}C HMBC spectrum (DMSO-d_6) of compound **5a**.

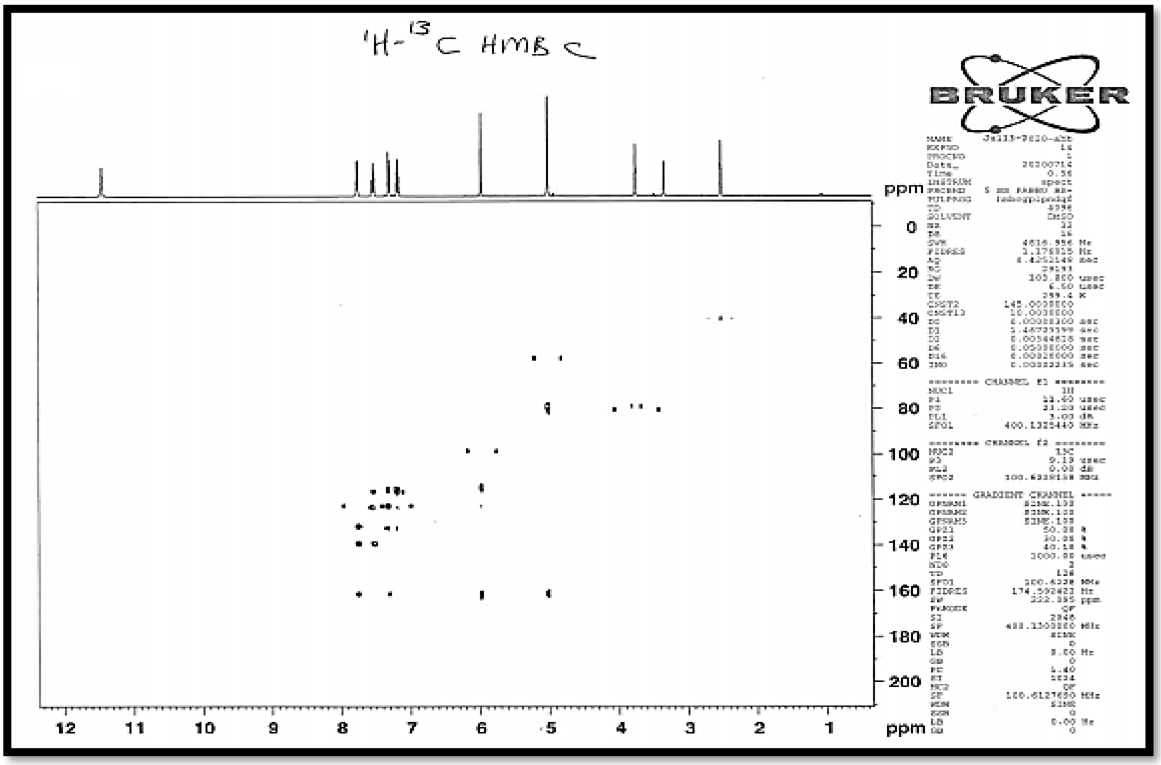

SI Fig. 8. ^{1}H ^{15}N HSQC spectrum (DMSO-d_6) of compound **5a**.

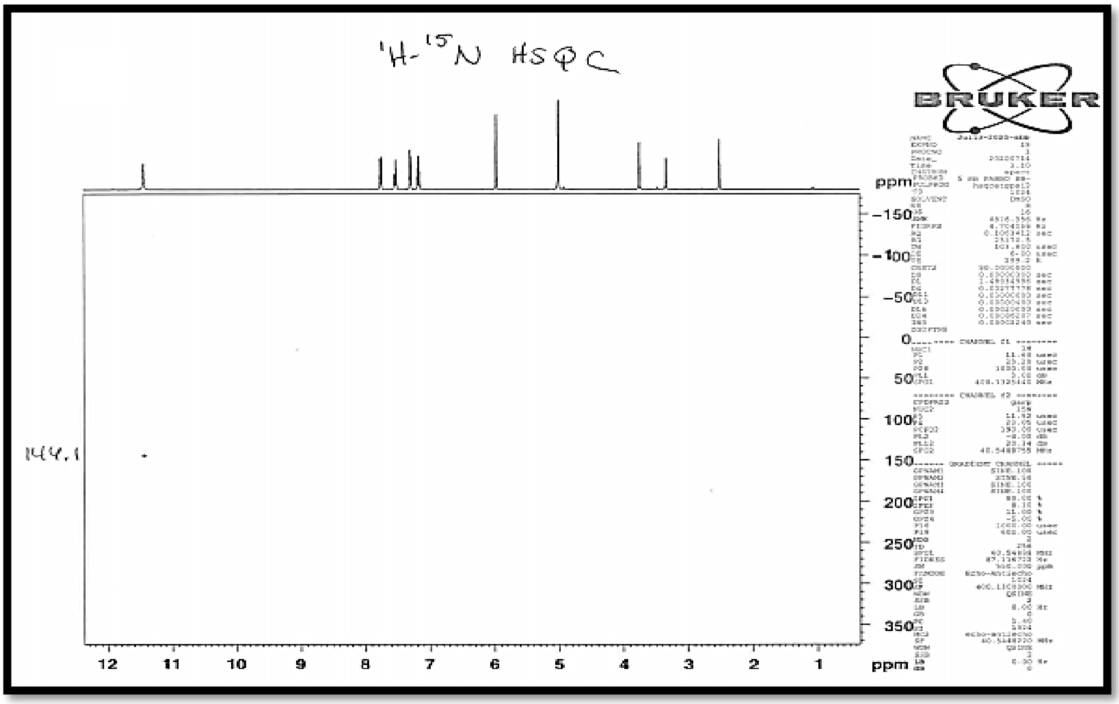

SI Fig. 9. ^{1}H NMR spectrum ($CDCl_3$) of compound **5a**.

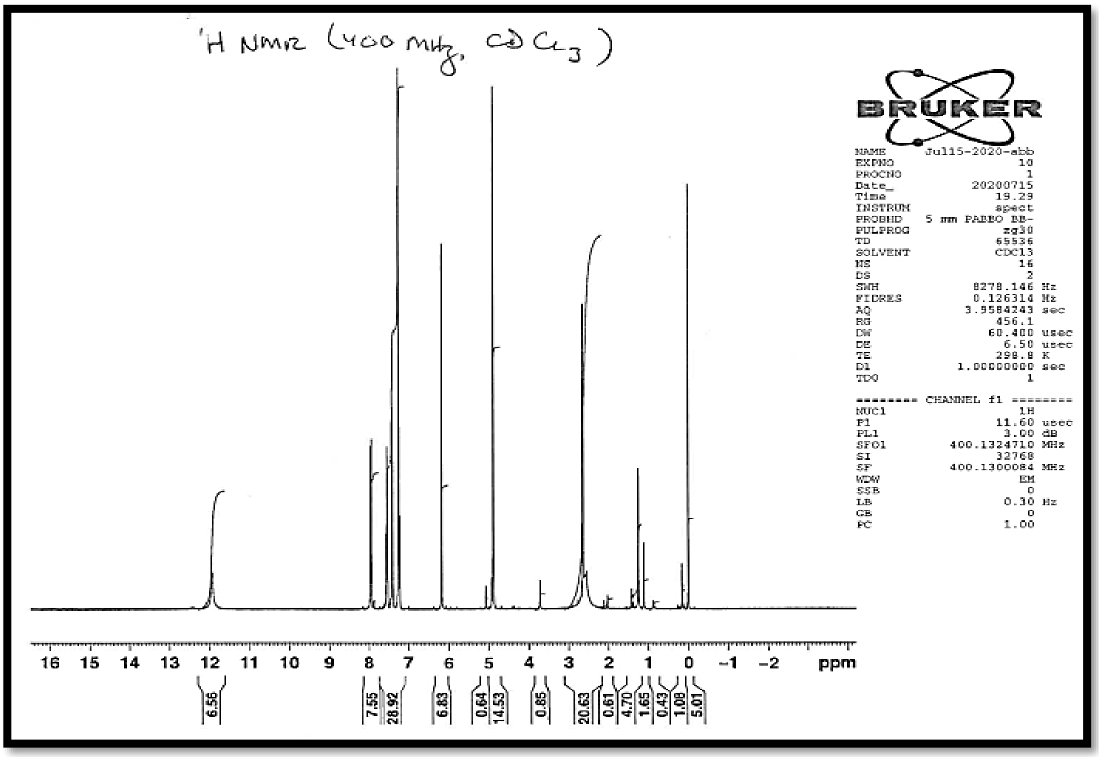

SI Fig. 10. A section of ^{1}H NMR spectrum ($CDCl_3$) of compound **5a**.

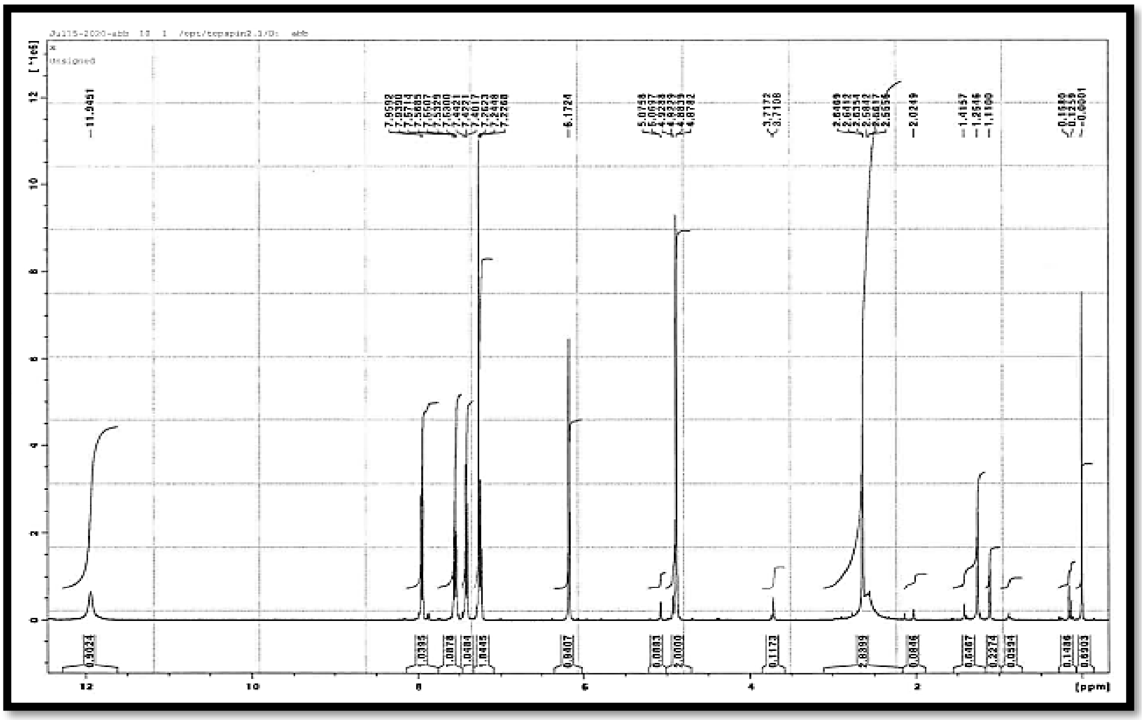

SI Fig. 11. ^{13}C NMR spectrum ($CDCl_3$) of compound **5a**.

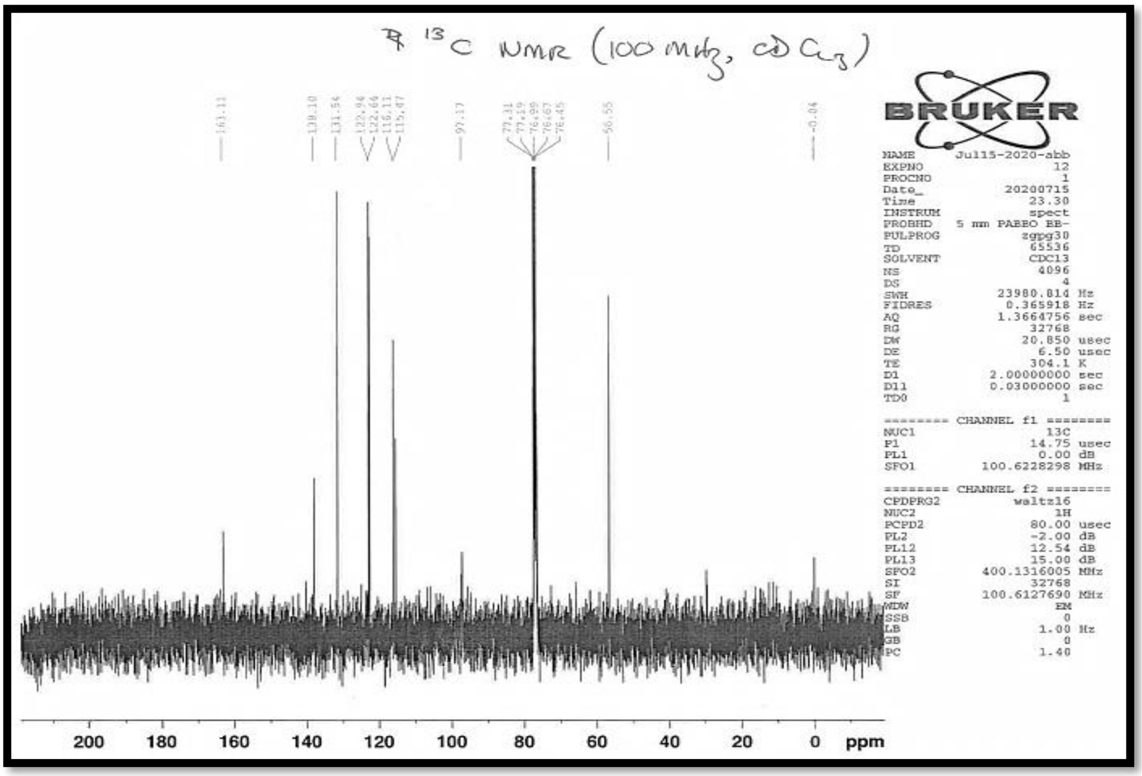

SI Fig. 12. ^{1}H ^{13}C HSQC spectrum ($CDCl_3$) of compound **5a**.

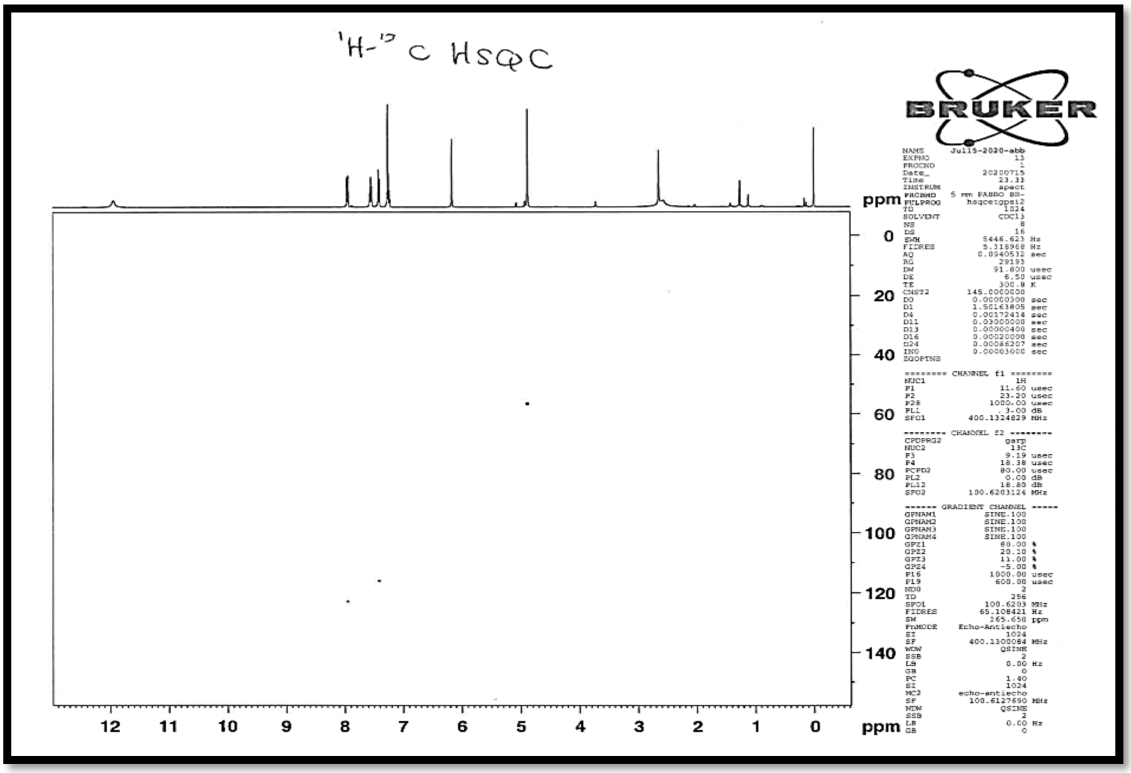

SI Fig. 13. A section of ^{1}H ^{13}C HSQC spectrum ($CDCl_3$) of compound **5a**.

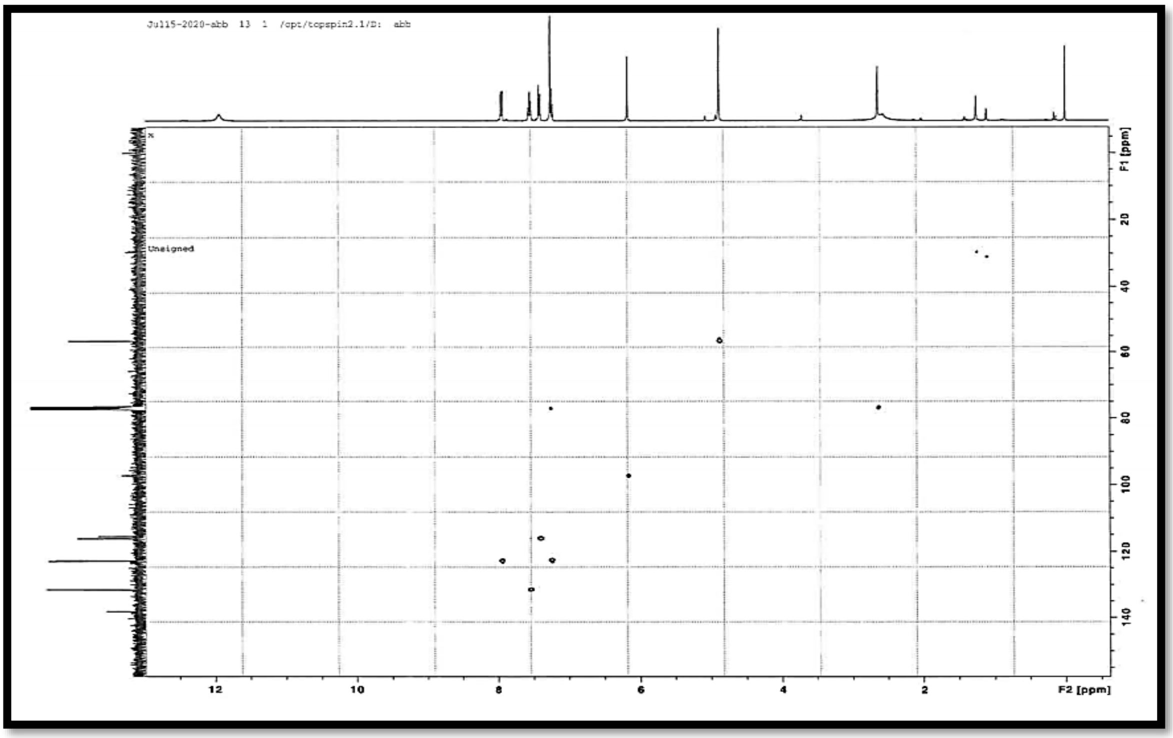

SI Fig. 14. ^{1}H ^{15}N HSQC spectrum ($CDCl_3$) of compound **5a**.

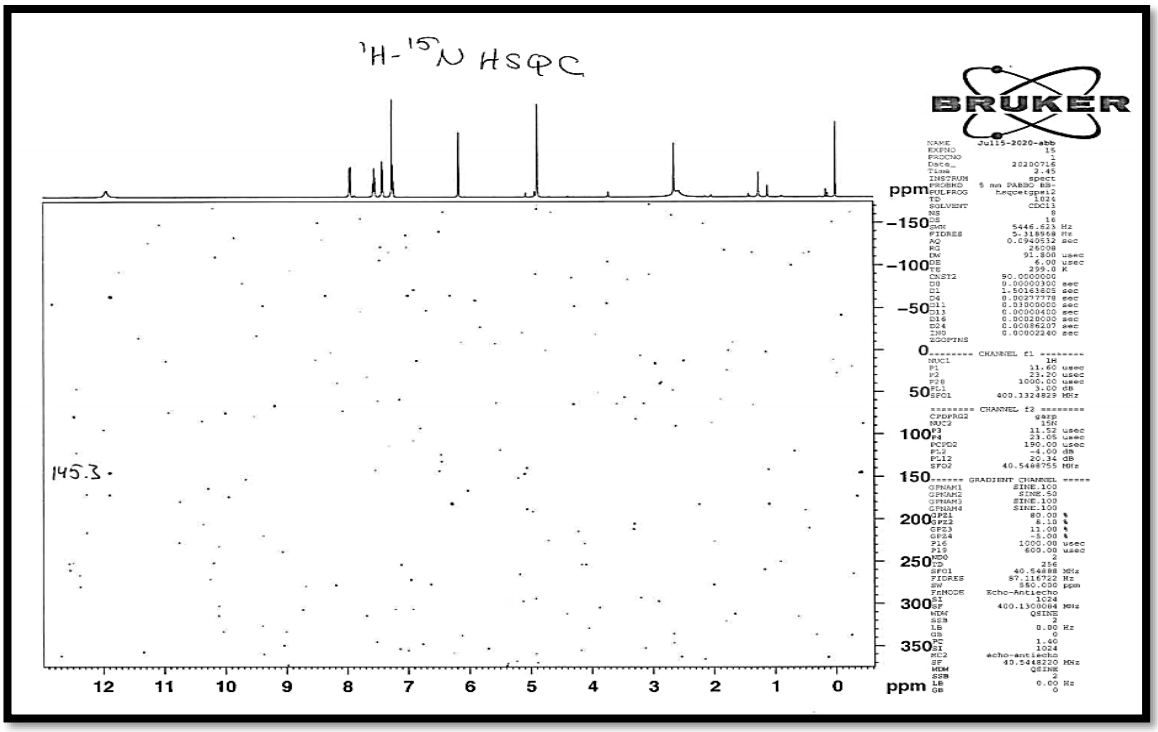

SI Fig. 15. ^{1}H NMR spectrum (DMSO-d_6) of compound **5b**.

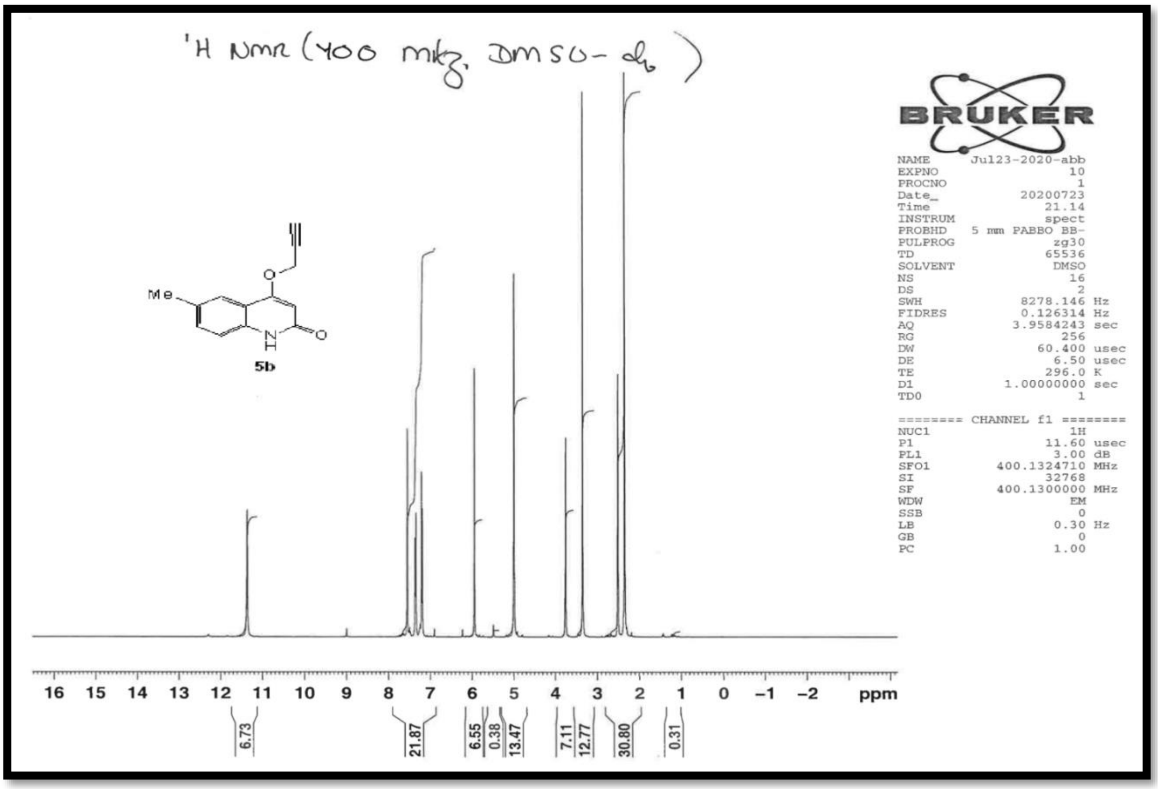

SI Fig. 16. A section of ^{1}H NMR spectrum (DMSO-d_6) of compound **5b**.

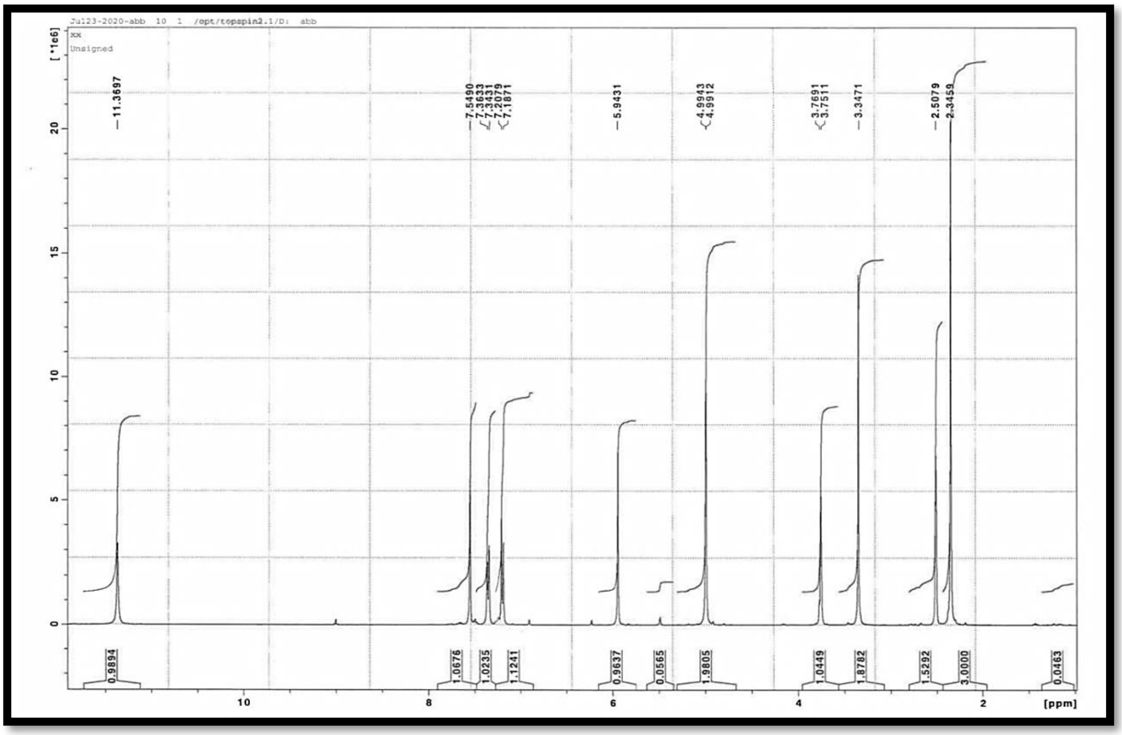

SI Fig. 17. ^{13}C NMR spectrum (DMSO-d_6) of compound **5b**.

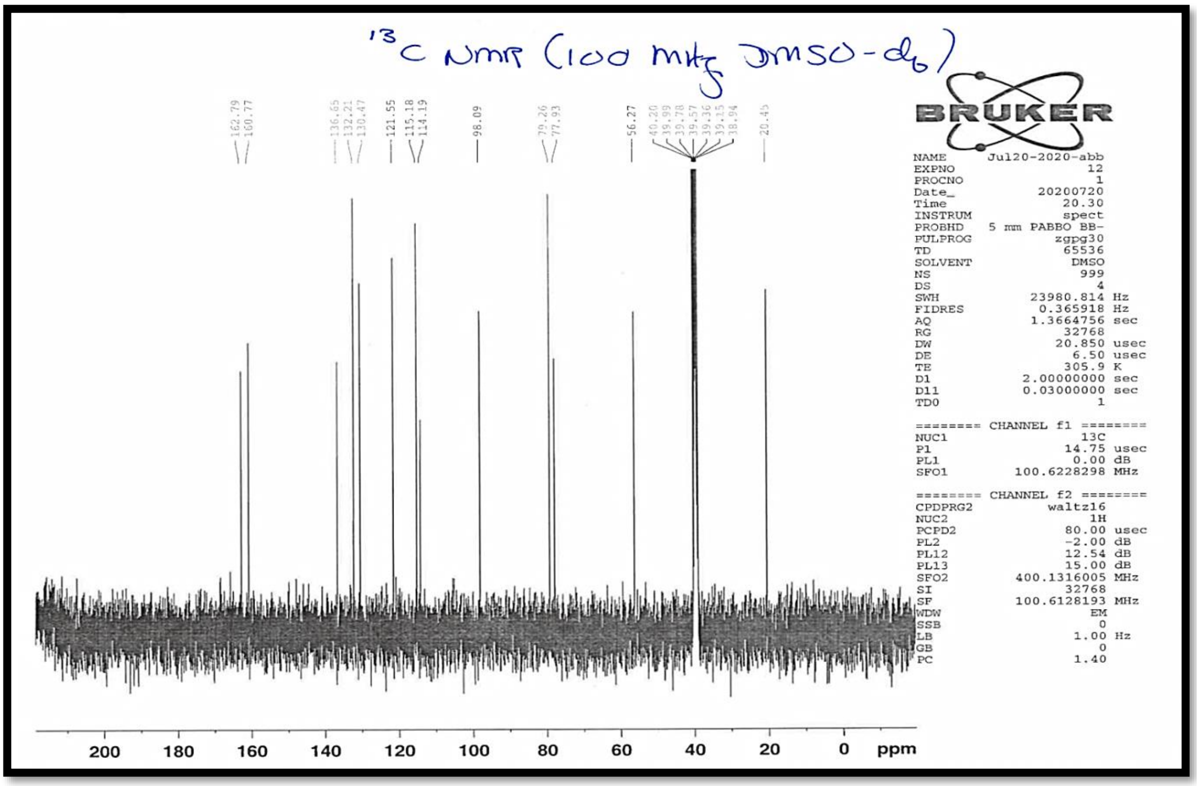

SI Fig. 18. ^{1}H ^{1}H Cosy spectrum (DMSO-d_6) of compound **5b**.

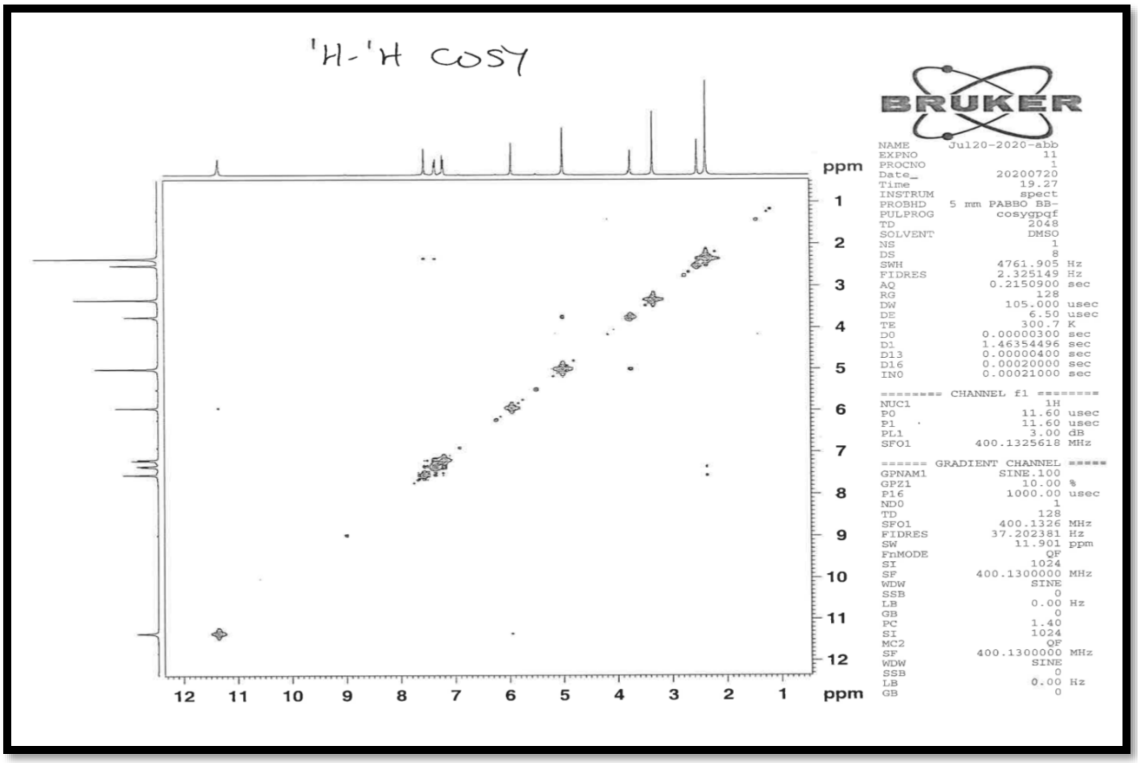

SI Fig. 19. A section of ^{1}H ^{1}H Cosy spectrum (DMSO-d_6) of compound **5b**.

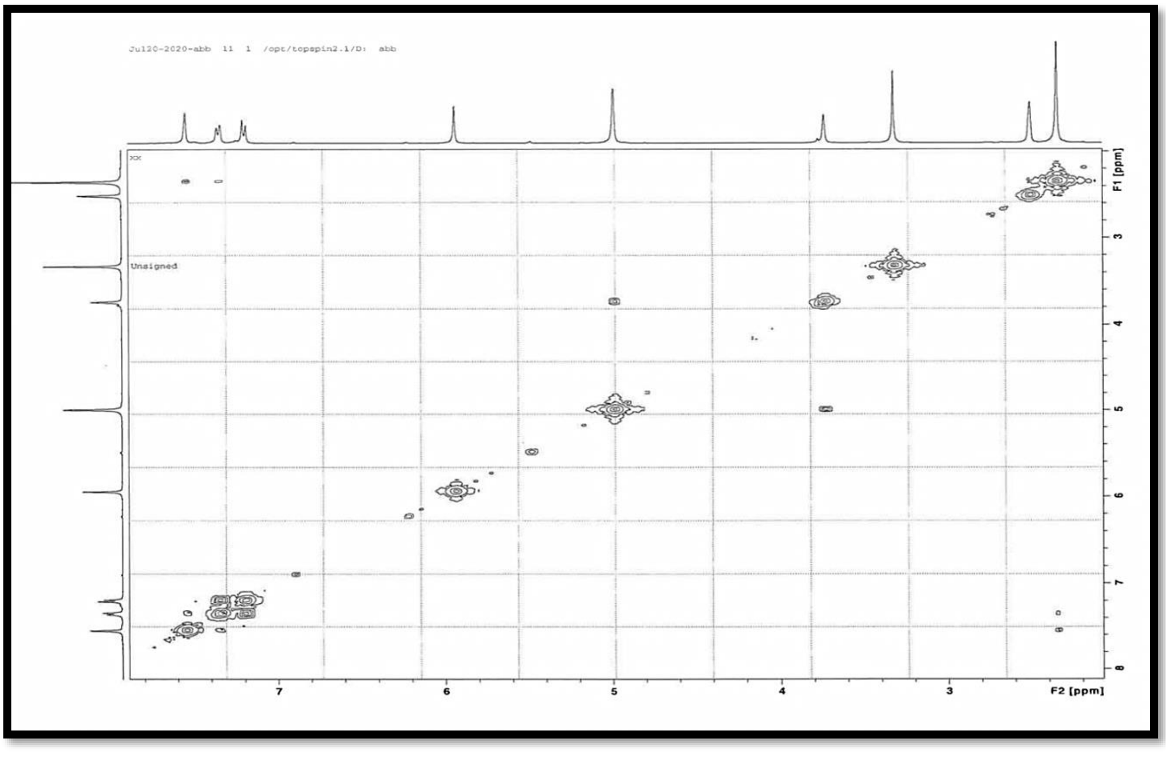

SI Fig. 20. ^{1}H ^{15}N HSQC spectrum (DMSO-d_6) of compound **5b**.

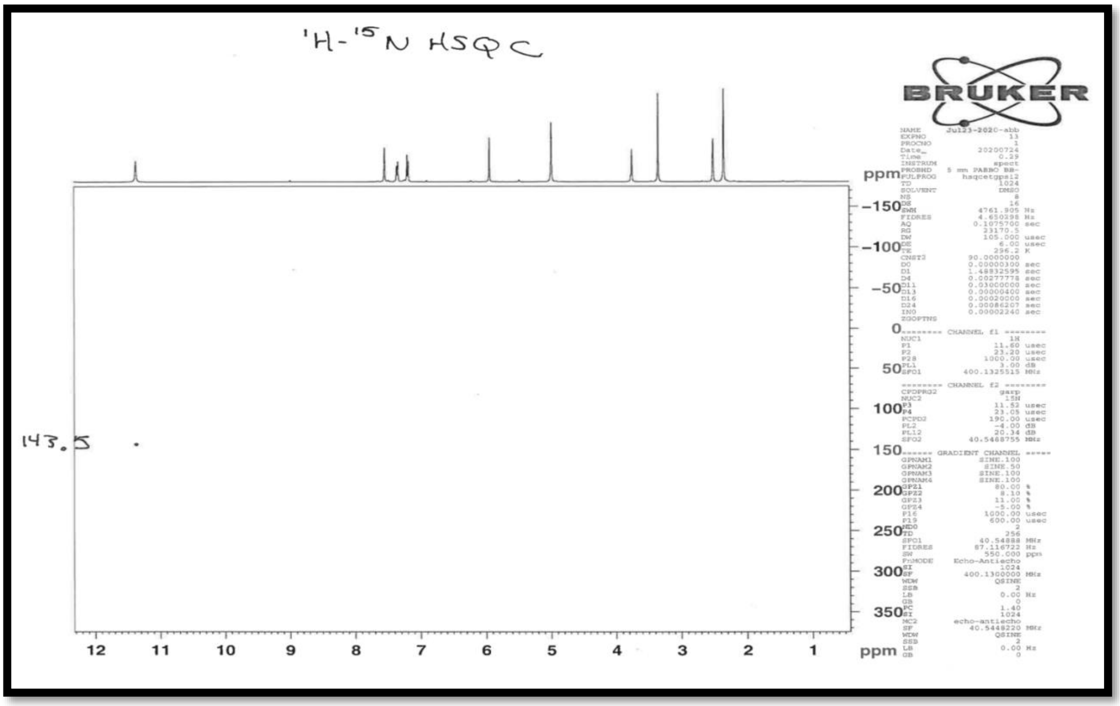

SI Fig. 21. A section of ^{1}H ^{15}N HSQC spectrum (DMSO-d_6) of compound **5b**.

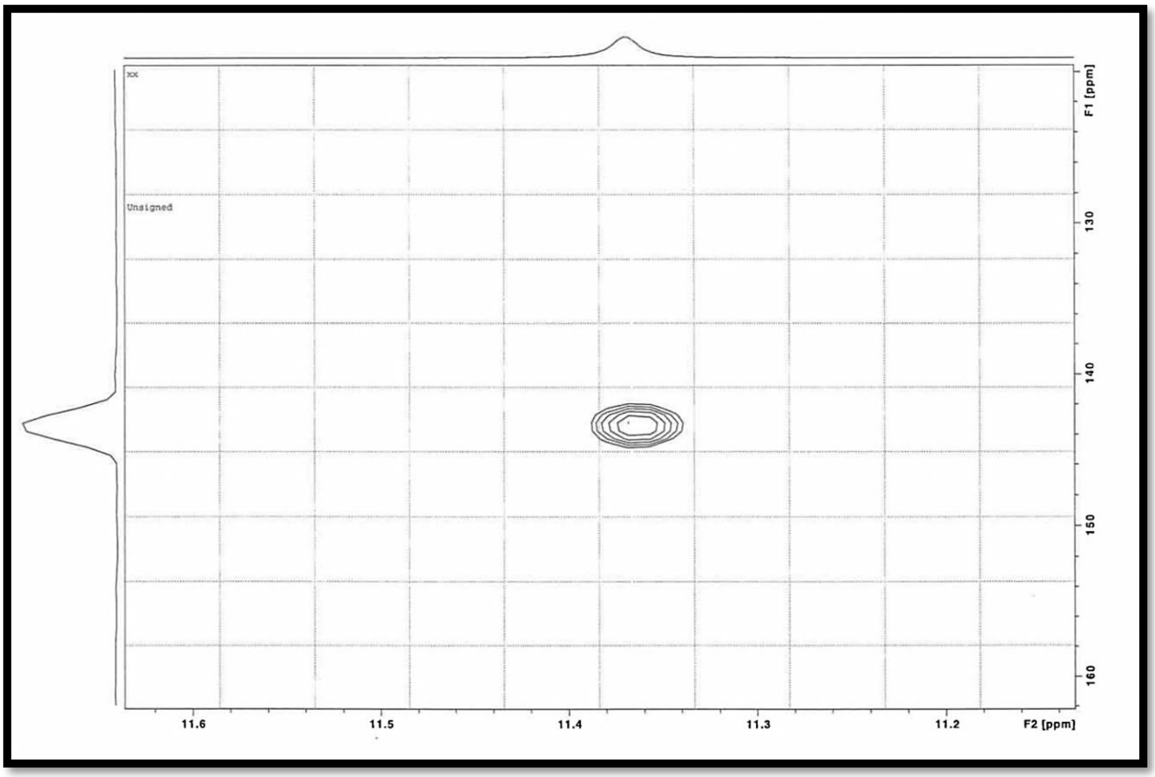

SI Fig. 22. ^{1}H ^{15}N HMBC spectrum (DMSO-d_6) of compound **5b**.

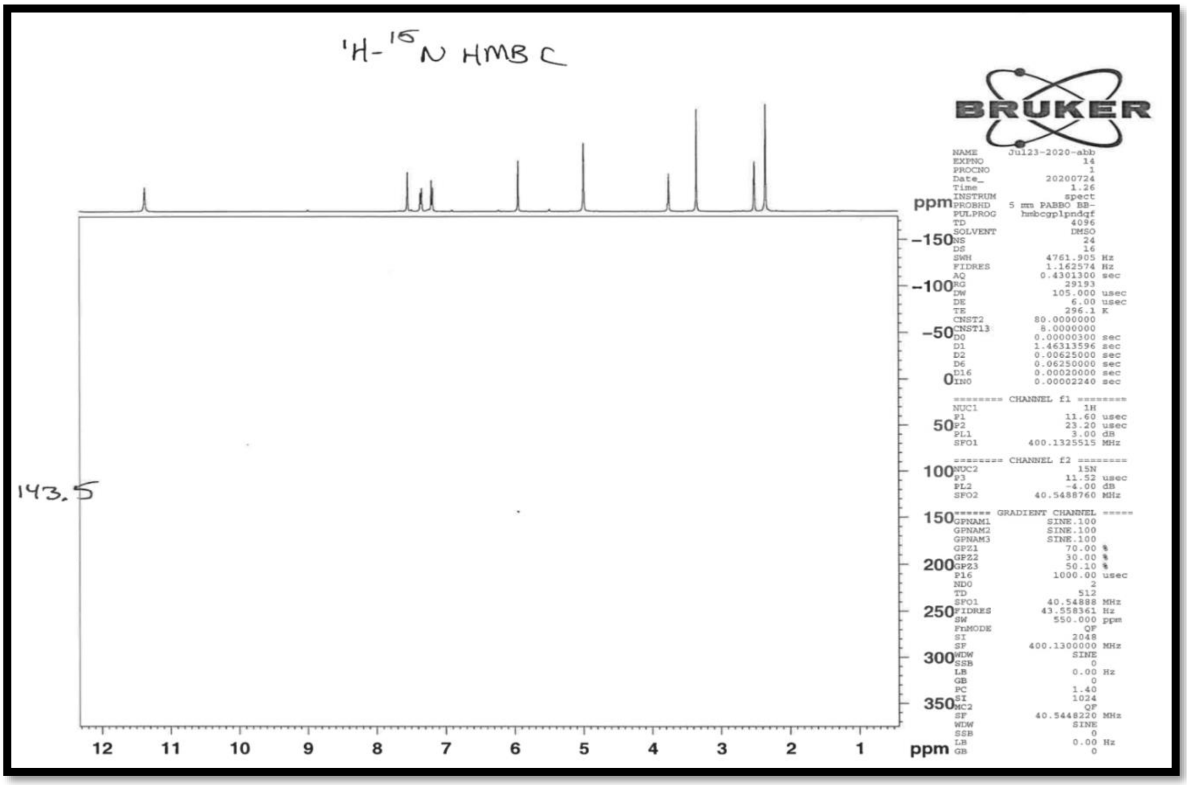

SI Fig. 23. ^{1}H NMR spectrum (DMSO-d_6) of compound **5c**.

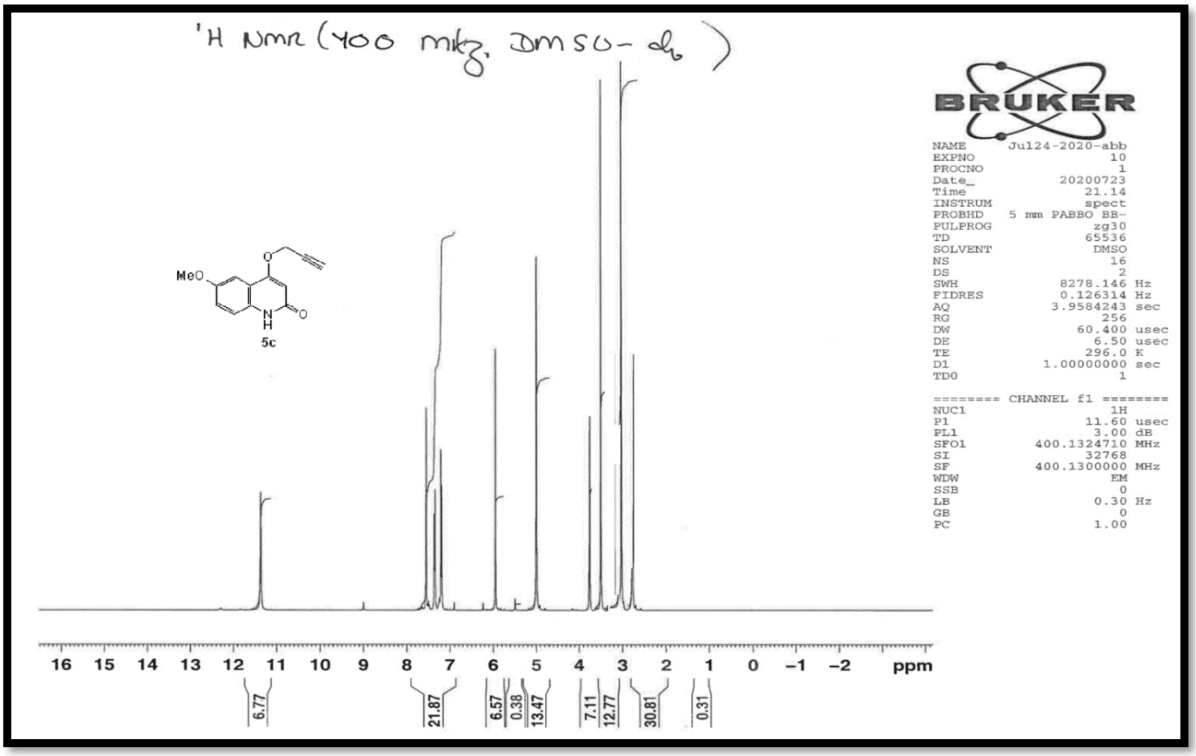

SI Fig. 24. ^{13}C NMR spectrum (DMSO-d_6) of compound **5c**.

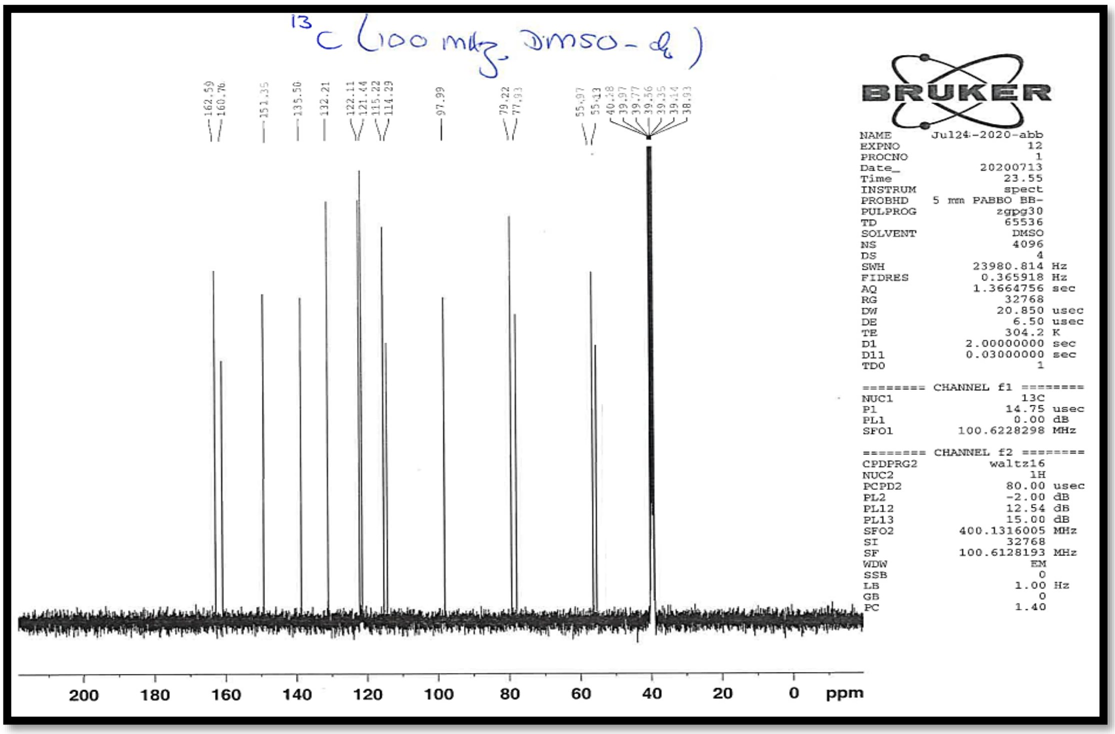

SI Fig. 25. ^{15}N HSQC spectrum (DMSO-d_6) of compound **5c**.

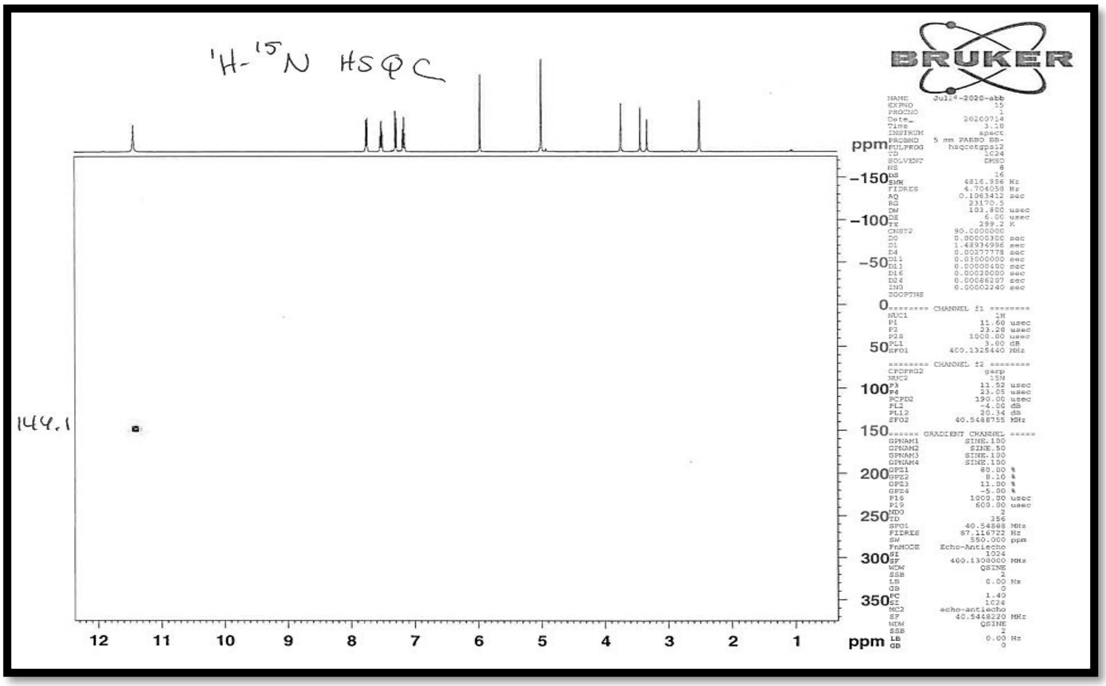

SI Fig. 26. ^{1}H NMR spectrum (DMSO-d_6) of compound **6a**.

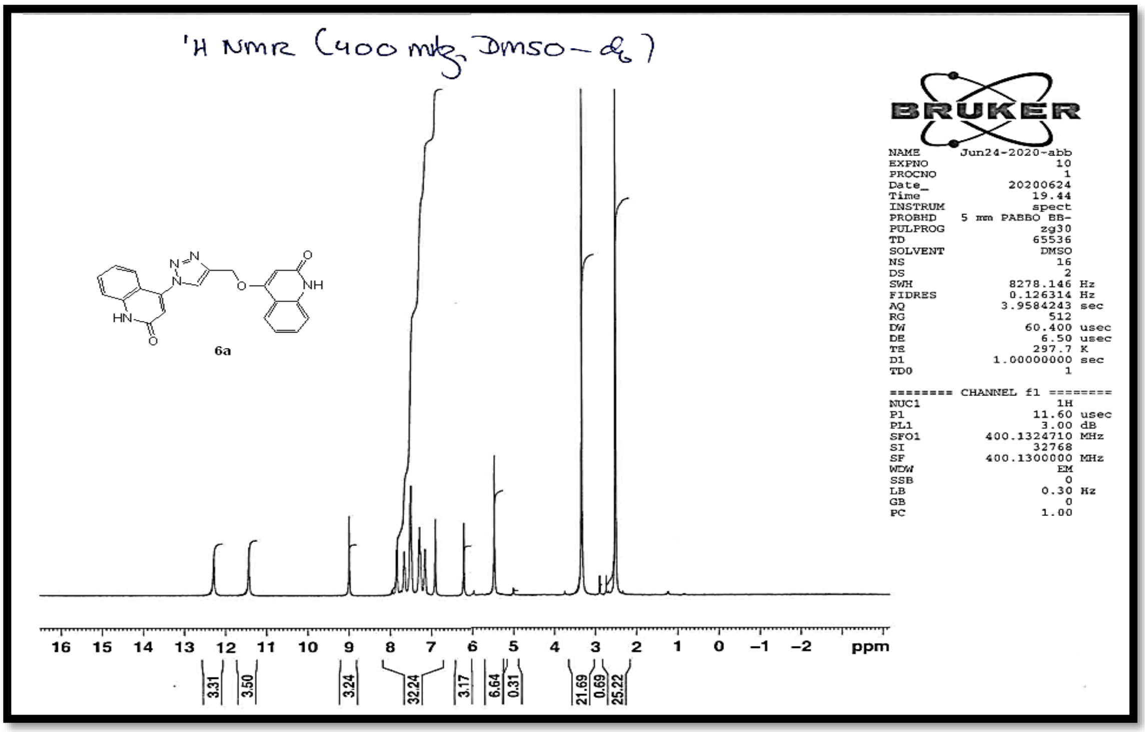

SI Fig. 27. A section of ^{1}H NMR spectrum (DMSO-d_6) of compound **6a**.

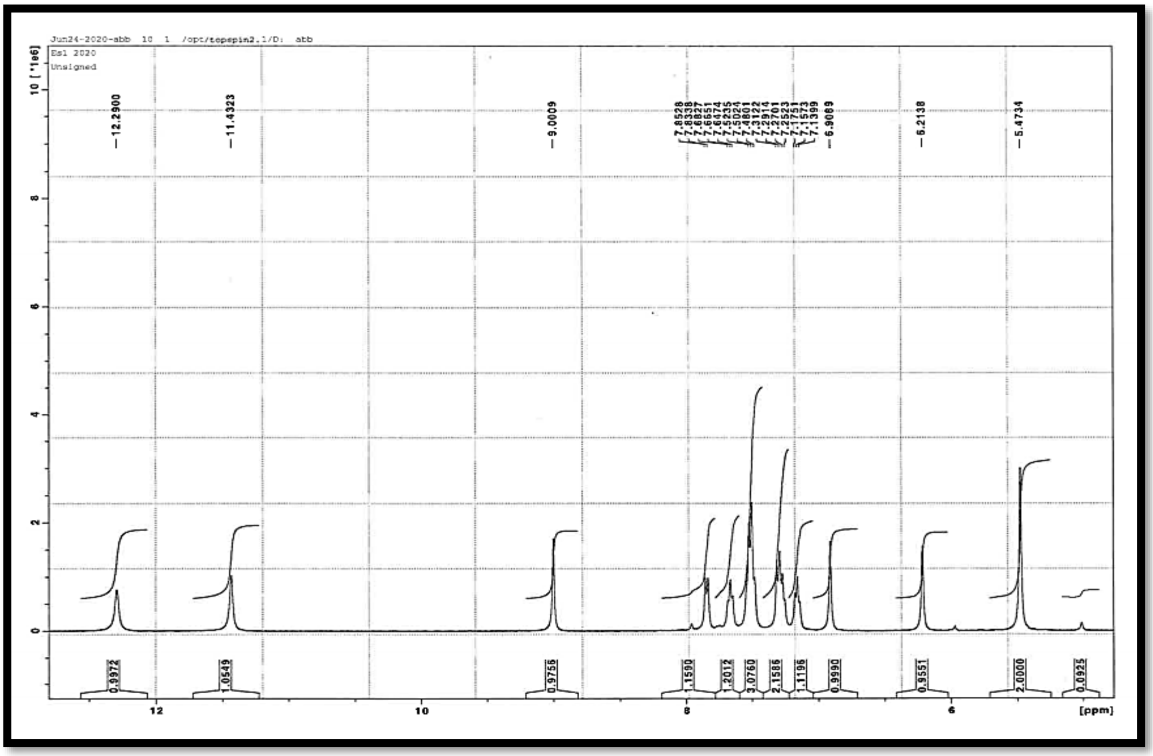

SI Fig. 28. ^{13}C NMR spectrum (DMSO-d_6) of compound **6a**.

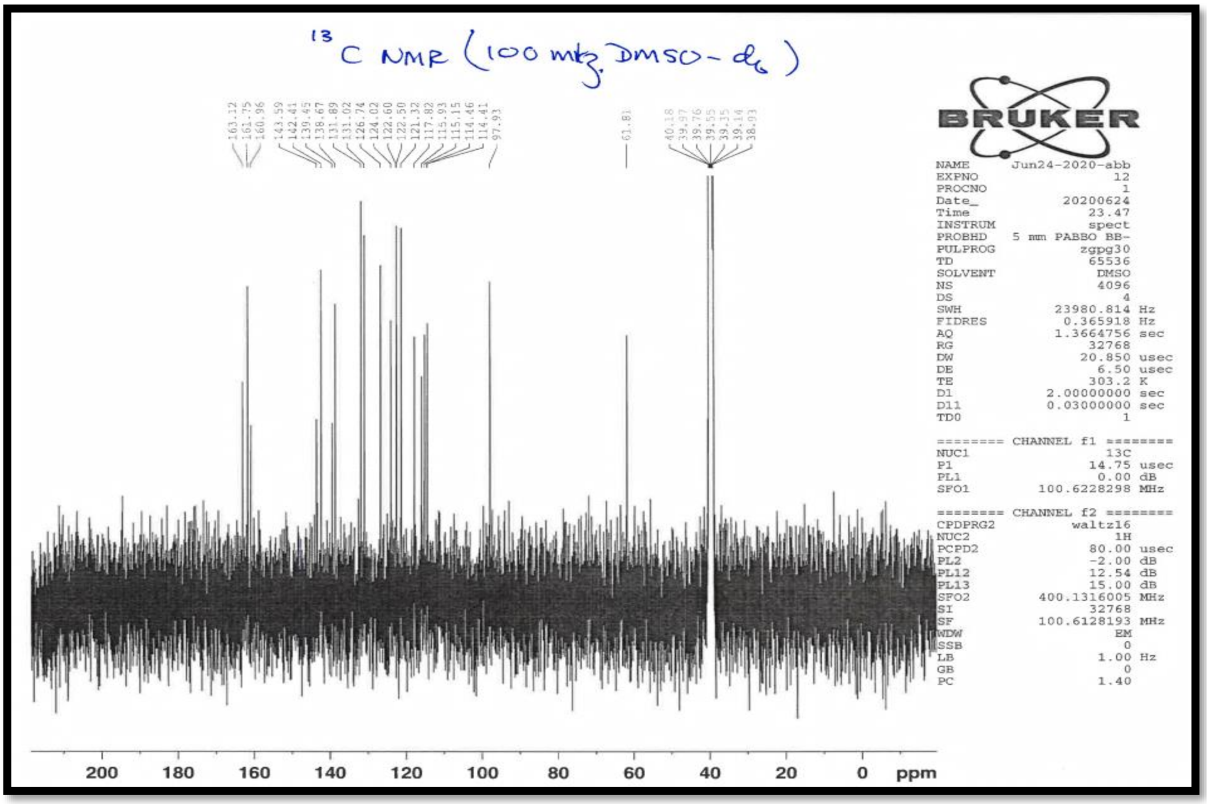

SI Fig. 29. A section of ^{13}C NMR spectrum (DMSO-d_6) of compound **6a**.

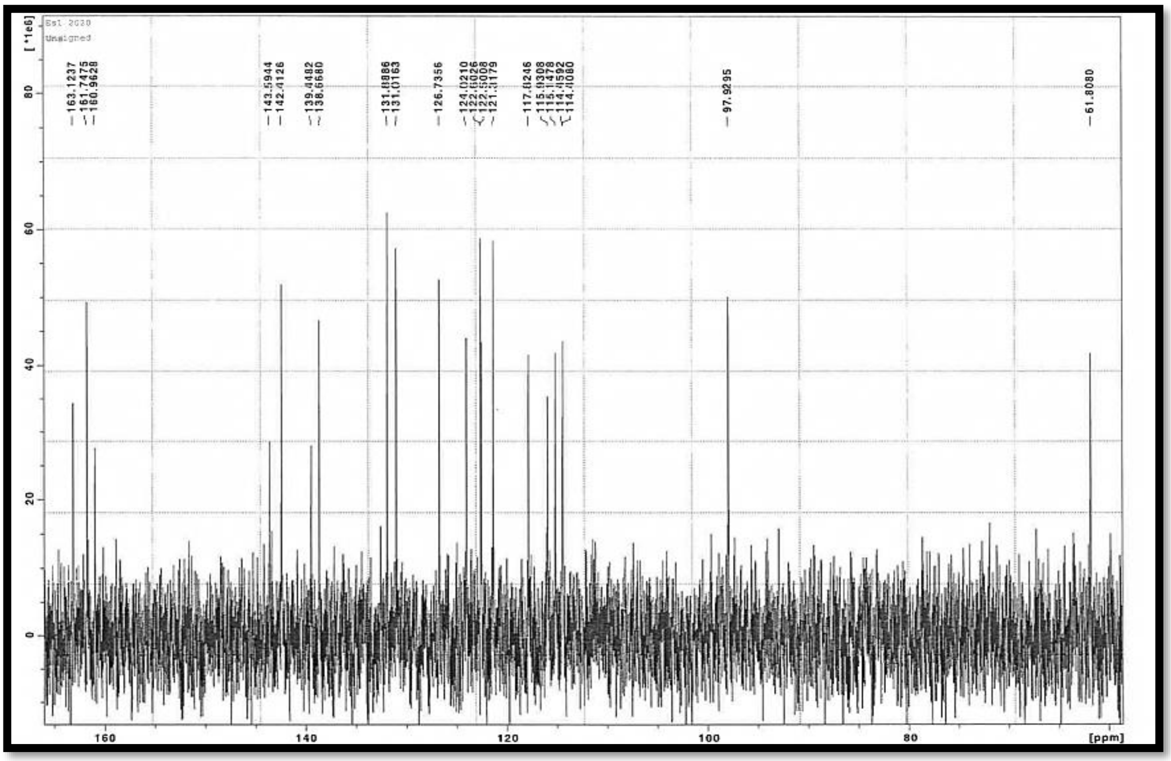

SI Fig. 30. ^{1}H ^{1}H Cosy spectrum (DMSO-d_6) of compound **6a**.

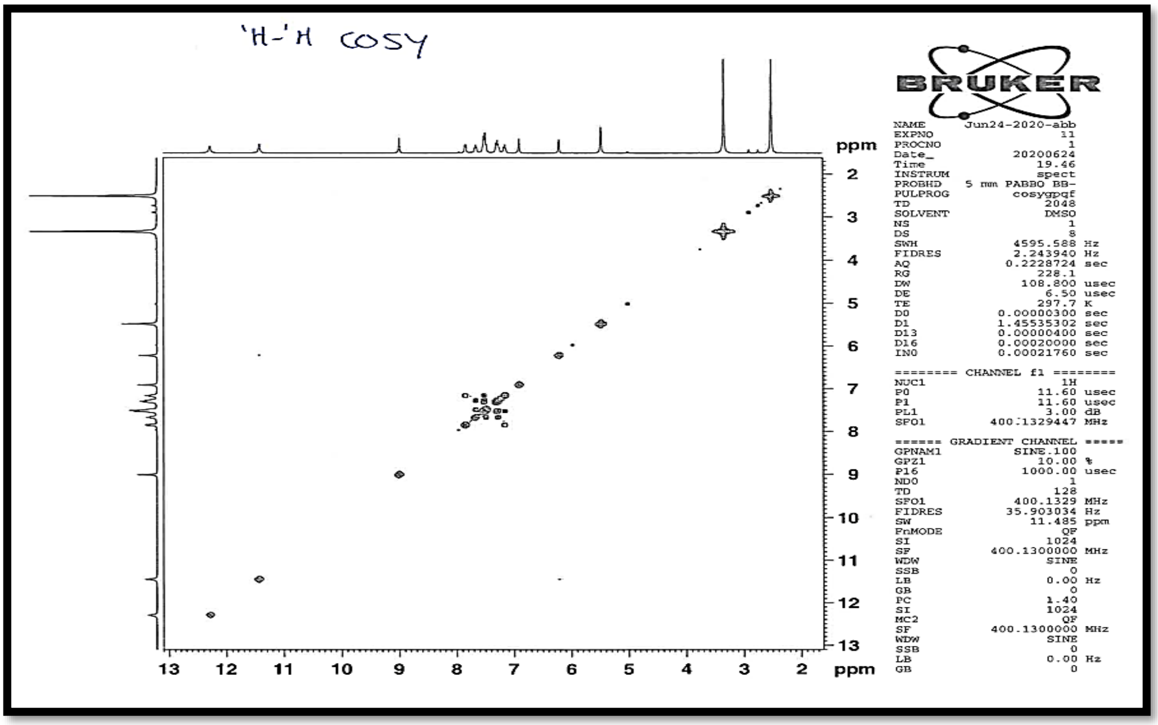

SI Fig. 31. ^{1}H ^{13}C HSQC spectrum (DMSO-d_6) of compound **6a**.

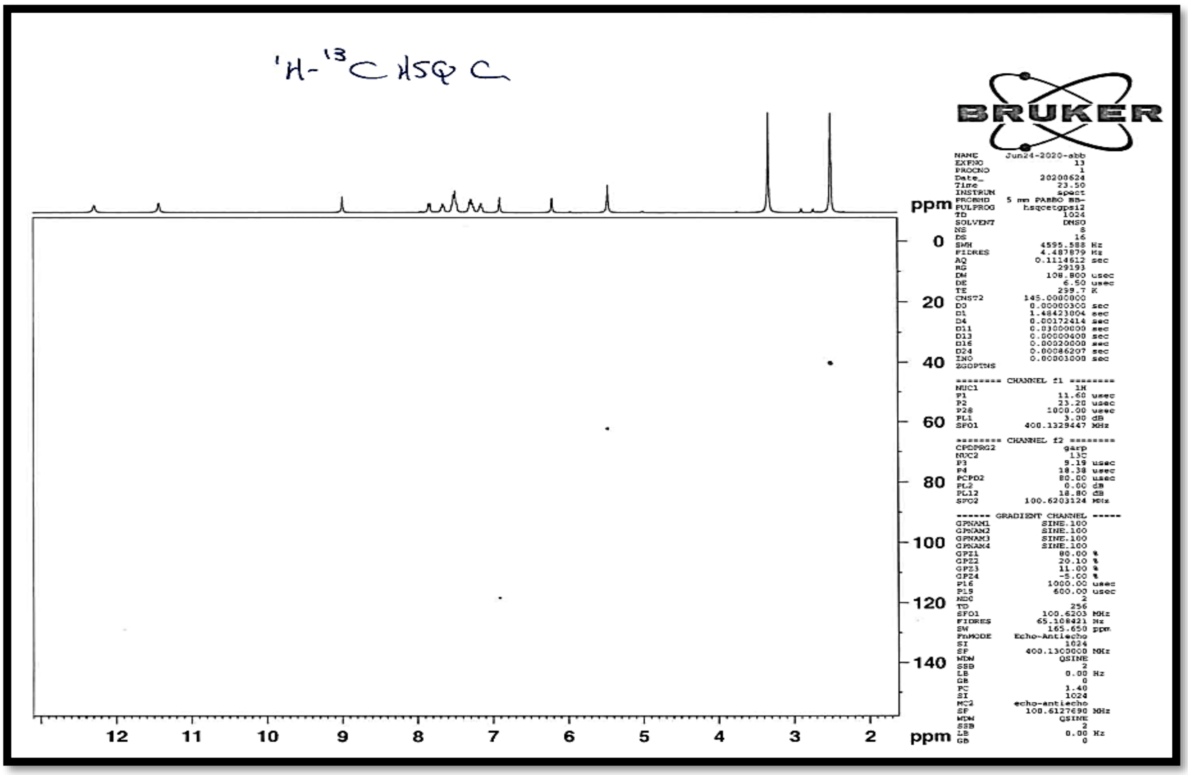

SI Fig. 32. A section of ^{1}H ^{13}C HSQC spectrum (DMSO-d_6) of compound **6a**.

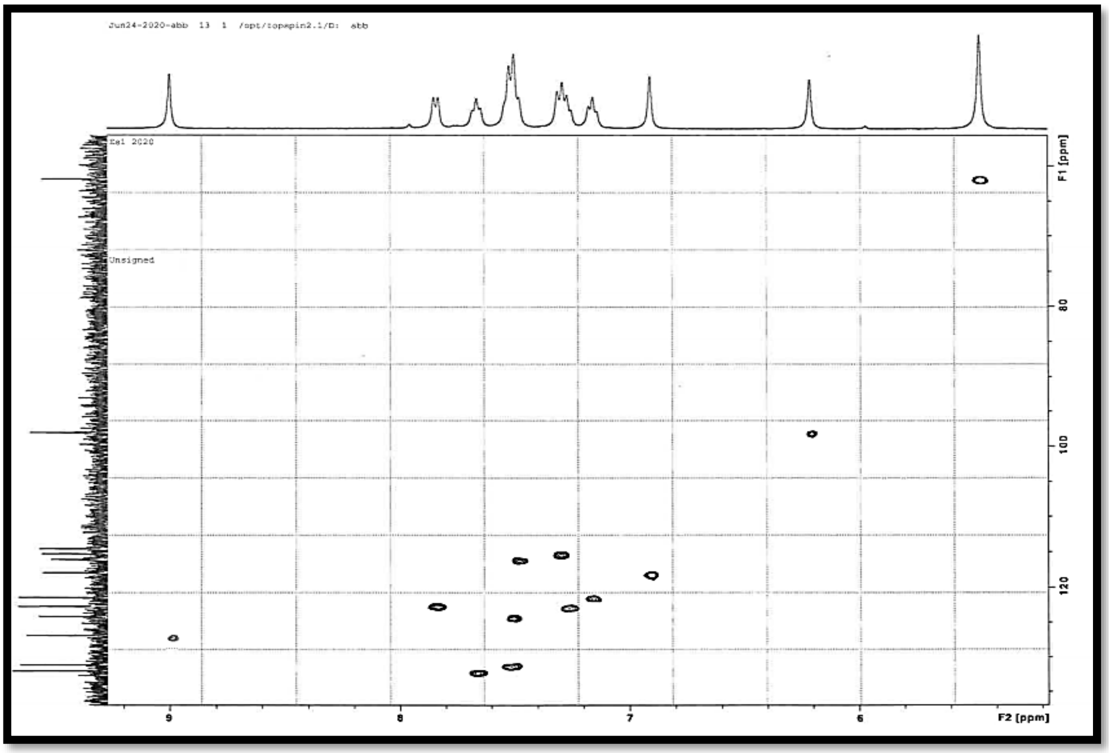

SI Fig. 33. ^{1}H ^{13}C HMBC spectrum (DMSO-d_6) of compound **6a**.

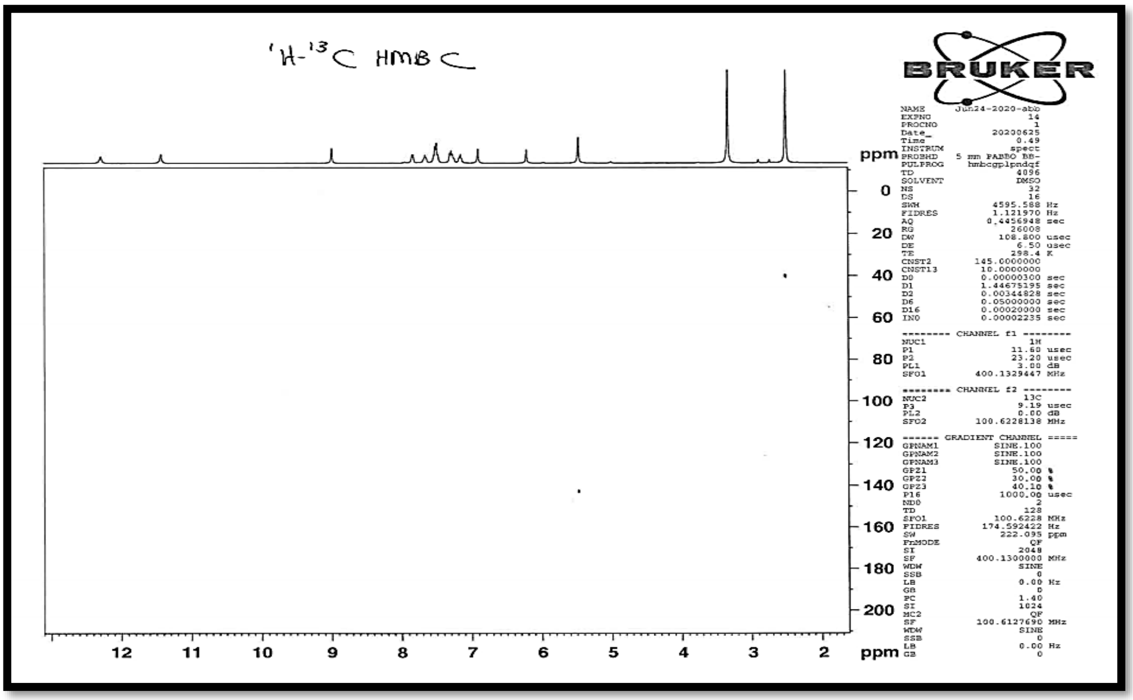

SI Fig. 34. A section of ^{1}H ^{13}C HMBC spectrum (DMSO-d_6) of compound **6a**.

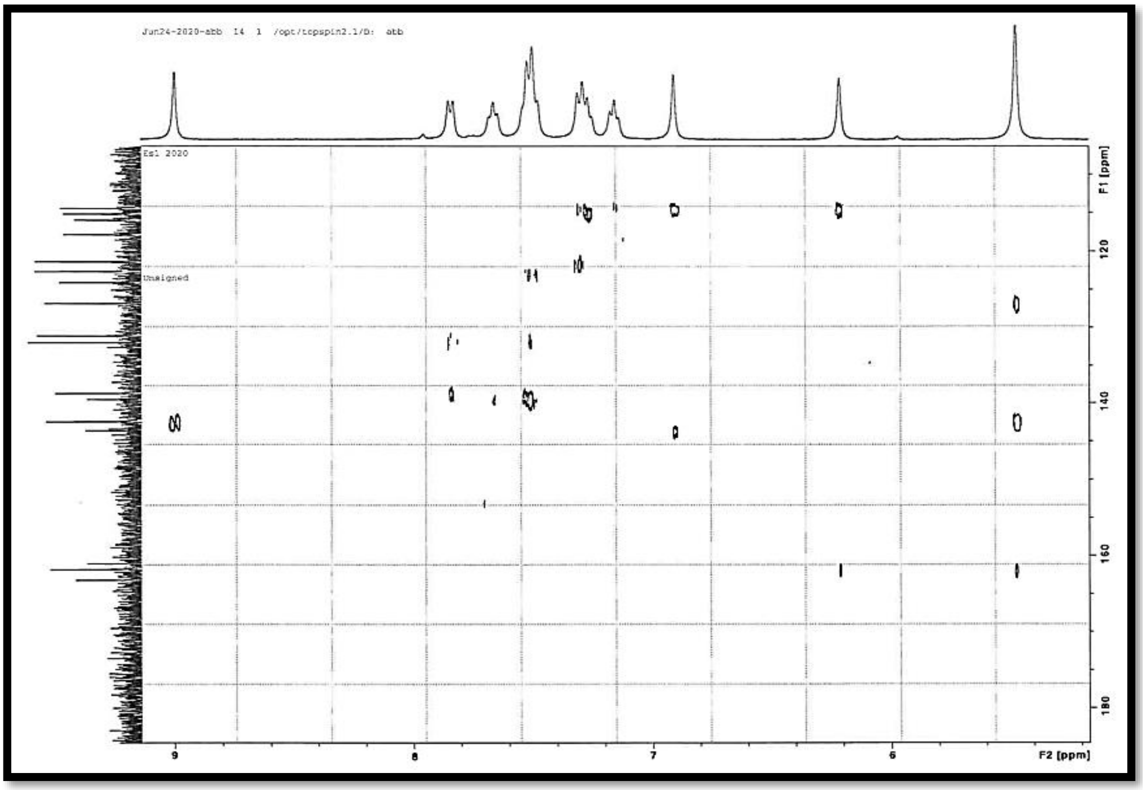

SI Fig. 35. ^{1}H ^{15}N HMBC spectrum (DMSO-d_6) of compound **6a**.

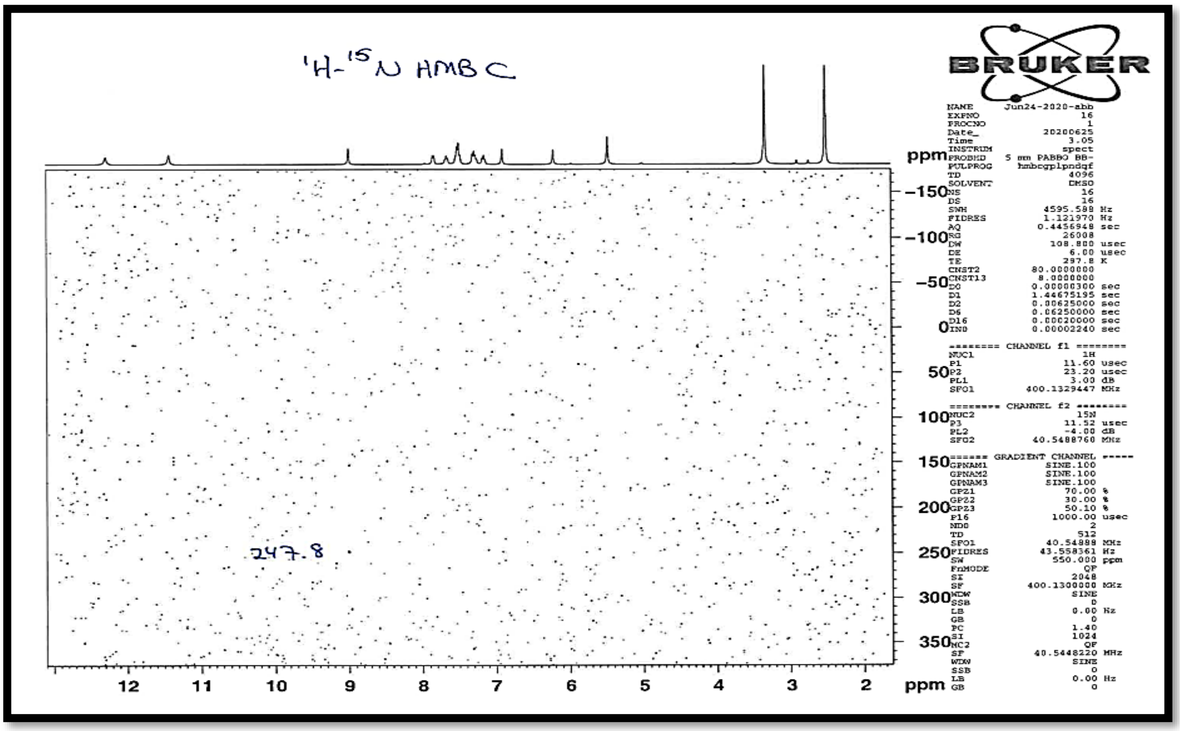

SI Fig. 36. A section of ^{1}H ^{15}N HMBC spectrum (DMSO-d_6) of compound **6a**.

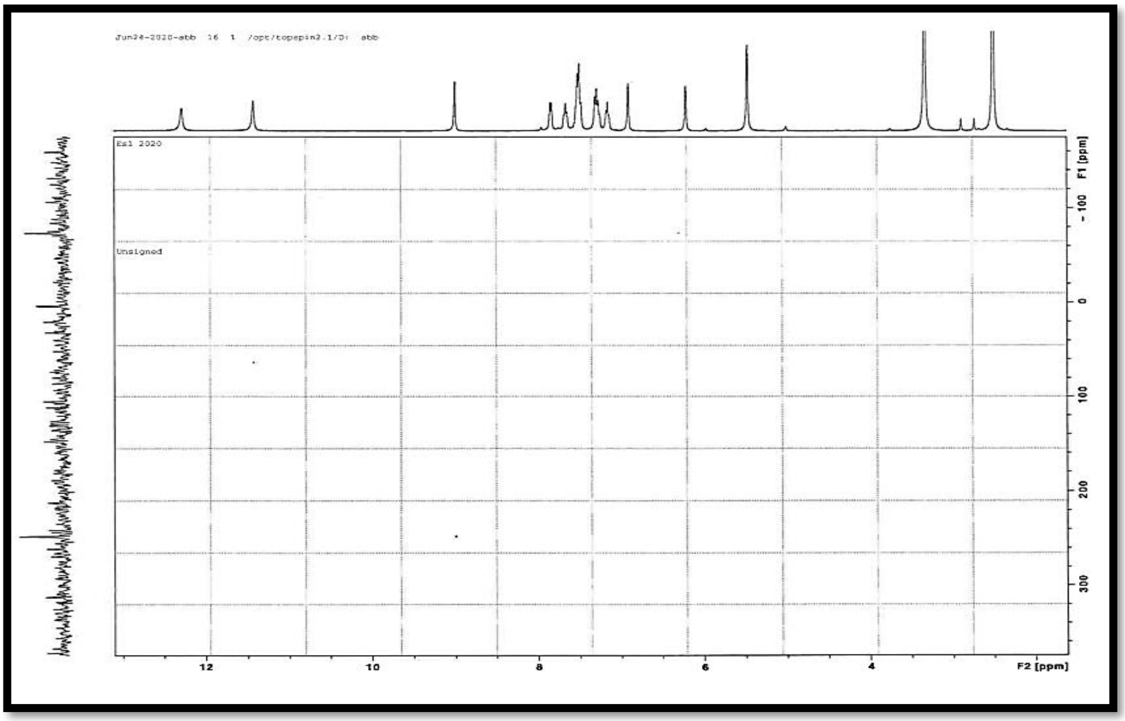

SI Fig. 37. The mass spectrum for compound **6a**.

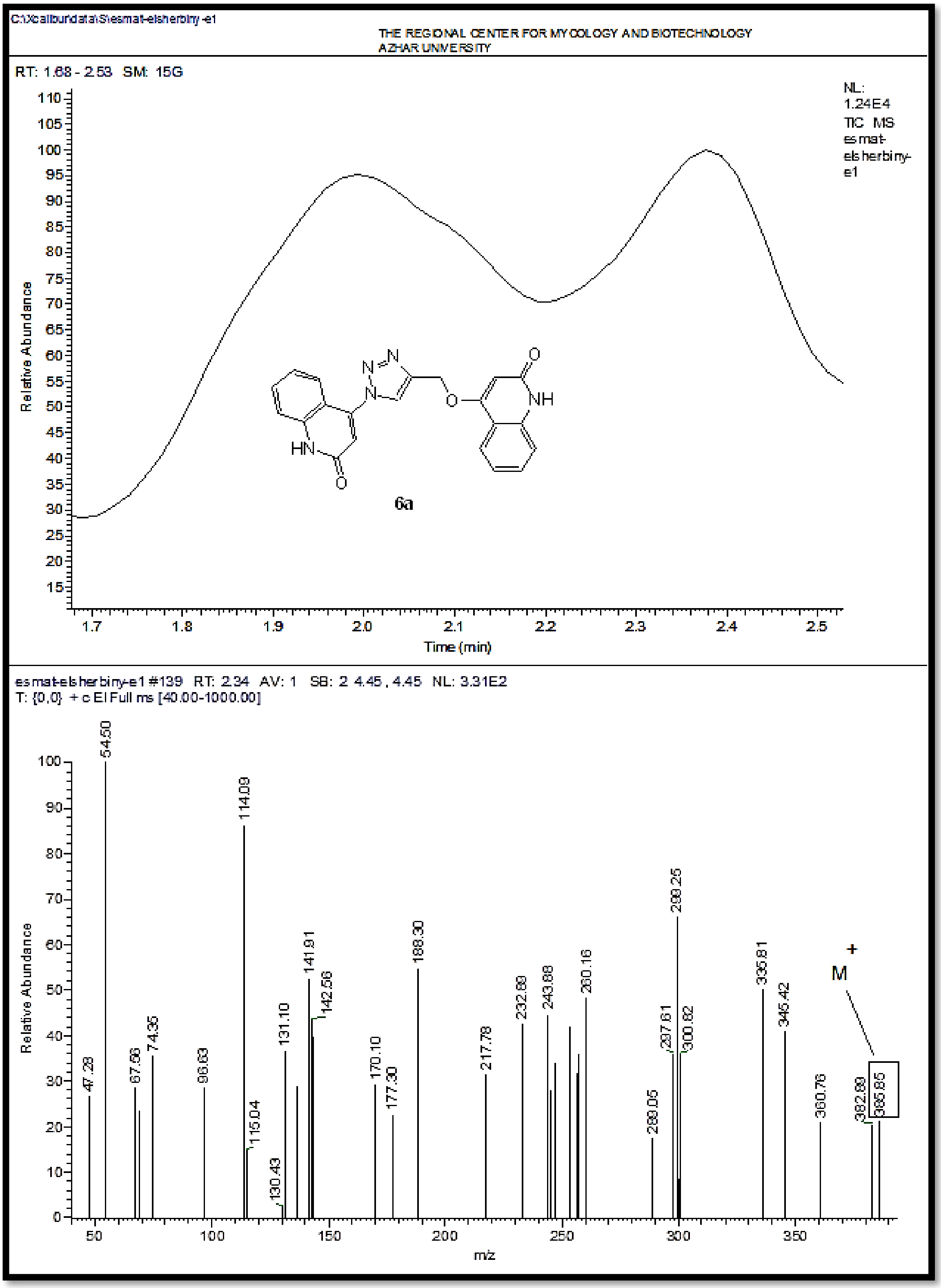

SI Fig. 38. ^{1}H NMR spectrum (DMSO-d_6) of compound **6b**.

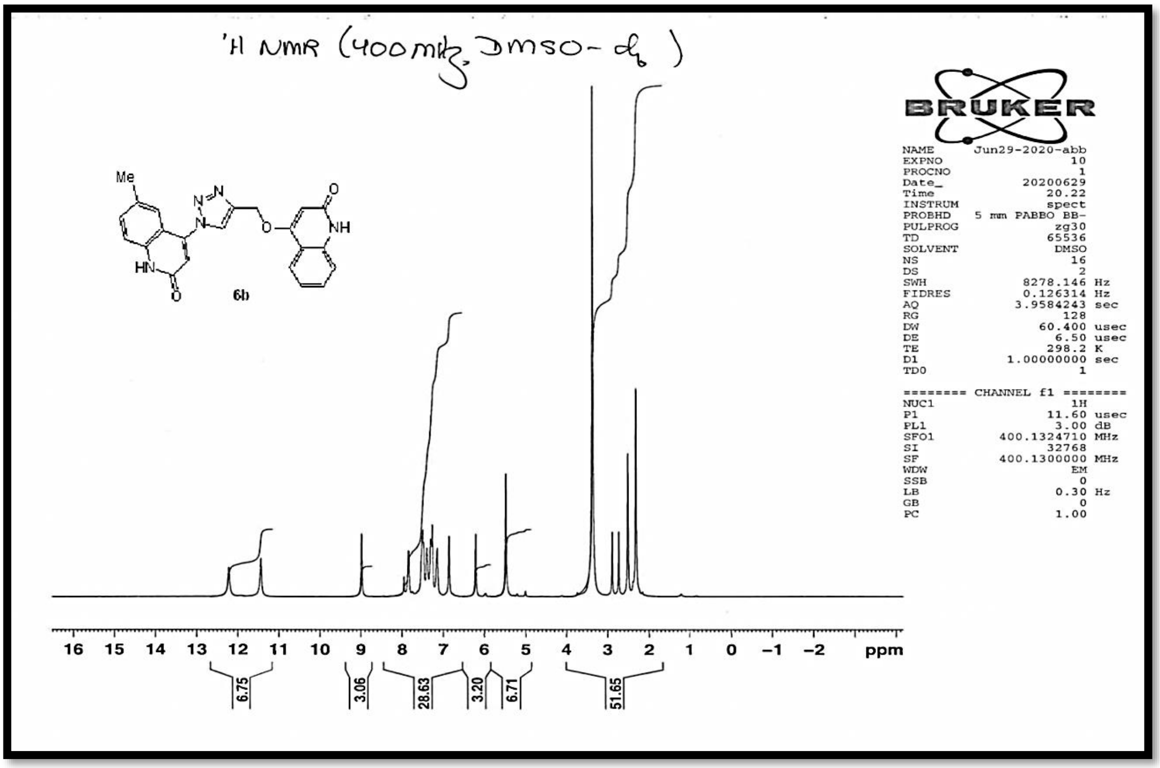

SI Fig. 39. A section of ^{1}H NMR spectrum (DMSO-d_6) of compound **6b**.

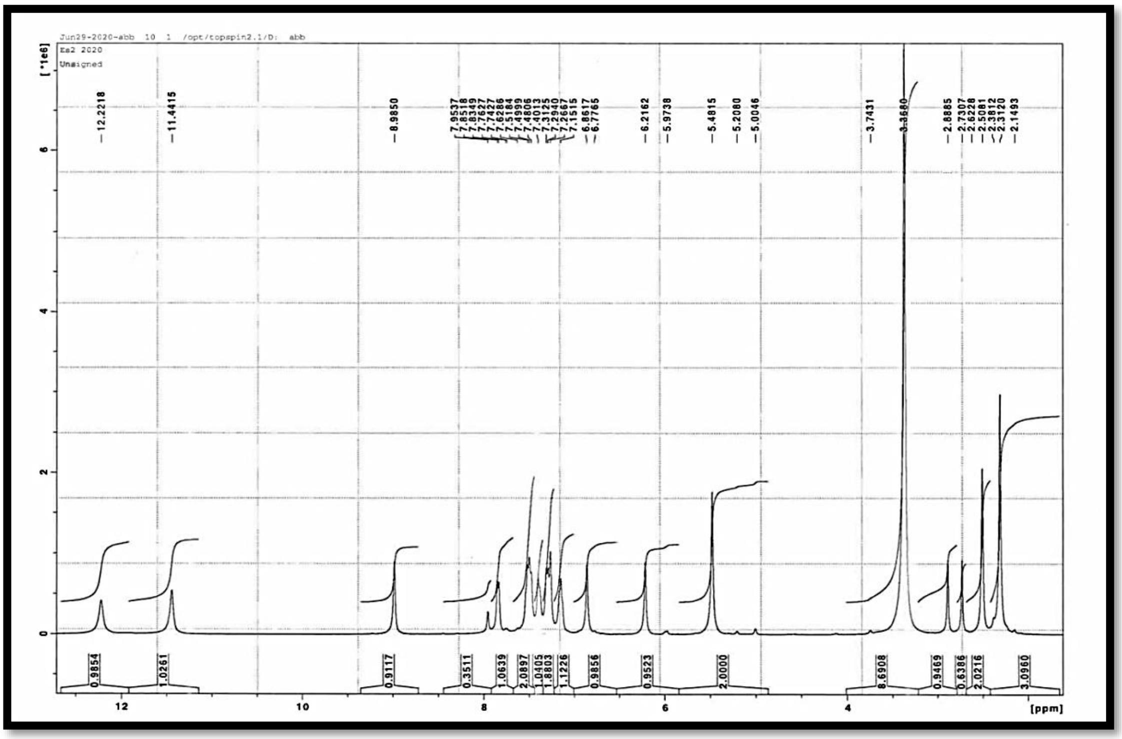

SI Fig. 40. ^{13}C NMR spectrum (DMSO-d_6) of compound **6b**.

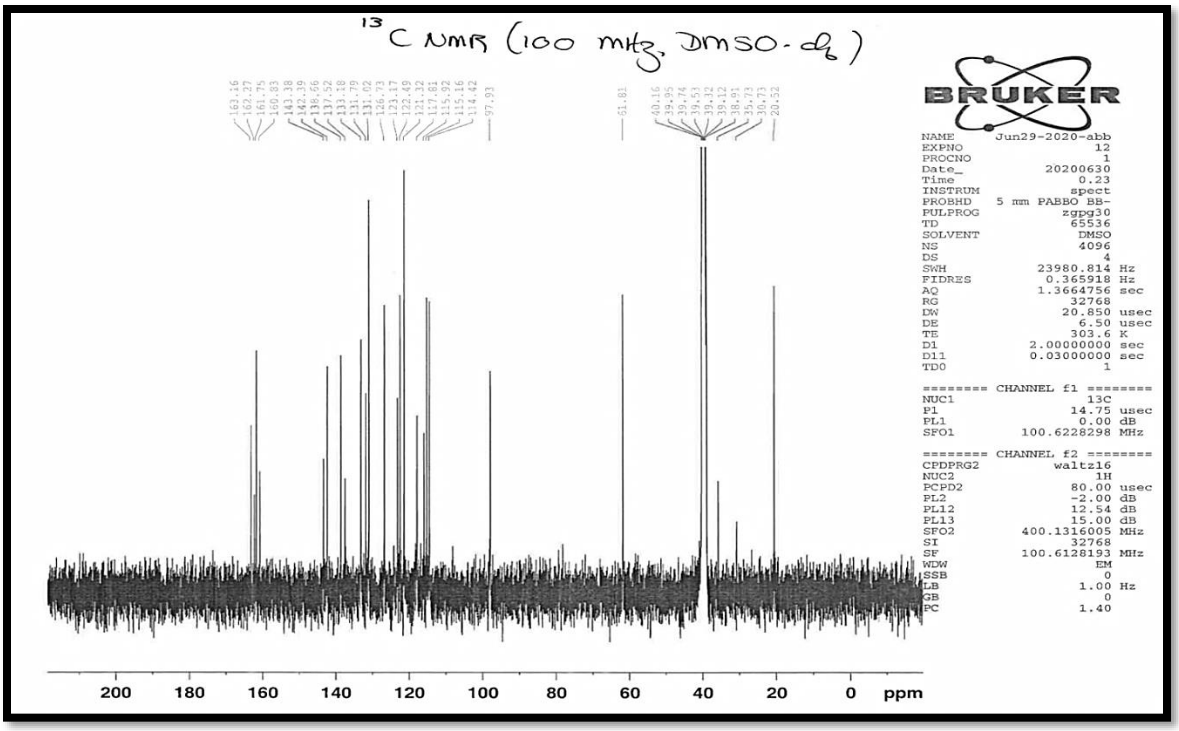

SI Fig. 41. ^{1}H ^{1}H Cosy spectrum (DMSO-d_6) of compound **6b**.

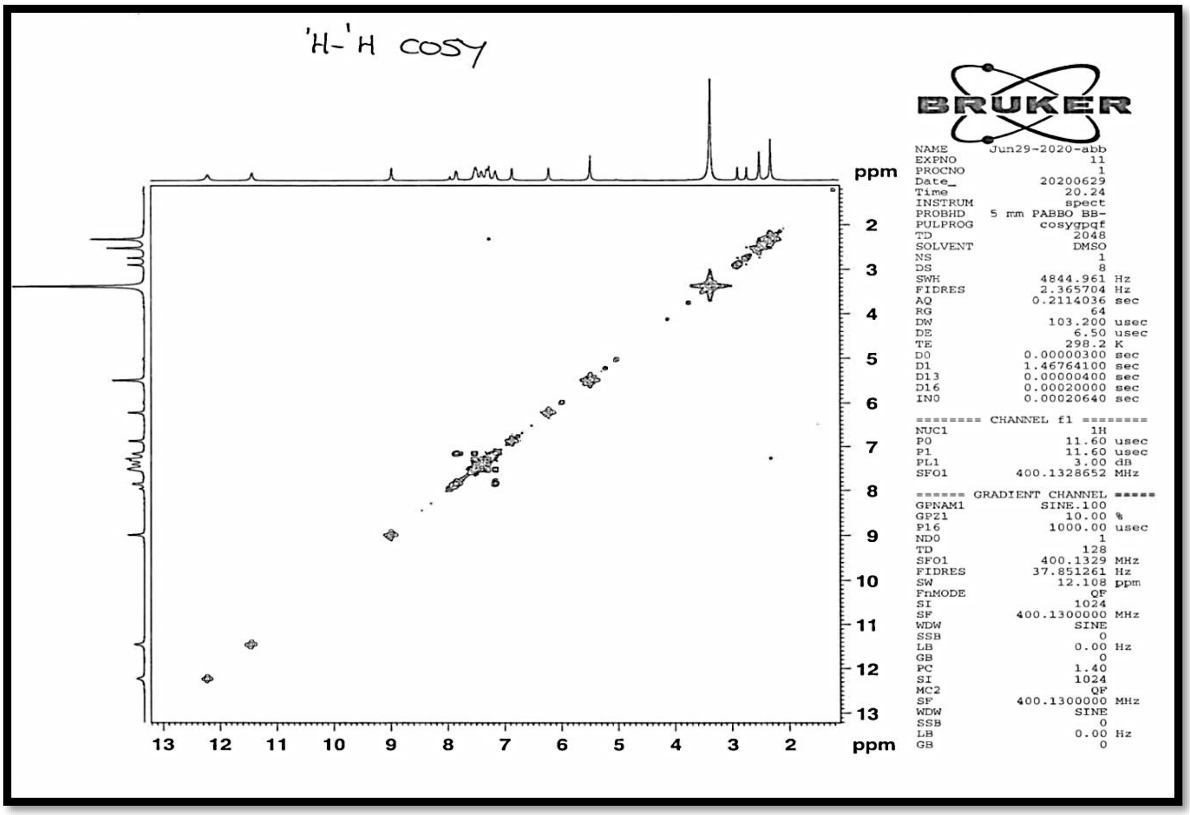

SI Fig. 42. A section of ^{1}H ^{1}H Cosy spectrum (DMSO-d_6) of compound **6b**.

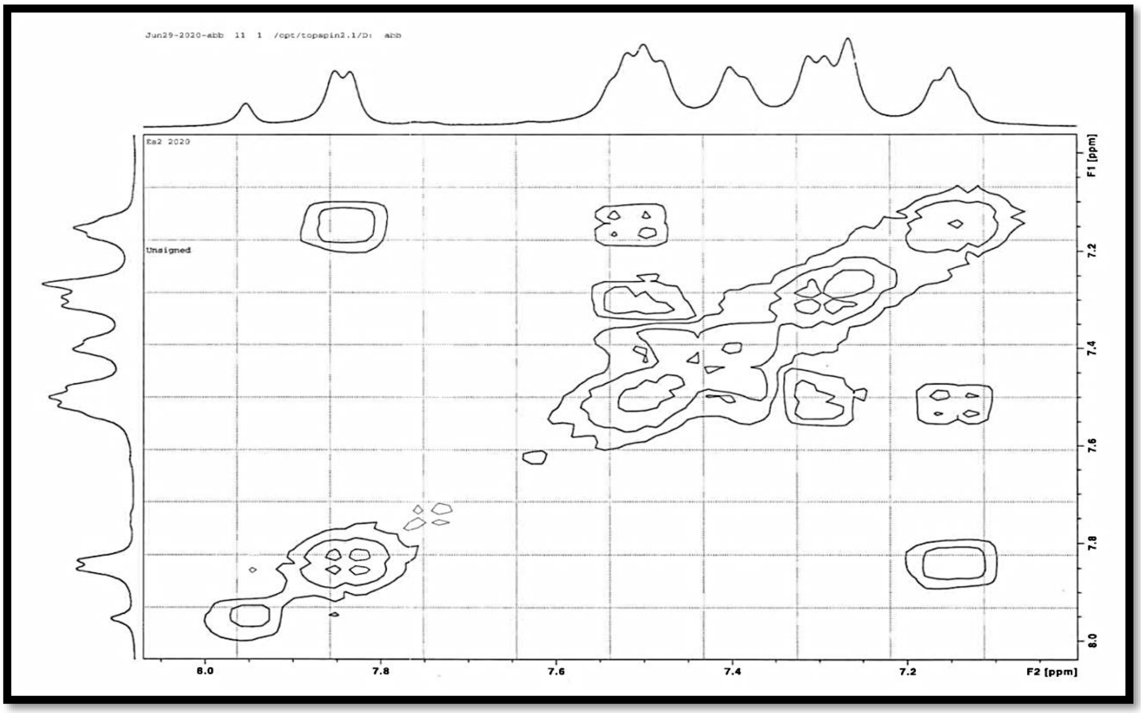

SI Fig. 43. ^{1}H ^{13}C HSQC spectrum (DMSO-d_6) of compound **6b**.

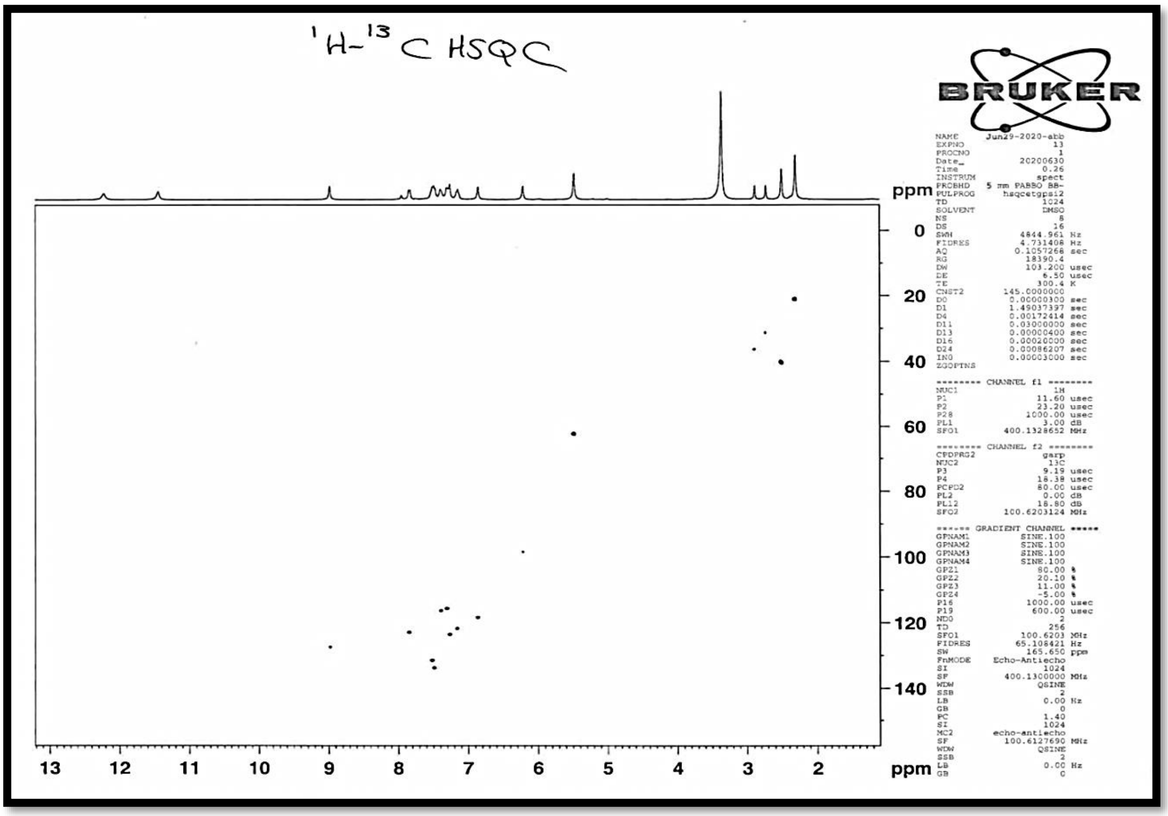

SI Fig. 44. ^{1}H ^{13}C HMBC spectrum (DMSO-d_6) of compound **6b**.

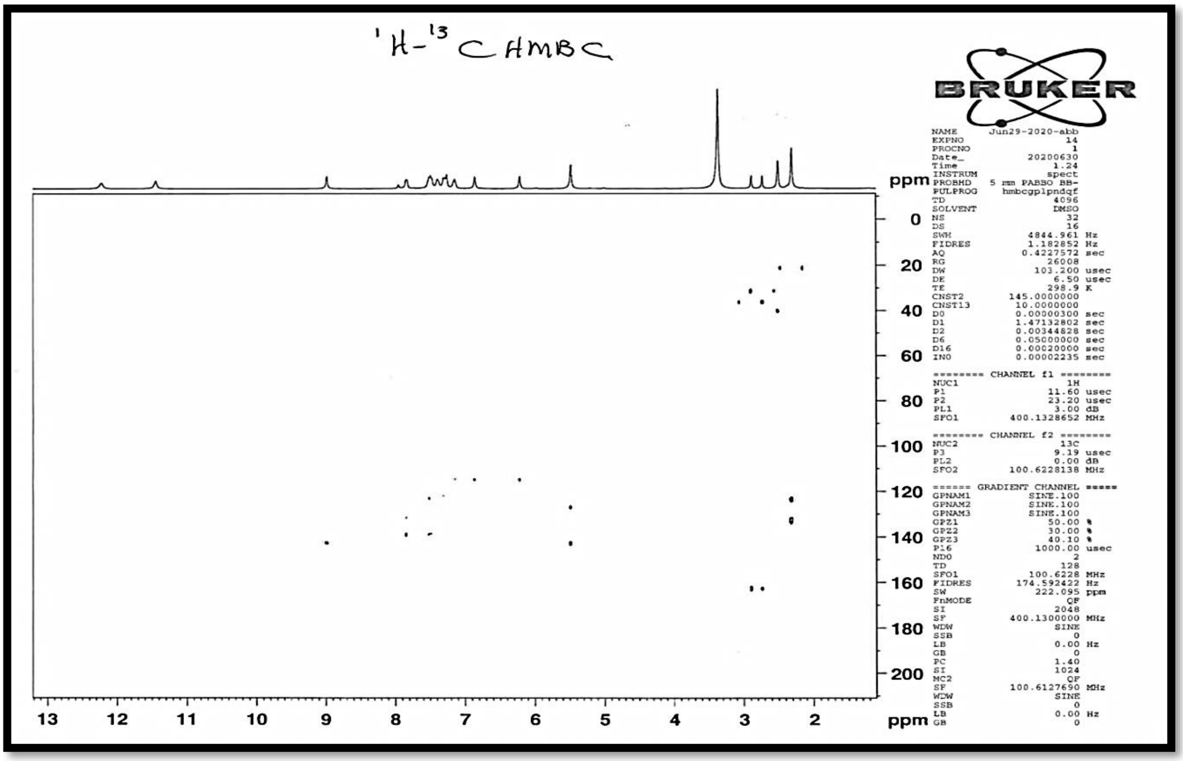

SI Fig. 45. A section of ^{1}H ^{13}C HMBC spectrum (DMSO-d_6) of compound **6b**.

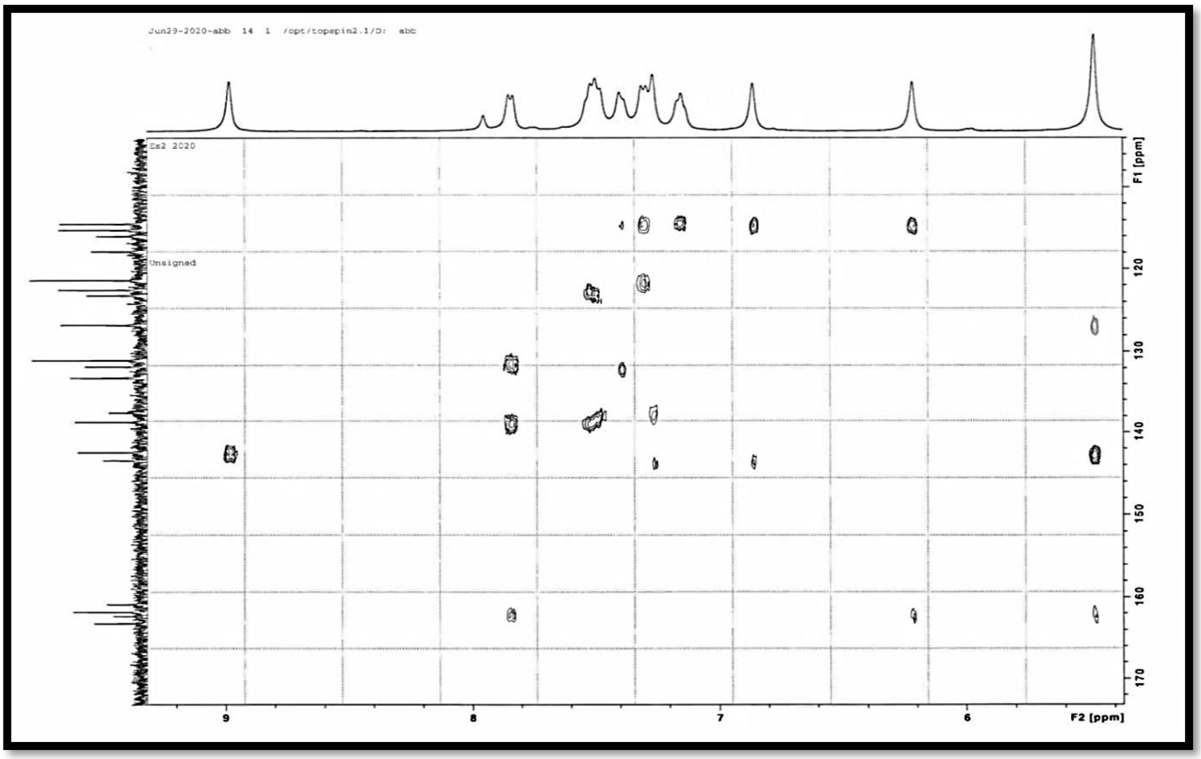

SI Fig. 46. ^{1}H ^{15}N HSQC spectrum (DMSO-d_6) of compound **6b**.

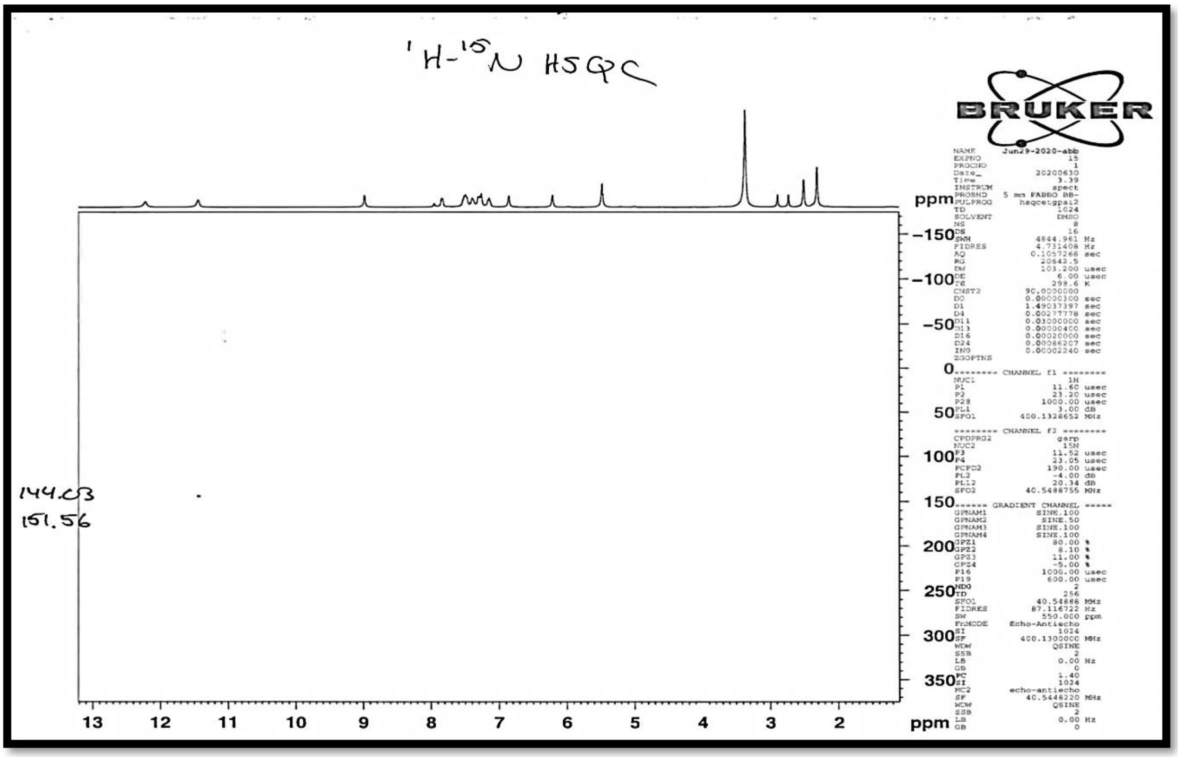

SI Fig. 47. ^{1}H ^{15}N HMBC spectrum (DMSO-d_6) of compound **6b**.

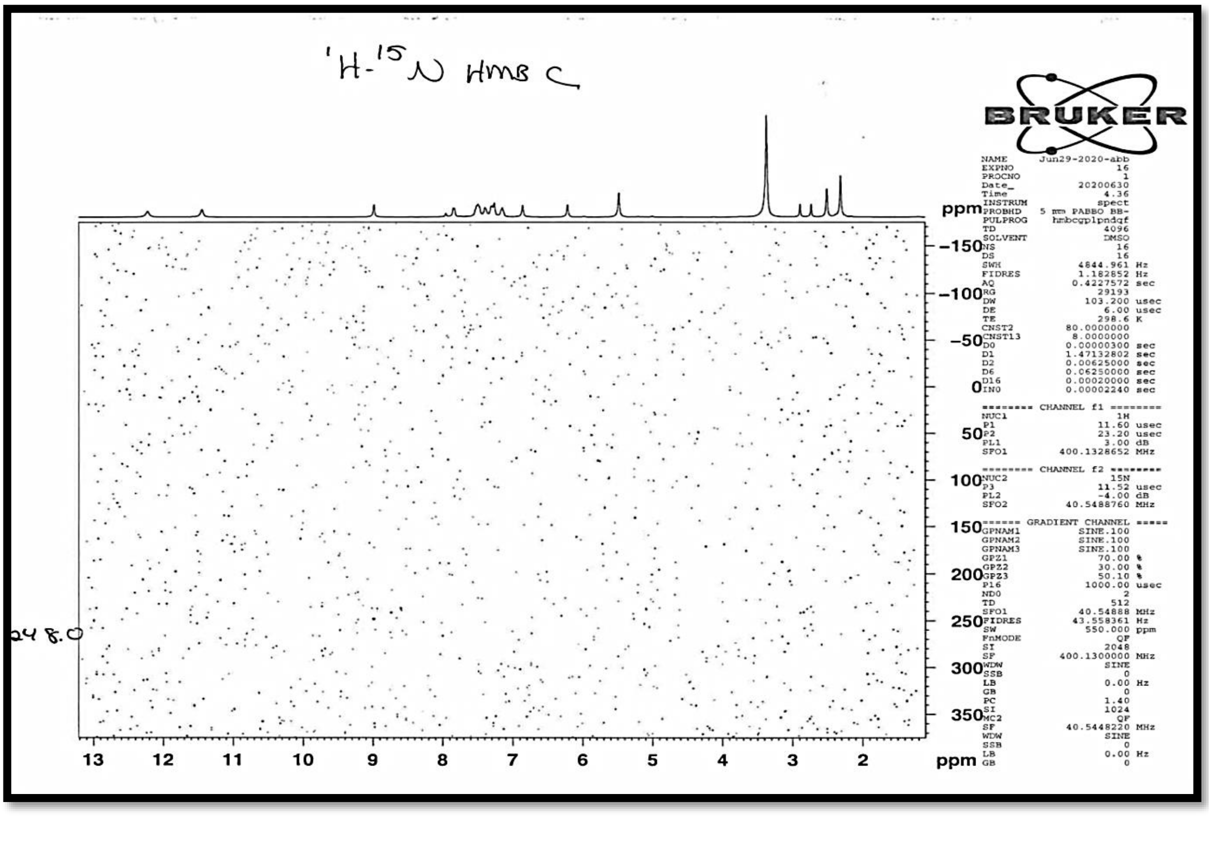

SI Fig. 48. The mass spectrum of compound **6b**.

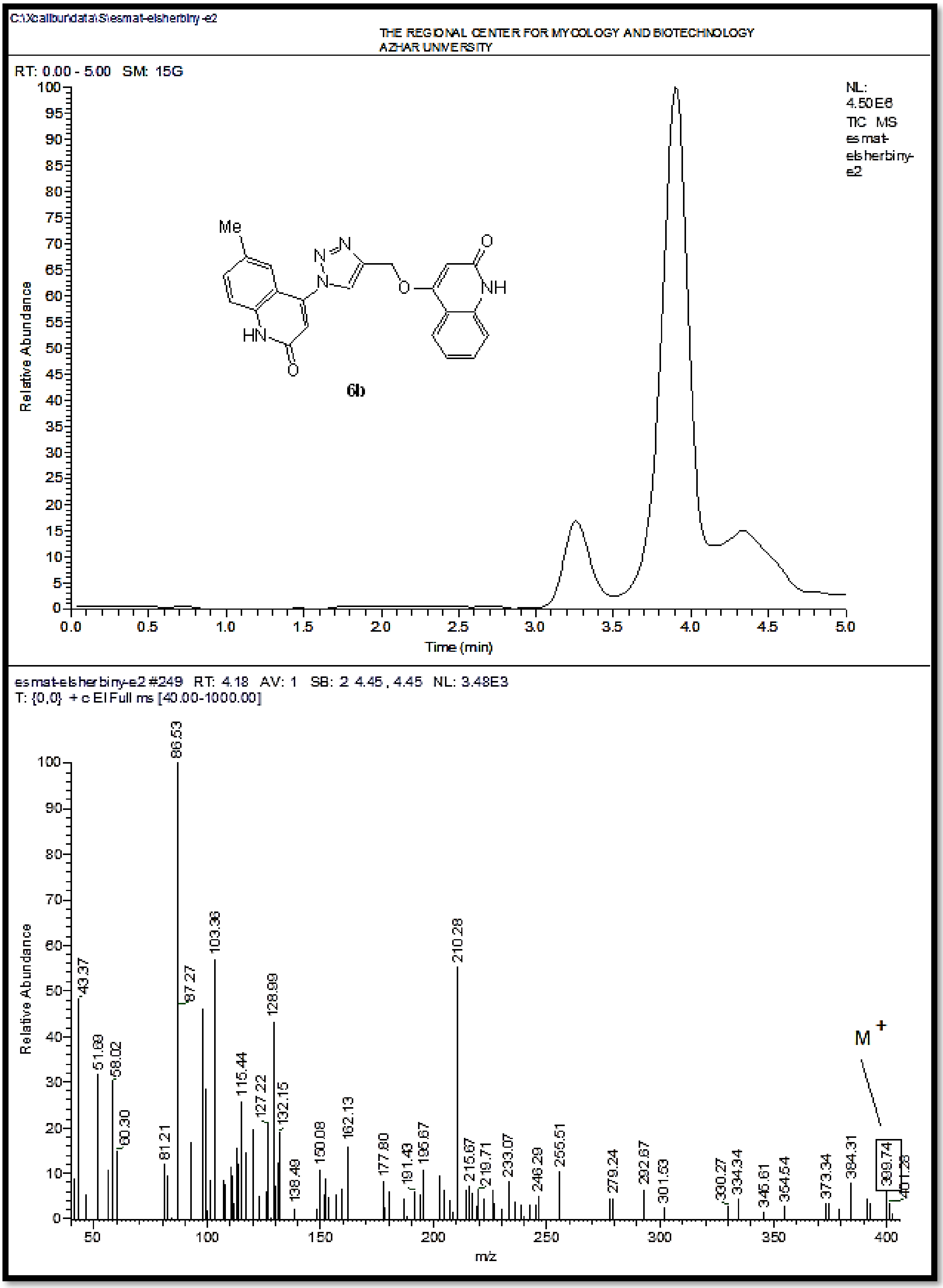

SI Fig. 49. ^{1}H NMR spectrum (DMSO-d_6) of compound **6c**.

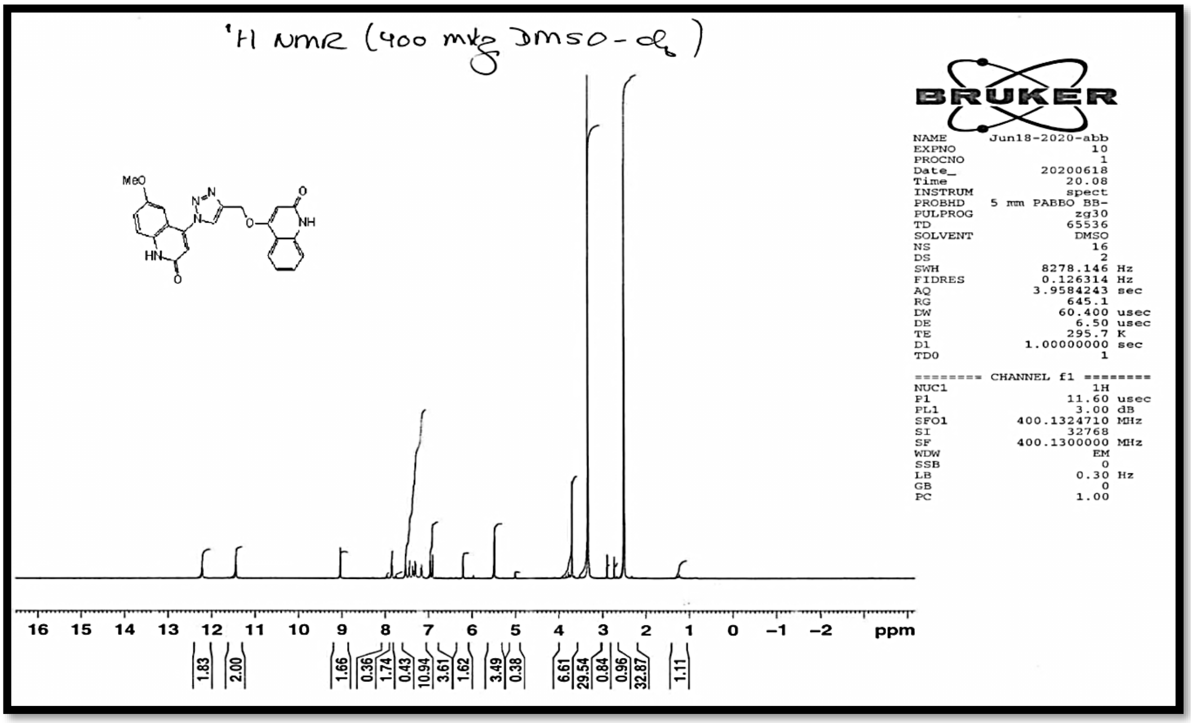

SI Fig. 50. A section of ^{1}H NMR spectrum (DMSO-d_6) of compound **6c**.

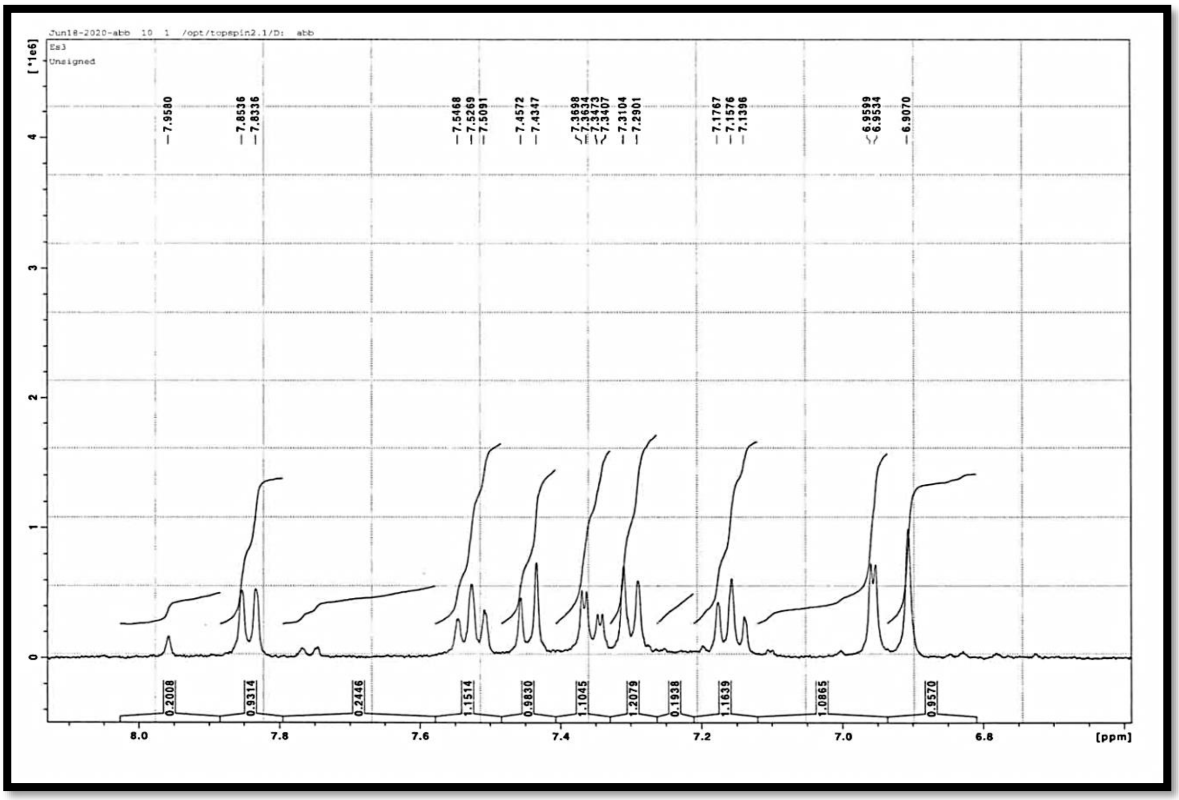

SI Fig. 51. ^{13}C NMR spectrum (DMSO-d_6) of compound **6c**.

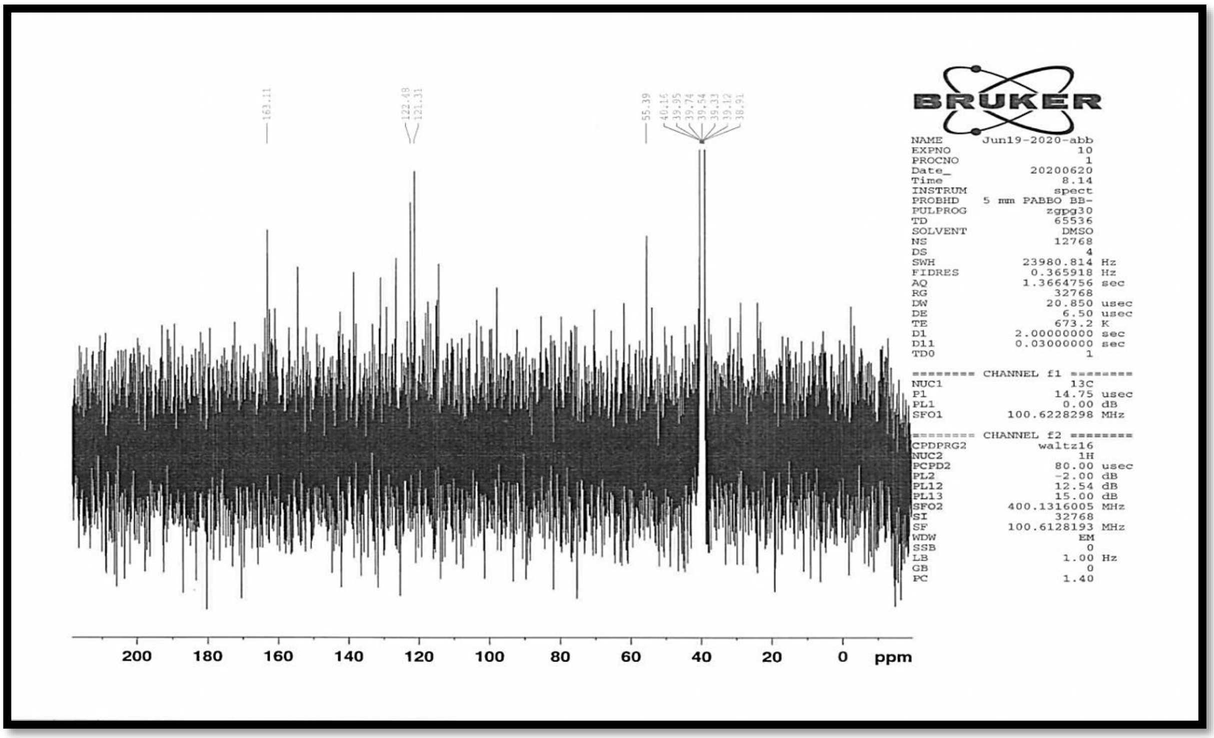

SI Fig. 52. ^{1}H ^{1}H Cosy spectrum (DMSO-d_6) of compound **6c**.

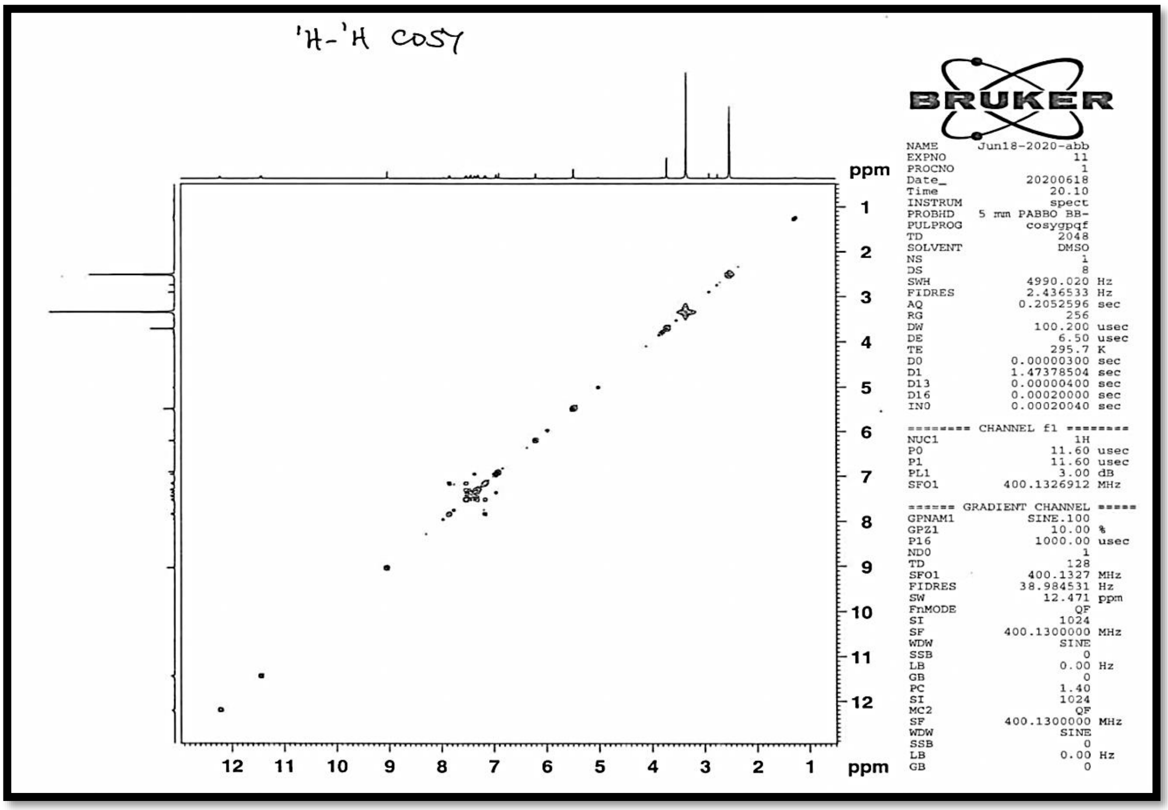

SI Fig. 53. A section of $^{1}H\,^{1}H$ Cosy spectrum (DMSO-d_6) of compound **6c**.

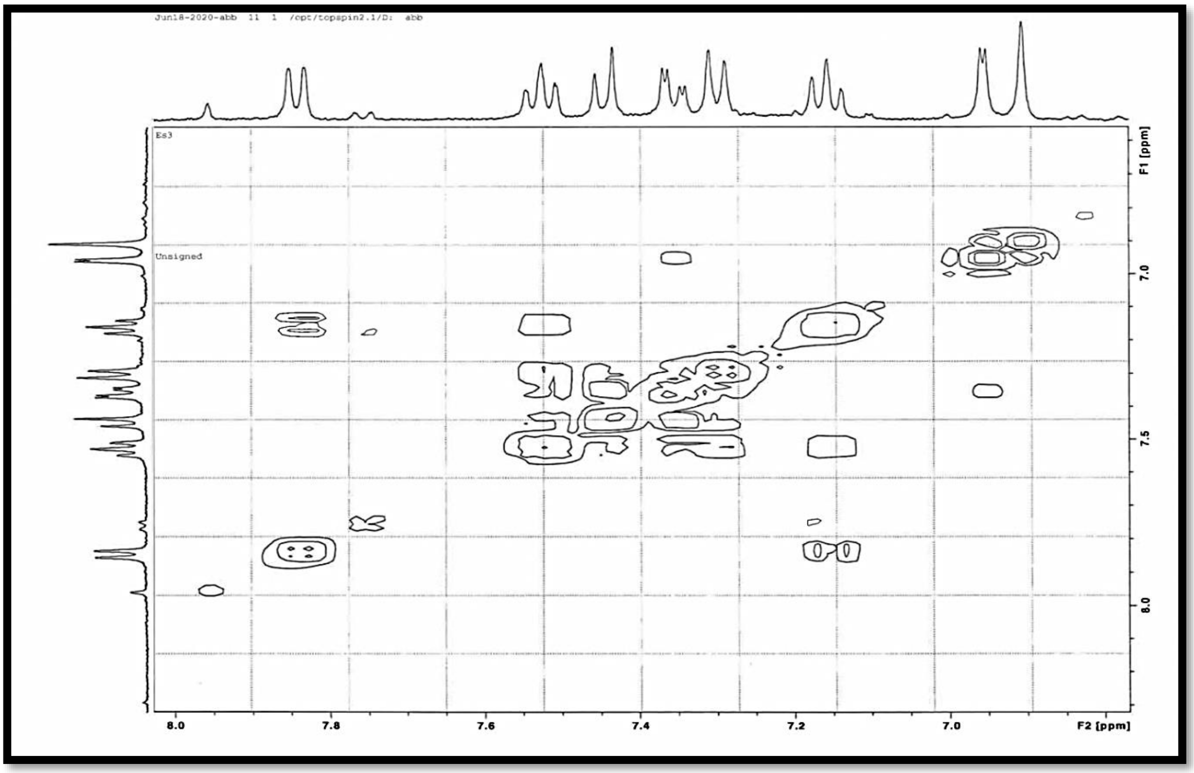

SI Fig. 54. A section of $^{1}H\,^{13}C$ HSQC spectrum (DMSO-d_6) of compound **6c**.

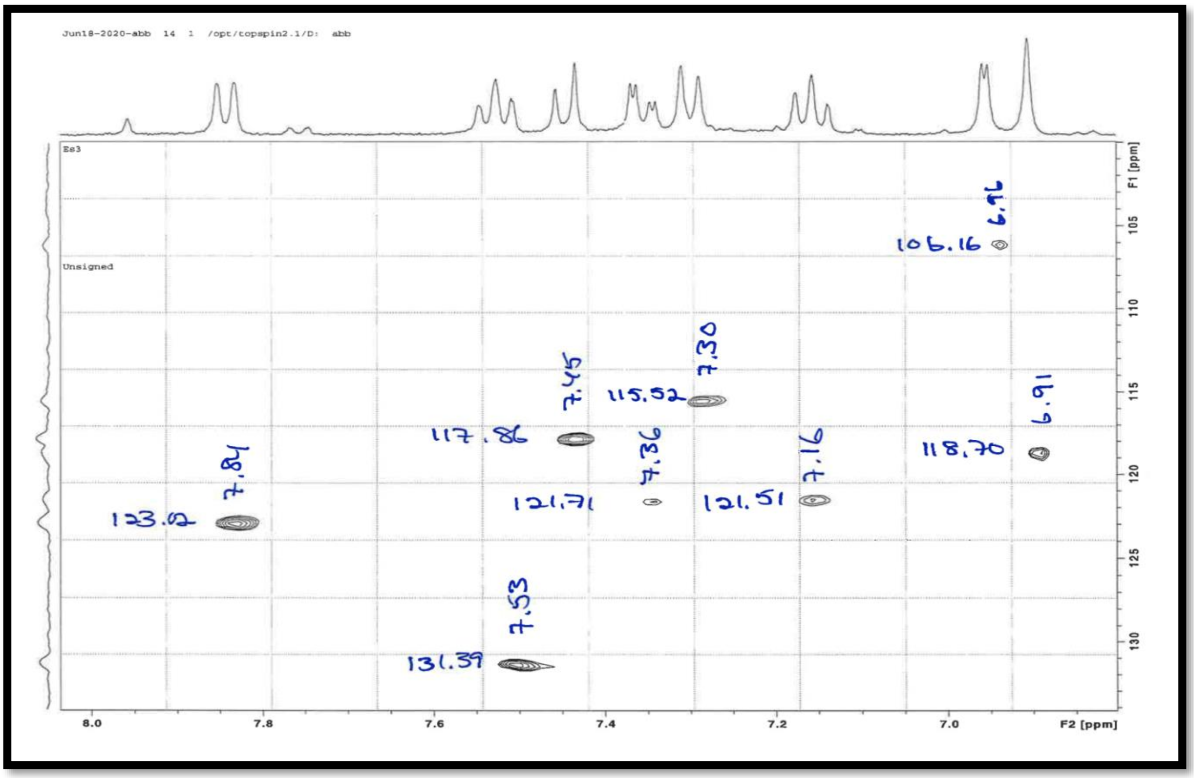

SI Fig. 55. A section of ^{1}H ^{13}C HSQC spectrum (DMSO-d_6) of compound **6c**.

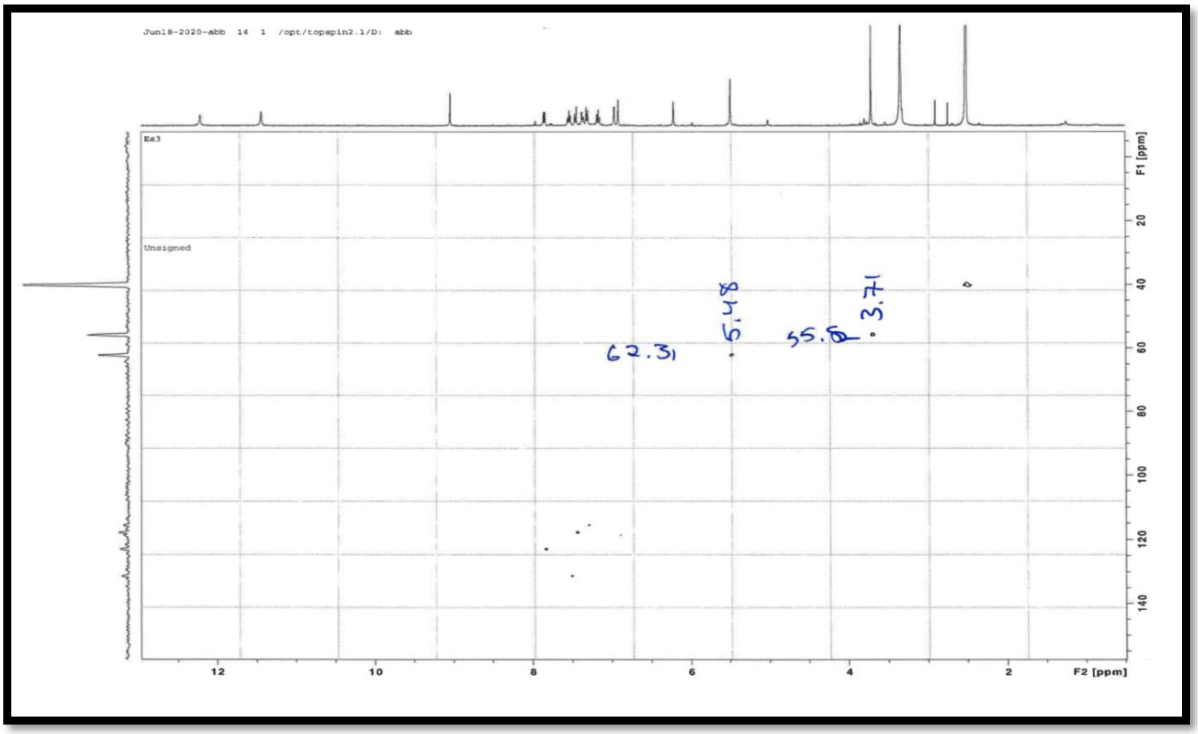

SI Fig. 56. ^{1}H ^{13}C HMBC spectrum (DMSO-d_6) of compound **6c**.

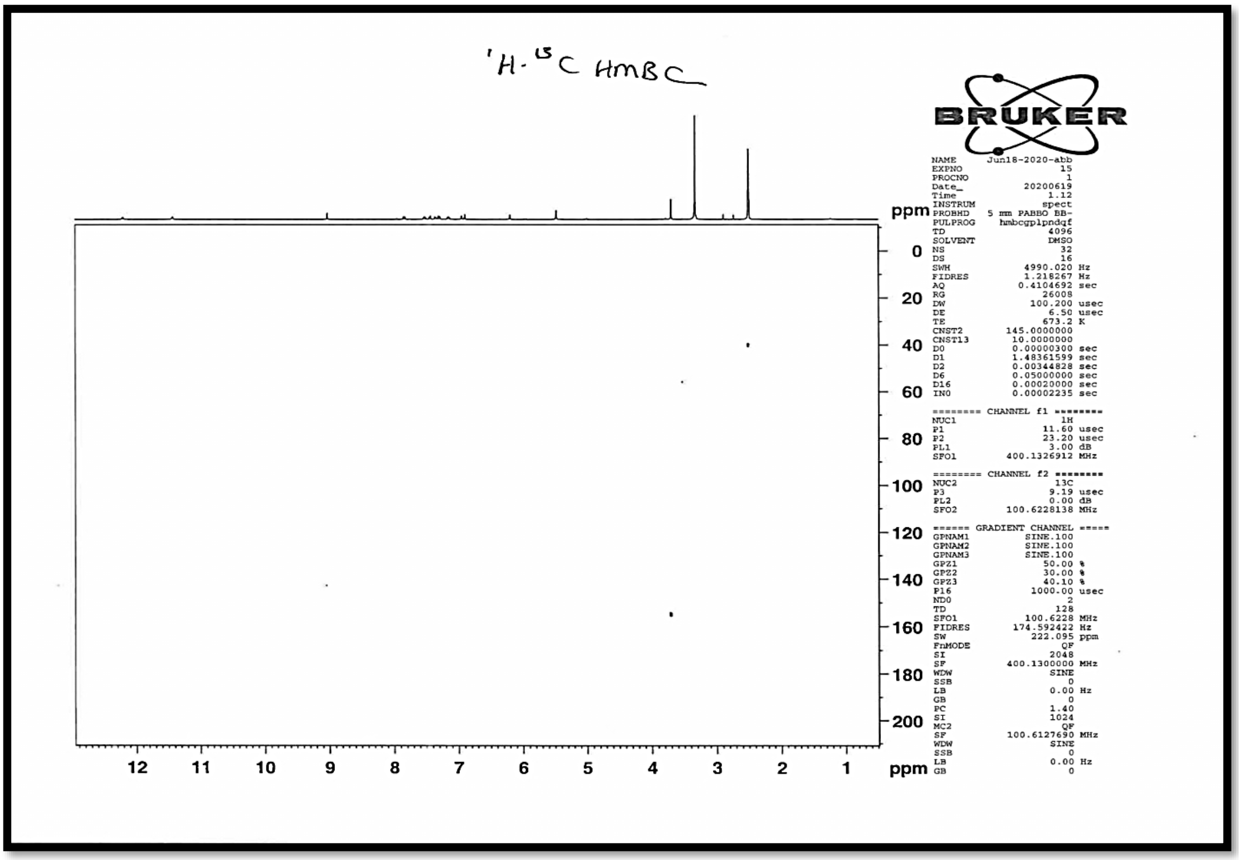

SI Fig. 57. A section of ^{1}H ^{13}C HMBC spectrum (DMSO-d_6) of compound **6c**.

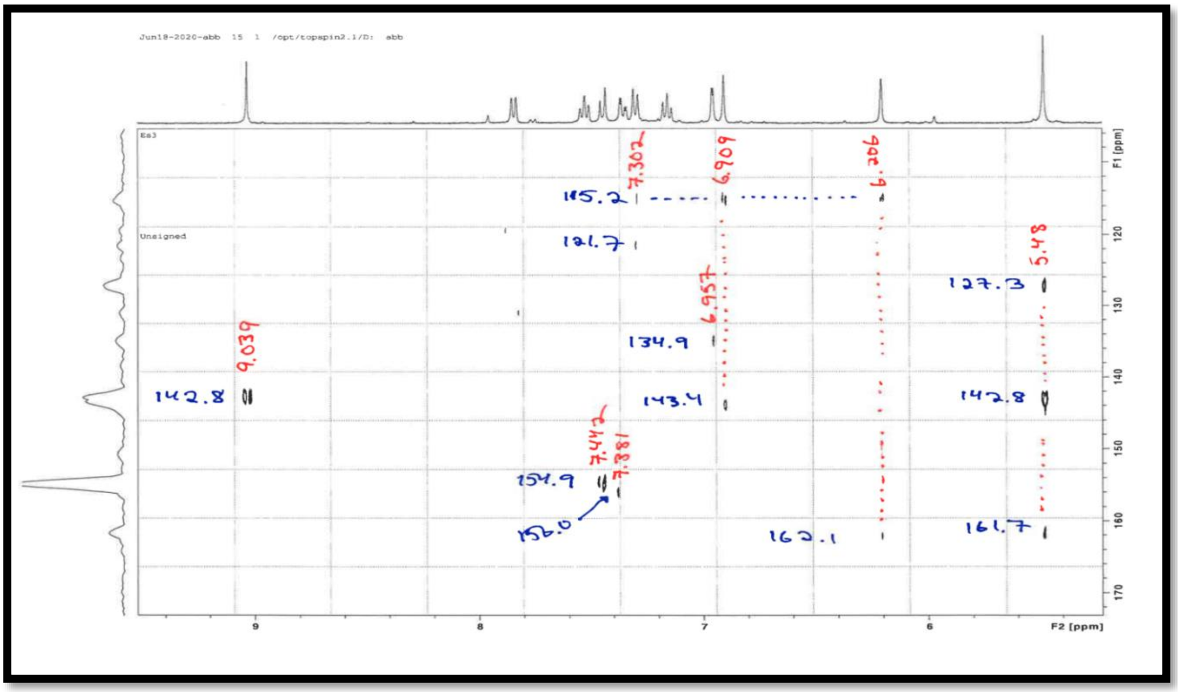

SI Fig. 58. The mass spectrum of compound **6c**.

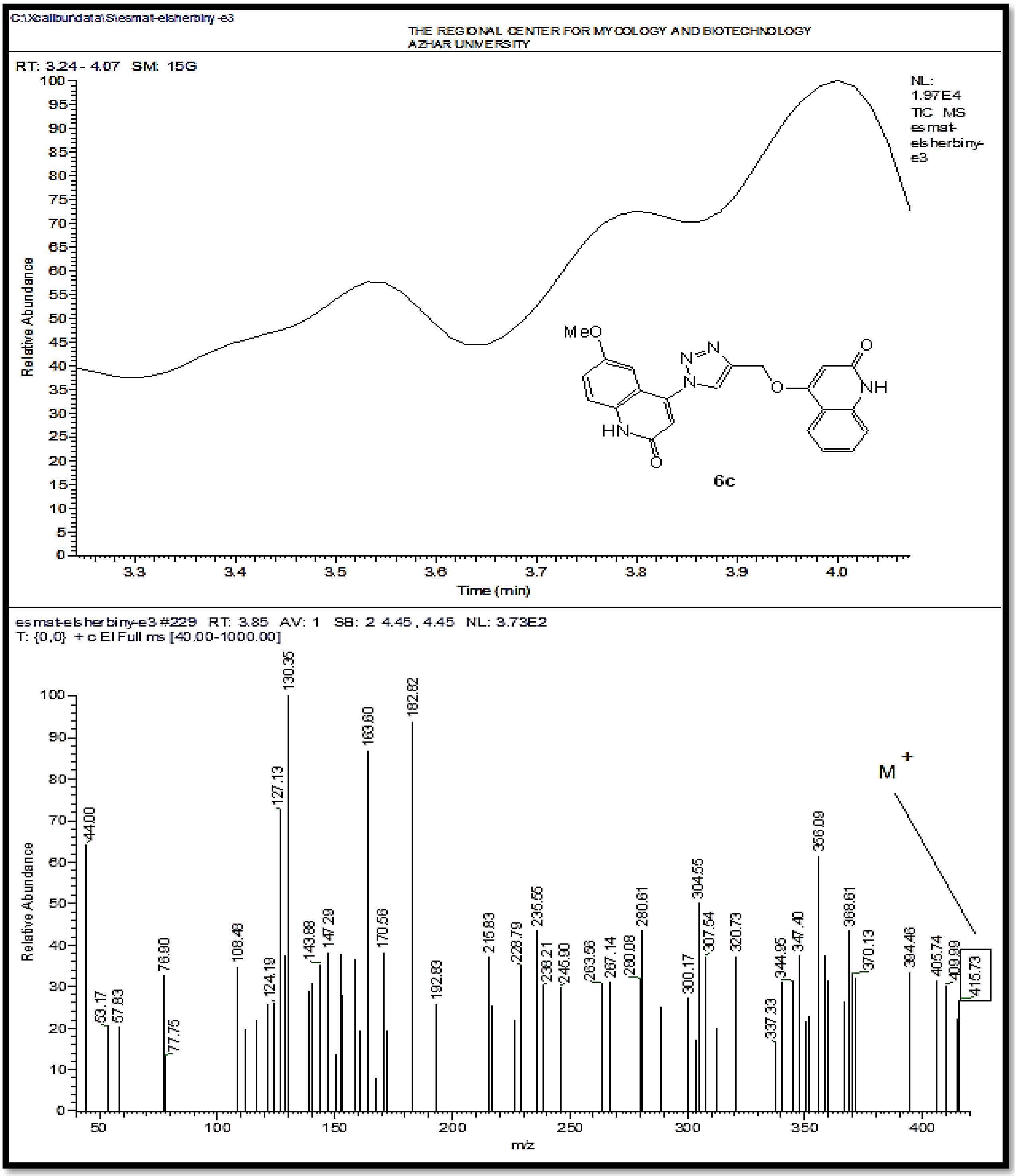

C:\Xcalibur\data\S\esmat-elsherbiny-e3
THE REGIONAL CENTER FOR MYCOLOGY AND BIOTECHNOLOGY
AZHAR UNIVERSITY
RT: 3.24 - 4.07 SM: 15G
NL: 1.97E4
TIC MS esmat-elsherbiny-e3
Relative Abundance
Time (min)
MeO
HN
NH
O
6c
esmat-elsherbiny-e3 #229 RT: 3.85 AV: 1 SB: 2 4.45 , 4.45 NL: 3.73E2
T: {0,0} + c EI Full ms [40.00-1000.00]
M+
415.73
m/z

SI Fig. 59. ^{1}H NMR spectrum (DMSO-d_6) of compound **6d**.

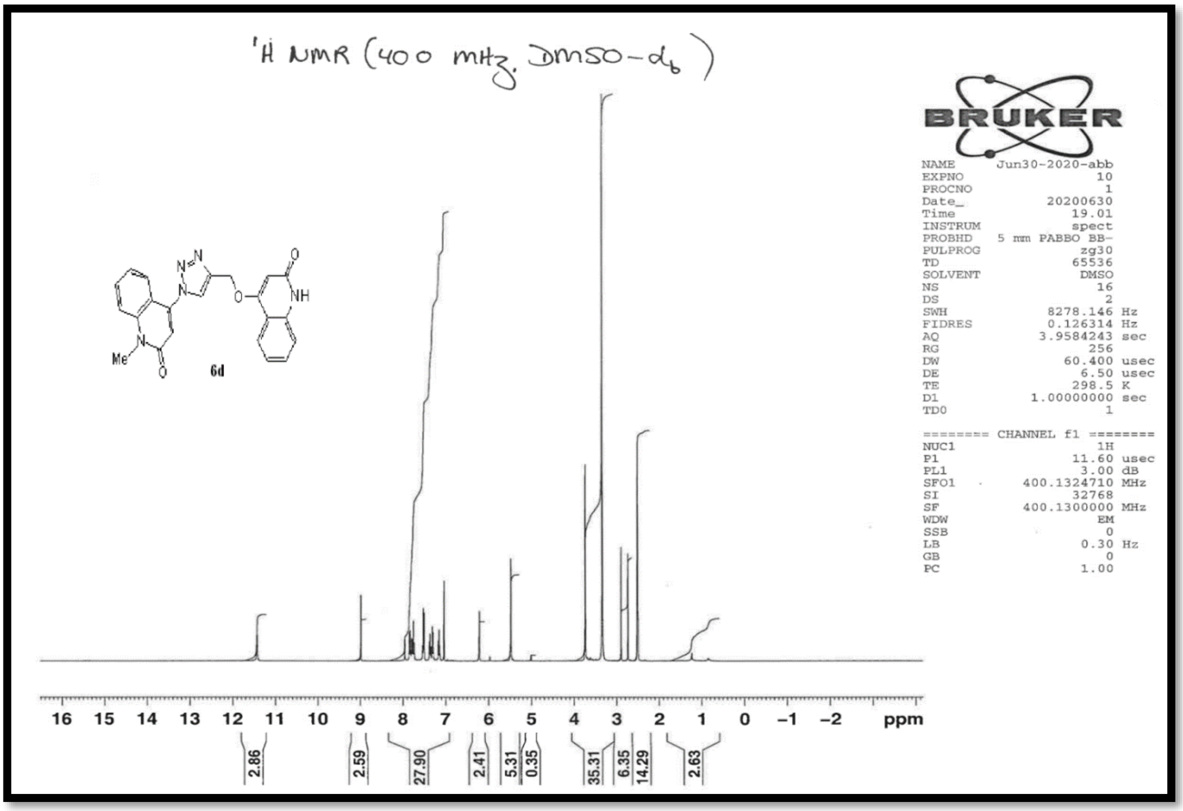

SI Fig. 60. A section of ^{1}H NMR spectrum (DMSO-d_6) of compound **6d**.

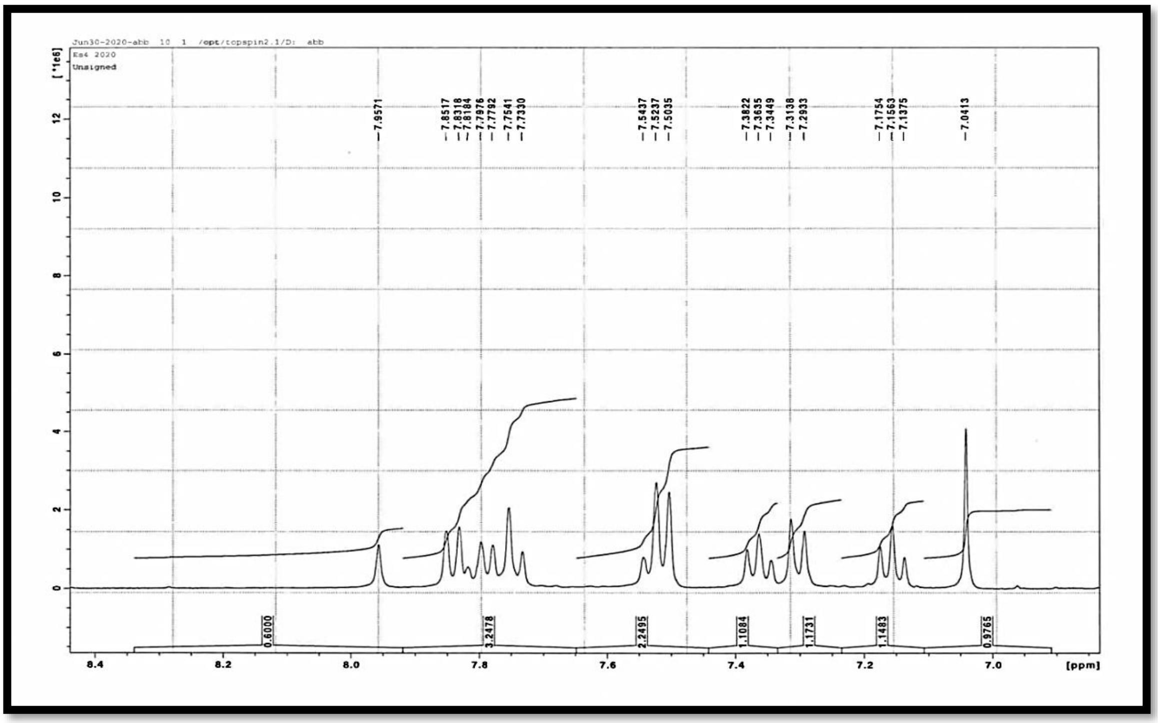

SI Fig. 61. ^{13}C NMR spectrum (DMSO-d_6) of compound **6d**.

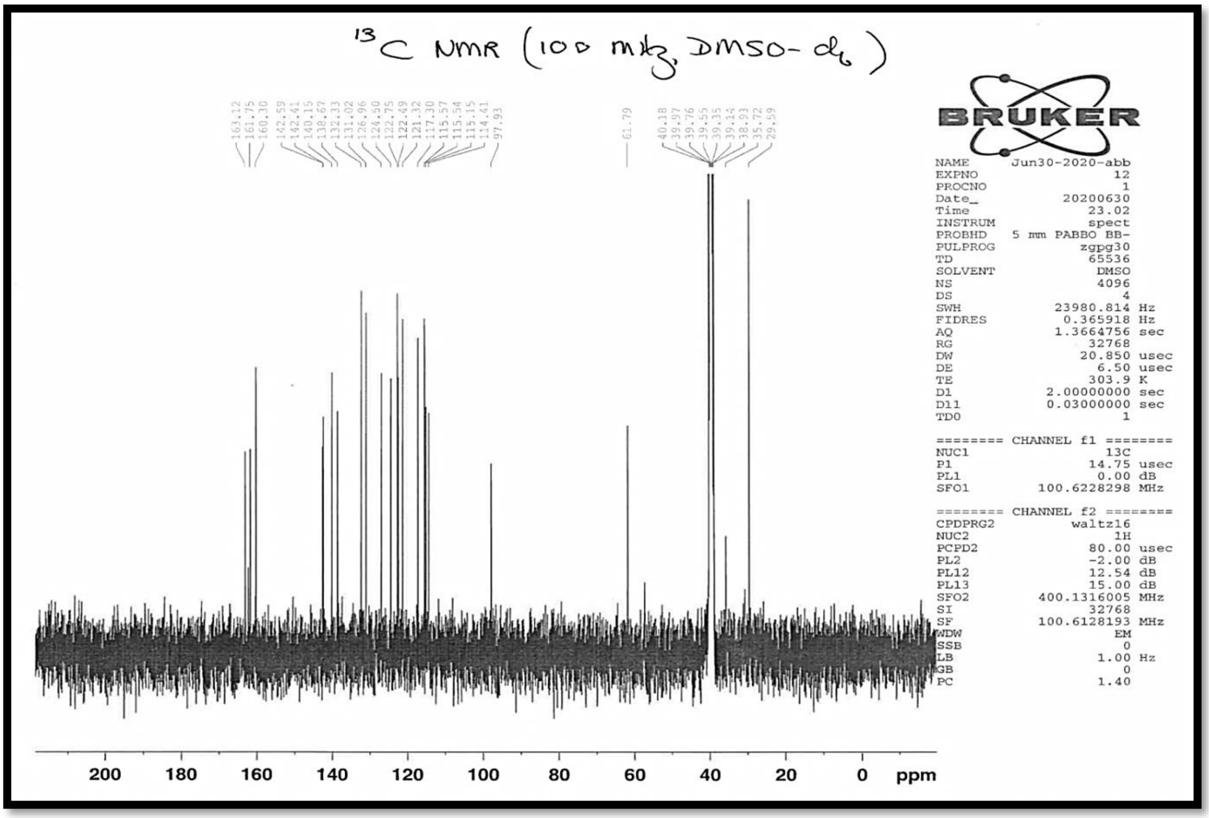

SI Fig. 62. ^{1}H ^{1}H Cosy spectrum (DMSO-d_6) of compound **6d**.

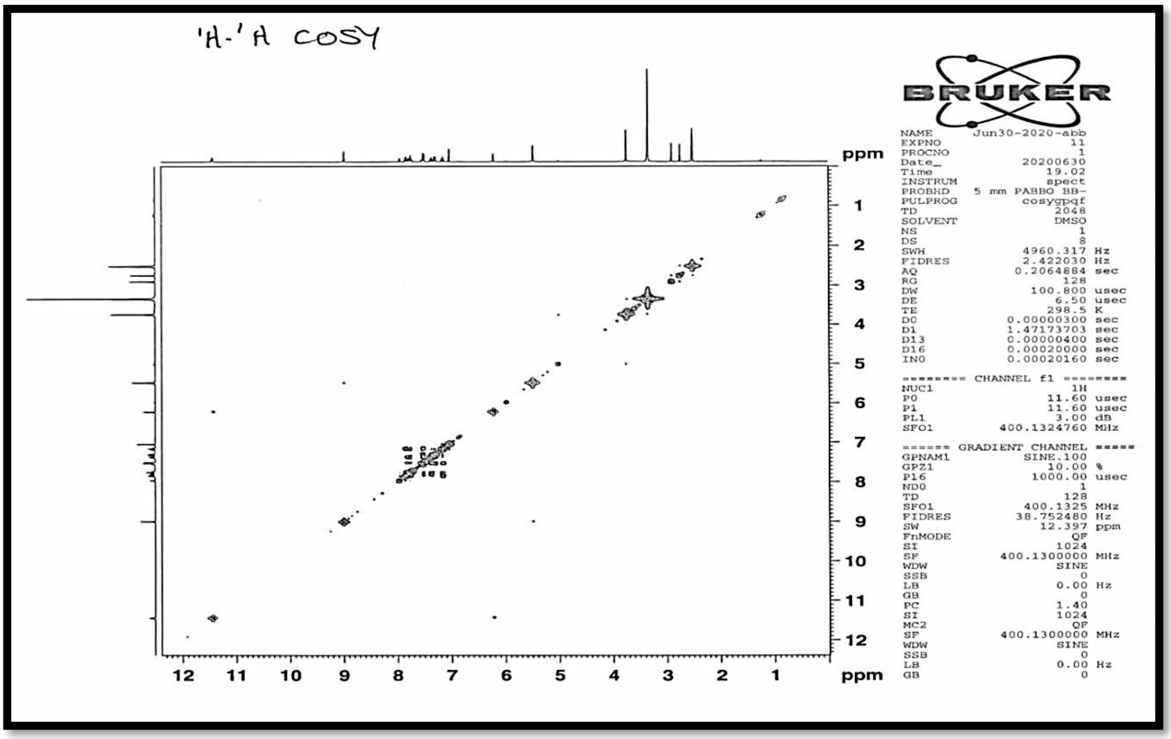

SI Fig. 63. A section of ^{1}H ^{1}H Cosy spectrum (DMSO-d_6) of compound **6d**.

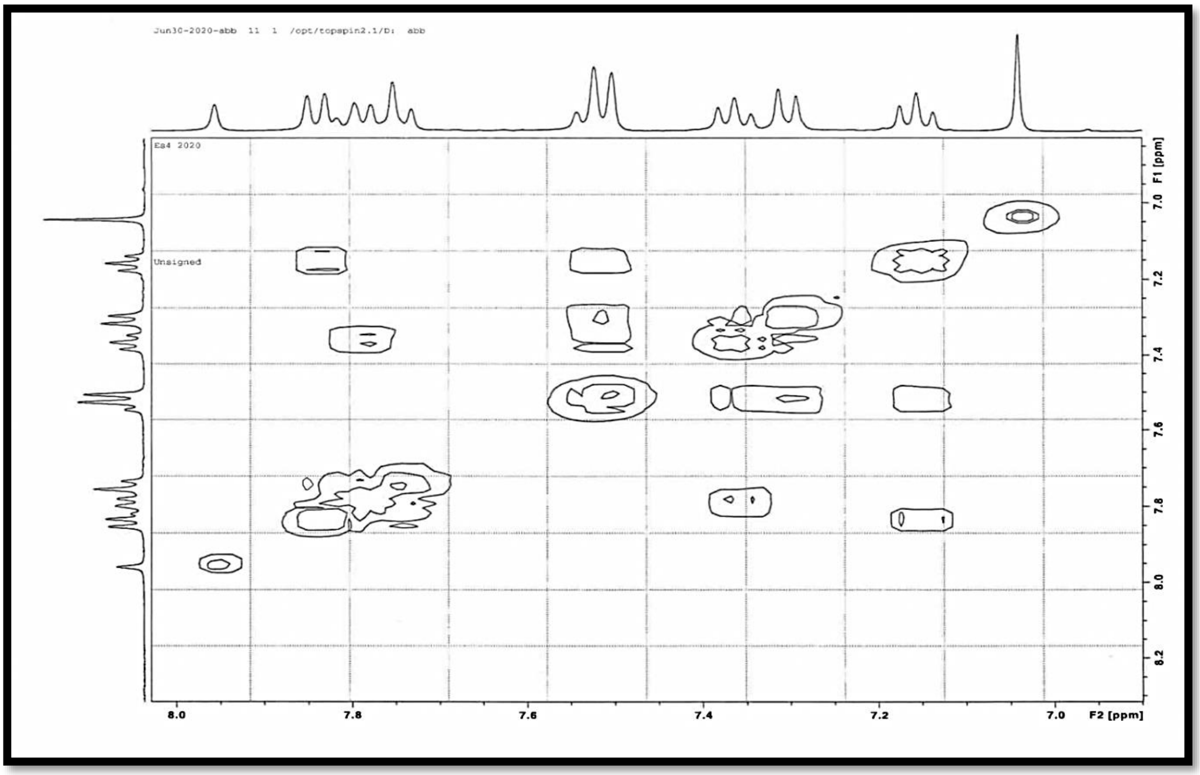

SI Fig. 64. ^{1}H ^{13}C HSQC spectrum (DMSO-d_6) of compound **6d**.

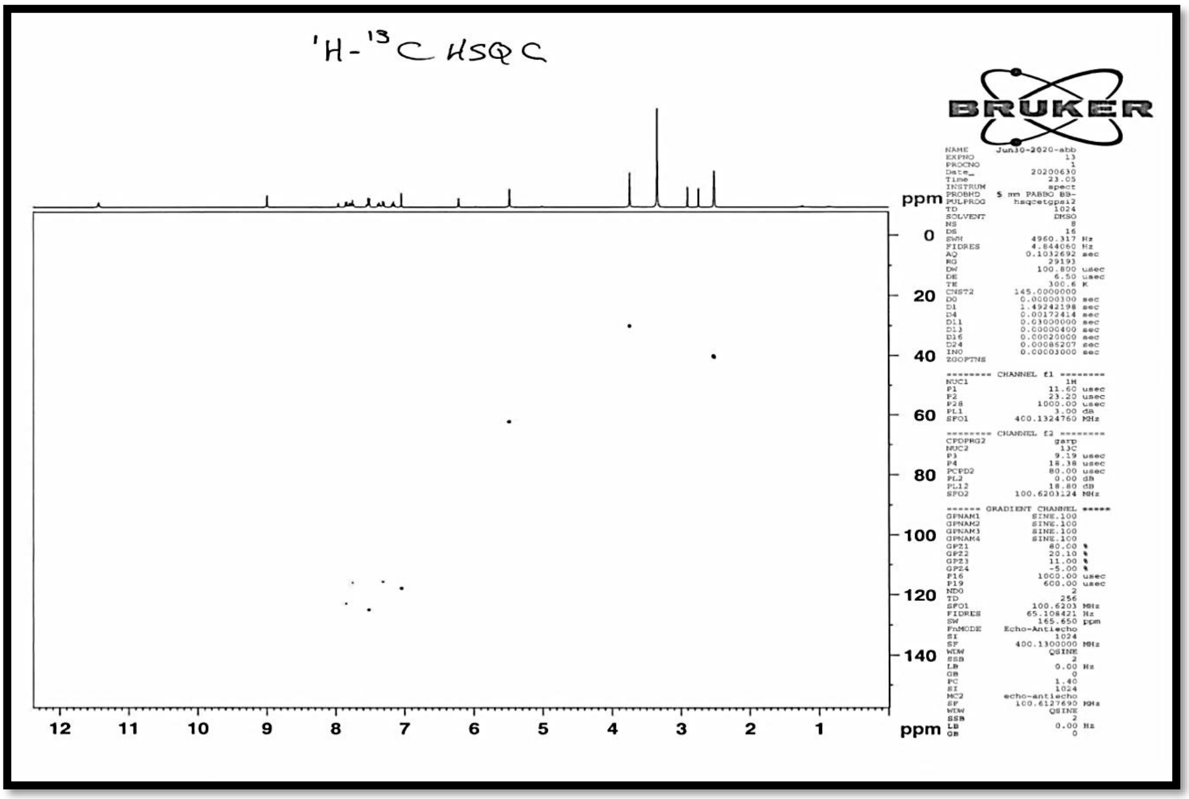

SI Fig. 65. A section of ^{1}H ^{13}C HSQC spectrum (DMSO-d_6) of compound **6d**.

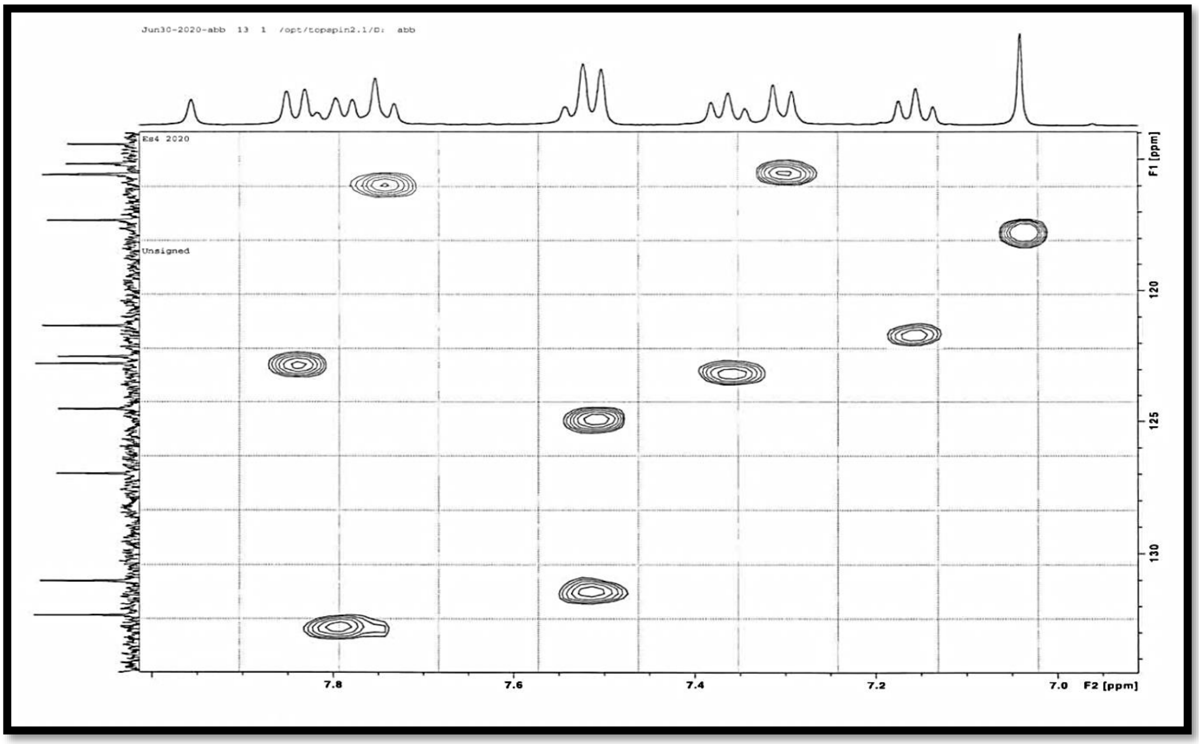

SI Fig. 66. ^{1}H ^{13}C HMBC spectrum (DMSO-d_6) of compound **6d**.

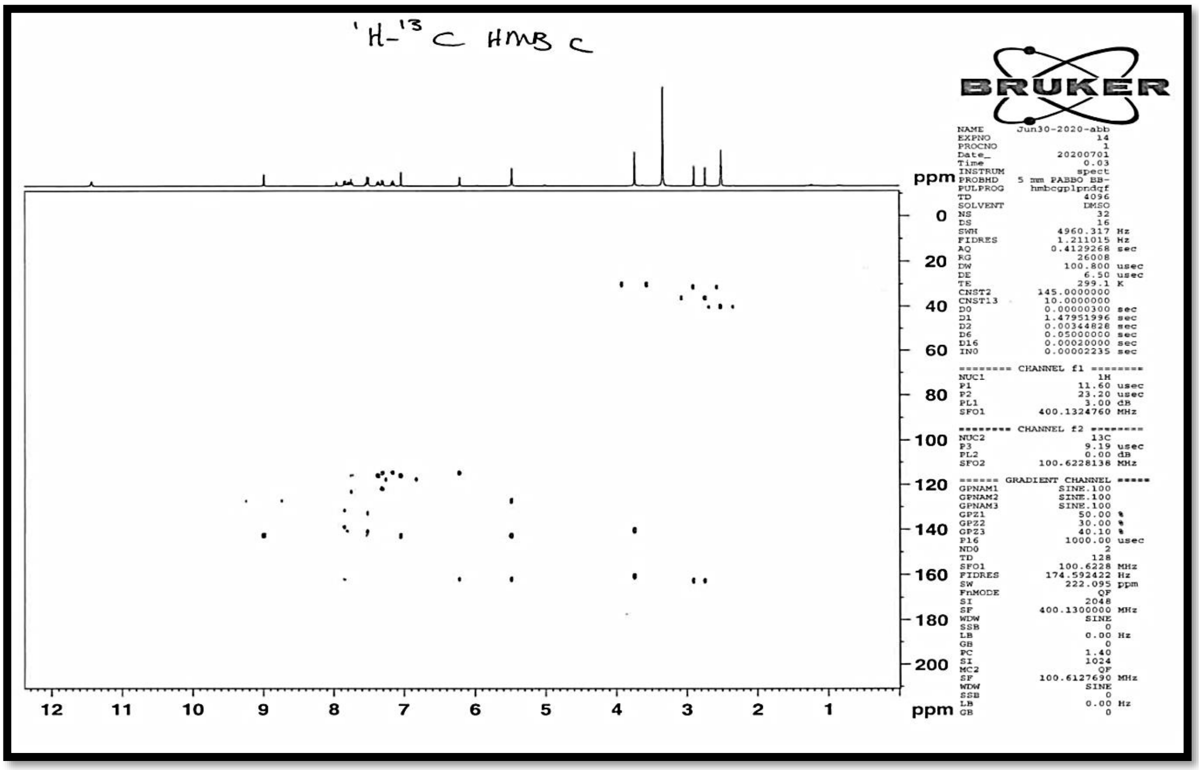

SI Fig. 67. A section of ^{1}H ^{13}C HMBC spectrum (DMSO-d_6) of compound **6d**.

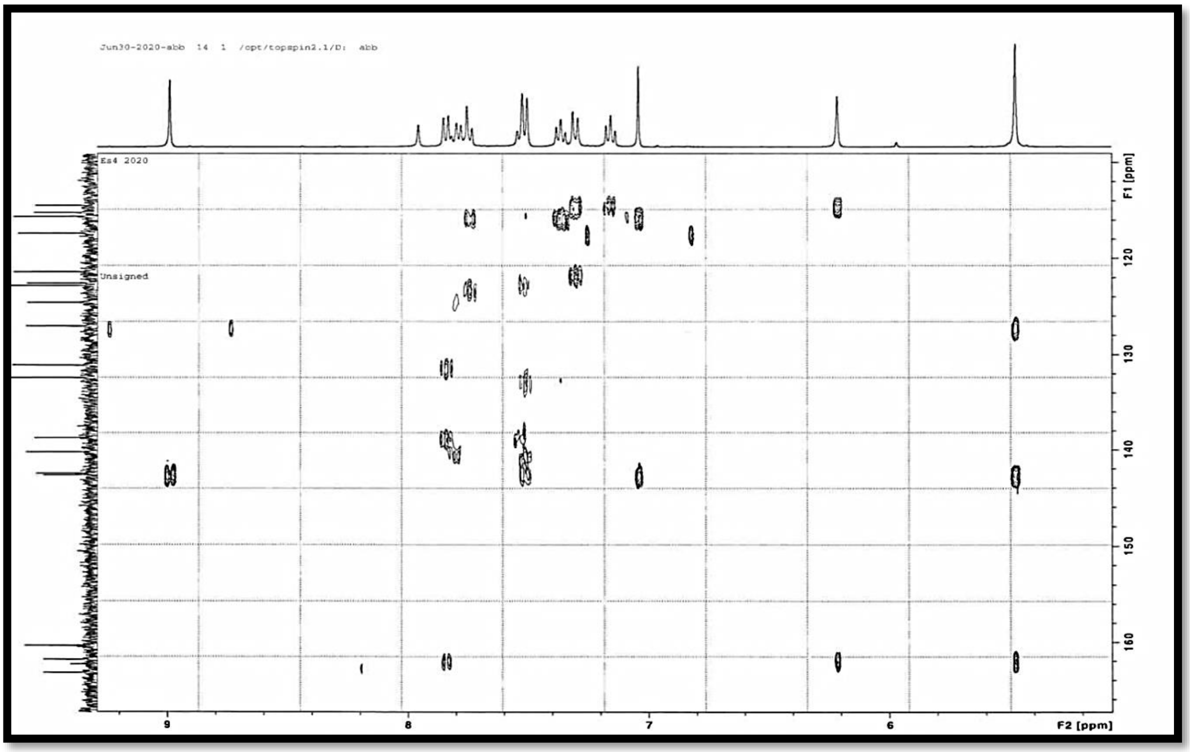

SI Fig. 68. ^{1}H ^{15}N HMBC spectrum (DMSO-d_6) of compound **6d**.

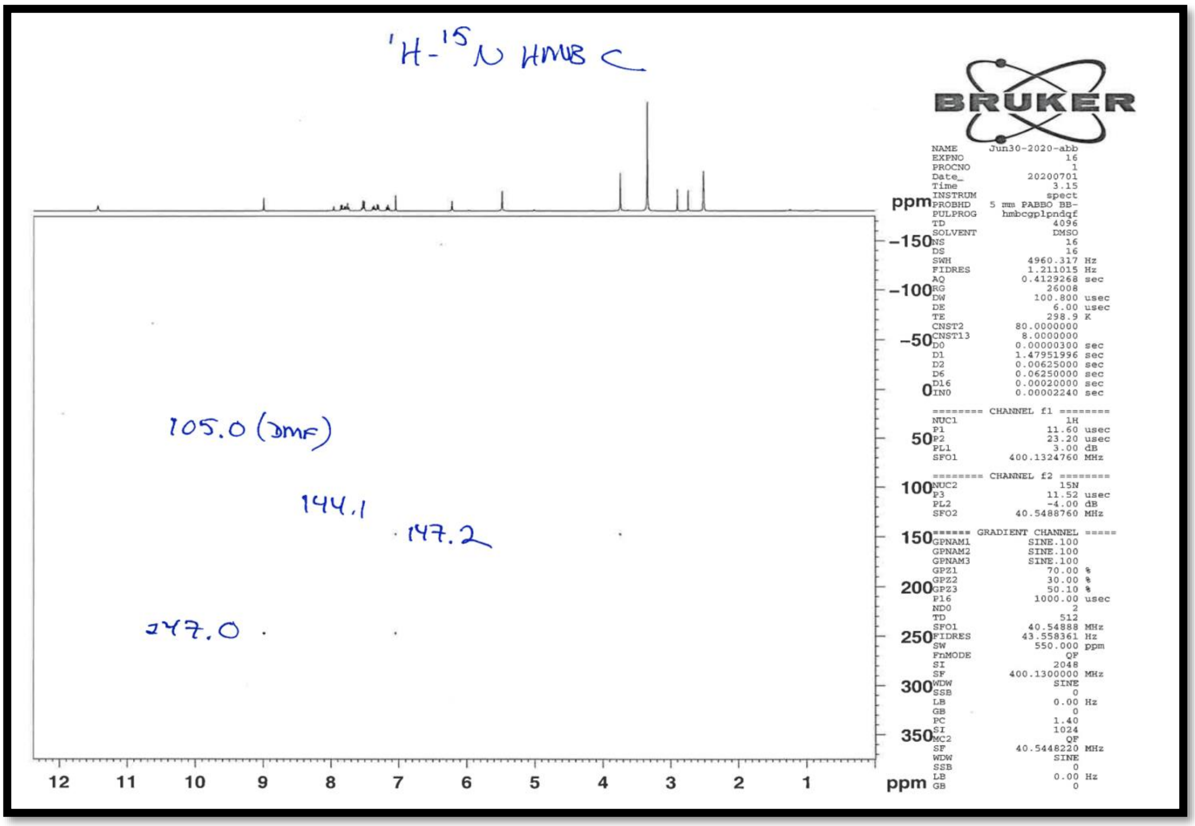

SI Fig. 69. A section of ^{1}H ^{15}N HMBC spectrum (DMSO-d_6) of compound **6d**.

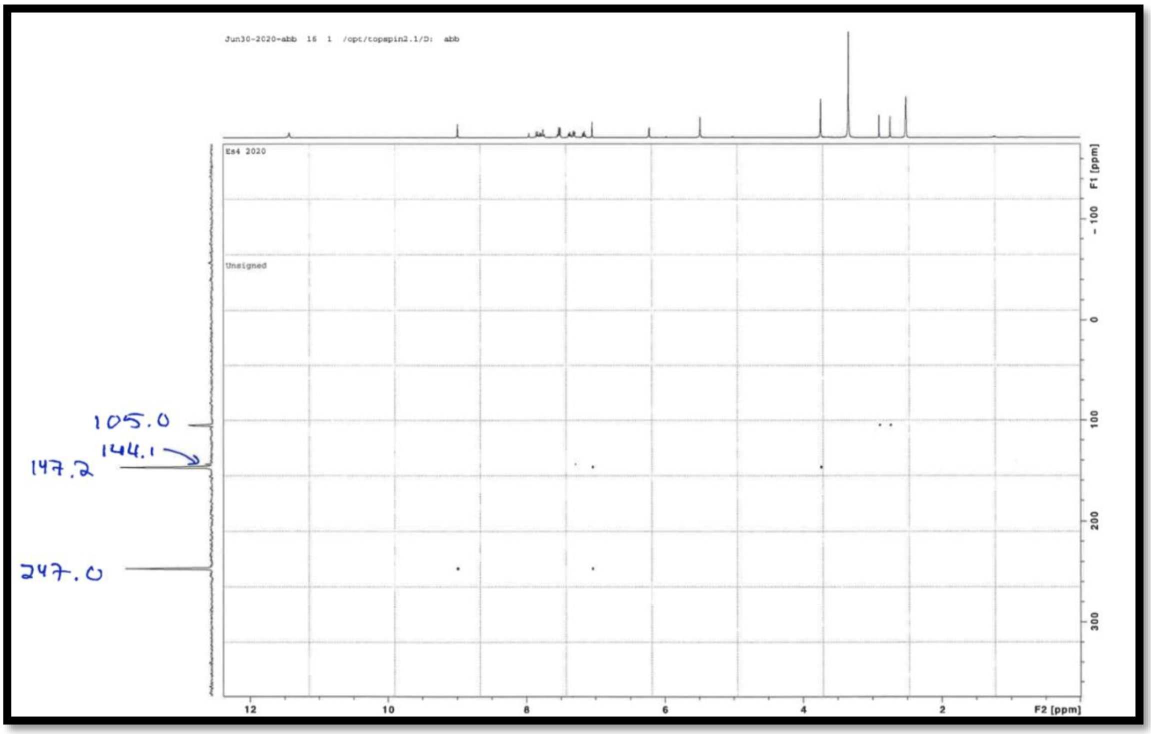

SI Fig. 70. The mass spectrum of compound **6d**.

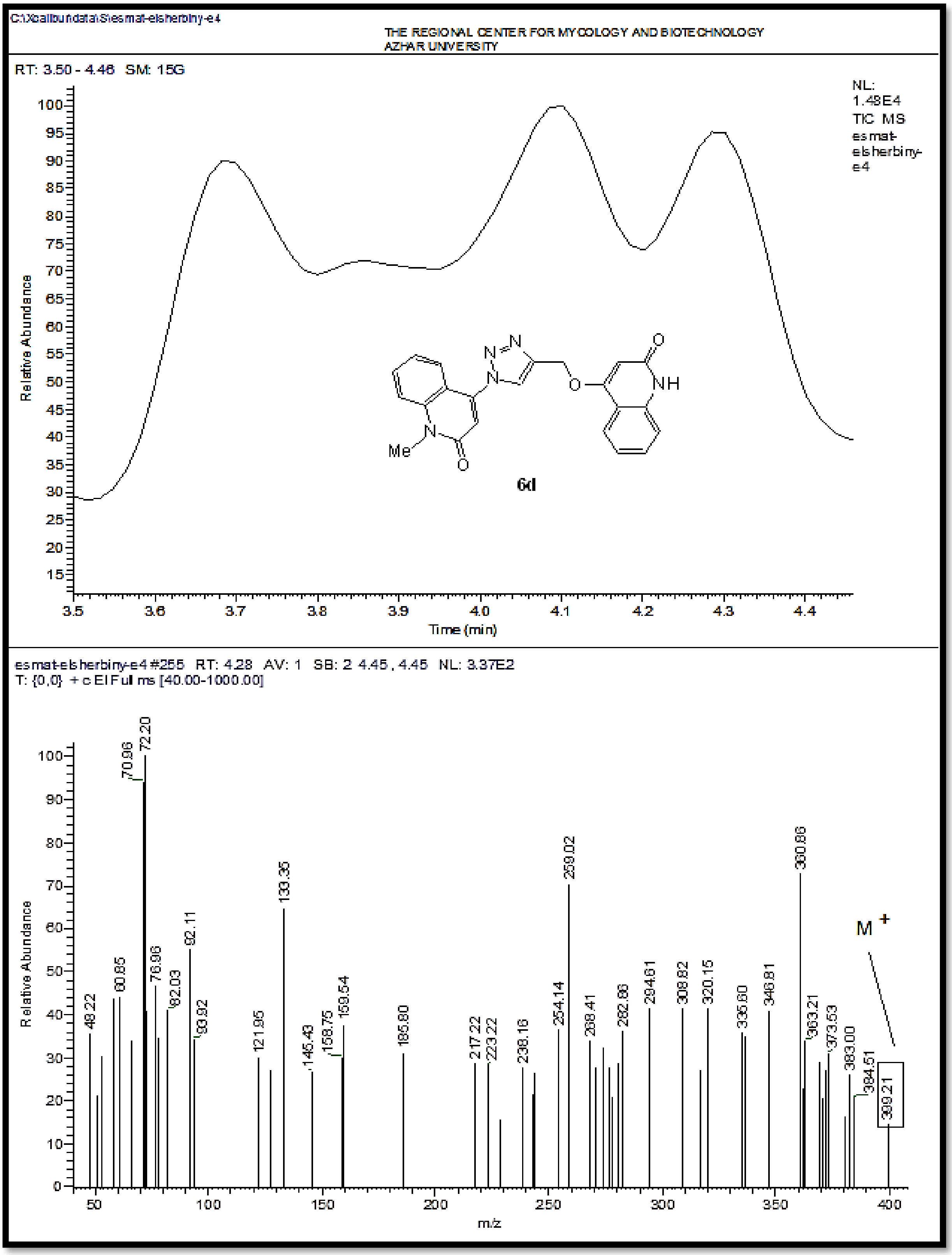

SI Fig. 71. ^{1}H NMR spectrum (DMSO-d_6) of compound **6e**.

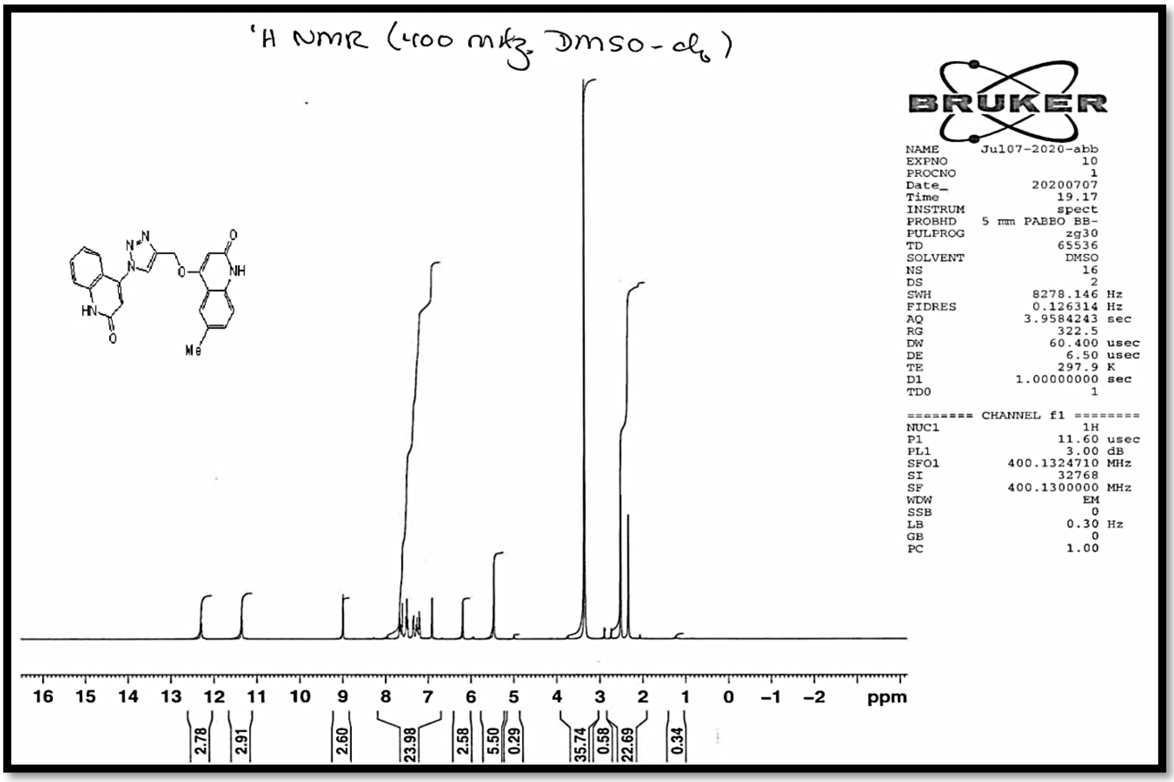

SI Fig. 72. A section of ^{1}H NMR spectrum (DMSO-d_6) of compound **6e**.

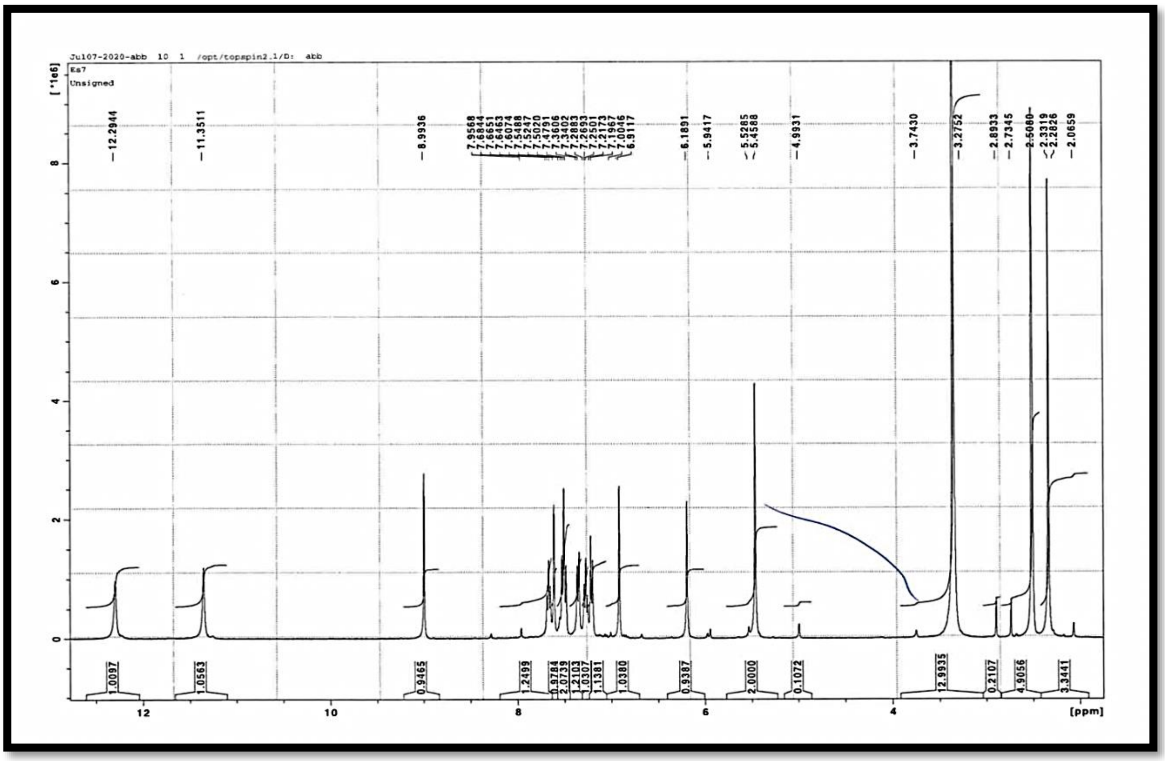

SI Fig. 73. ^{13}C NMR spectrum (DMSO-d_6) of compound **6e**.

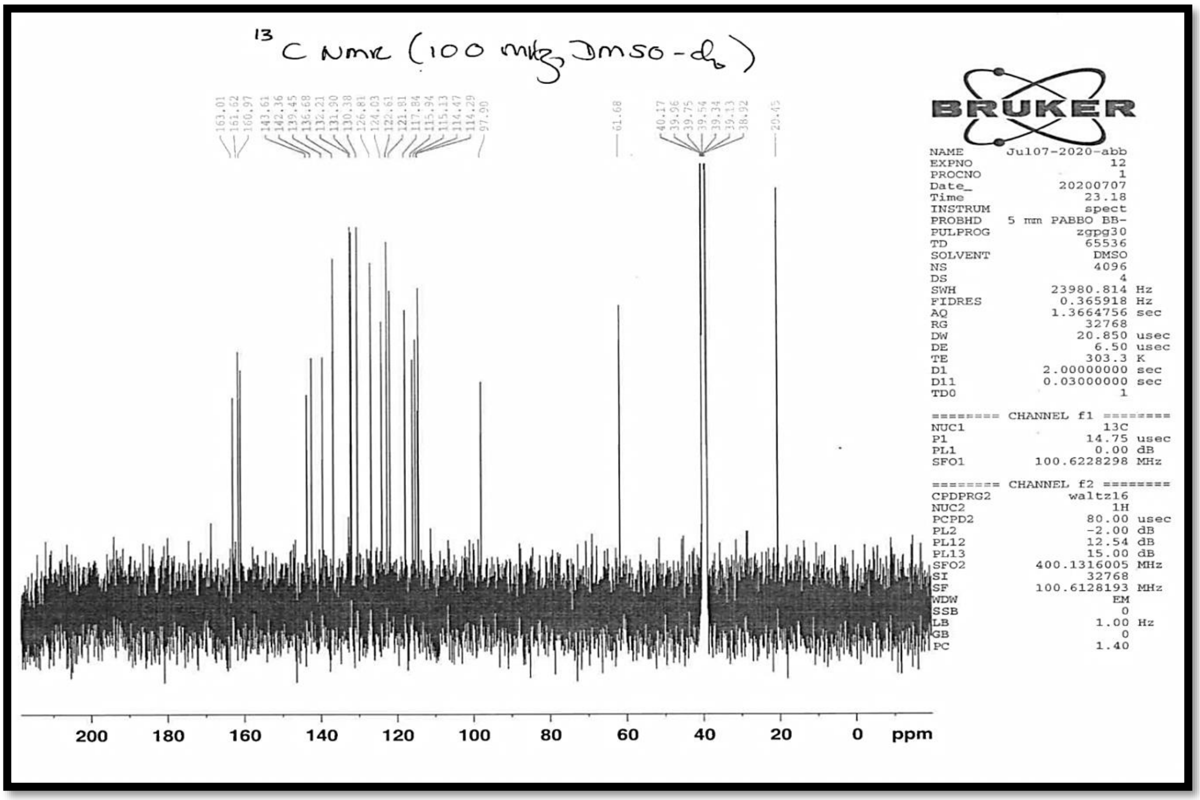

SI Fig. 74. ^{1}H ^{1}H Cosy spectrum (DMSO-d_6) of compound **6e**.

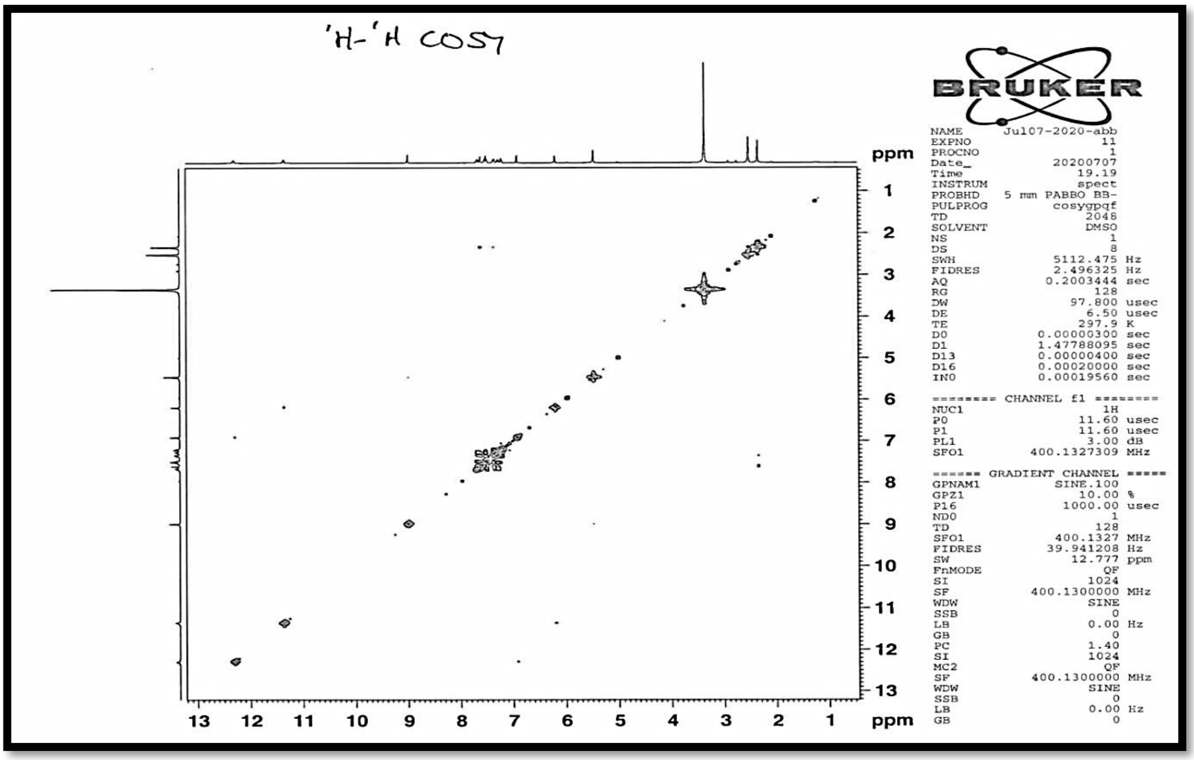

SI Fig. 75. ^{1}H ^{13}C HSQC spectrum (DMSO-d_6) for compound **6e**.

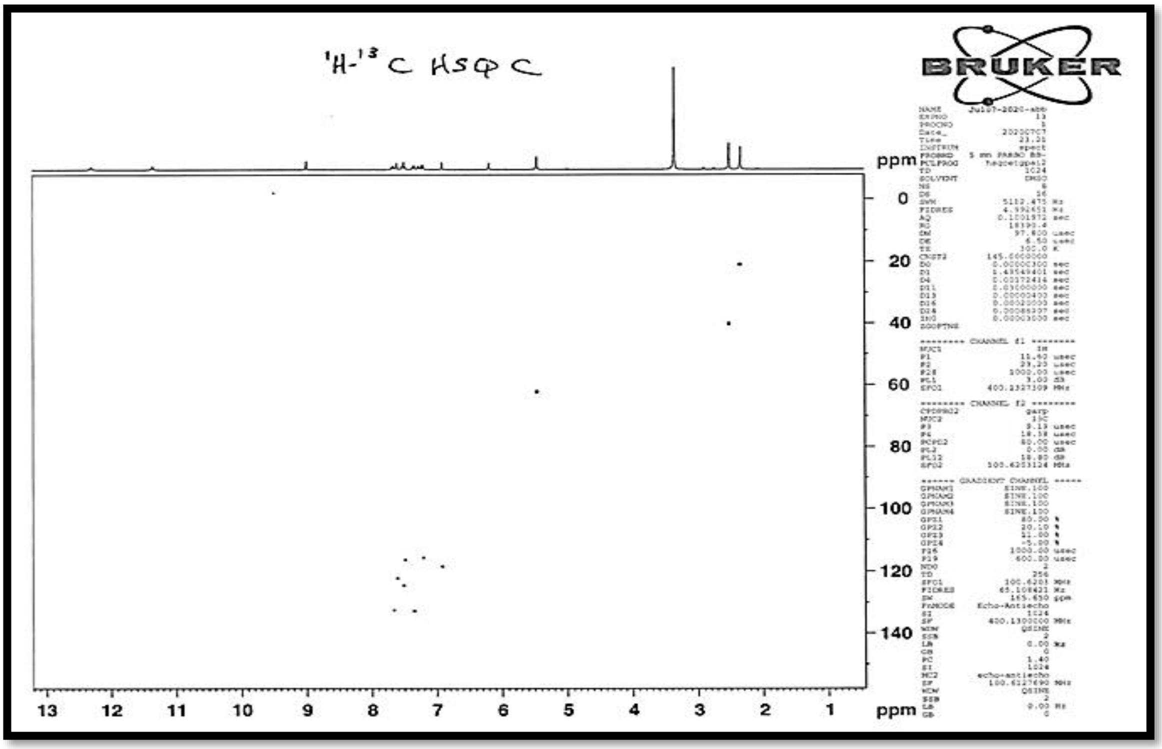

SI Fig. 76. A section of ^{1}H ^{13}C HSQC spectrum (DMSO-d_6) of compound **6e**.

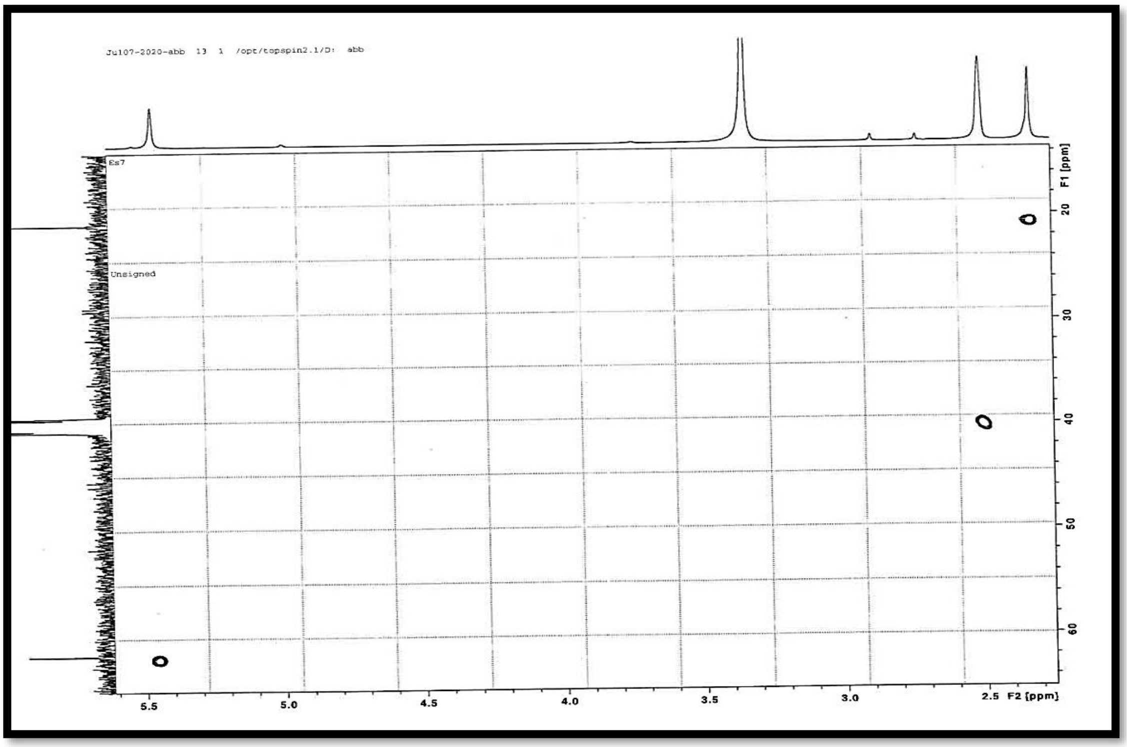

SI Fig. 77. ^{1}H ^{13}C HMBC spectrum (DMSO-d_6) of compound **6e**.

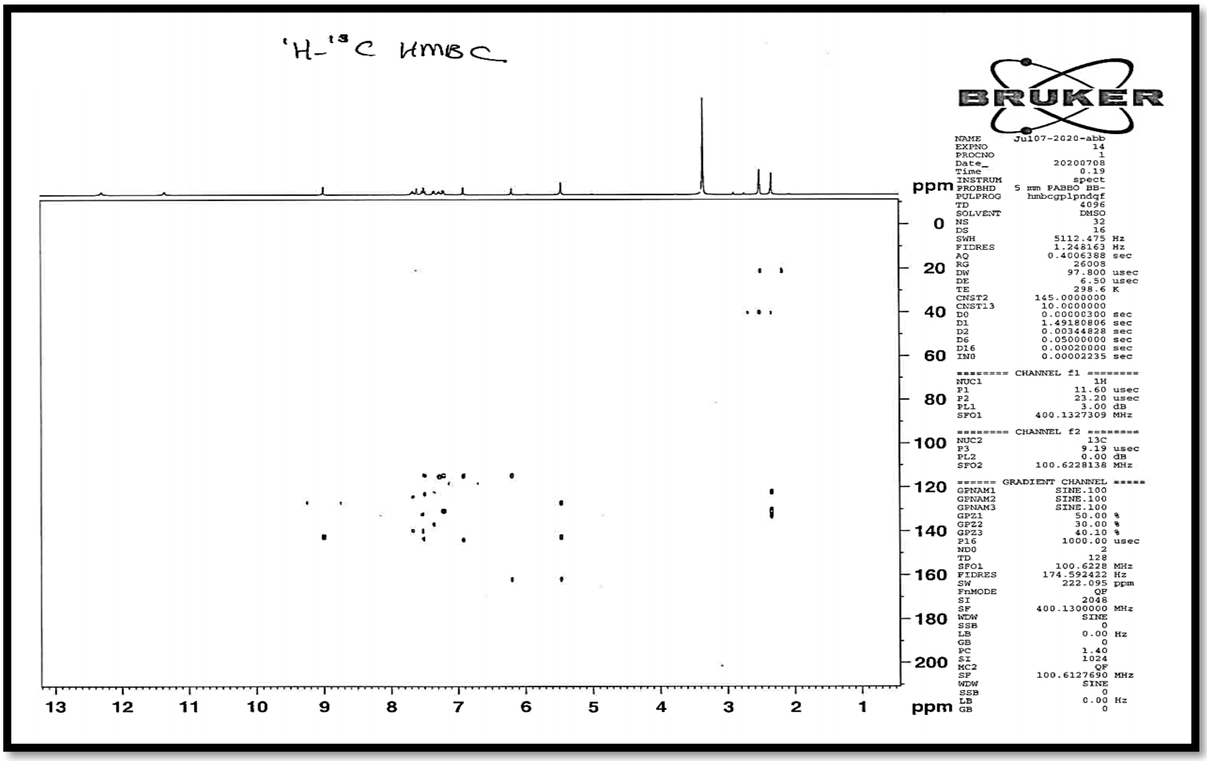

SI Fig. 78. A section of ^{1}H ^{13}C HMBC spectrum (DMSO-d_6) of compound **6e**.

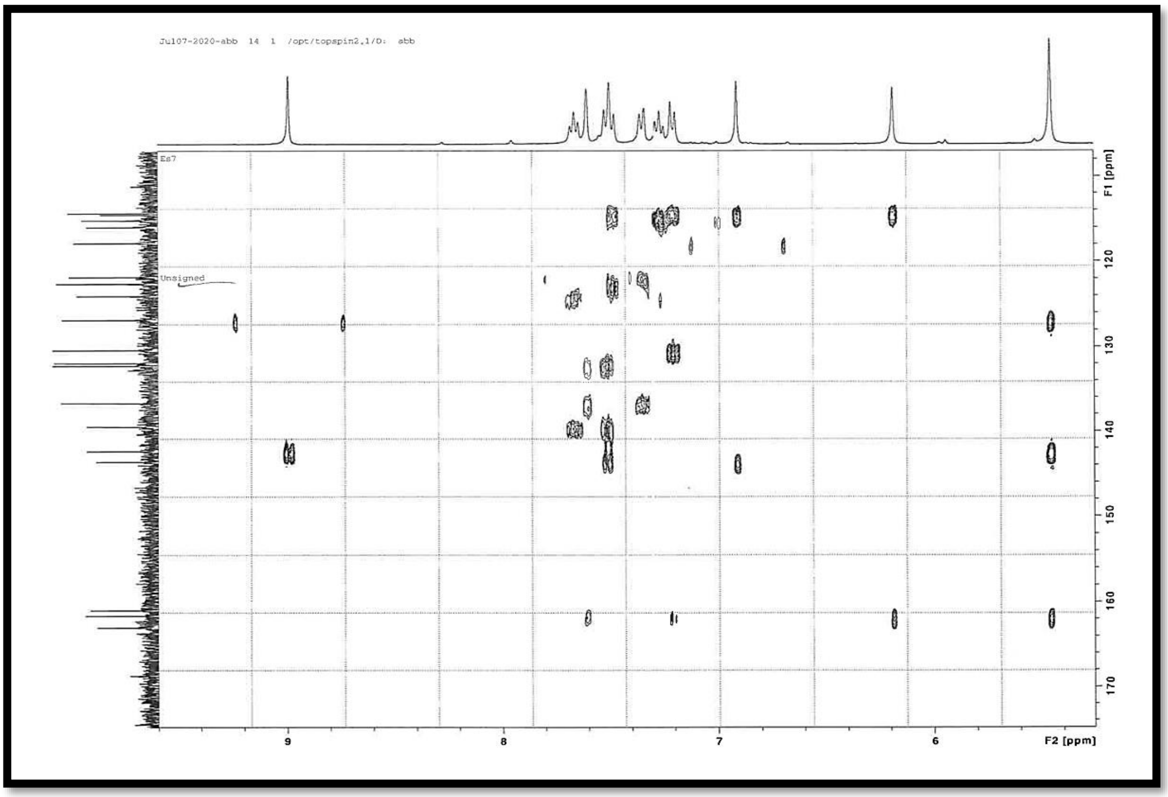

SI Fig. 79. ^{1}H ^{15}N HSQC spectrum (DMSO-d_6) of compound **6e**.

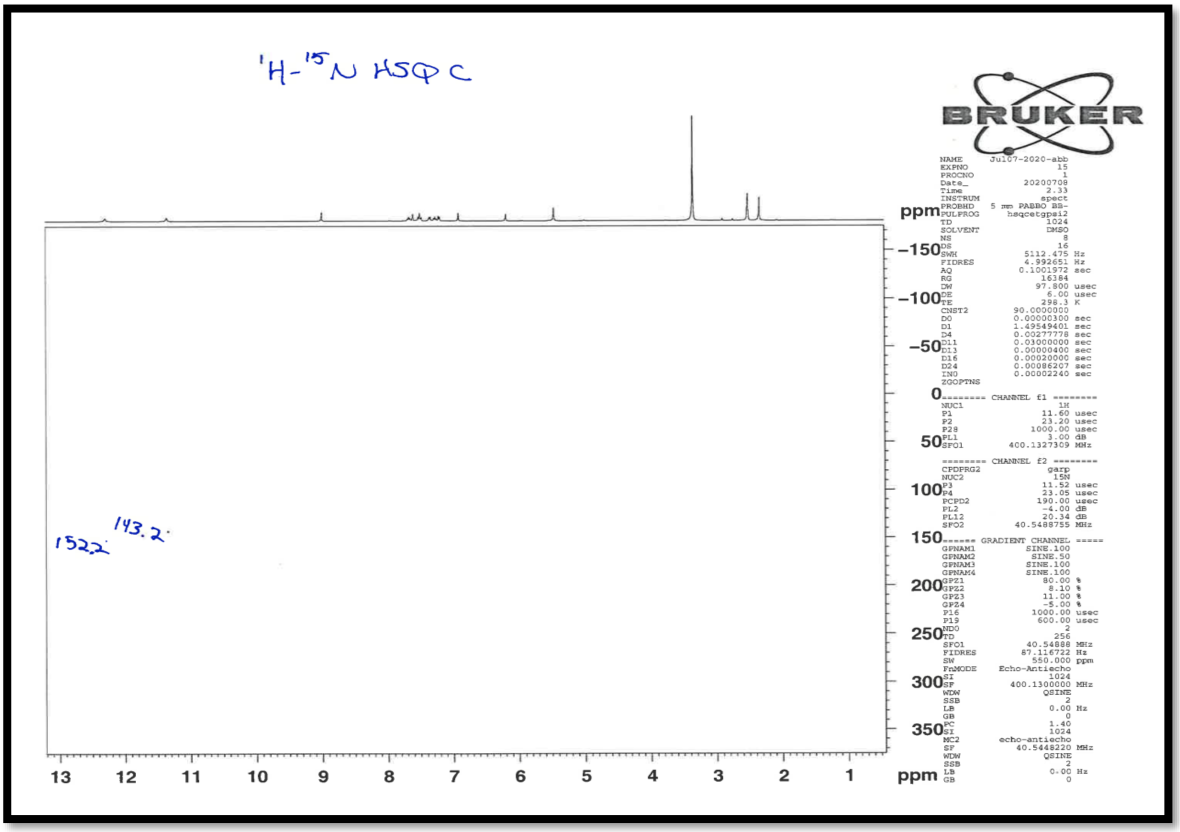

SI Fig. 80. ^{1}H ^{15}N HMBC spectrum (DMSO-d_6) of compound **6e**.

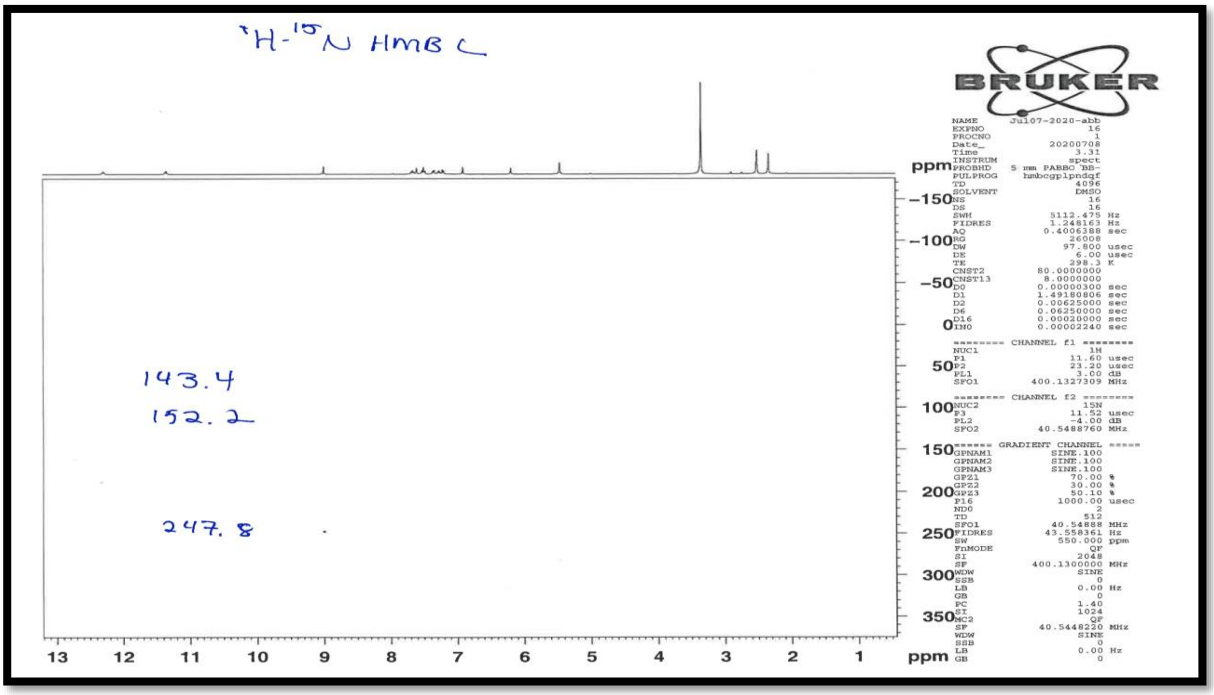

SI Fig. 81. The mass spectrum of compound **6e**.

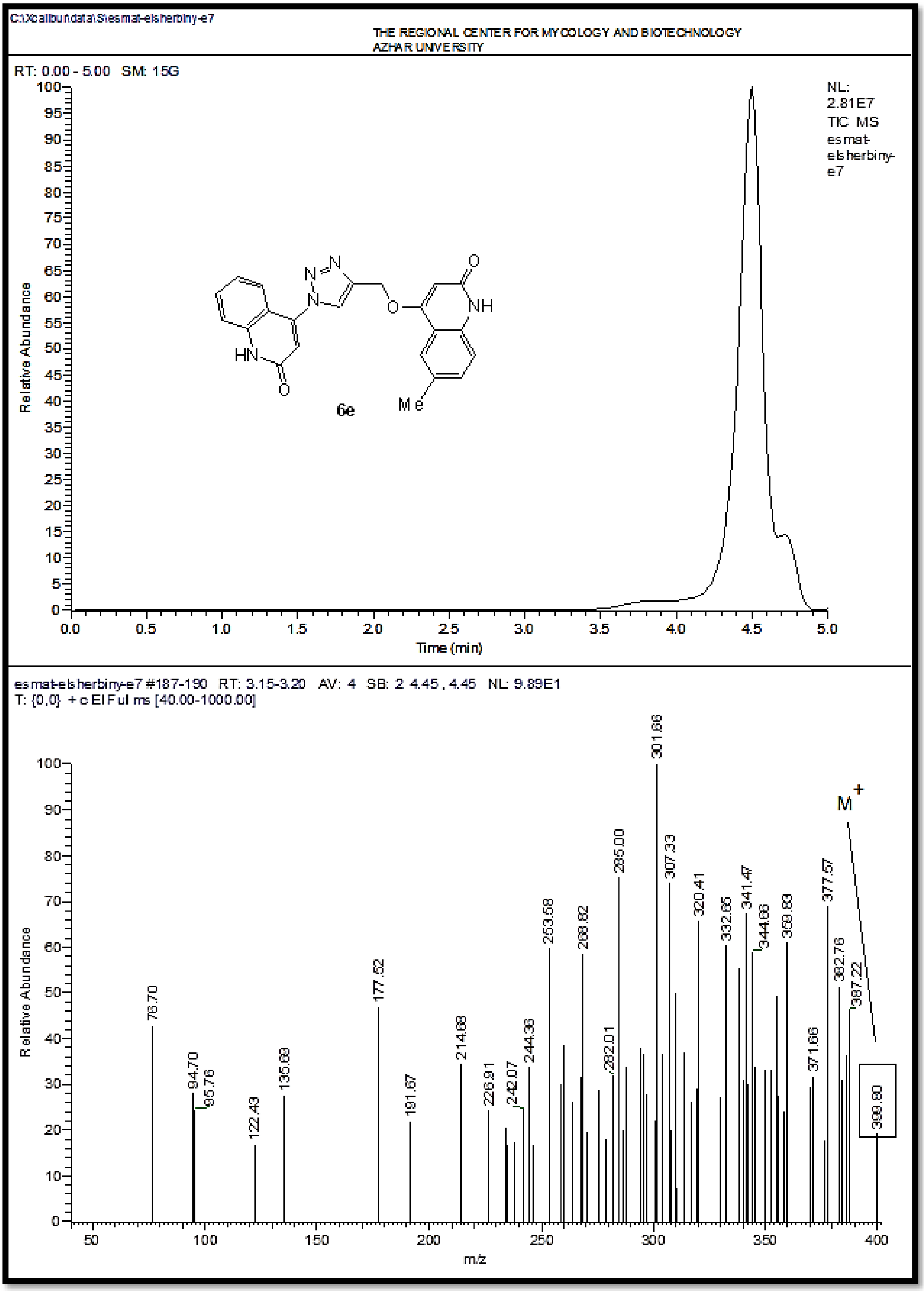

SI Fig. 82. ^{1}H NMR spectrum (DMSO-d_6) of compound **6f**.

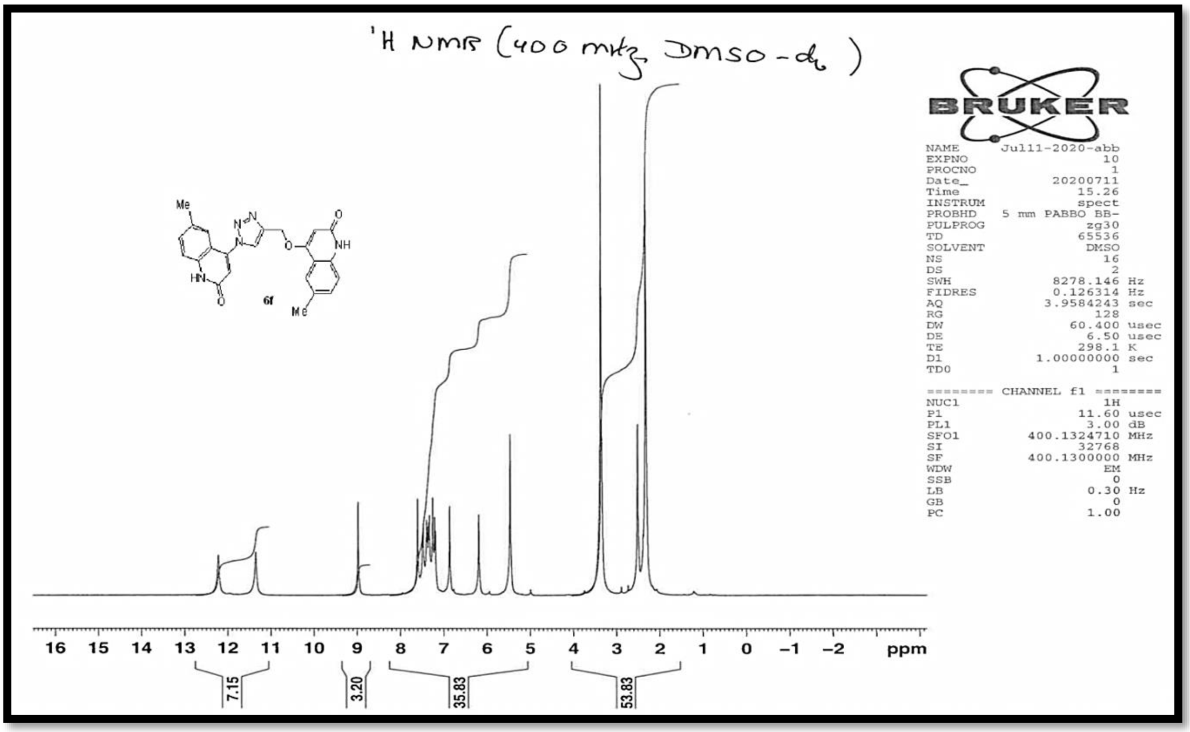

SI Fig. 83. A section of ^{1}H NMR spectrum (DMSO-d_6) of compound **6f**.

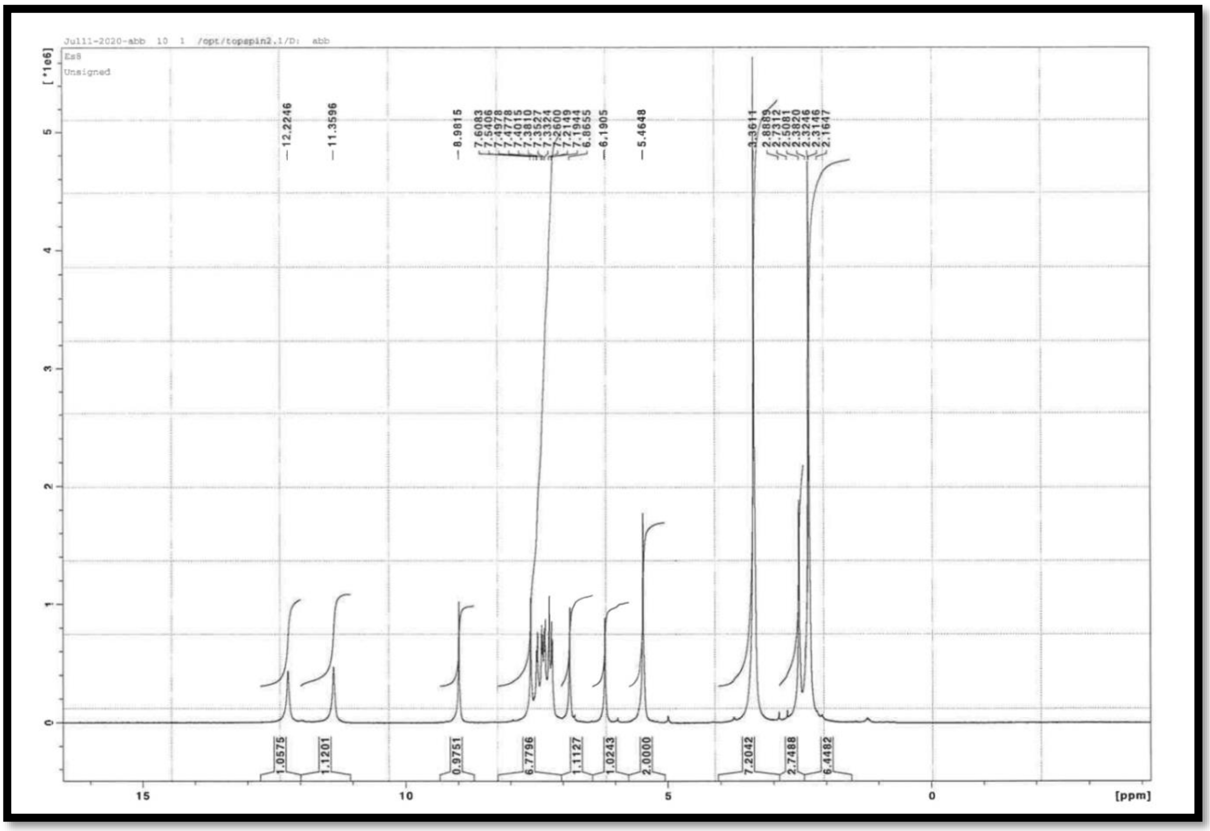

SI Fig. 84. ^{13}C NMR spectrum (DMSO-d_6) of compound **6f**.

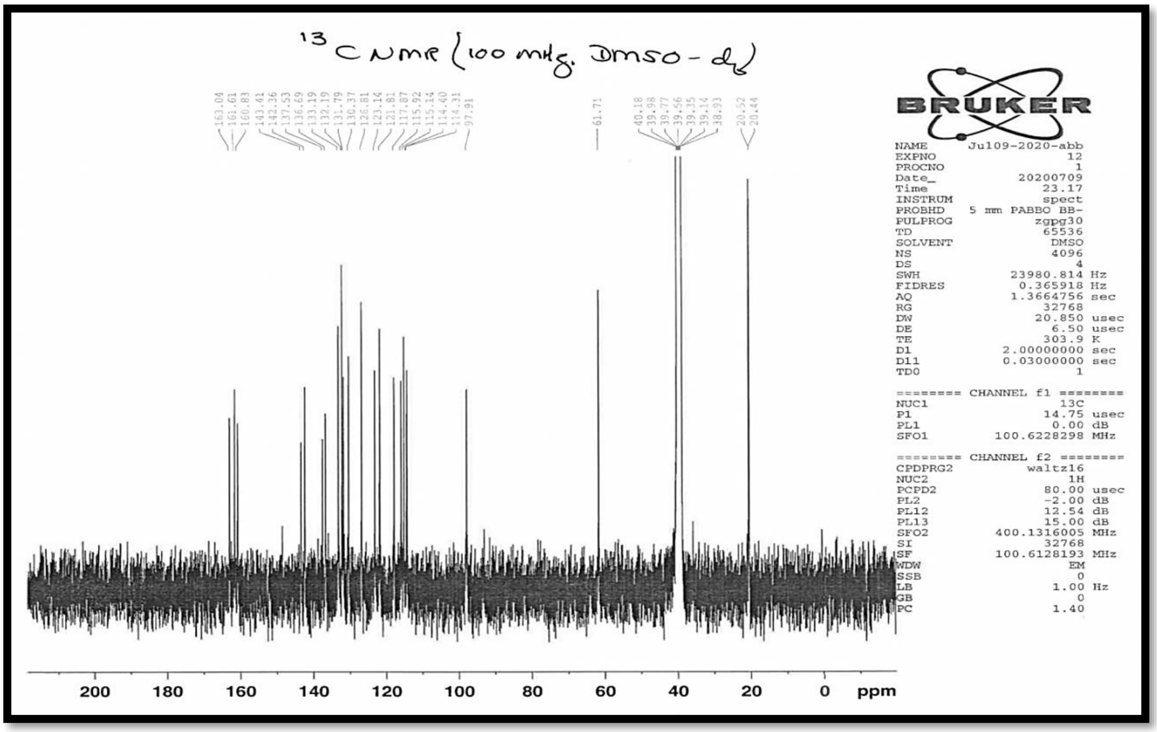

SI Fig. 85. A section of ^{13}C NMR spectrum (DMSO-d_6) of compound **6f**.

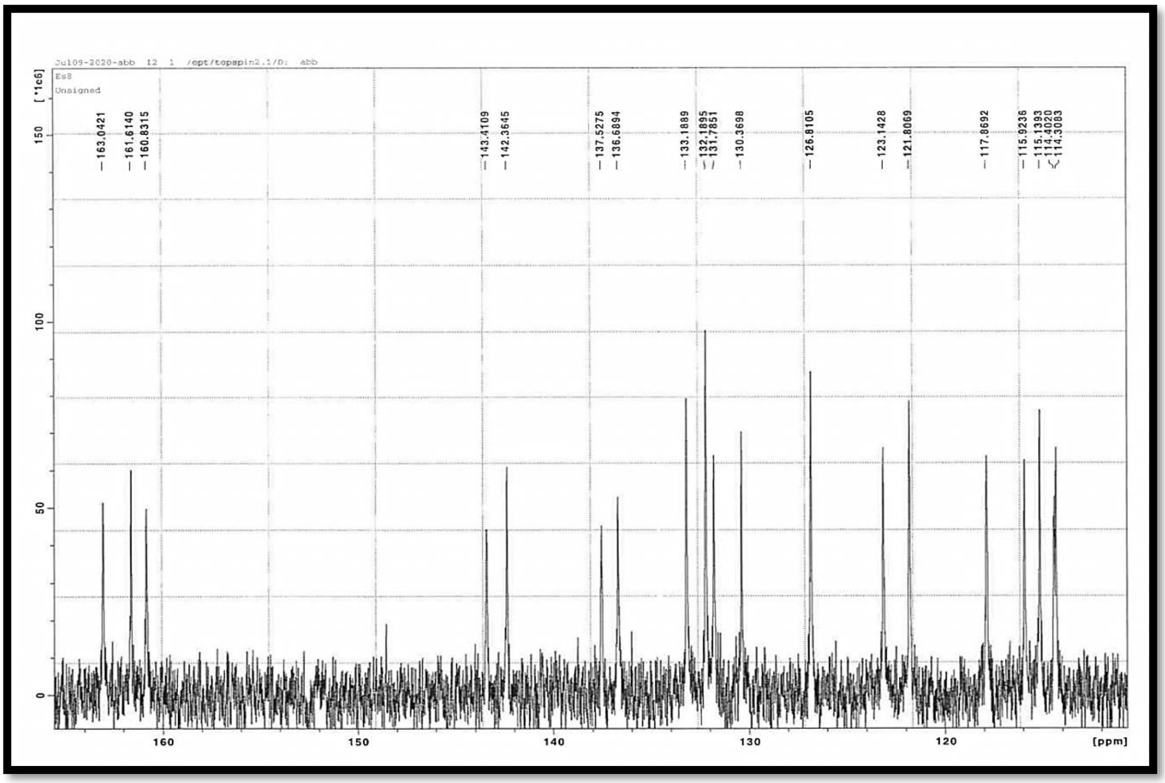

SI Fig. 86. ^{1}H ^{1}H Cosy spectrum (DMSO-d_6) of compound **6f**.

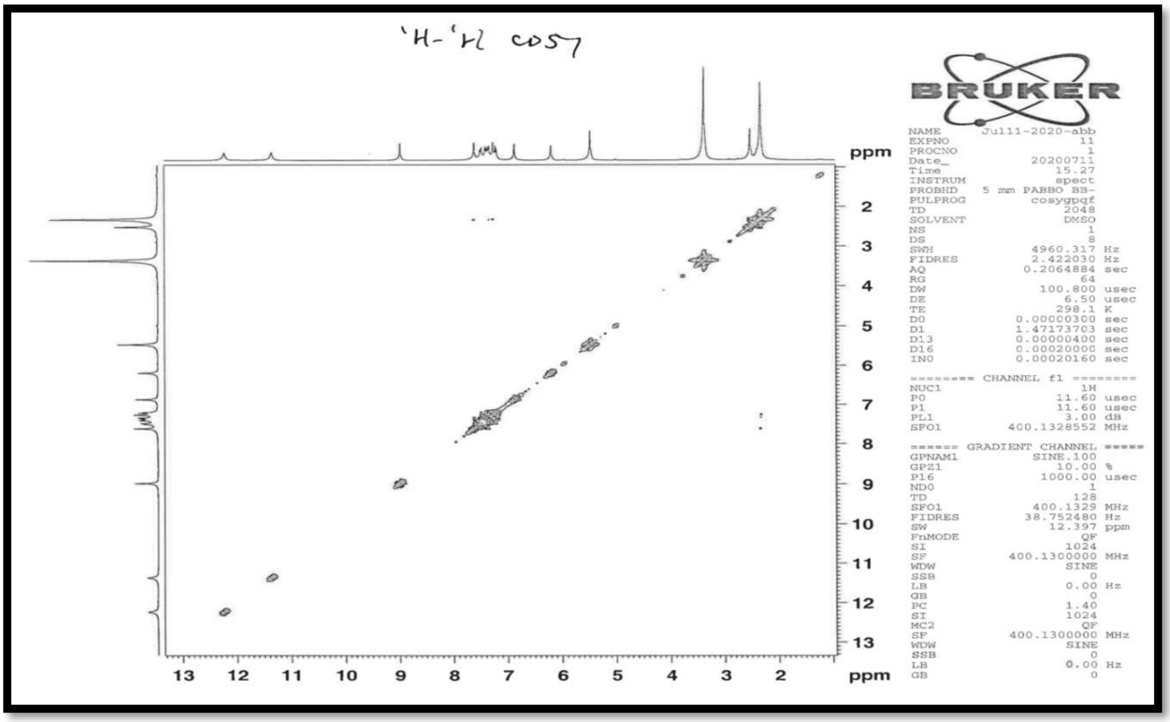

SI Fig. 87. ^{1}H ^{1}H Cosy spectrum (DMSO-d_6) of compound **6f**.

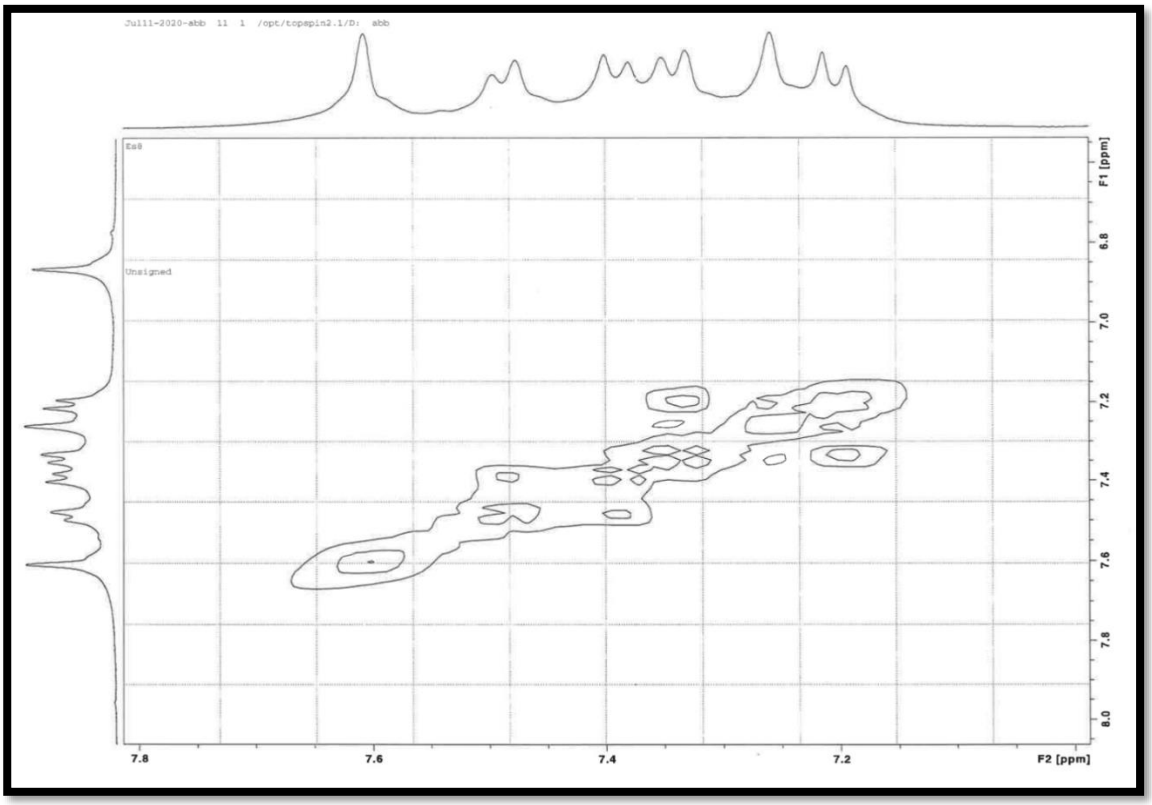

SI Fig. 88. ^{1}H ^{13}C HSQC spectrum (DMSO-d_6) of compound **6f**.

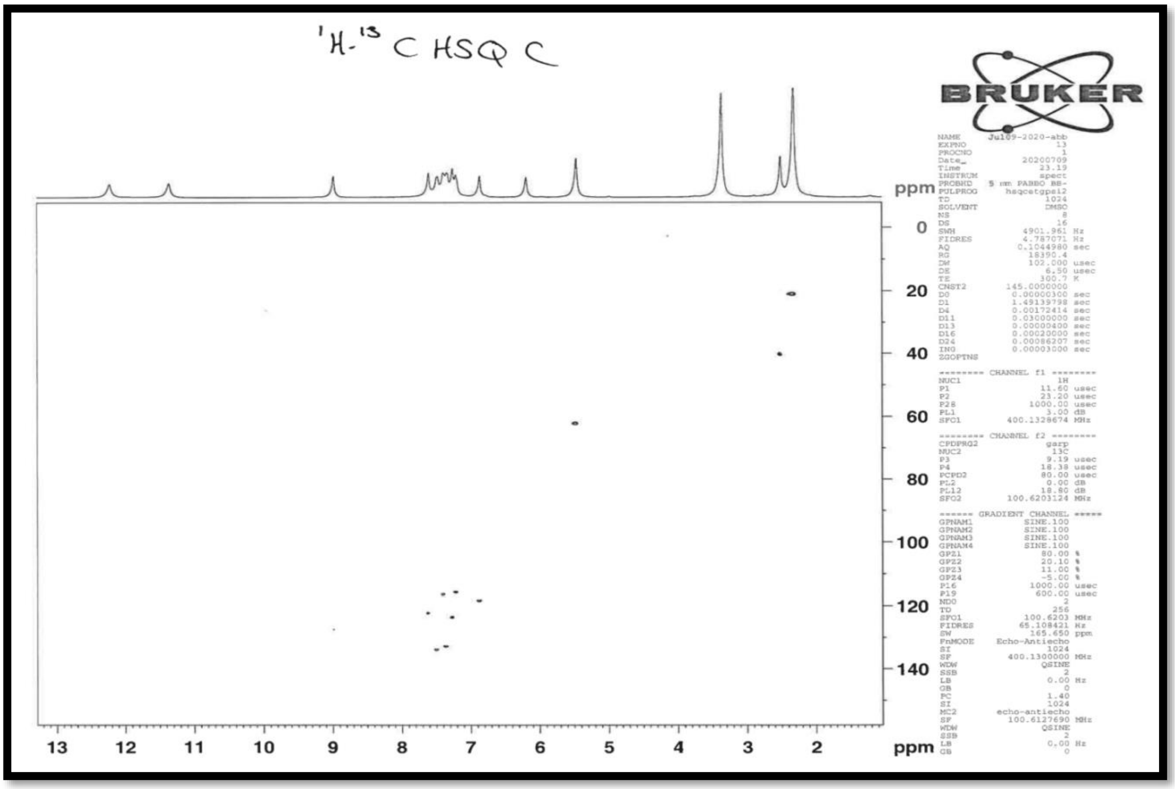

SI Fig. 89. ^{1}H ^{13}C HSQC spectrum (DMSO-d_6) of compound **6f**.

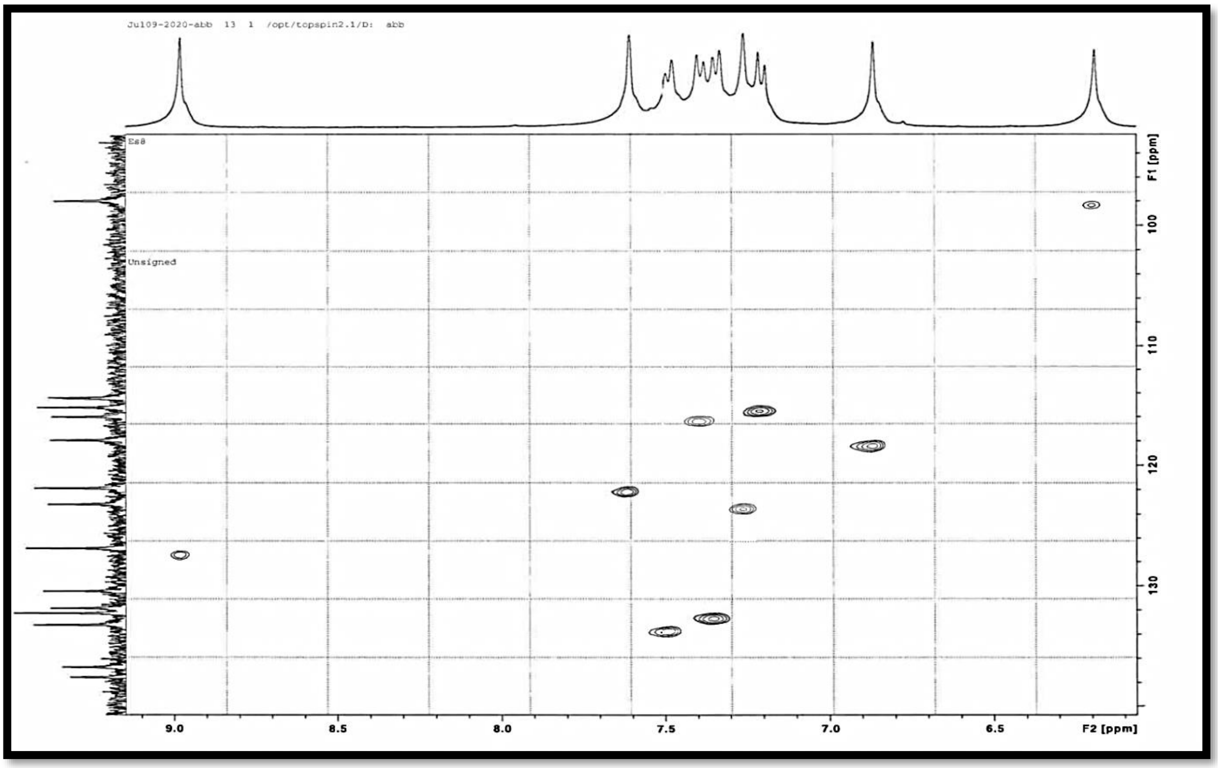

SI Fig. 90. ^{1}H ^{13}C HSQC spectrum (DMSO-d_6) of compound **6f**.

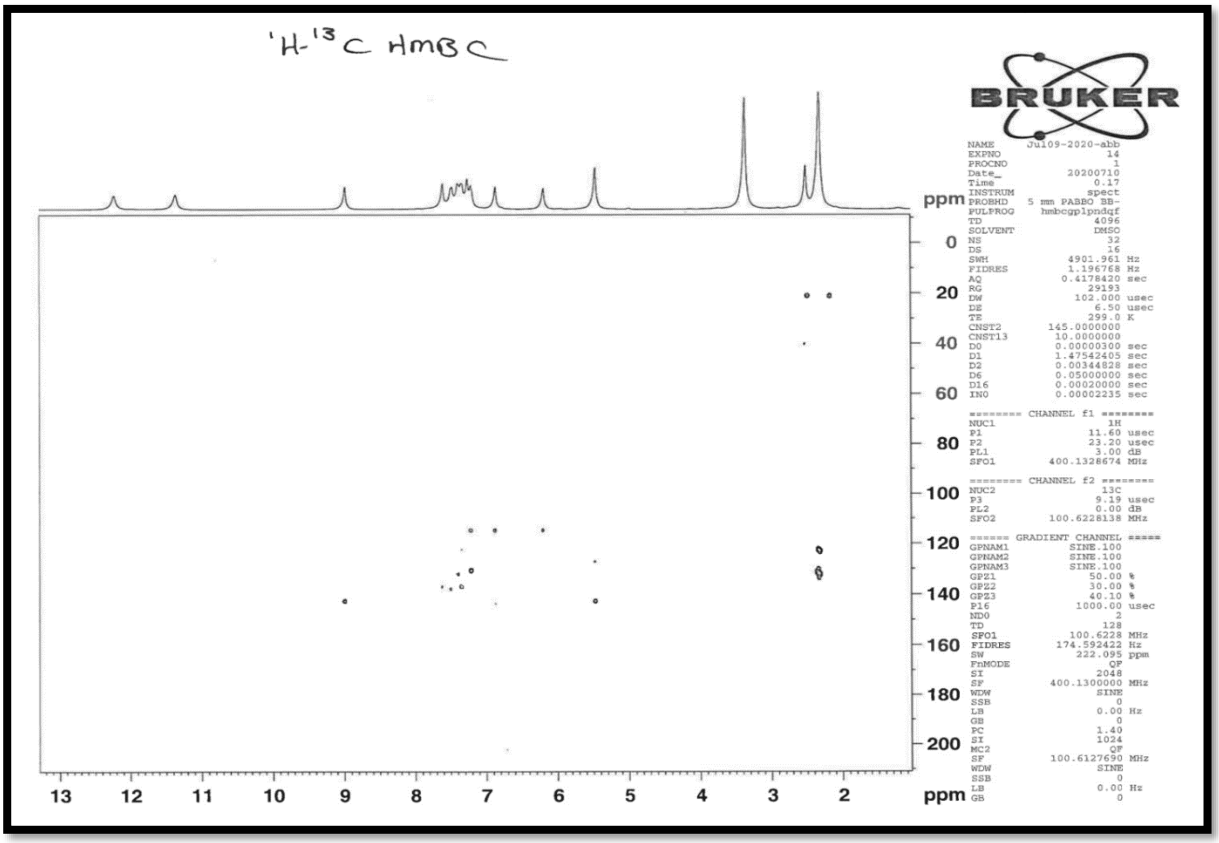

SI Fig. 91. ^{1}H ^{13}C HSQC spectrum (DMSO-d_6) of compound **6f**.

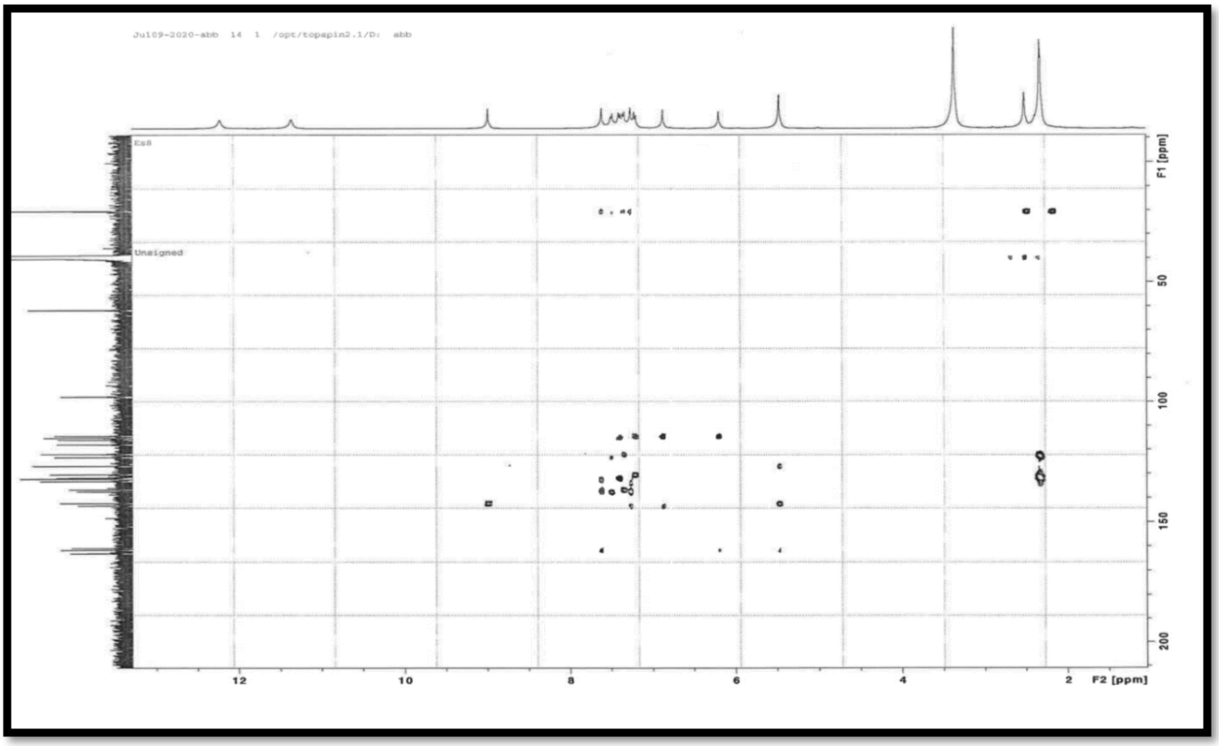

SI Fig. 92. ^{1}H ^{13}C HMBC spectrum (DMSO-d_6) for compound **6f**.

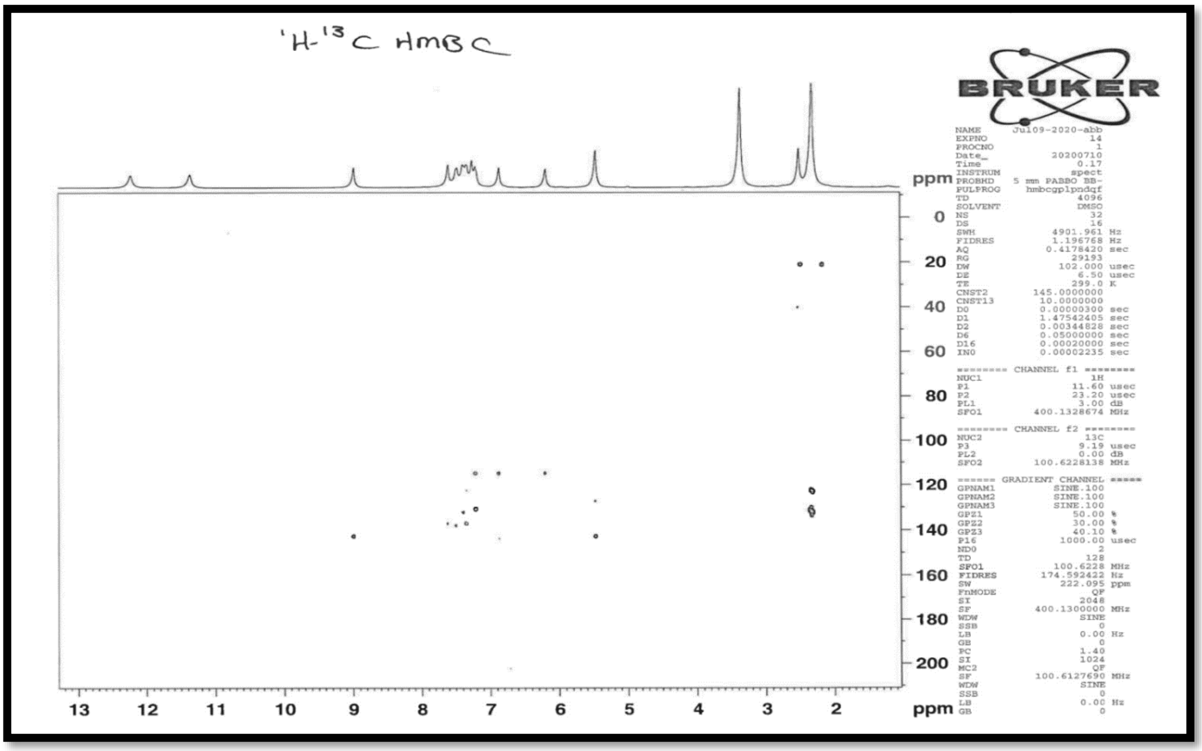

SI Fig. 93. ^{1}H ^{15}N HSQC spectrum (DMSO-d_6) of compound **6f**.

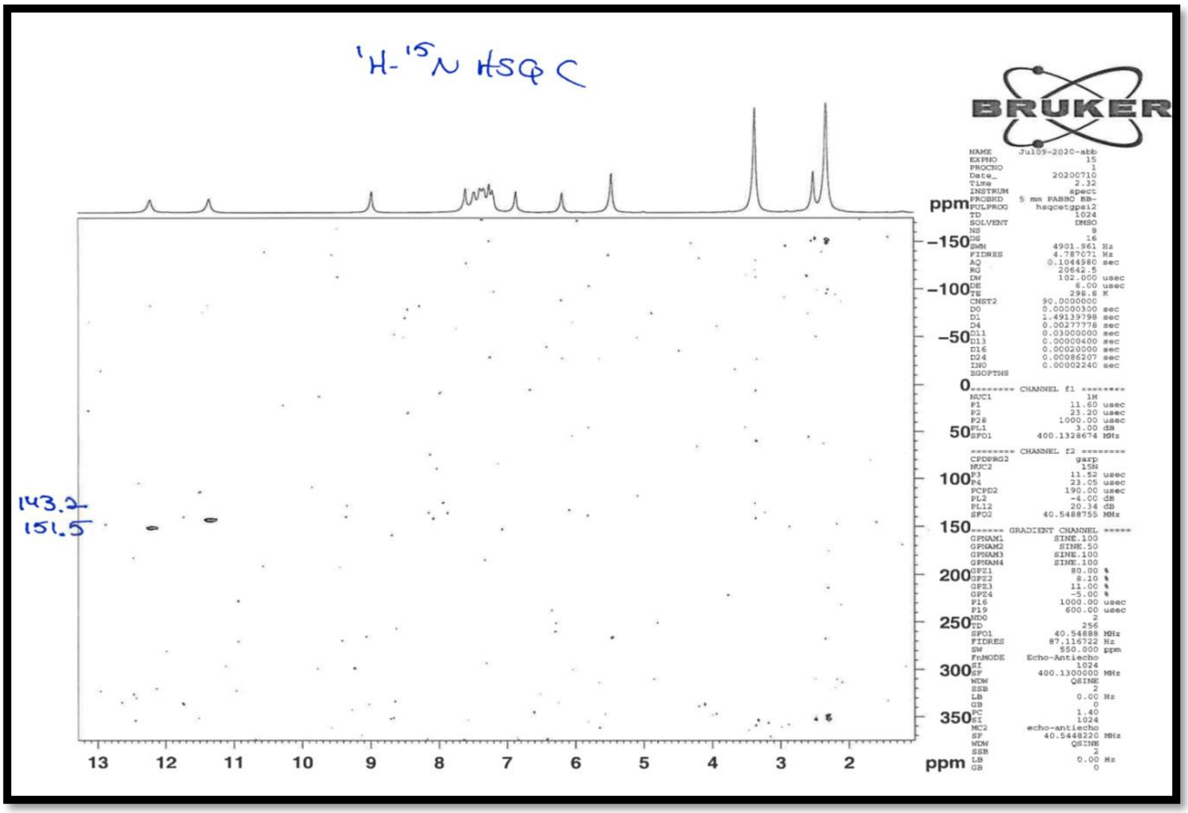

SI Fig. 94. ^{1}H ^{15}N HMBC spectrum (DMSO-d_6) of compound **6f**.

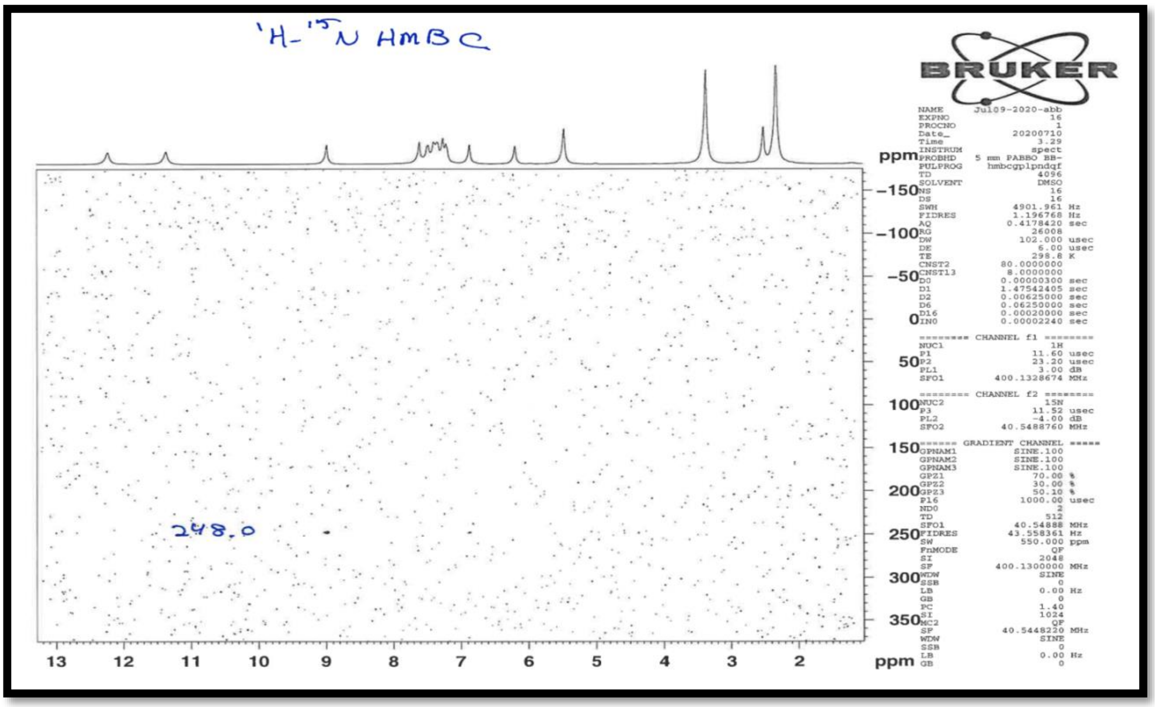

SI Fig. 95. The mass spectrum for compound **6f**.

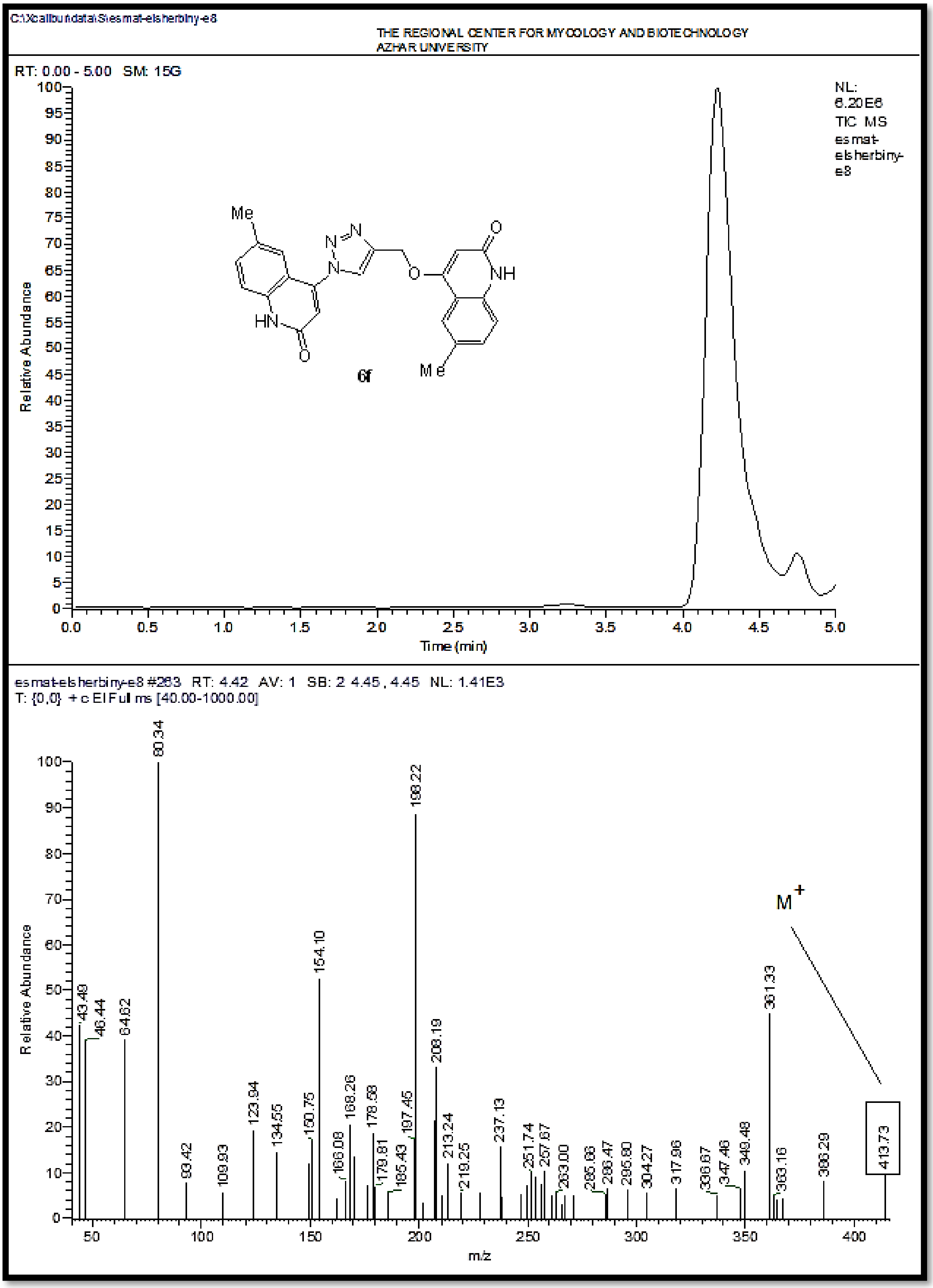

SI Fig. 96. ^{1}H NMR spectrum (DMSO-d_6) of compound **6g**.

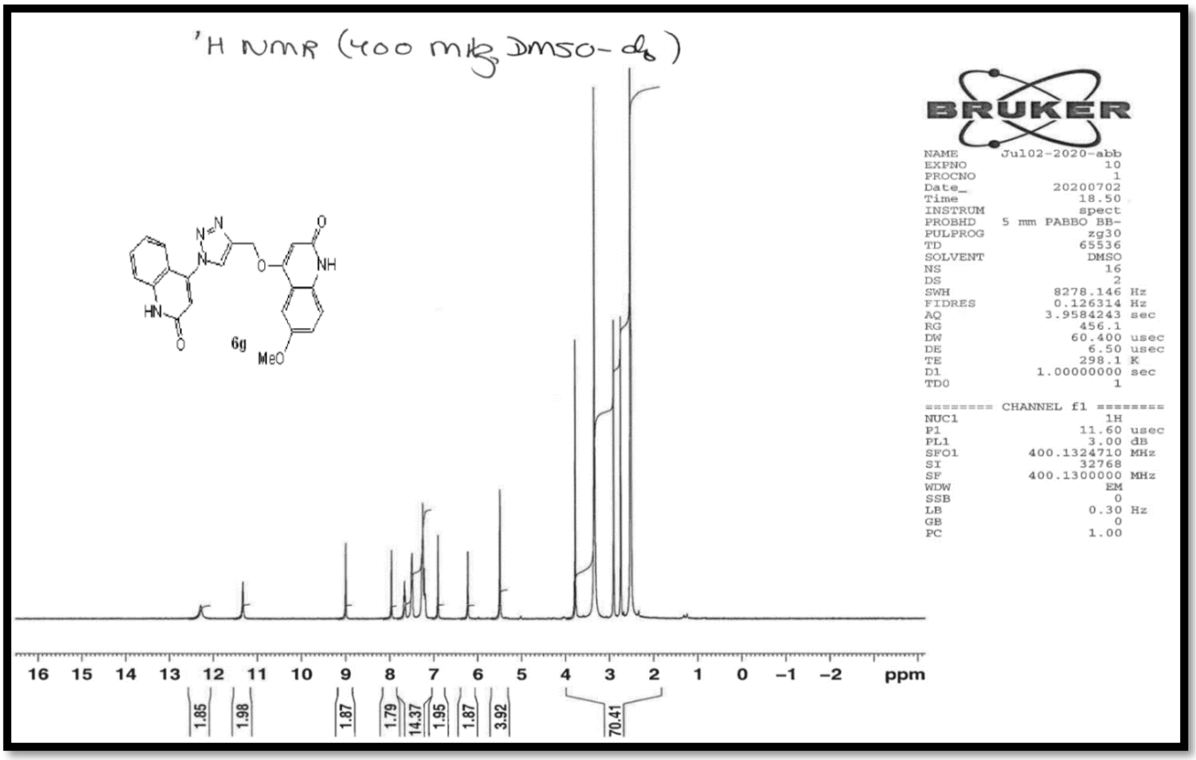

SI Fig. 97. A section of ^{1}H NMR spectrum (DMSO-d_6) of compound **6g**.

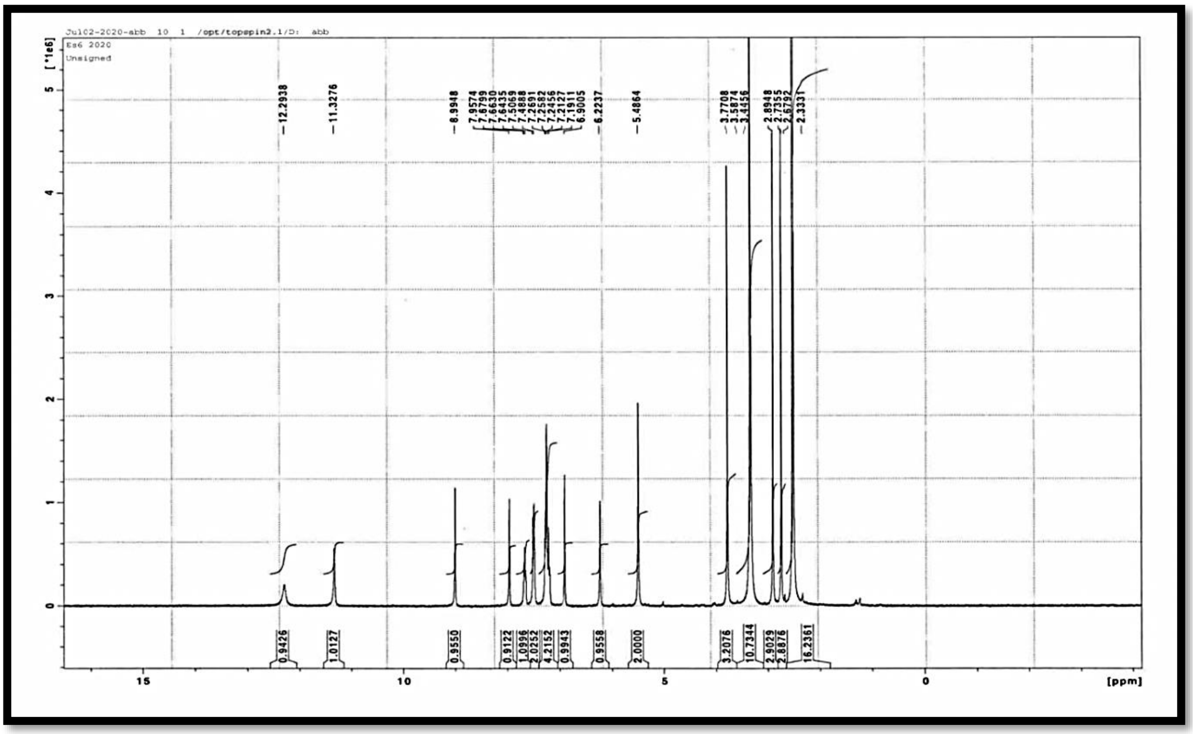

SI Fig. 98. ^{13}C NMR spectrum (DMSO-d_6) of compound **6g**.

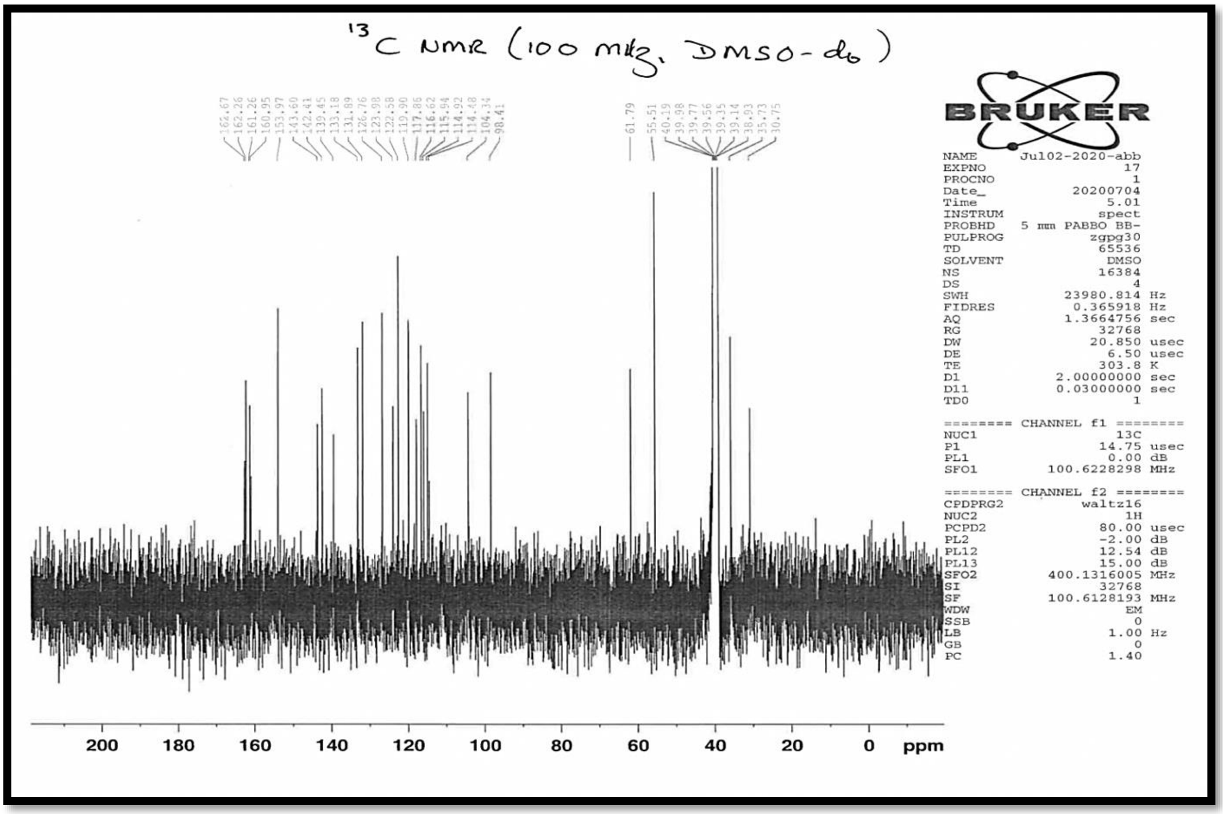

SI Fig. 99. ^{13}C NMR spectrum (DMSO-d_6) of compound **6g**.

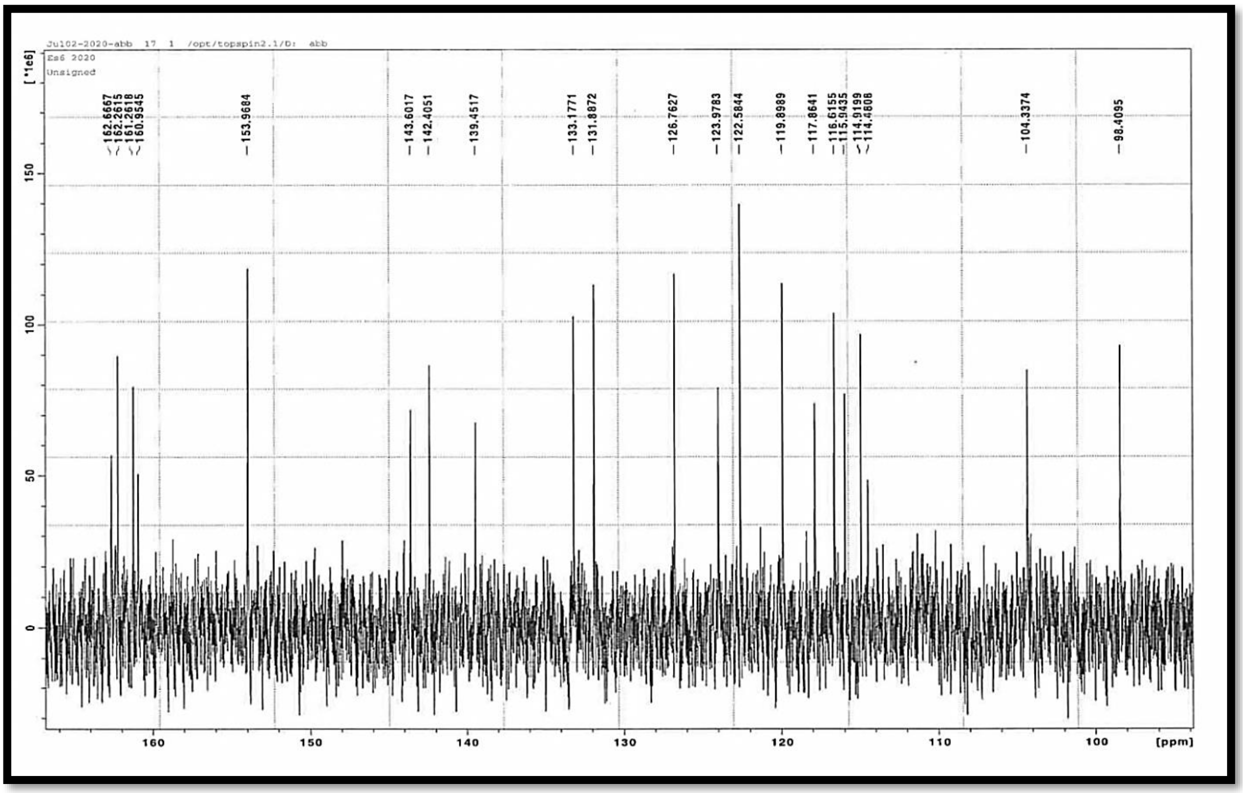

SI Fig. 100. ^{1}H ^{1}H Cosy spectrum (DMSO-d_6) of compound **6g**.

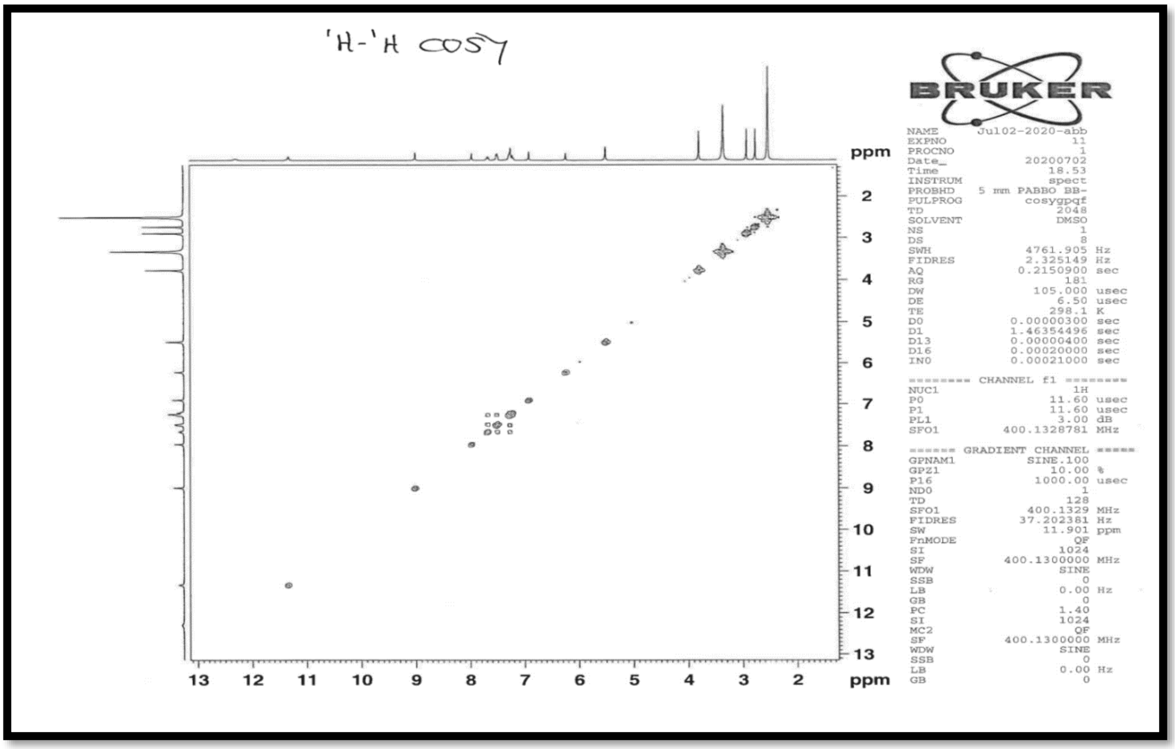

SI Fig. 101. A section of ^{1}H ^{1}H Cosy spectrum (DMSO-d_6) of compound **6g**.

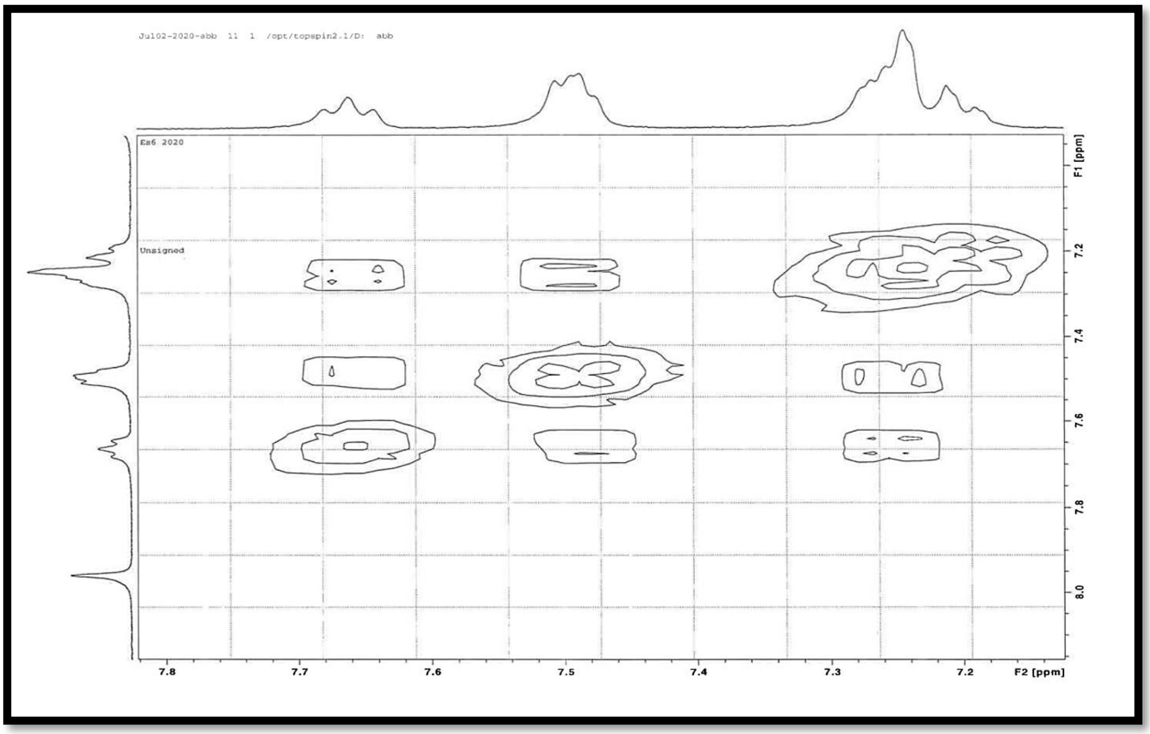

SI Fig. 102. ^{1}H ^{13}C HSQC spectrum (DMSO-d_6) of compound **6g**.

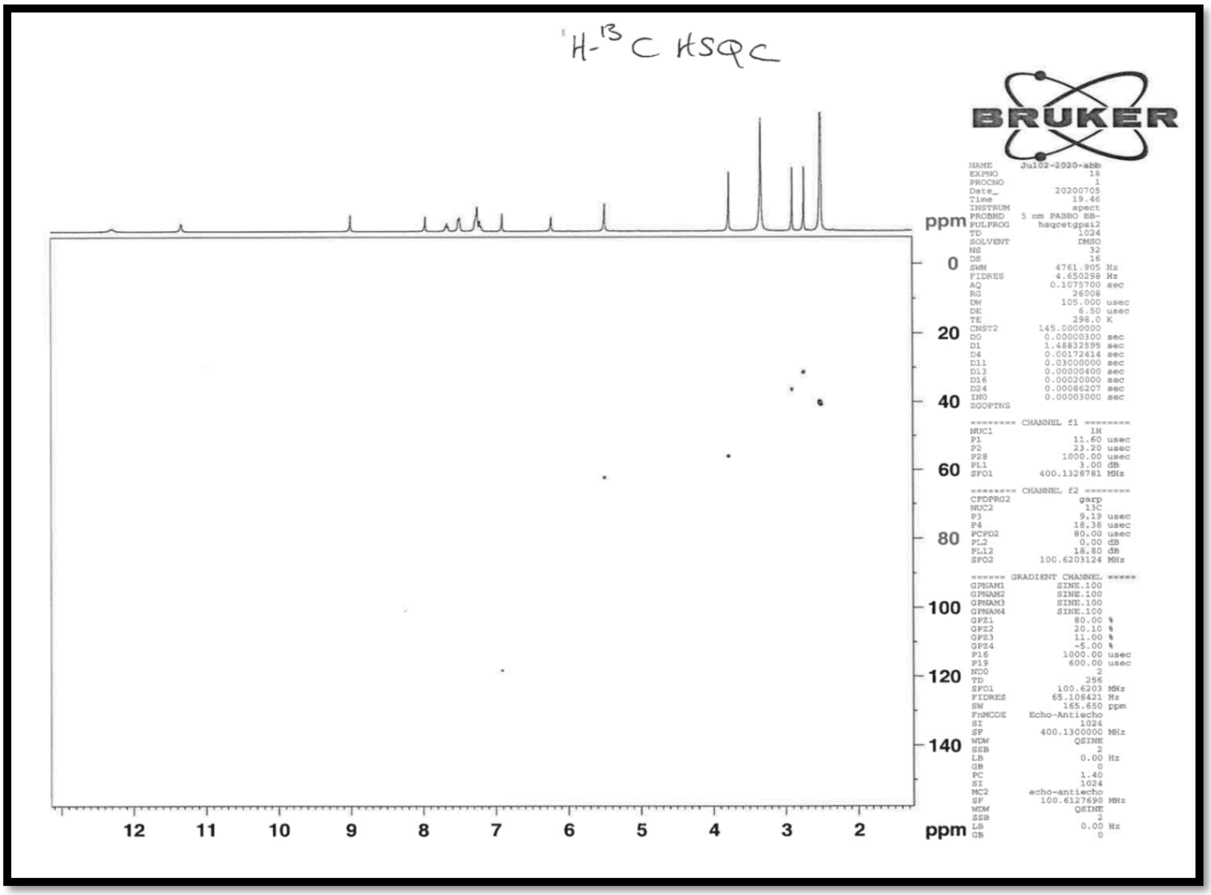

SI Fig. 103. ^{1}H ^{13}C HMBC spectrum (DMSO-d_6) of compound **6g**.

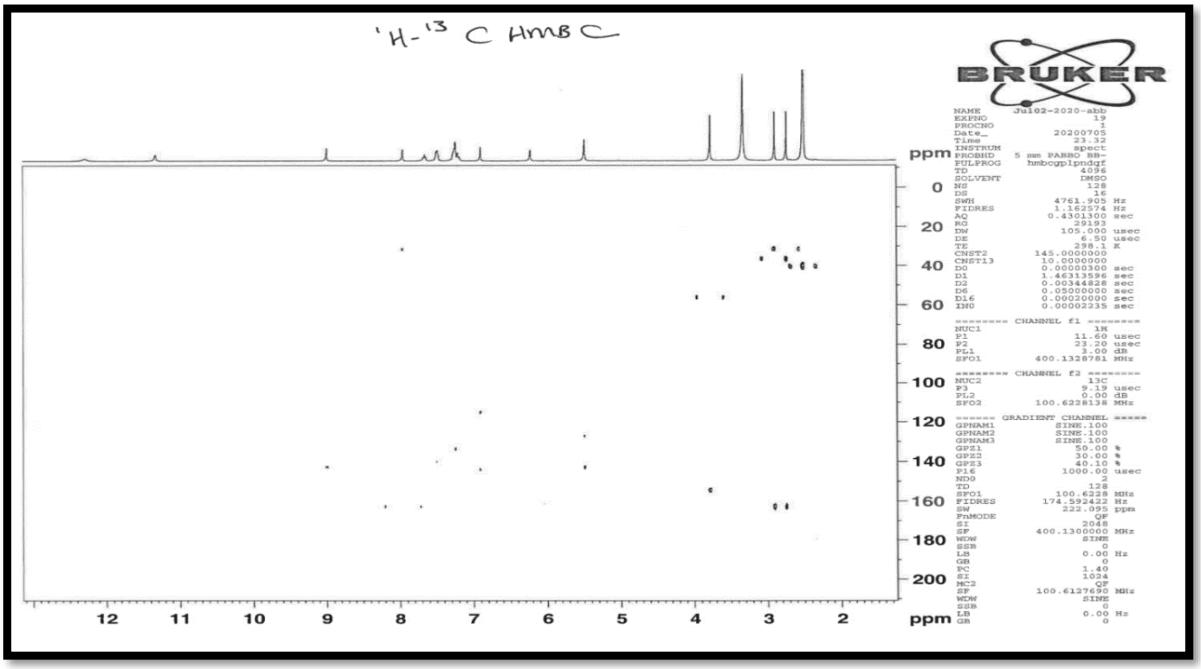

SI Fig. 104. ^{1}H ^{15}N HSQC spectrum (DMSO-d_6) of compound **6g**.

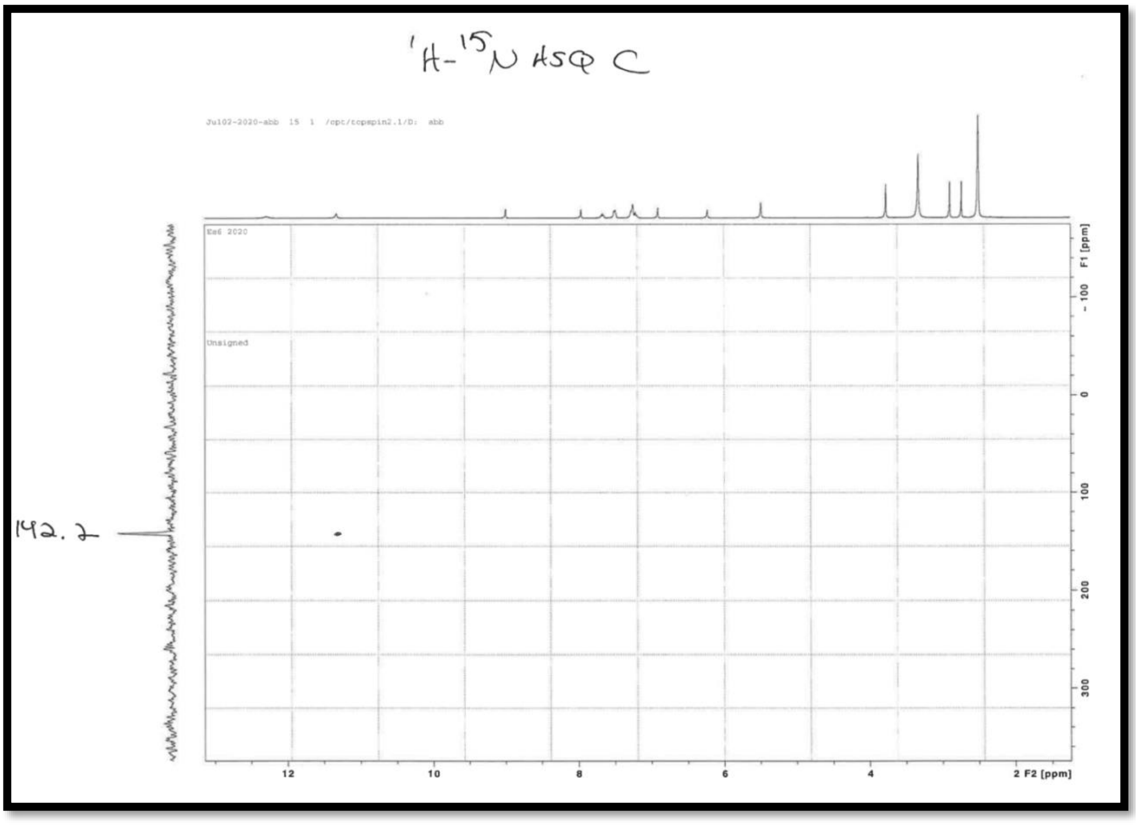

SI Fig. 105. ^{1}H ^{15}N HMBC spectrum (DMSO-d_6) of compound **6g**.

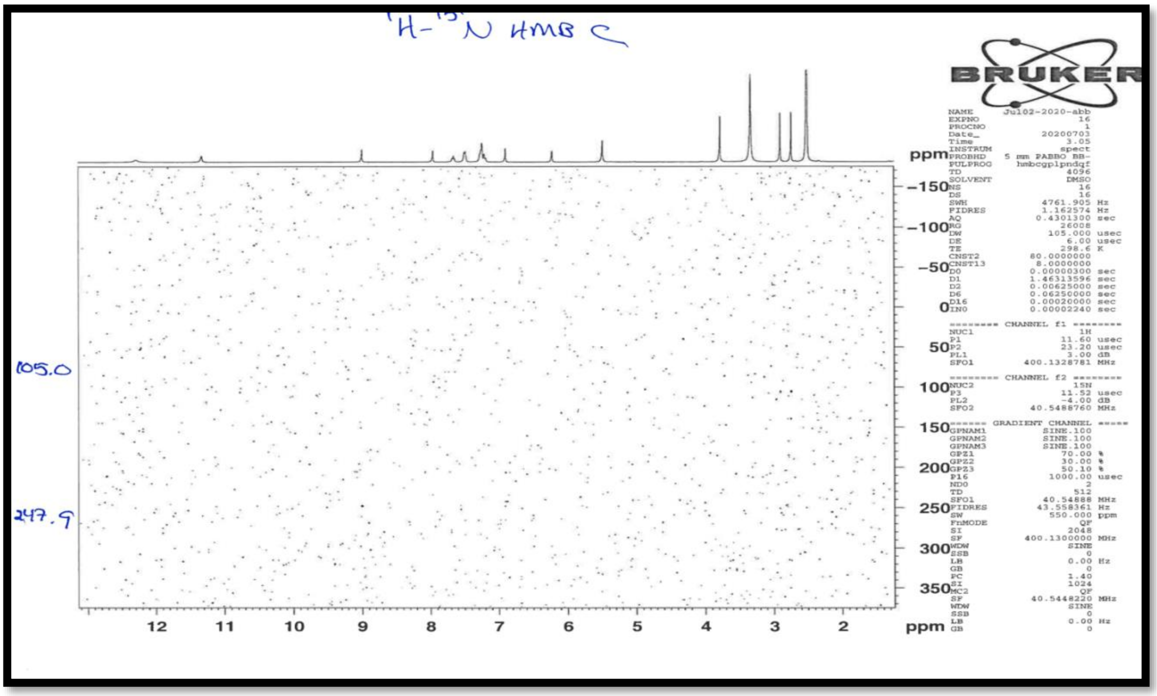

SI Fig. 106. A section of ^{1}H ^{15}N HMBC spectrum (DMSO-d_6) of compound **6g**.

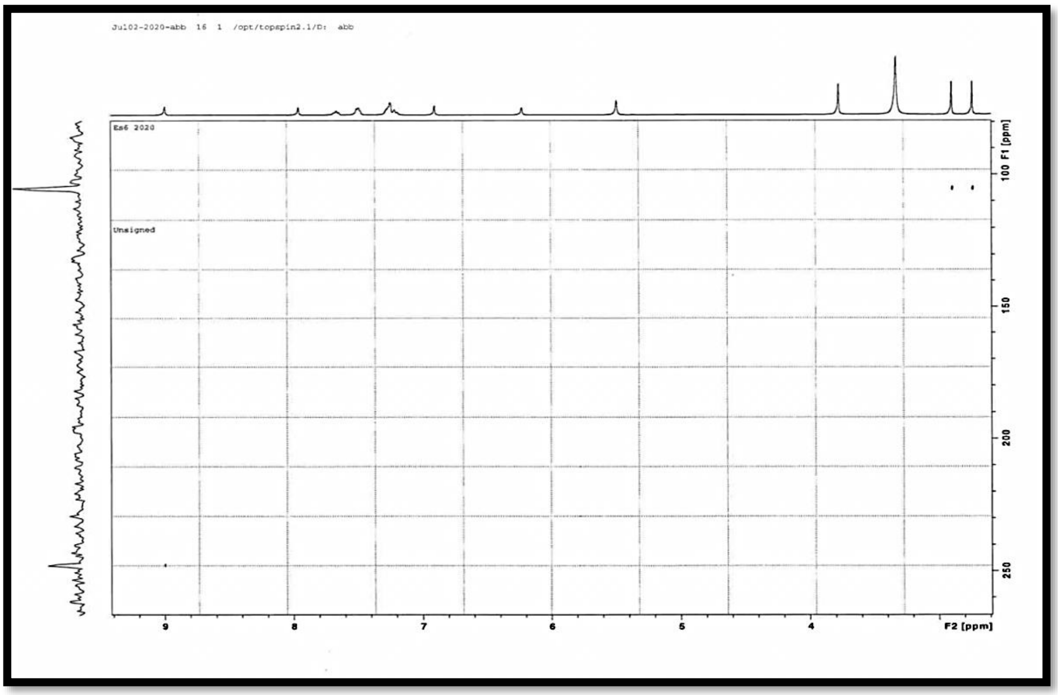

SI Fig. 107. The mass spectrum of compound **6g**.

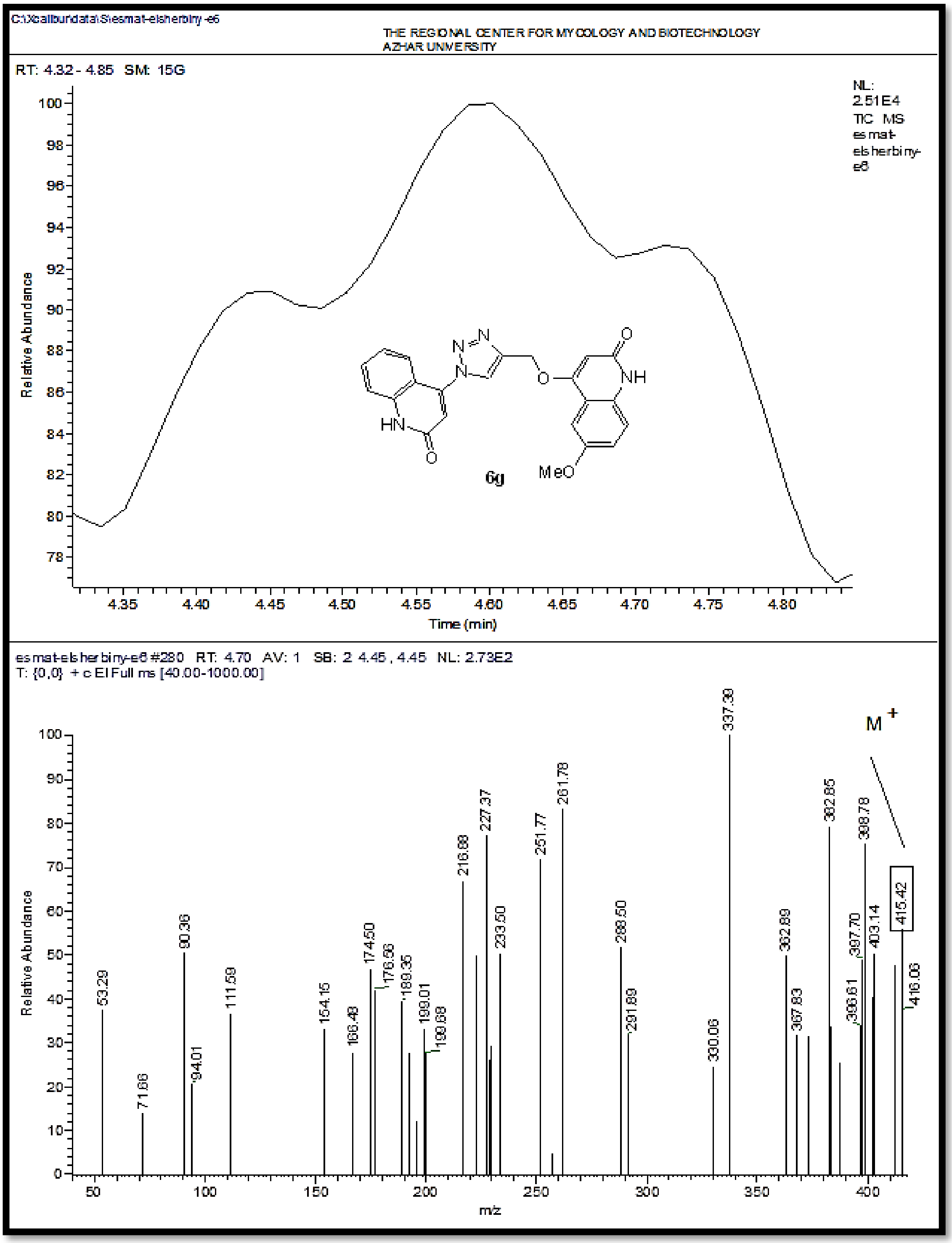

Biology Section

Table S1. Effect of compounds **6c** and NAC on the active Cell-based caspases-3, 8 and 9 in MOLT-4 cell line.

Code	IC_{50} nM		
	Casp-3	Casp-8	Casp-9
**6c**	16.31±0.6	35.57±1.3	96.24±2.7
**NAC**	46.83±1.3	11.96±0.8	135.2±3.8
**Control**	259.4±7.3	276.5±7.8	900.7±25

Table S2. Energy scores for the complexes formed by the tested compounds **6a-g** and the reference NAC in the active site of caspase-3 (PDB: 3GJQ)

Compounc	S score	Ligand-receptor interaction				ΔG (Kcal/mole)
		Residue	RMSD(ºA)	Type	Length (Å)	
**NAC**	-3.517	ASP135	1.71	H-donor	2.89	-6.5
		LYS137		H-acceptor	3.23	-0.8
**6a**	-5.258	LYS156	3.43	H-donor	3.20	-2.5
		LYS137		H- acceptor	3.24	-3.7
		GLY125		Pi-H	4.07	-0.9
		THR140		Pi-H	4.14	-0.6
		THR140		Pi-H	4.25	-0.7
**6b**	-5.290	LYS156	1.79	H-donor	3.14	-0.9
		LYS137		H- acceptor	3.09	-5.8
		THR140		Pi-H	3.91	-1.1
**6c**	-5.059	ASP135	2.60	H-donor	3.47	-1.5
		GLY125		Pi-H	4.49	-0.7
		GLY125		Pi-H	3.85	-1.4
**6d**	-5.061	LYS137	2.92	H- acceptor	2.98	-9.4
		THR140		Pi-H	3.97	-0.7
		PHE158		Pi-H	4.41	-0.7
**6e**	-5.034	LYS156	3.51	H-donor	3.16	-2.4
		LYS137		H- acceptor	3.14	-0.7
		THR140		Pi-H	4.05	-0.8
**6f**	-5.417	LYS156	3.68	H-donor	3.09	-3.5
		PHE158		H- acceptor	3.38	-0.6
		GLY125		Pi-H	3.77	-0.7
**6g**	-5.105	LYS156	2.78	H-donor	2.94	-5.4
		LYS137		H- acceptor	3.05	-6.8
		GLY125		Pi-H	3.89	-1.3
		THR140		Pi-H	3.91	-1.0

ΔG (Kcal/mole)[a]; The binding free energies

Table S3. Testicular MDA and Testicular TAC concentrations in testis of I/R rats treated with compounds **6a-g** and NAC

Groups	Testicular MDA (nmol/g tissue)	Testicular TAC (mmol/g tissue)
**Sham (control)**	54.29 ±3.17	4.96±0.47
**I/R**	121.40±4.15[ac]	[ac] 1.73±0.28
**NAC**	67.58±3.76[b]	4.45±0.40
**6a**	62.38±4.23[b]	[b]5.48±0.41
**6b**	61.84±4.77[b]	[b]4.62±0.33
**6c**	64.93±4.50[b]	[b]4.78±0.39
**6d**	117.50 ± 3.71[ac]	[ac] 1.89±0.29
**6e**	[ab]85.00±6.46	[ab]3.34±0.25
**6f**	[ac]117.00±3.46	[ac]1.73±0.25
**6g**	[abc]89.59±5.58	[ab]3.34±0.21

[I/R=ischemia reperfusion; NAC=N-acetylcysteine; MDA malondialdehyde; TAC=total antioxidant capacity]. Results represent the mean ± S.E.M (n= 6). [a] Significant (P < 0.05) difference from sham operated group, [b] Significant (P < 0.05) difference from I/R group, [c] Significant (P < 0.05) difference from NAC treated group.

Table S4. Testicular testosterone and TNF**α** concentrations in testis of I/R rats treated with compounds **6a-g** and **NAC**.

Groups	Testicular testosterone (mg/g tissue)	TNF-α(pg/ml)
**Sham control**	5.09±0.36	84.60±4.13
**IR**	2.03±0.18[ac]	240.60±5.57[ac]
**NAC**	4.29±0.30[b]	93.39±4.59[b]
**6a**	4.93±0.38[b]	106.40±4.44[b]
**6b**	5.07±0.45[b]	107.90±2.96[b]
**6c**	5.00±0.26[b]	106.70±4.75[b]
**6d**	2.67±0.25[ac]	234.50±6.15[ac]
**6e**	3.62±0.17[ab]	188.90±6.82[abc]
**6f**	1.83±0.14[ac]	237.90±4.52[ac]
**6g**	3.45±0.22[ab]	192.00±6.31[abc]

[I/R=ischemia reperfusion; NAC=N-acetylcysteine; TNF-α=Tumor necrosis factor]. Results represent the mean ± S.E.M (n= 6). [a] Significant (P < 0.05) difference from sham operated group, [b] Significant (P < 0.05) difference from I/R group, [c] Significant (P < 0.05) difference from NAC treated group.

Table S5. Caspase-3 level in serum of testicular I/R rats treated with compounds **6a-g** and **NAC**.

Groups	Caspase-3 (ng/ml)
**Sham control**	1.95±0.12

**IR**	15.78±1.14 ^{a}
**NAC**	2.54±0.22 ^{b}
**6a**	2.66±0.21 ^{b}
**6b**	2.94±0.28 ^{b}
**6c**	2.39±0.23 ^{b}
**6d**	15.28±1.51 ^{a}
**6e**	7.86±0.28 ^{ab}
**6f**	16.34±1.49 ^{a}
**6g**	6.99±0.24 ^{ab}

[I/R=ischemia reperfusion; NAC=N-acetylcysteine; TNF-α=Tumor necrosis factor]. Results represent the mean ± S.E.M (n= 6). [a] Significant ($P < 0.05$) difference from sham operated group, [b] Significant ($P < 0.05$) difference from I/R group, [c] Significant ($P < 0.05$) difference from NAC treated group.

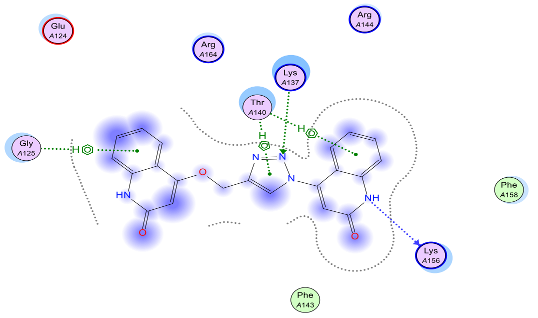

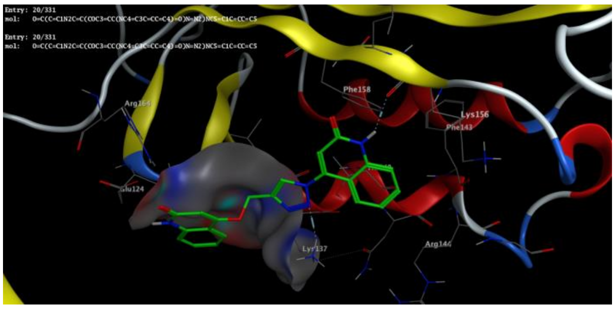

6a

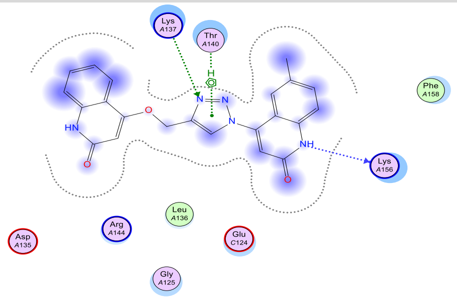

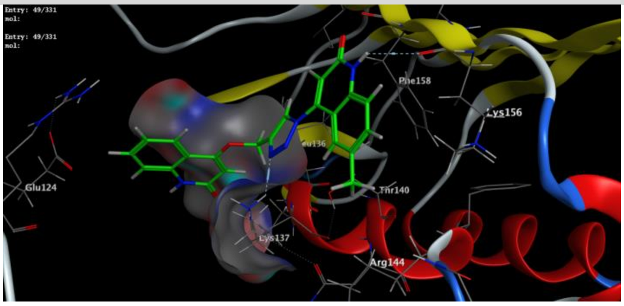

6b

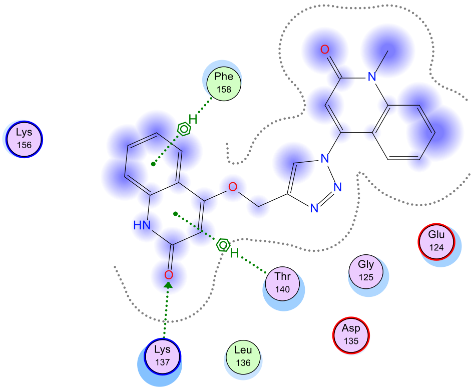
Phe 158
Lys 156
Glu 124
Gly 125
Thr 140
Asp 135
Lys 137
Leu 136

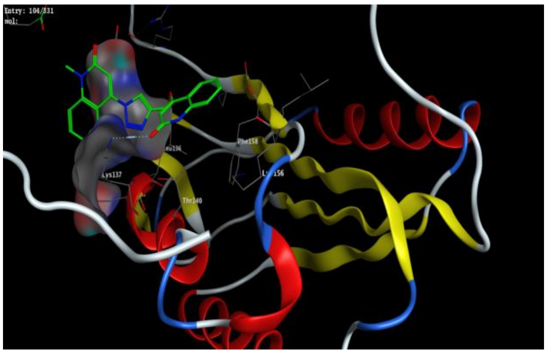

6d

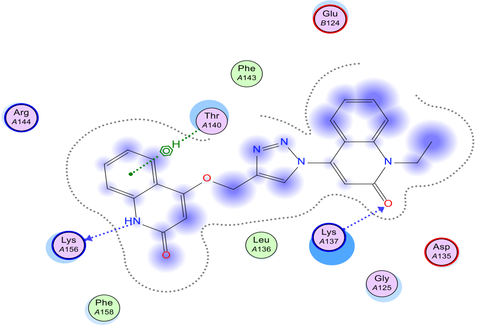
Glu B124
Phe A143
Arg A144
Thr A140
Lys A156
Leu A136
Lys A137
Asp A135
Gly A125
Phe A158

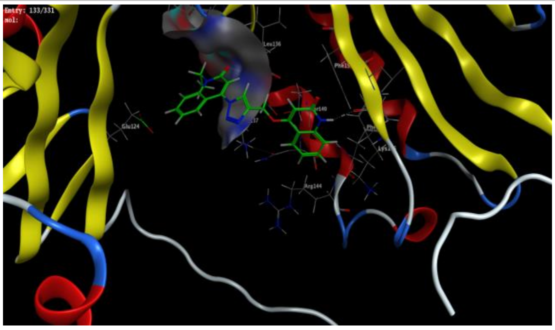

6e

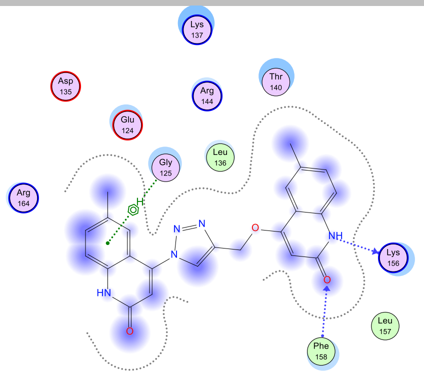
Lys 137
Asp 135
Thr 140
Arg 144
Glu 124
Leu 136
Gly 125
Arg 164
Lys 156
Leu 157
Phe 158

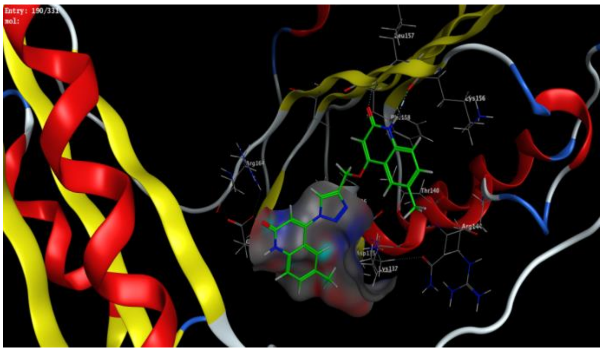

6f

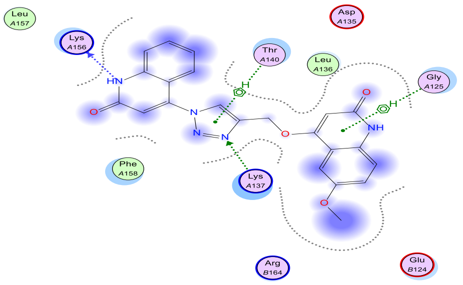
Leu A157
Lys A156
Asp A135
Thr A140
Leu A136
Gly A125
Phe A158
Lys A137
Arg B164
Glu B124

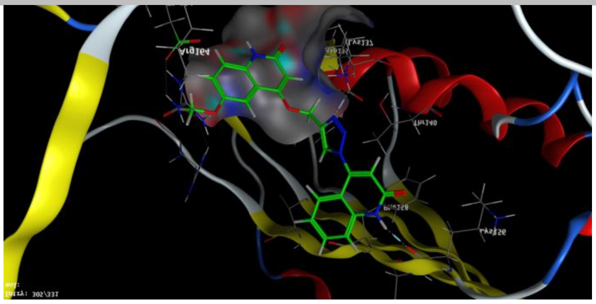

6g

Fig. 14. 2D and 3D diagrams illustrate the binding modes of the reference NAC and 6a-g interacted with the active site of caspase-3 (PDB: 3GJQ)

Experimental

1. Determination of testicular function

1.1. Determination of lipid peroxides in the form of Malondialdehyde (MDA) in testis

MDA, a reactive aldehyde that is a measure of lipid peroxidation, bone marrow cardiac contents of MDA were determined using the thiobarbituric acid method described by Buege and Aust (1978), which measures the thiobarbituric acid reactive substances concentration, sometimes referred to as MDA concentration.

Principle

This method measures the hepatic and ovarian MDA level which is the breakdown product of tissue lipid peroxides.

Procedures

- To a 0.5 mL of 10 % homogenate of the tissue sample, 0.5 mL of distilled water was added.
- The reagent was freshly prepared (26 mmol/L thiobarbituric acid, 0.92 mol/L trichloroacetic acid in 0.25 mol/L HCl) and 2 mL were added to each tube.
- The mixture was heated in a boiling water bath for 15 min.
- After cooling, the supernatant was removed by centrifugation at 5000 rpm for 10 min.
- The absorbance of the sample was determined at 535 nm against a blank using Bachman DU-64 spectrophotometer (Phoenix Equipment Inc, New York).
- 1,1,3,3–tetramethoxypropane was used as an external standard to prepare standard concentrations of MDA (1,2,4,6,8 and 10 nmol/mL) and the procedure was repeated to prepare a standard curve using 1,1,3,3–tetramethoxypropane instead of hepatic and ovarian homogenates. From this curve, the MDA concentration in the unknown sample was extrapolated from the corresponding absorbance using the regression line from the standard curve then multiplied in tissue and expressed as nmoL/g tissue.

Standard curve of MDA.

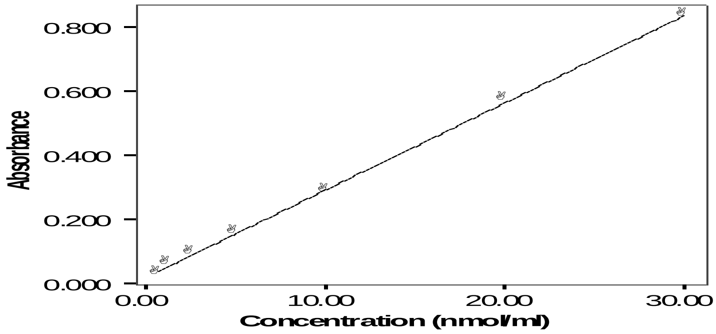

1.2.Determination of serum testosterone using ELISA kit

Principle:

This ELISA kit uses the Competitive-ELISA principle. The micro ELISA plate provided in this kit has been pre-coated with T. During the reaction, T in the sample or standard competes with a fixed amount of T on the solid phase supporter for sites on the Biotinylated Detection Ab specific to T. Excess conjugate and unbound sample or standard are washed from the plate, and Avidin conjugated to Horseradish Peroxidase (HRP) are added to each microplate well and incubated. Then a TMB substrate solution is added to each well. The enzyme-substrate reaction is terminated by the addition of stop solution and the color change is measured spectrophotometrically at a wavelength of 450 nm ± 2 nm. The concentration of T in the samples is then determined by comparing the OD of the samples to the standard curve.

Sample collection

Serum: Allow samples to clot for 2 hours at room temperature or overnight at 4°C before centrifugation for 20 min at 1000×g at 2-8°C. Collect the supernatant to carry out the assay. Blood collection tubes should be disposable and be non-endotoxin.

Plasma: Collect plasma using EDTA-Na_2 as anticoagulant. Centrifuge samples for 15 min at 1000×g at

2-8°C within 30 min of collection. Collect the supernatant to carry out the assay. Hemolysed samples are not suitable for ELISA assay.

Reagent preparation

1. Bring all reagents to room temperature (18~25°C) before use. Follow the Microplate reader manual for set-up and preheat it for 15 min before OD measurement.

2. Wash Buffer: Dilute 30 mL of Concentrated Wash Buffer with 720 mL of deionized or distilled water to prepare 750 mL of Wash Buffer. Note: if crystals have formed in the concentrate, warm it in a 40°C water-bath and mix it gently until the crystals have completely dissolved.

3. Standard working solution: Centrifuge the standard at 10,000×g for 1min. Add 1.0mL of Reference Standard &Sample Diluent, let it stand for 10min and invert it gently several times. After it dissolves fully, mix it thoroughly with a pipette. This reconstitution produces a working solution of 20 ng/mL. Then make serial dilutions as needed. The recommended dilution gradient is as follows: 20, 10, 5, 2.5, 1.25, 0.62, 0.31, 0 ng/mL.

Dilution method: Take 7 EP tubes, add 500uL of Reference Standard & Sample Diluent to each tube. Pipette 500uL of the 20 ng/mL working solution to the first tube and mix up to produce a 10 ng/mL working solution. Pipette 500uL of the solution from the former tube to the latter one according to this step. The illustration below is for reference. Note: the last tube is regarded as a blank. Don't pipette solution into it from the former tube.

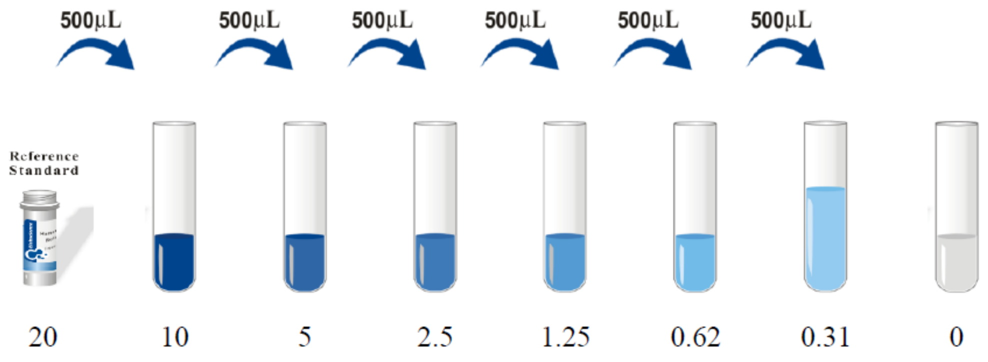

4. Biotinylated Detection Ab working solution: Calculate the required amount before the experiment (50μL/well). In preparation, slightly more than calculated should be prepared. Centrifuge the stock tube before use, dilute the 100× Concentrated Biotinylated Detection Ab to 1×working solution with Biotinylated Detection Ab Diluent.

5. Concentrated HRP Conjugate working solution: Calculate the required amount before the experiment (100μL/well). In preparation, slightly more than calculated should be prepared. Centrifuge the stock tube before use, dilute the 100×Concentrated HRP Conjugate to 1×working solution with Concentrated HRP Conjugate Diluent.

Calculation of results

Average the duplicate readings for each standard and samples. Plot a four-parameter logistic curve on log-log graph paper, with standard concentration on the x-axis and OD values on the y-axis.

If the samples have been diluted, the concentration calculated from the standard curve must be multiplied by the dilution factor. If the OD of the sample is under the lowest limit of the standard curve, you should re-test it with an appropriate dilution. The actual concentration is the calculated concentration multiplied by the dilution factor.

Typical data

As the OD values of the standard curve may vary according to the conditions of the actual assay performance (e.g. operator, pipetting technique, washing technique or temperature effects), the operator should establish a standard curve for each test. Typical standard curve and data is provided below for reference only.

**Concentration(ng/mL)**	**20**	**10**	**5**	**2.5**	**1.25**	**0.62**	**0.31**	**0**
**OD**	**0.269**	**0.545**	**0.740**	**1.006**	**1.299**	**1.608**	**1.919**	**2.426**

Reference values

Samples from different species were evaluated for the presence of T in this assay.

Sample type	Reference range of T in different species(ng/mL)						
	Human	Female Rat	Female Mouse	Chicken	Porcine	Sheep	Cattle
Serum(n=10)	1.43-9.69	4.97-6.58	0.36-0.62	4.61-8.45	0.75-1.99	0.94-2.72	0.85-1.00
Plasma(EDTA)(n=10)	0.69-8.05	1.01-1.08	ND	1.51-2.53	0.30-0.97	0.77-3.73	0.83-1.44
Urine(n=5)	3.88-21.05	-	-	-	-	-	-
Saliva(n=5)	0.84-1.67	-	-	-	-	-	-

The above values were all from normal healthy samples.

1.3.Determination of Total Antioxidant Capacity (Testicular tissue)

Free radicals or reactive oxygen species (ROS) are produced during biochemical redox reactions as part of normal physiological cell metabolism (protection from infectious organisms) and as a response to environmental factors such as UV light, cigarette smoke, environmental pollutants, and g-radiation. Once formed, ROS attack cellular components causing damage to lipids, proteins, and DNA which can initiate numerous diseases.

Antioxidants in the sample reduce dark blue-green colored ABTS radical to colorless reduced ABTS form. The change of absorbance at 660 nm is related with total antioxidant level of the sample. The assay is calibrated with a stable antioxidant standard solution which is traditionally named as Trolox Equivalent that is a vitamin E analog.

PRINCIPLE:

The determination of the antioxidative capacity is performed by the reaction of antioxidants in the sample with a defined amount of exogenously provide hydrogen peroxide (H_2O_2) The antioxidants in the sample eliminate a certain amount of the provided hydrogen peroxide. The residual H2O2 is determined

colorimetrically by an enzymatic reaction which involves the conversion of 3,5,dichloro–2–hydroxy benzensulphonate to a colored product.

Reagents

**1.**	Substrate (H_2O_2)<br>**(Dilute 1000 times before use)**
**2.**	Chromogen
**3.**	Enzyme – Buffer

Procedure:

* Dilute R11000 times immediately before use (10 μl R1 + 10 ml d. Water mix Discard after use.

*Working Reagent: mix equal volumes of R2 and R3 immediately before use.

*Dilute sample if necessary

	Blank ml	Sample ml
d. H_2O	0.02	-
Sample	-	0.02
R1 (substrate)	0.50	0.50
Mix well. Incubate 10 min at 37°C then add:		
Working reagent	0.5	0.5
Mix well . Incubate 5 min. at 37°C		
Read immediately the absorbances of blank (A_B) and sample (A_{SA}) against d. water at 505 (500 – 510 nm) . Linearity up to 2 mM / L .		

CALCULATION:

Total Antioxidant concentration

Mm/L = AB - ASA x 3.33

2. Assay of caspase-3, 8 and 9 inhibition

A. *General Considerations & Reagent Preparations*

• After thawing, store the 2X Reaction Buffer at 4oC. Aliquot enough 2X Reaction Buffer for the number of assays to be performed. Add DTT to the 2X Reaction Buffer immediately before use (10 mM final concentration: add 10 µl of 1.0 M DTT stock per 1 ml of 2X Reaction Buffer).

• Protect DEVD-AFC from light.

• Reconstitute the Active Caspase-3, 8 or 9 in 550 µl 2X Reaction Buffer. Aliquot and immediately store at –70°C.

B. ***Assay Procedure***

1. Prepare testing sample in d. H_2O to a final volume of 50 µl/well. Add 5 µl of Active Caspase-3, 8 or 9. Mix well. Prepare a background control by omitting the Active Caspase-3, 8 or 9 from the reaction mixture. Prepare a positive inhibition control by adding 1 µl of the Caspase-3, 8 or 9 Inhibitor (provided with the kit) instead of your testing inhibitor.

2. Prepare a Master Mix for each assay containing the follows: 45 µl 2X Reaction Buffer (containing 10 mM DTT) 5 µl 1 mM DEVD-AFC substrate (50 µM final concentration)

3. Mix well and add 50 µl of the Master Mix to each well to start the reaction.

4. Incubate at 37°C for 0.5-1 hour.

5. Read samples in a fluorescence plate reader equipped with a 400-nm excitation filter and 505-nm emission filter. Comparison of the fluorescence intensity of the testing samples with samples containing no inhibitors to determine the inhibition efficiency of the testing inhibitors.

3. **Assay of cytochrome *C***

- **Equilibrate all materials and prepared reagents to room temperature prior to use.**
- **It is recommended to assay all standards, controls and samples in duplicate.**

13.1. Prepare all reagents, working standards, and samples as directed in the previous sections. Determine the number of microplate strips required to test the desired number of samples plus appropriate number of wells needed for running blanks and standards. Predilute samples before starting the test procedure with one-part sample to one-part 1X Assay Buffer.

13.2. Wash the microplate twice with approximately 400 μL 1X Wash Buffer per well with thorough aspiration of microplate contents between washes. Allow the 1X Wash Buffer to remain in the wells for about 10 - 15 seconds before aspiration. Take care not to scratch the surface of the microplate.

13.3. After the last wash step, empty wells and tap microplate on absorbent pad or paper towel to remove excess 1X Wash Buffer. Use the microplate strips immediately after washing. Alternatively the microplate strips can be placed upside down on a wet absorbent paper for not longer than 15 minutes. Do not allow wells to dry.

13.4. Add 100 μL of prepared standards (including the no standard blank control) to the appropriate wells.

13.5. Add 100 μL of samples to appropriate wells.

13.6. Add 50 μL of 1X Biotin Conjugated Antibody to all wells.

13.7. Cover with adhesive film and incubate at room temperature (18° to 25°C) for 2 hours.

13.8. Remove adhesive film and empty wells. Wash microplate strips 3 times according to step 13.2. Proceed immediately to step 13.9. Add 100 μL of 1X Streptavidin-HRP to all wells, including the blank wells.

13.10. Cover with an adhesive film and incubate at room temperature (18° to 25°C) for 1 hour.

13.11. Remove adhesive film and empty wells. Wash microplate strips 3 times according to step 13.2. Proceed immediately to the next step.

13.12. Pipette 100 μL of TMB Substrate Solution to all wells.

13.13. Incubate the microplate strips at room temperature (18 to 25°C) for 10 minutes. Avoid direct exposure to intense light.

Note: The color development on the plate should be monitored and the substrate reaction stopped (see step

13.14) before the signal in the positive wells becomes saturated. Determination of the ideal time period for color development should be done individually for each assay. It is recommended to add

the stop solution when the highest standard has developed a dark blue color. Alternatively, the color development can be monitored by the ELISA reader at 620 nm. The substrate reaction should be stopped as soon as Standard 1 has reached an OD of 0.9 - 0.95.

13.14. Stop the enzyme reaction by adding 100 µL of Stop Solution into each well.

Note: It is important that the Stop Solution is mixed quickly and uniformly throughout the microplate to completely inactivate the enzyme. Results must be read immediately after the Stop Solution is added or within one hour if the microplate strips are stored at 2 - 8°C in the dark.

13.15. Read absorbance of each microplate on a spectrophotometer using 450 nm as the primary wavelength (optionally 620 nm as the reference wavelength; 610 nm to 650 nm is acceptable). Blank the plate reader according to the manufacturer's instructions by using the blank wells. Determine the absorbance of both the samples and the standards.

Note: In case of incubation without shaking the obtained O.D. values may be lower than indicated below. Nevertheless the results are still valid.

4. Histopathological Investigation

4.1.Material and methods

Multiple testicular specimens were fixed in Bouin's solution for about 24 hour, dehydrated in an alcohol grades, cleaned with xylene and then embedded in paraffin wax. Tissue sections (about six µm) were stained by haematoxylin–eosin (H&E) and were studied by an experienced histologist. In the current study, we used Cosentino's score for semi-quantitation of pathological changes on different seminiferous tubules in the whole groups by grading these tubules between one and four, and the mean scores were taken by dividing the totality to the whole number of testicular tubules . By using Johnson’s scoring system, the effect of ischemia on the spermatogenesis can be studied. The grades were counted one to ten, and the total scores were taken by dividing the totality to the whole number of testicular tubules.

4.2.Histopathological grading score (Cosentino's score)

Grade Characteristics

I   Normal testicular structure

II less orderly germinal cells with closely packed seminiferous tubules

III Anarchic sloughed germinal cells, with less pyknotic nuclei size and less distinct seminiferous tubule borders

IV   Closely packed seminiferous tubules and coagulative necrosis

Note: Semi-quantitation of pathological changes on different seminiferous tubules in the whole groups by grading the tubules between 1 and 4, and the mean scores were obtained by dividing the totality to the whole number of testicular tubules.

4.3.Spermatogenesis grading; Score Morphological picture

10 Normal spermatogenesis

9   less defect in spermatogenesis with multiple late spermatids, little disorganized epithelium

8 Less than five spermatozoa/tubule, less late spermatids

7 Absence of spermatozoa, absent late spermatids, more early spermatids

6 No spermatozoa, absence of late spermatids with little early spermatids

5 No spermatozoa or spermatids, more spermatocytes

4 No spermatozoa or spermatids, fewer spermatocytes

3 Only spermatogonia

2 No germinal cells, only Sertoli cells

1 No seminiferous epithelium

Note: By using Johnsen's scoring system, the effect of ischaemia on the spermatogenesis can be detected. The grades were from 1 to 10, and the total scores were taken by dividing to the whole number of testicular tubules.

5. **Molecular docking study**

- All conformers were subjected to energy minimization, all the minimizations were Performed with MOE until a RMSD gradient of 0.01 Kcal/mole and RMS (Root Mean Square) distance of 0.1 Å with MMFF94X force-field and the partial charges were automatically calculated.
- The obtained database was then saved as Molecular Data Base (MDB) file to be Used in the docking calculations.

Optimization of the target:

The X-ray crystallographic structure of the target caspase-3 enzyme (PDB: 3GJQ) obtained from Protein data bank. The compounds were docked on the active site the target enzyme.

The enzyme was prepared for docking studies by:

- The co-crystallized ligand, Moxifloxacin was deleted.
- Hydrogen atoms were added to the system with their standard geometry.
- The atoms connection and type were checked for any errors with automatic Correction.
- Selection of the receptor and its atoms potential were fixed.

Docking of the target molecules to caspase-3 enzyme active site

Docking of the target compounds was done using MOE-Dock software. The following methodology was generally applied:

- The enzyme active site file was loaded, and the Dock tool was initiated. The program specifications were adjusted to:

  - Dummy atoms as the docking site.

  - Triangle matcher as the placement methodology to be used.

  - London dG as Scoring methodology to be used and was adjusted to its default values.

- The MDB file of the ligand to be docked was loaded and Dock calculations were run automatically.
- The obtained poses were studied and the poses showed best ligand-enzyme interactions were selected and stored for energy calculations.
